# L-type voltage-gated Ca^2+^ channels control T cell killing via non-canonical Hedgehog signalling

**DOI:** 10.1038/s44319-026-00810-8

**Published:** 2026-06-08

**Authors:** Flavio Beke, Joachim Hanna, Chrysa Kapeni, Valentina Carbonaro, Nico Mueller, Sophie Trotter, Chandra Chilamakuri, Louise M O’Brien, Maike de la Roche

**Affiliations:** University of Cambridge, Cancer Research UK Cambridge Institute, Robinson Way, Cambridge, CB2 0RE UK

**Keywords:** Immunology, Membranes & Trafficking, Signal Transduction

## Abstract

Cytotoxic CD8^+^ T lymphocytes (CTLs) efficiently eliminate infected and cancerous cells throughout the body. T cell receptor (TCR)-induced Hedgehog signalling contributes to CTL-mediated killing, but how the pathway is activated downstream of the TCR is unknown. Here, we show that extracellular calcium (Ca^2+^) flux through L-type voltage-gated Ca^2+^ (Ca_v_1) channels at the plasma membrane downstream of the TCR drives induction of the Hedgehog transcription factor Gli1, which is important for CTL killing in vitro and in vivo. This previously unknown non-canonical Hedgehog pathway is independent of canonical signalling and represents a primary mechanism of Gli1 induction in naive CD8^+^ T cells, whereas CTLs can also activate Gli1 via MAPK. We further show that Ca_v_1 channel-controlled Gli1 induction is functionally important for CTL killing in mice and humans and other cytotoxic lymphocytes. Notably, killing capacity can be amplified using a small molecule Ca_v_1 agonist or by overexpressing a gain-of-function Ca_v_1 subunit. These findings suggest a strategy to improve cytotoxic lymphocyte function in the clinic, including in CAR T cell therapy.

## Synopsis

The Hedgehog transcription factor Gli1 is important for T cell killing and is induced downstream of the TCR through Ca_v_1 Ca^2+^ channels. Potentiation of Ca_v_1 function enhances the killing capacity of cytotoxic lymphocytes.

- Loss of Gli1 in murine CD8^+^ T cells or pharmacological inhibition in human CD8^+^ T cells impairs tumour cell killing.
- Gli1 can be induced downstream of the TCR by MAPK signalling and Ca^2+^ flux via Ca_v_1 channels.
- Deletion or inhibition of Ca_v_1 channels diminishes TCR-mediated Ca^2+^ flux, Gli1 induction and cytotoxic T cell killing.
- Ca_v_1 agonist or overexpression of a Ca_v_1 gain-of-function mutant enhances Gli1 and killing in cytotoxic lymphocytes.

## Abstract (web summary)

The Hedgehog transcription factor Gli1 is important for T cell killing and is induced downstream of the TCR through Ca_v_1 Ca^2+^ channels. Potentiation of Ca_v_1 function enhances the killing capacity of cytotoxic lymphocytes.

## Graphical abstract

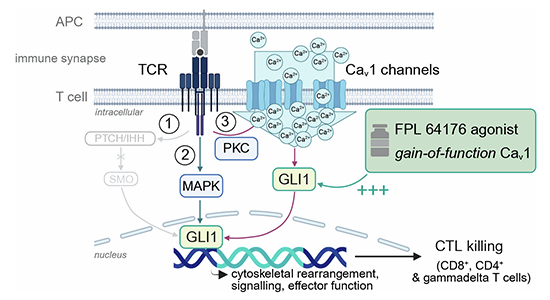

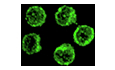

## Introduction

Ca^2+^ signalling orchestrates many aspects of T cell biology through a number of distinct channels including Calcium Release-Activated Calcium (CRAC), transient receptor potential (TRP), P2RX, plasma membrane Ca^2+^ ATPase (PMCA), and L-type (long lasting) voltage-gated Ca^2+^ (Ca_v_) channels (Trebak and Kinet, 2019). Ca_v_1 channels have emerged as putative regulators of T cell function despite T cells being non-excitable cells. Previous work indicates that in T cells Ca_v_1 channels might be regulated by the T cell receptor (TCR) rather than depolarisation of the plasma membrane (Jha et al, 2009; Omilusik et al, 2011). Constitutive loss of Ca_v_1 channels in all tissues leads to defects in thymic T cell development and maturation, with subsequent functional impairment of peripheral T cells (Jha et al, 2009; Omilusik et al, 2011). Furthermore, depletion of Ca_v_1 in Th2 cells led to a reduction in Ca^2+^ flux and effector cytokine production (Cabral et al, 2010). However, expression and function of Ca_v_1 channels in mature cytotoxic lymphocytes is unknown.

Cytotoxic CD8^+^ T lymphocytes (CTLs) eradicate infected and cancerous cells by targeted release of cytotoxic granules at the immune synapse. Immune synapses are structurally and functionally very similar to the primary cilium, a hairlike protrusion from the cell body present on most cells. Both structures dock the centrosome at the plasma membrane via distal appendage proteins and are key signalling hubs and sites of focussed endo- and exocytosis (de la Roche et al, 2016; Griffiths et al, 2010). The similarities between immune synapses and primary cilia prompted us to study Hedgehog (Hh) signalling—a pathway functionally tied to the primary cilium in vertebrates—at the T cell synapse.

Canonical Hh signalling is initiated when one of three Hh ligands—Sonic Hh (Shh), Indian Hh (Ihh) or Desert Hh (Dhh)—bind to the transmembrane receptor Patched (Ptch) at the base of the primary cilium. Upon ligand binding, Ptch releases its inhibition of the key signal transducer Smoothened (Smo) that translocates to the cilium and activates glioma associated oncogene (Gli) transcription factors Gli1, Gli2 and Gli3. These translocate to the nucleus and initiate the transcription of Hh target genes including Gli1 (Briscoe and Therond, 2013). More recently, however, various non-canonical Hh signalling modes have been described which can be divided into two major groups. The first group is independent of Gli transcription: Ptch can act as a dependence receptor and triggers apoptosis in the absence of Hh ligands (Chen et al, 2014), and Smo can regulate the actin cytoskeleton via small GTPases RhoA and Rac1 which occurs through G-proteins and PI3K in fibroblasts and through Src and Fyn in neurons (Polizio et al, 2011; Sasaki et al, 2010). Smo can also trigger calcium (Ca^2+^) release from the endoplasmic reticulum (ER) and in spinal neurons through G_i_ protein and PLCγ-catalysed generation of IP_3_ and the opening of IP_3_-dependent channels (Belgacem and Borodinsky, 2011). Furthermore, Smo has been shown to reprogramme metabolism of adipocytes by inducing extracellular Ca^2+^ flux via Ca_v_1 channels (Teperino et al, 2012). The second group is Gli transcription-dependent and Ptch/Smo-independent. This non-canonical activation of Gli1 has been described in cancers and stem cells. Positive regulators include the MAP Kinase (MAPK) pathway, PI3K-AKT-mTOR, and TGFβ signalling as well as oncogenes such as c-myc (Pietrobono et al, 2019).

Although various roles for Hh signalling during T cell development in the thymus have been proposed (Crompton et al, 2007), little is known about Hh signalling in mature T cells. We have previously shown that Hh signalling component Smo is necessary for CD8^+^ T cell killing. Interestingly, we found that the pathway is initiated independently of extracellular Hh ligands and instead proximal TCR signalling is the main inducer of the Hh pathway in T cells (de la Roche et al, 2013; Hanna et al, 2022). The mechanism by which Hh signalling is initiated downstream of the TCR remains unknown.

Gli1 is the main Gli transcription factor expressed in CD8^+^ T cells and here we show that Gli1 is important for CD8^+^ T cell killing. We demonstrate a key role for MAPK signalling inducing Gli1 in CTLs, as has been previously described in other cell types and tumour cells (Rovida and Stecca, 2015). Additionally, we find binding sites of the MAPK induced transcription factor activator protein 1 (AP-1) in the Gli1 promoter. Most importantly, we identify a previously unknown, non-canonical mode of Hh signalling that culminates in Gli activation via Ca_v_1 channel-mediated Ca^2+^ flux in both CTLs and naive CD8^+^ T cells. Using a selective Ca_v_1 channel agonist or overexpression of a gain-of-function Ca_v_1 subunit we can enhance tumour cell killing capacity in various cytotoxic lymphocytes.

## Results

### Gli1 regulates CTL killing in vitro and in vivo

**Figure EV1:**
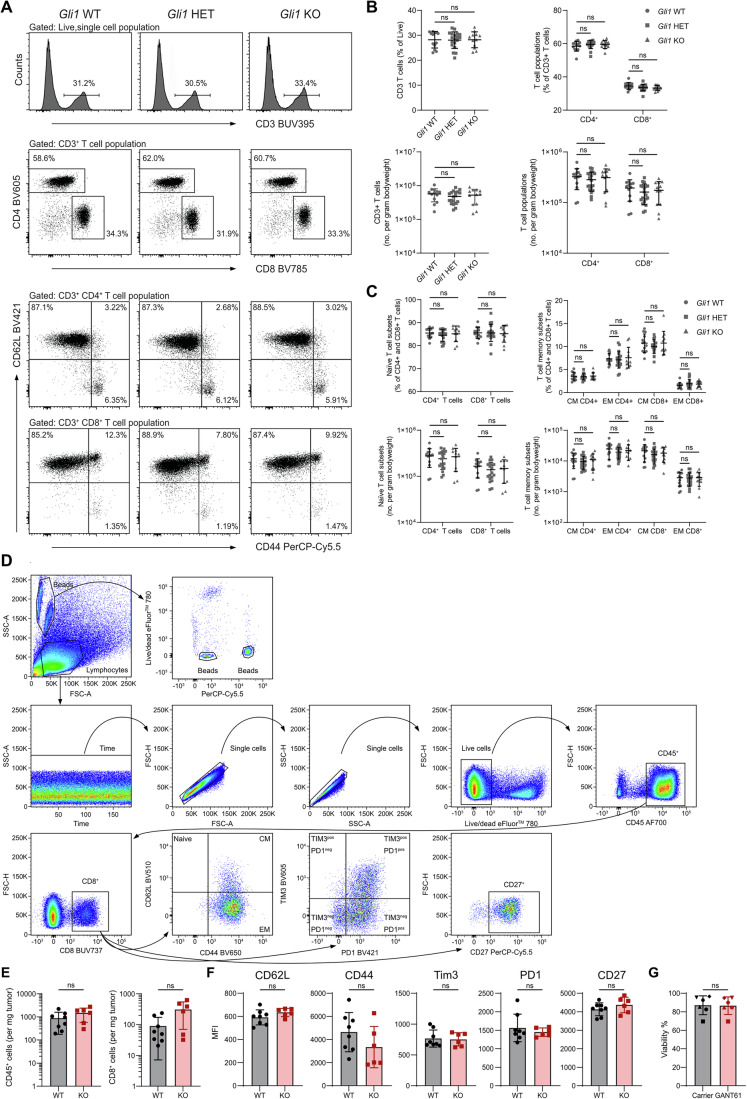
Phenotyping of *Gli1* KO mice, tumour-infiltrating *Gli1* KO T lymphocytes, and viability of GANT61-treated human CTLs. (**A**–**C**) *Gli1* KO mice have phenotypically normal peripheral T cells. Splenocytes were isolated from *Gli1* WT, *Gli1* HET, and *Gli1* KO mice. (**A**) Splenocytes were stained for flow cytometry analysis of T cells (CD3^+^, top panel), CD4^+^ and CD8^+^ T cells (middle panel), and naive (CD62L^+^, CD44^-^), central memory (CM, CD62L^+^, CD44^+^), and effector memory (EM, CD62L^-^, CD44^-^) subsets (bottom two panels). Representative flow cytometry plots for WT, HET, and KO mice are shown. (**B**, **C**) Quantitative analysis of percentages and cell numbers from stainings in (**A**). *n* = 12–19 biological replicates from 9 independent experiments. Every circle (WT), square (HET), and triangle (KO) represents a single mouse. Error bars indicate SD. Statistical significance was assessed using a one-way ANOVA with Dunnett’s multiple comparison test. (**D**–**F**) Tumour-infiltrating *Gli1* WT and *Gli1* KO CD8^+^ lymphocytes are phenotypically similar. Tumour-infiltrating CD8^+^ lymphocytes (CD8^+^ TILs) were isolated from tumours on day 27 (experimental setup shown in Fig. 1D). (**D**) Representative gating strategy. In brief, cells were gated on live, singlet CD45^+^ CD8^+^ cells for further analysis of CD44, CD62L, CD27, TIM3 and PD1 expression. Gating was performed based on FMO controls. (**E**) Absolute numbers of CD45^+^ and CD8^+^ cells within the tumour. (**F**) Mean fluorescence intensities (MFIs) of CD62L, CD44, TIM3, PD1 and CD27 on CD8^+^ cells 27 (*Gli1* WT *n* = 8*, Gli1* KO *n* = 6 biological replicates pooled from 2 independent experiments). Bars represent the mean; error bars indicate SD. Statistical significance was assessed using an unpaired two-tailed Student’s t test (**E**—left panel), a Mann-Whitney two-tailed test (**E**—right panel) or an unpaired two-tailed Student’s t test (**F**). (**G**) Viability of human CTLs was determined by flow cytometry after treatment with 5 mM GANT61 for 8–18 h on day 12–14 post stimulation. *n* = 6 biological replicates from 2 independent experiments. Symbols indicate individual human donors. Bars represent the mean; error bars shown are SD. Statistical significance was assessed using an paired two-tailed Student’s t test. ns = not significant.

**Figure 1:**
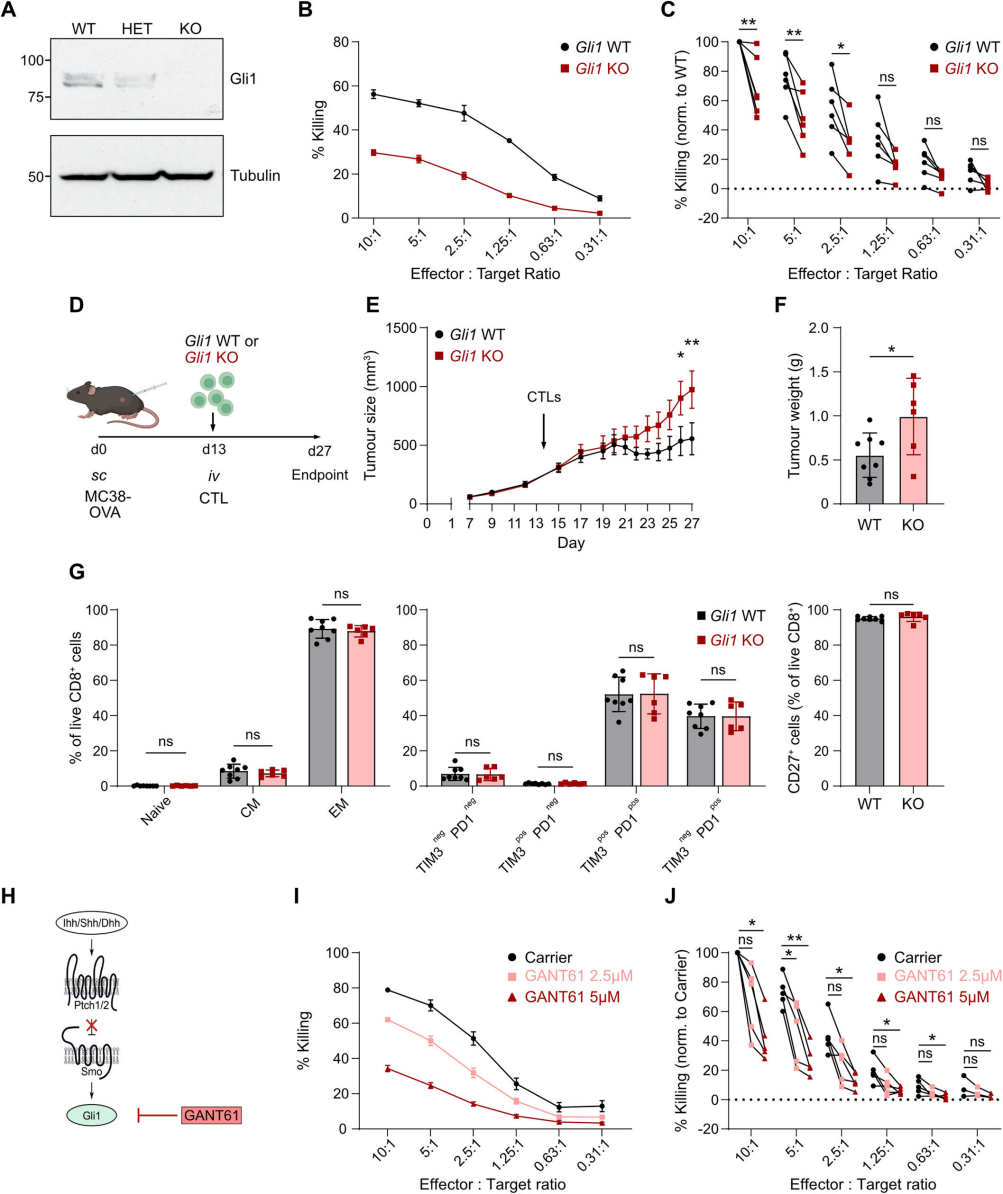
Loss of *Gli1* diminishes cytotoxic T lymphocyte (CTL) killing. (**A**) CTLs were generated from *Gli1* WT, *Gli1* HET and *Gli1* KO mice and restimulated on day 10 with plate-bound anti-CD3ε antibodies for 24 h. Cells were lysed and lysates blotted for protein expression of Gli1 and Tubulin. *n* = 2 biological replicates from 2 independent experiments. Molecular masses are shown in kilodaltons. (**B**, **C**) On day 7 post stimulation CTLs from *Gli1* WT and *Gli1* KO *OT-I* mice were co-cultured with ovalbumin-pulsed EL4 target cells for 4 h at the indicated effector to target ratios and subjected to an LDH cytotoxicity assay. (**B**) Representative killing assay. Data points represent mean of three technical replicates; error bars indicate SD. (**C**) Quantification of *n* = 6 biological replicates from 6 independent experiments normalised to killing of *Gli1* WT cells at a 10:1 Effector:Target ratio. Individual biological replicates are shown. *p*-values were calculated using two-way ANOVA (10:1 *p* = 0.0021, 5:1 *p* = 0.0071, 2.5:1 *p* = 0.0362). (**D**) Experimental setup for in vivo tumour model: *Rag2* KO mice were injected 0.5 × 10^6^ MC38-OvaT4 cells sc. On day 13 post tumour cell injection, mice received 4 × 10^6^ *OT-I* CTLs, either wild type or knockout for *Gli1*, via tail vein injection. (**E**) Tumour size of MC38-OvaT4 tumours as determined by caliper measurements (*Gli1* WT *n* = 8*, Gli1* KO *n* = 6 biological replicates pooled from 2 independent experiments). Arrow indicates the timepoint of *Gli1* WT and *Gli1* KO *OT-I* CTL injection. Data points represent the mean; error bars indicate SEM. *P*-values were calculated using two-way ANOVA with Tukey’s multiple comparisons test, pooled from two independent experiments (day 26 *p* = 0.0311, day 27 *p* = 0.0070, no significant differences were observed at other timepoints). (**F**) Tumour size in grams on day 27 (*Gli1* WT *n* = 8*, Gli1* KO *n* = 6 biological replicates pooled from 2 independent experiments). Bars represent the mean; error bars indicate SD. *p*-values were calculated using an unpaired two-tailed Student’s t test (*p* = 0.0337). (**G**) Flow cytometric analysis of percentages of differentiation and exhaustion/activation markers on tumour-infiltrating CD8^+^ T cells (*Gli1* WT *n* = 8*, Gli1* KO *n* = 6 biological replicates pooled from 2 independent experiments*)*. Bars represent the mean; error bars indicate SD. Statistical significance was assessed using multiple unpaired Welch’s tests with Holm-Sidak multiple comparisons test (left and middle panel) or an unpaired two-tailed Student’s t test (right panel). (**H**) Basic schematic overview of the Hedgehog pathway. GANT61 (red) is a small-molecule Gli inhibitor. (**I**,** J**) On day 12–21 post stimulation CTLs from healthy human donors were co-cultured with anti-CD3ε-pulsed P815 target cells for 3 h in the presence or absence of GANT61 at the indicated effector to target ratios and subjected to an LDH cytotoxicity assay. (**I**) Representative killing assay. Data points represent mean of three technical replicates; error bars indicate SD. (**J**) Quantification of *n* = 5 biological replicates from 3 independent experiments (except for 0.31:1 effector:target ratio, *n* = 3 biological replicates from 2 independent experiments) normalised to killing of carrier-treated cells at a 10:1 effector:target ratio. Individual biological replicates are shown. *p*-values were calculated using two-way ANOVA with Tukey’s multiple comparisons test (10:1 *p* = 0.0027, 5:1 *p* = 0.0284 & *p* = 0.0016, 2.5:1 *p* = 0.0129, 1.25:1 *p* = 0.0375, 0.625:1 *p* = 0.0404). *p < 0.05, **p < 0.01, and ***p < 0.001. ns = not significant. Source data are available online for this figure.

Gli1 is the main Gli transcription factor expressed in mature CD8^+^ T cells (de la Roche et al, 2013). Gli1 is a transcriptional target of the Hh pathway and *Gli1* mRNA levels thus serve as a bona fide readout of Hh signalling activity (Park et al, 2000). Previous work has shown that murine CTLs treated with the small molecule Gli inhibitor GANT61 have diminished killing ability (de la Roche et al, 2013). We wanted to confirm this observation genetically and generated *Gli1*^*eGFP/eGFP*^ (*Gli1* KO) mice by breeding *Gli1*^*eGFP/+*^ mice (Brownell et al, 2011) to homozygosity, disrupting the expression of the *Gli1* gene. *Gli1* KO mice have a phenotypically normal peripheral T cell compartment (Fig. EV1A–C) and CD8^+^ T cells from *Gli1* KO mice lack Gli1 protein (Fig. 1A). CD8^+^ T cells from OTI TCR transgenic mice recognise ovalbumin (Ova) peptide residues 257–264 in the context of MHCI H2K^b^ and we bred *Gli1* KO mice expressing the OTI TCR. We compared *Gli1* WT and KO OTI-transgenic CD8^+^ T cells in their ability to kill Ova-presenting EL-4 murine lymphoma target cells and found that specific T cell killing was reduced by 25–50% in the *Gli1* KO cells compared with the *Gli1* WT controls (Fig. 1B,C).

To assess the role of Gli1 in vivo, we established an Ova-expressing MC38 colorectal carcinoma mouse model. T and B cell-deficient *Rag2* KO mice were implanted with MC38-OvaT4 tumours and given 13 days for the tumour to establish. On day 13, mice were treated with *Gli1* WT or KO  *OT-I*-transgenic CD8^+^ T cells, respectively, and tumour growth was monitored for another 2 weeks (Fig. 1D). *Gli1* KO CD8^+^ T cells were significantly impaired in their ability to control tumour growth (Fig. 1E,F), despite similar numbers of tumour-infiltrating CD8^+^ lymphocytes (Fig. EV1D,E) with similar activation and exhaustion characteristics as *Gli1* WT CD8^+^ T cells (Figs. 1G and EV1F). Thus, Gli1 contributes significantly to effective CTL killing during the anti-tumour response in vivo.

To determine whether Gli1 is also important for the killing ability of human CD8^+^ T cells, we treated human CTLs with the small molecule Gli antagonist GANT61 (Fig. 1H) and assessed their ability to kill P815 mastocytoma target cells in a redirected lysis assay. GANT61 treatment did not affect viability of human CD8^+^ T cells (Fig. EV1G) but significantly inhibited human CTL killing in a dose-dependent manner (Fig. 1I,J), demonstrating that Gli1 is important for both murine and human CTL killing.

### MAP kinase signalling downstream of the TCR promotes Gli1 induction in CTLs

Having demonstrated that Gli1 is required for murine and human CTL killing, we next sought to elucidate how Gli1 is mechanistically activated. CD8^+^ T cell killing is initiated by engagement of the TCR and the TCR-associated proximal tyrosine kinase Lck, which has been shown to be required for the induction of Gli1 (de la Roche et al, 2013). Induction of *Gli1* mRNA is a robust readout of Gli1 activation and thus active Hh signalling (Park et al, 2000). We therefore wanted to investigate how signalling downstream of the TCR leads to Gli1 activation.

**Figure 2:**
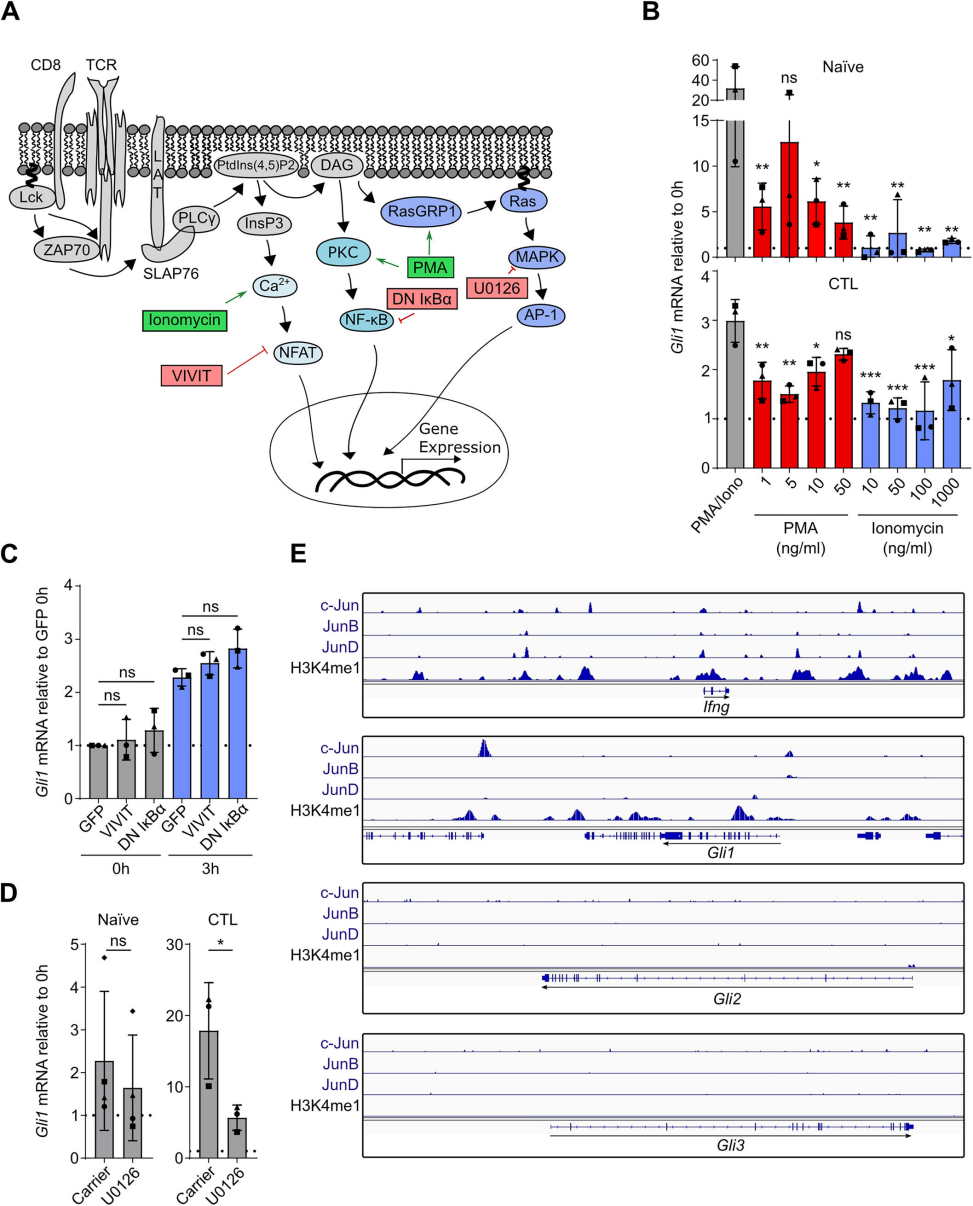
MAPK signalling drives Gli1 induction in CTLs post TCR stimulation. (**A**) Schematic overview of signalling downstream of the TCR culminating in the activation of transcription factors NFAT, NF-κB and AP-1. Small molecule agonists (green) as well as antagonists and dominant negative constructs (red) of the branches of TCR signalling are shown. (**B**–**D**) CD8^+^ T cells were isolated from spleens and inguinal lymph nodes of *Rag2* KO *OT-I* mice. (**B**) Naive CD8^+^ T cells or CTLs were treated with indicated doses of PMA and/or Ionomycin for 3 h (naive, top panel) (CTL, bottom panel) before being subjected to qRT-PCR analysis. For double treatment with PMA and Ionomycin, 50 ng/ml PMA and 1 μg/ml Ionomycin was used. *n* = 3 biological replicates from 3 independent experiments. Data is normalised to *CD3ε* as a reference gene. Similar results were obtained when *Tbp* was used as a reference gene. Bars represent the mean; error bars indicate SD. *p* values were calculated using an ordinary one-way ANOVA with Dunnett’s multiple comparison test comparing each condition to PMA/Iono (Naive—PMA 1 *p* = 0.0092, PMA 5 *p* = 0.0706, PMA 10 *p* = 0.0108, PMA 50 *p* = 0.0055, Iono 10 *p* = 0.0024, Iono 50 *p* = 0.0039, Iono 100 *p* = 0.0022, Iono 1000 *p* = 0.0030, CTL—PMA 1 *p* = 0.0095, PMA 5 *p* = 0.0017 PMA 10 *p* = 0.0299, PMA 50 *p* = 0.2308, Iono 10 *p* = 0.0006, Iono 50 *p* = 0.0003, Iono 100 *p* = 0.0002, Iono 1000 *p* = 0.0102). (**C**) CTLs were nucleofected with GFP, GFP-VIVIT or Dominant Negative IκBα (DN IκBα) on day 6. Cells were restimulated with plate-bound anti-CD3ε antibodies on day 7 for 3 h for qRT-PCR analysis. *n* = 3 biological replicates from 3 independent experiments. Data is normalised to *CD3ε* as a reference gene. Similar results were obtained when *Tbp* was used as a reference gene. Bars represent the mean; error bars indicate SD. Statistical significance was assessed using a two-way ANOVA with Dunnett’s multiple comparison test. (**D**) Naive CD8^+^ T cells were stimulated with plate-bound anti-CD3/CD28 antibodies (left) and CTLs were restimulated with plate-bound anti-CD3ε (right) for 15 h in the presence of 10 μM U0126 or carrier control before RNA was extracted for qRT-PCR analysis. *n* = 4 (left panel) or *n* = 3 (right panel) biological replicates from 2 and 3 independent experiments, respectively. Data is normalised to *Tbp* as a reference gene. Similar results were obtained when *CD3ε* was used as a reference gene. Bars represent the mean; error bars indicate SD. *p* values were calculated using an unpaired two-tailed Student’s t test (Naive *p* = 0.1335, CTL *p* = 0.0389). Symbols indicate biological replicates. *p < 0.05, **p < 0.01, and ***p < 0.001. ns = not significant. (**E**) Analysis of ChIP-Seq data from (*Kurachi* et al) showing binding of AP-1 family members (c-Jun, JunB, JunD) at the Gli1 promoter. Binding at the *Ifng* promoter serves as a positive control and no binding at the *Gli2* and *Gli3* promoters serve as negative controls. Source data are available online for this figure.

TCR engagement by cognate antigen presented on MHCI molecules leads to recruitment of Lck to the TCR complex (Fig. 2A). Lck phosphorylates ZAP70 (ζ-chain-associated protein kinase of 70 kDa) which in turn phosphorylates the adaptor protein LAT (Linker for activation of T cells) leading to the formation of the LAT signalosome. The LAT signalosome propagates signal branching into three major signalling pathways ultimately culminating in the activation of the transcription factors Nuclear factor of activated T cells (NFAT), Nuclear factor kappa-light-chain-enhancer of activated B cells (NFκB), and Activator protein 1 (AP-1) (Brownlie and Zamoyska, 2013).

The three signalling branches of the TCR: NFAT, NF-κB, and AP-1, respectively, can be pharmacologically manipulated. Ionomycin is a Ca²⁺ ionophore that increases intracellular Ca²⁺ concentrations by allowing extracellular Ca²⁺ entry into the cytosol, which in turn induces further Ca²⁺ release from ER stores, leading to activation of NFAT. Phorbol 12-myristate 13-acetate (PMA) activates protein kinase C (PKC) isoforms and thus NFκB and AP-1 transcription factors. When naive CD8^+^ T cells or CTLs were treated with both PMA and Ionomycin, to achieve maximum activation of the T cells, *Gli1* RNA was induced 30 and 3-fold, respectively (Fig. 2B). On their own, however, only PMA induced significant upregulation of Gli1 in naive T cells and CTLs, while ionomycin had only a small effect on Gli1 levels at the highest concentration. This suggested that NFAT signalling might be less important in Gli1 induction.

Since mimicking TCR engagement by PMA and Ionomycin is not physiological, we decided to specifically block the three branches of TCR signalling downstream of TCR cross-linking.

**Figure EV2:**
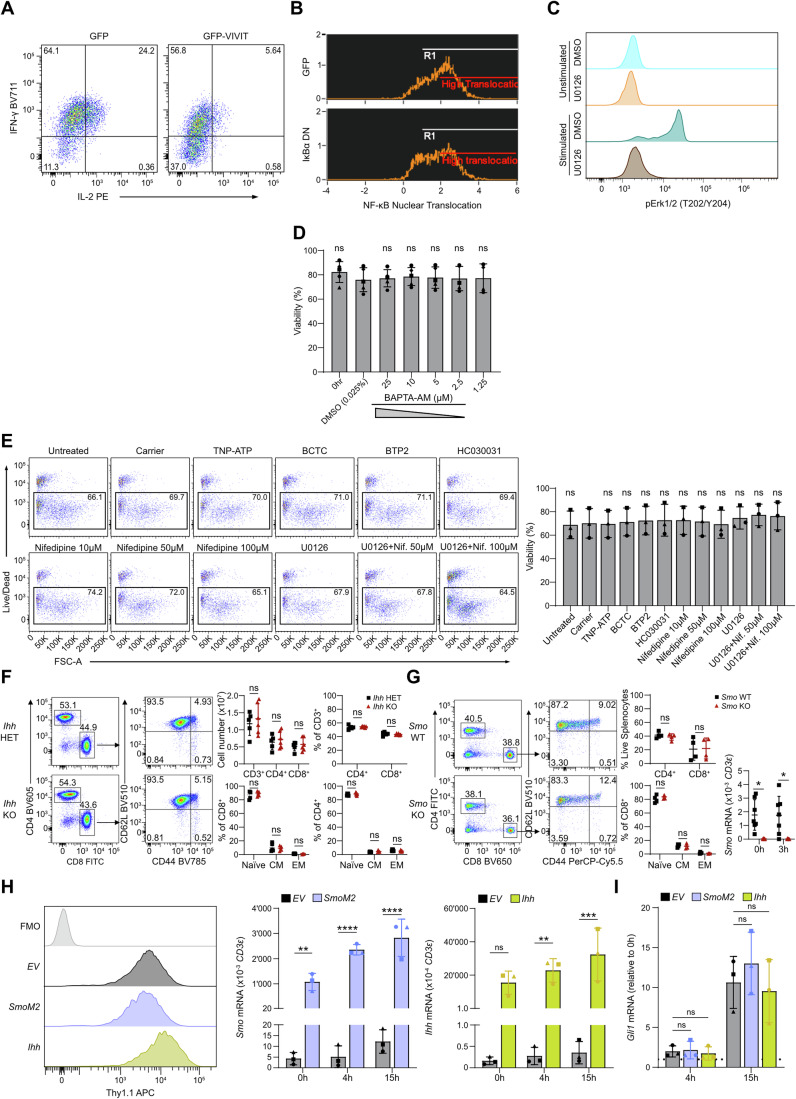
Validation of TCR signalling inhibitors, cell viability of mouse CD8 + T cells upon Ca^2+^ blockade and characterisation of *dLckCre*^*+*^*Ihh* KO, *GzmBER*^*T2*^*Cre*^*+*^*Smo* KO mice and retroviral overexpression. (**A**–**C**) Validation of TCR signalling inhibitors. (**A**) CTLs were nucleofected with GFP or GFP-VIVIT on day 6. Cells were restimulated with PMA/Ionomycin on day 7 prior to intracellular flow cytometric staining for IL-2 and IFN-γ. FACS plots are gated on GFP^+^ cells. (**B**) CTLs were nucleofected with GFP or Dominant Negative I*κ*Bα (DN I*κ*Bα) on day 6. Cells were restimulated with anti-CD3 on day 7 for 1 h prior to ImageStream analysis. (**C**) Naive CD8^+^ T cells were isolated from spleens and peripheral lymph nodes of *Rag2* KO *OT-I* mice and stimulated with cross-linked soluble anti-CD3/CD28 in the presence of 10 µM U0126 or carrier control prior to analysis by flow cytometry of phosphorylated Erk1/2 (pErk1/2). (**A**–**C**) One representative experiment of *n* = 2–3 biological replicates from 2–3 independent experiments is shown. (**D**,** E**) Cell viability of mouse CD8^+^ T cells upon Ca^2+^ blockade. (**D**) Murine CTLs were restimulated with plate-bound anti-CD3ε antibodies for 3 h in the presence of indicated doses of the cell permeable Ca^2+^ chelator BAPTA-AM or carrier control before viability was assessed via the Beckman Coulter Vi-Cell XR. *n* = 4–5 biological replicates from 4–5 independent experiments. Symbols indicate biological replicates. Bars represent the mean; error bars shown are SD. Statistical significance was assessed using a one-way ANOVA with Dunnett’s multiple comparison test comparing each condition to DMSO (0.025%). (**E**) Murine CTLs were restimulated with plate-bound anti-CD3e in the presence of the indicated Ca^2+^ channel inhibitors or carrier control for 15 h before flow cytometric analysis for cell viability. *n* = 3 biological replicates from 3 independent experiments. Symbols indicate biological replicates. Bars represent the mean; error bars indicate SD. Statistical significance was assessed using a one-way ANOVA with Dunnett’s multiple comparison test comparing each condition to carrier. (**F**–**I**) Characterization of *dLckCre*^*+*^ *Ihh* KO, *GzmBER*^*T2*^*Cre*^*+*^ *Smo* KO mice and retroviral transduction constructs. (**F**) Splenocytes were isolated from *dLckCre*^*+*^*Ihh*^*+/fl*^ (*Ihh* HET) and *dLckCre*^*+*^*Ihh*^*fl/fl*^ (*Ihh* KO) mice and subjected to flow cytometric phenotypic analysis. Representative FACS plots are shown (left 2 panels). Quantification of relative percentages, cell numbers and steady-state phenotype: naive (CD62L^+^, CD44^-^), central memory (CM, CD62L^+^, CD44^+^), and effector memory (EM, CD62L^-^, CD44^-^) shown on the right two panels. Every square represents one HET, and every triangle one individual KO mouse. *n* = 5–6 biological replicates from 5–6 independent experiments. Error bars indicate SD. Statistical significance was assessed using multiple unpaired Welch’s tests with Holm-Sidak multiple comparisons test. (**G**) Splenocytes were isolated from *GzmBER*^*T2*^*Cre*^*+*^*Smo*^*+/+*^ (*Smo* WT) and *GzmBER*^*T2*^*Cre*^*+*^ *Smo*^*fl/fl*^ (*Smo* KO) mice and subjected to flow cytometric phenotypic analysis. Representative FACS plots are shown (left). Quantification of relative percentages and steady-state memory phenotype shown (middle). Every square represents one WT, and every triangle one individual KO mouse. CTLs were generated from these mice and restimulated at day 8/9 for qRT-PCR analysis of Smo (right). *n* = 4–7 biological replicates from 4 independent experiments. Error bars indicate SD. *p* values were calculated using multiple unpaired Welch’s tests with Holm-Sidak multiple comparisons (0 h *p* = 0.0296, 3 h *p* = 0.0222). (H) CD8^+^ T cells were retrovirally transduced with constructs encoding empty vector (EV), SmoM2 or Ihh, respectively. Representative flow cytometry plots of sorted, transduced (Thy1.1^+^) cell populations shown (left panel). Sorted, transduced CTLs were restimulated at day 8/9 for qRT-PCR analysis of Smo (middle panel) or Ihh (right panel), respectively. *n* = 3 biological replicates from 3 independent experiments. *p* values were calculated using a two-way ANOVA with Sidak’s multiple comparison test (Smo 0 h *p* = 0.0073, Smo 4 h *p* < 0.0001, Smo 15 h *p* < 0.0001, Ihh 0 h *p* = 0.084, Ihh 4 h *p* = 0.0098, Ihh 15 h *p* = 0.0007). (**I**) CD8^+^ T cells were retrovirally transduced with pMig constructs encoding empty vector (EV), SmoM2 or Ihh, respectively. Sorted, transduced CTLs were restimulated on day 8/9 for qRT-PCR analysis. *n* = 3 biological replicates from 3 independent experiments. Statistical significance was assessed using a two-way ANOVA with Tukey’s multiple comparison test. (**H**, **I**) Data is normalised to *CD3ε* as a reference gene. Similar results were obtained when *Tbp* was used as a reference gene. Symbols indicate biological replicates. Bars represent the mean; error bars indicate SD. *p < 0.05, **p < 0.01, ****p < 0.0001. ns = not significant.

The NFAT and NFκB signalling branches can be specifically blocked by transfecting primary CD8^+^ T cells with VIVIT or Dominant Negative (DN) IkBα, respectively. VIVIT is a 16mer peptide that selectively disrupts the interface between Calcineurin and NFAT (Aramburu et al, 1999). As expected, VIVIT-expressing CTLs showed reduced IL-2 and IFNγ production (Fig. EV2A). DN IkBα cannot be phosphorylated by IKKβ and thus NFκB is sequestered in the cytosol (Brockman et al, 1995). Indeed, CTLs transfected with DN IkBα had reduced nuclear NFκB translocation (Fig. EV2B). When Gli1 induction was assessed at steady state and after 3 h of restimulation, VIVIT- and DN IkBα-expressing CTLs had no defect in Gli1 induction compared to GFP-transfected controls (Fig. 2C).

The third branch of TCR signalling leading to AP-1 activation can be blocked by U0126, a selective MAP Kinase (MEK1 and MEK2) inhibitor (Fig. EV2C) (Adachi and Davis, 2011). U0126 treatment did not affect cell viability (Fig. EV2E) but led to a significant reduction of Gli1 induction after TCR stimulation in CTLs (Fig. 2D). In naive CD8^+^ T cells U0126 treatment led to a modest, non-significant reduction of *Gli1* (Fig. 2D). MAPK signalling has been shown to be able to activate Gli1 through one of two mechanisms. The first is through direct activation of Gli1 transcription by AP-1 heterodimer which is formed by one Fos and one Jun (c-Jun, JunB or JunD) family member. The second mechanism is via the kinase activity of MAPK which is purported to activate an unknown upstream regulator of Gli1 (Rovida and Stecca, 2015).

To determine which mode of MAPK signalling is responsible for the Gli1 induction, we analysed existing ChIP-Seq datasets generated by Kurachi et al to assess direct AP-1 binding to the genomic locus of *Gli1* in CTLs (Kurachi et al, 2014). Since AP-1 has been demonstrated to bind upstream of the *Ifng* locus (Roychoudhuri et al, 2016; Samten et al, 2008), we analysed whether Jun transcription factors bind to this locus in the dataset, and indeed, all three Jun family members bind upstream of the *Ifng* locus (Fig. 2E, top panel). Interestingly, we observed that both c-Jun and JunB also bind upstream of the *Gli1* locus (Fig. 2E). By contrast, no binding was observed upstream of the *Gli2* and the *Gli3* loci, which are both not reported to be expressed in murine CTLs (de la Roche et al, 2013) (Fig. 2E).

Taken together, this data indicates that of the main downstream signalling arms of the TCR (NFAT, NF-*κ*B, AP-1), AP-1 signalling selectively controls Gli1 induction in CTLs, in part by direct binding of AP-1 to the genomic locus of *Gli1*. However, none of the major downstream signalling arms appear to be individually responsible for Gli1 induction in naive CD8^+^ T cells.

### Ca^2+^ flux contributes to Gli1 induction

**Figure 3:**
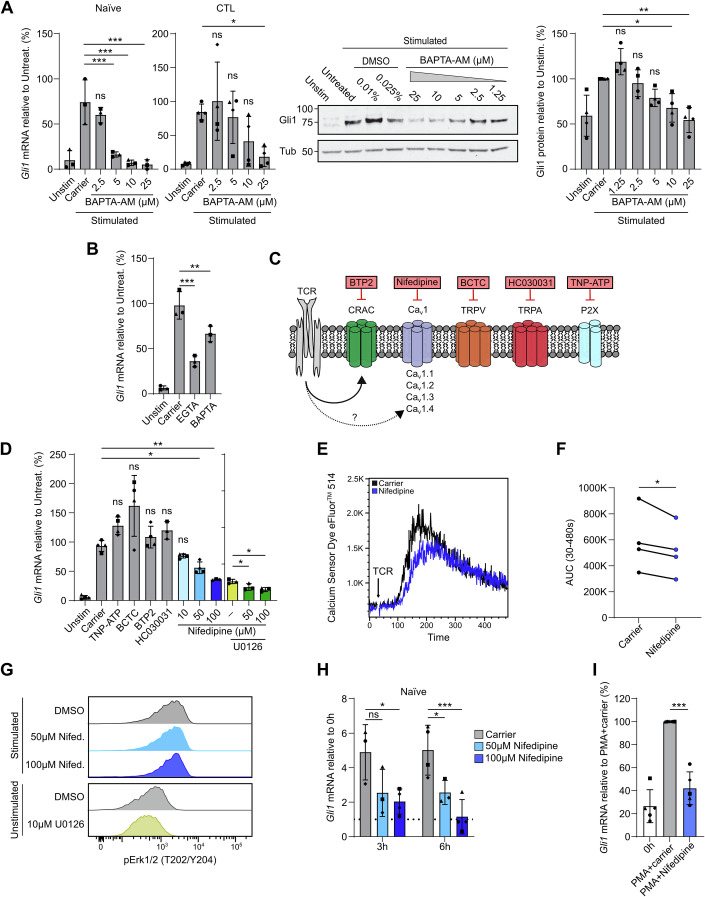
Nifedipine-sensitive Ca_v_ channels control Gli1 induction in naive CD8^+^ T cells and CTLs post TCR stimulation. (**A**) Naive CD8^+^ T cells (left panel) or CTLs (middle panel) from *RAG1* KO *OT-I* mice were stimulated with plate-bound anti-CD3/CD28 or anti-CD3ε antibodies alone for 3 h in the presence of indicated doses of the cell permeable Ca^2+^ chelator BAPTA-AM or carrier control. RNA was extracted for qRT-PCR analysis. Data is normalised using *CD3ε* as a reference gene. Similar results were obtained when *Tbp* was used as a reference gene. Immunoblot analysis and quantification of Gli1 and tubulin of CTLs left unstimulated or restimulated with plate-bound anti-CD3ε for 3 h in the presence of BAPTA-AM or carrier control (right panel). Molecular masses are shown in kilodaltons. *n* = 3 (left panel) or *n* = 4 (right panel) biological replicates from 3 and 4 independent experiments, respectively. Bars represent the mean; error bars indicate SD. *p* values were calculated using a one-way ANOVA with Dunnett’s multiple comparison test (naive 5 µM *p* = 0.003, naive 10 µM *p* < 0.0001, naive 25 µM p < 0.0001, A CTL 25 µM *p* = 0.0437) or a one-way ANOVA with Holm-Sidak multiple comparison test (10 µM *p* = 0.0147, 25 µM *p* = 0.0010). (**B**) CTLs were restimulated for 3 h with plate-bound anti-CD3ε in the presence of 1.25 mM BAPTA, 1.25 mM EGTA or carrier control before RNA was extracted for qRT-PCR analysis (right panel). *n* = 3 biological replicates from 3 independent experiments. Data is normalised using *CD3ε* as a reference gene. Similar results were obtained when *Tbp* was used as a reference gene. Bars represent the mean; error bars indicate SD. *p* values were calculated using a one-way ANOVA with Holm-Sidak multiple comparison test (EGTA *p* = 0.0003, BAPTA *p* = 0.0053). (**C**) Schematic overview of Ca^2+^ channels with respective antagonists (red) used in this study. (**D**) CTLs were restimulated with plate-bound anti-CD3ε in the presence of the indicated inhibitors or carrier control for 15 h before RNA was extracted for qRT-PCR analysis. *n* = 3–4 biological replicates from 3–4 independent experiments. Data is normalised using *CD3ε* as a reference gene. Similar results were obtained when *Tbp* was used as a reference gene. Bars represent the mean; error bars indicate SD. *p* values were calculated using a one-way ANOVA with Holm-Sidak multiple comparison test (carrier vs Nif50µM *p* = 0.0202, carrier vs Nif100µM *p* = 0.0002, U0126 vs U0126+Nif50µM *p* = 0.0311, U0126 vs U0126+Nif50µM *p* = 0.0118). (**E**,** F**) On day 6–8 post stimulation murine CTLs were loaded with Calcium Sensor Dye eFluor^TM^ 514 and Ca^2+^ flux was analysed by flow cytometry in the presence or absence of 100 μM nifedipine. After 30 s, the TCR was activated. (**E**) Representative flow cytometry plot is shown. Arrow indicates time of TCR-cross-linking. (**F**) Quantification of Ca^2+^ flux after TCR cross-linking (between 30s-480s). *n* = 4 biological replicates from 4 independent experiments. Data points represent individual biological replicates. *p* values were calculated using a paired two-tailed Student’s t test (*p* = 0.0456). AUC = area under the curve. (**G**) CTLs were restimulated with 10 µg/ml cross-linked soluble anti-CD3ε in the presence of nifedipine, 10 µM U0126 or carrier control. Cells were subsequently prepared for intracellular flow cytometric analysis. *n* = 2 biological replicates from 2 independent experiments. (**H**) Naive CD8^+^ T cells were stimulated with plate-bound anti-CD3ε in the presence of the indicated doses of nifedipine or carrier control. Data is normalised using *CD3ε* as a reference gene. Similar results were obtained when *Tbp* was used as a reference gene. Bars represent the mean; error bars indicate SD. *p* values were calculated using a two-way ANOVA with Sidak’s multiple comparison test (3 h carrier vs Nif50µM *p* = 0.0185, 6 h carrier vs Nif50µM *p* = 0.0455, 6 h carrier vs Nif50µM *p* = 0.001). *n* = 3 biological replicates from 3 independent experiments. (**I**) Naive CD8^+^ T cells were stimulated with 50 ng/mL PMA in the presence of carrier control or 100 μM nifedipine for 3 h before RNA was extracted for qRT-PCR analysis. *Tbp* served as a reference gene. *n* = 5 biological replicates from 2 independent experiments. Bars represent the mean; error bars indicate SD. *p* values were calculated using a one-sample Student’s t-test (*p* = 0.0008). Symbols indicate biological replicates. *p < 0.05, **p < 0.01, ***p < 0.001. ns = not significant. Source data are available online for this figure.

We could not clearly associate the induction of Gli1 with one specific branch of TCR signalling in naive CD8^+^ T cells. While MAPK signalling accounted to some extent for the induction of Gli1 in CTLs, it was still not clear what other pathways drive the induction of Gli1 in CD8^+^ T cells. Ca^2+^ is required for the initiation of canonical Hh signalling at the level of the Ihh:Ptch interaction (McLellan et al, 2008) and is an important second messenger in T cells (Trebak and Kinet, 2019). We therefore investigated whether Ca^2+^ signalling was important for Gli1 induction. We subjected naive T cells and CTLs to treatment with a cell-permeable high-affinity Ca^2+^ chelator, BAPTA-AM, prior to TCR stimulation. Ca^2+^ chelation with BAPTA-AM led to complete abrogation of Gli1 induction in naive T cells and a significant dose-dependent reduction of *Gli1* mRNA and protein induction in CTLs upon TCR stimulation (Fig. 3A) without affecting cell viability at all concentrations used (Fig. EV2D). BAPTA-AM can chelate Ca^2+^ in both intra- and extracellular compartments. To determine the relative contribution of each of these pools of Ca^2+^ to Gli1 induction, we repeated the experiments using non-cell-permeable BAPTA or EGTA which both chelate extracellular Ca^2+^. Extracellular Ca^2+^ chelation significantly reduced Gli1 induction in CTLs (Fig. 3B). Thus, Ca^2+^ flux is required for Gli1 induction and relies on both extra- and intracellular Ca^2+^ pools.

### L-type voltage-gated Ca^2+^ channels mediate Gli1 induction and act independently of MAPK signalling

We next sought to determine whether Ca^2+^ signalling through a specific Ca^2+^ channel is responsible for regulating Gli1 induction. We targeted channels previously shown to be expressed in T cells such as CRAC channels, Ca_v_1, TRPV, TRPA and P2X channels (Trebak and Kinet, 2019) with small-molecule inhibitors (Fig. 3C) at doses previously reported in literature (Bertin et al, 2014; Kotturi et al, 2003; Sahoo et al, 2019; Weidinger et al, 2013; Woehrle et al, 2010). Treatment of CTLs at these at doses had no effect on cell viability during the course of the experiment (Fig. EV2E). Interestingly, inhibition of CRAC channels showed no effect on Gli1 induction and neither did inhibition of TRPV, TRPA and P2X channels.

Only inhibition of Ca_v_1 channels with nifedipine showed a dose-dependent reduction of Gli1 induction in CTLs (Fig. 3D). To confirm that this mechanism was due to reduced Ca^2+^ flux, we measured TCR-induced Ca^2+^ flux in the presence or absence of nifedipine and show that nifedipine significantly inhibited TCR-induced Ca^2+^ flux (Fig. 3E,F). Ca_v_1 channels have been shown to play a critical role in CD8^+^ T cell development (Badou et al, 2013). Evidence in the literature has shown that constitutive knockout of *CACNA1F* (encoding Ca_v_1.4) during T cell development results in impaired MAPK signalling strength (Omilusik et al, 2011). We hence sought to determine whether the effect of nifedipine on Gli1 induction in CTLs was through inhibition of MAPK signalling or through an independent mechanism. Inhibition of both, MAPK and Ca_v_1 channels, showed an additive decrease in Gli1 induction (Fig. 3D) but importantly phosphorylated Erk (pErk) levels were unaffected by treatment with nifedipine (Fig. 3G), indicating that Ca_v_1 channels were regulating Gli1 induction through a mechanism distinct from MAPK signalling. Given that MAPK signalling is not a major driver of Gli1 induction in naive CD8^+^ T cells (Fig. 2D) we sought to investigate whether Ca_v_1 channels might be the main source of Gli1 induction. Nifedipine treatment of naive CD8^+^ T cells resulted in complete abrogation of Gli1 induction post TCR stimulation (Fig. 3H). Published work had suggested that Ca_v_1 channel activity might be modulated by PKC (Robert et al, 2013; Strauss et al, 1997). To investigate this, we stimulated naive CD8^+^ T cells with PMA, a potent activator of PKC, in the presence or absence of nifedipine. While PMA alone led to a robust upregulation of *Gli1* expression, presence of nifedipine abrogated *Gli1* induction (Fig. 3I), indicating that Ca_v_1 channels are indeed activated downstream of PKC.

### L-type voltage-gated Ca^2+^ channels regulate T cell killing in a Gli1-dependent manner

**Figure 4:**
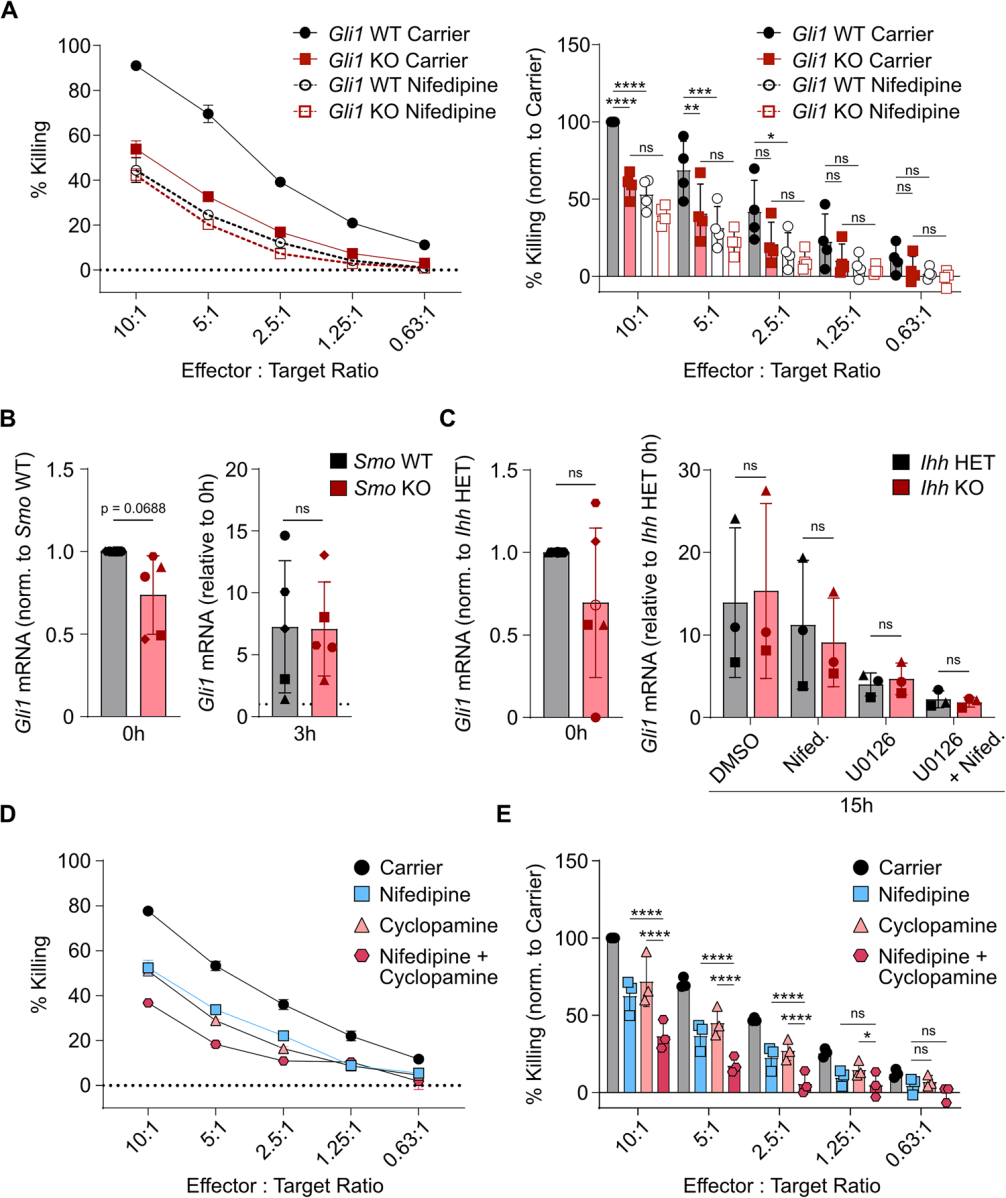
Ca_v_1 family channels control CTL killing in a Gli1-dependent manner and function independently of canonical Hh signalling. (**A**) CTLs were generated from *Gli1* WT and *Gli1* KO mice. On day 7, cells were co-cultured with either Ova-pulsed EL4 cells or anti-CD3ε antibody-coated P815 target cells at the indicated effector to target ratios and subjected to an LDH cytotoxicity assay in the presence of 100 μM nifedipine or carrier control. Representative killing assay (left, data points represent mean of three technical replicates; error bars indicate SD) and quantification of *n* = 4 biological replicates from 4 independent experiments normalised to killing of *Gli1* WT carrier condition at a 10:1 effector:target ratio (right, bars represent the mean; error bars indicate SD) are shown. *p* values were calculated using a two-way ANOVA with Tukey’s multiple comparison test (10:1 *p* < 0.0001 & *p* < 0.0001, 5:1 *p* = 0.0051 & *p* = 0.0001, 2.5:1 *p* = 0.0118). (**B**) CTLs were generated from *GzmBER*^*T2*^*Cre*^*+*^ *Smo*^*+/+*^ (*Smo* WT) or *GzmBER*^*T2*^*Cre*^*+*^ *Smo*^*fl/fl*^ (*Smo* KO) mice. Cells were treated with 4-OH-Tamoxifen for the duration of the in vitro experiments. CTLs were restimulated for 3 h on day 8/9 for qRT-PCR analysis. *n* = 5 biological replicates from 5 independent experiments per condition. Bars represent the mean; error bars indicate SD. Statistical significance was assessed using a one sample Student’s t test (left panel) or a paired Student’s t test (right panel). Fold change of *Gli1* expression relative to 0 h (set to “1”) is shown for *Smo* WT (left bar) and *Smo* KO (right bar) using their respective 0 h timepoint. Symbols indicate biological replicates. (**C**) CD8^+^ T cells were isolated from *dLckCre*^*+*^ *Ihh*^*fl/+*^ (*Ihh* Het) or *dLckCre*^*+*^ *Ihh*^*fl/fl*^ (*Ihh* KO) mice. CTLs were restimulated for 15 h on day 8/9 in the presence of 100 µM nifedipine and/or 10 µM U0126 or carrier control for qRT-PCR analysis. *n* = 5 (naive) and *n* = 3 (restimulation) biological replicates from 5 and 3 independent experiments, respectively. (**B**, **C**) Data is normalised to *CD3ε* as a reference gene. Similar results were obtained when *Tbp* was used as a reference gene. Bars represent the mean; error bars indicate SD. Statistical significance was assessed using a one sample Student’s t test (left panel) or multiple unpaired Welch’s tests with Holm-Sidak multiple comparisons test (right panel). (**D**,** E**) On day 6–8 of culture, *Rag2* KO *OT-I* CTLs were pre-treated with either carrier or 2.5 μM cyclopamine for 18 h before being co-cultured with ovalbumin-pulsed EL4 target cells for 4 h in the presence or absence of 50 μM nifedipine at the indicated effector to target ratios. (**D**) representative LDH cytotoxicity assay (**E**) *n* = 3 biological replicates from 3 independent experiments. *p* values were calculated using a two-way ANOVA with Tukey’s multiple comparisons test (**E**, all *p* < 0.0001 except for 1.25:1 Cyclopamine vs Nifedipine+Cyclopamine *p* = 0.0193). Bars represent the mean; error bars indicate SD. * *p* < 0.05, *** *p* < 0.001 **** *p* < 0.0001. ns = not significant. Source data are available online for this figure.

**Figure EV3:**
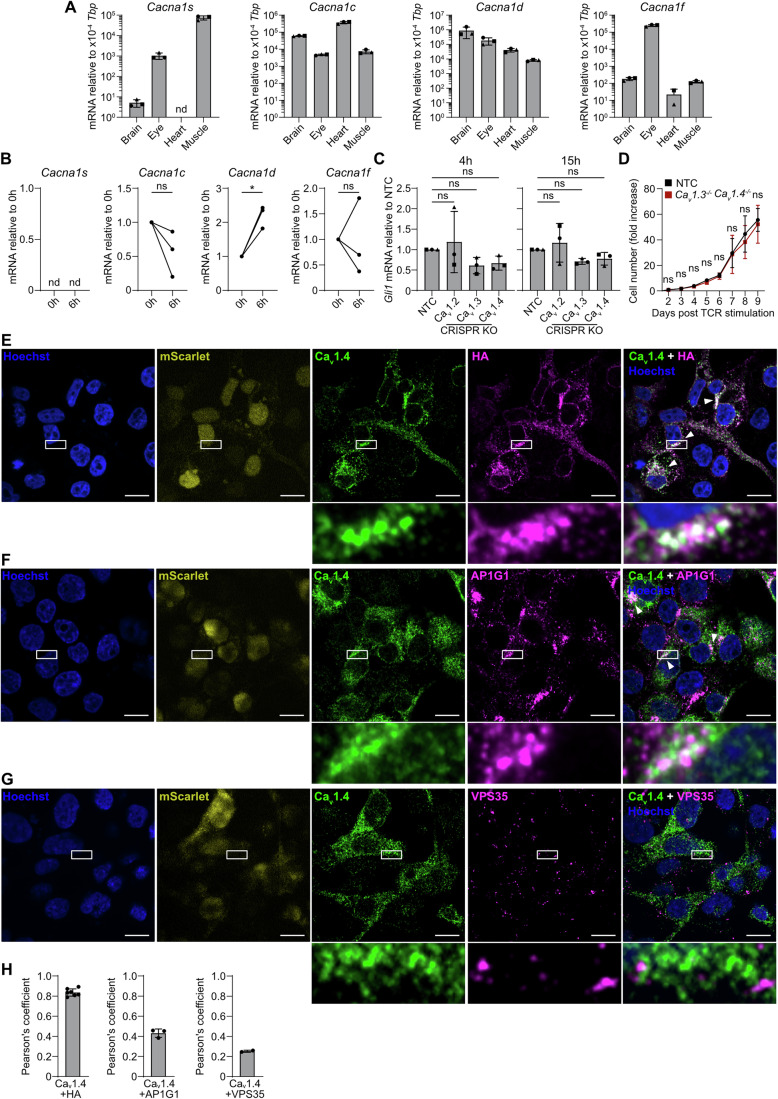
Expression of Ca_v_1 channels in murine CTLs, characterisation of CRISPR knockouts and validation of Ca_v_1.4 antibody specificity and intracellular colocalisation in HEK293T cells. (**A**–**D**) Expression of Ca_v_1 channels and characterization of CRISPR knockouts. (**A**) Expression analysis of *Cacna1s,c,d,f* in murine tissues relative to *Tbp* as a housekeeping gene. *n* = 3 biological replicates. Similar results were obtained when *b2m* was used as a reference gene. Bars represent the mean; error bars indicate SD. (**B**) mRNA expression of *Cacna1s,c,d,f* in murine day 8 CTLs at steady state and after 6 h of TCR stimulation. Data is normalised to *Tbp* as a reference gene. Similar results were obtained when *b2m* was used as a reference gene. *n* = 3 biological replicates. *p* values were calculated using a two-tailed one sample t test (*p* = 0.0245). (**C**) CTLs were electroporated with RNP complexes at day 2 post stimulation to generate *Ca*_*v*_*1.1*^*-/-*^*, Ca*_*v*_*1.2*^*-/-*^*, Ca*_*v*_*1.3*^*-/-*^*, Ca*_*v*_*1.4*^*-/-*^ (KO) or non-targeting control (NTC) CTLs. CTLs were restimulated on day 8/9 with plate-bound anti-CD3ε for qRT-PCR analysis. *n* = 3 biological replicates from 3 independent experiments. Data is normalised to *CD3ε* as a reference gene. Similar results were obtained when *Tbp* was used as a reference gene. Bars represent the mean; error bars indicate SD. Statistical significance was assessed using a one-way ANOVA with Dunnett’s multiple comparison test. (**D**) *Ca*_*v*_*1.3*^*-/-*^ and *Ca*_*v*_*1*.*4*^*-/-*^ CRISPR double KO CTLs and NTC CTLs were generated as described previously. Fold expansion in cell numbers after CRISPR performed at day 2 post-stimulation is shown. *n* = 3 biological replicates from 3 independent experiments. Symbols indicate biological replicates. Error bars indicate SD. Statistical significance was assessed using a two-way ANOVA with Sidak’s multiple comparison test. (**E**–**H**) Validation of Ca_v_1.4 antibody specificity and intracellular colocalisation with vesicular markers in HEK293T cells. HEK 293T cells were plated on coverslips prior to lipofection with a construct expressing an HA-tagged human Ca_v_1.4 and mScarlet reporter. 18 h post lipofection, cells were fixed and stained with antibodies against (**E**) Ca_v_1.4 and HA, (**F**) Ca_v_1.4 and AP1G1, and (**G**) Ca_v_1.4, VPS35. Nuclei were stained with Hoechst 33342. Representative images are shown. (**E**) *n* = 3 independent experiments. (**F**, **G**) *n* = 1 biological replicate. Single Z-stack is shown. Scale bar = 10 µm. White arrowheads indicate areas of colocalisation between Ca_v_1.4 and HA (**E**) as well as Ca_v_1.4 and AP1G1 (**F**). White box indicates the region shown at higher magnification in (**E**–**G**). Bar graphs in (**H**) show the Pearson’s correlation coefficient between Ca_v_1.4 and the indicated markers. Bars represent the mean; error bars indicate SD. (Ca_v_1.4 + HA) *n* = 198 cells, from 7 images. (Ca_v_1.4 + AP1G1) *n* = 192 cells, from 3 images. (Ca_v_1.4 + VPS35) *n* = 138 cells, from 2 images. *p < 0.05. ns = not significant.

To answer whether the effect of Ca_v_1 channel blockade via nifedipine on CTL killing is achieved through Gli1 or an independent mechanism, we treated *Gli1* WT and *Gli1* KO CTLs with nifedipine or carrier control (Fig. 4A). We find that nifedipine treatment leads to a defect in killing in *Gli1* WT CTLs, which we have shown to be independent of effects on cell viability or MAPK signalling (Figs. EV2E and 3G). This indicates that Ca_v_1 channels are important regulators of CTL killing. Treatment of *Gli1* KO CTLs with nifedipine showed no additive defect in killing compared to carrier control (Fig. 4A), indicating that the presence of Gli1 is required for nifedipine to exert its effect on CTL killing. Thus, we have shown that L-type voltage-gated Ca^2+^ channels are required for CTL killing and that the mechanism for this killing phenotype is by regulating the induction of Gli1.

### Gli1 induction upon TCR signalling is independent of canonical Hh signalling via Ihh and Smo

Given the role of Smo in maintaining Gli1 steady-state protein expression (de la Roche et al, 2013) we asked whether canonical Hh signalling was needed for the induction of Gli1 after TCR stimulation. To investigate this, we generated two different conditional knockout lines. For the first mouse line, we crossed *GzmBER*^*T2*^*Cre* mice (Bannard et al, 2009) to *Smo*^*f/f*^ mice, to generate mice in which Cre is only active in mature CTLs. The second mouse line was generated by crossing *dLckCre* mice to *Ihh*^*f/f*^ mice, in which the Cre is only active from the late stages of T cell development in the thymus. Ihh is the only Hh ligand expressed by CD8^+^ T cells and Hh signalling has been suggested to be cell-autonomous in CD8^+^ T cells (de la Roche et al, 2013) and CD4^+^ T cells (Hanna et al, 2022). We therefore decided to knock out endogenous Ihh in CD8^+^ T cells as well as Smo, the key signal transducer of canonical Hh signalling. Importantly, the peripheral T cell compartment in conditional *Ihh* KO mice and inducible *Smo* KO mice was normal (Fig. EV2F,H). Interestingly, CD8^+^ T cells from *Smo* KO and *Ihh* KO mice showed a modest reduction of *Gli1* mRNA at steady state but upregulated *Gli1* mRNA to the same extent as WT cells upon TCR stimulation (Fig. 4B,C), indicating that Gli1 induction upon TCR signalling is not mediated by canonical Hh signalling.

Ca^2+^ is required for the binding of Ihh to Ptch (McLellan et al, 2008), thought to initiate canonical Hh signalling in CD8^+^ T cells. To confirm that canonical Hh signalling is indeed independent of our newly identified Ca_v_1-regulated Gli1 induction, we treated CTLs from *Ihh* Het and *Ihh* KO mice with nifedipine and U0126. We observed the same levels of inhibition of Gli1 induction in both conditions (Fig. 4C), suggesting that the MAPK-Gli1 and Ca_v_1-Gli1 axes are independent of canonical Hh signalling. We also considered whether overexpression of either Ihh or Smo would further increase Gli1 induction. For this, we transduced primary CD8^+^ T cells with retroviruses encoding Ihh or a constitutively active form of Smo (SmoM2) (Xie et al, 1998), resulting in 80000- and 300-fold overexpression by mRNA, respectively (Fig. EV2H). Under these conditions we did not observe any further increase in the induction of Gli1 as compared to empty vector-transduced control CD8^+^ T cells (Fig. EV2I).

To further confirm that Ca_v_1 and Smo-mediated Gli1 induction are two separate signalling pathways important for killing, we treated CTLs with Ca_v_1 inhibitor nifedipine or Smo inhibitor cyclopamine alone and in combination. As expected, inhibition of either Ca_v_1 or Smo alone led to a significant reduction in tumour cell killing. Combined inhibition however, led to an additive effect further indicating that both pathways operate independently in CTLs (Fig. 4D,E).

Thus, canonical Hh signalling via Ihh and Smo is independent of the MAPK and L-type voltage-gated Ca^2+^ channel-induced Gli1 activation.

### Ca^2+^ flux via Ca_v_1.3 and Ca_v_1.4 is required for Gli1 activation and CTL killing in vitro and in vivo

Having shown that nifedipine-sensitive channels were important for Gli1 activation we sought to confirm that the effect was indeed being specifically mediated by Ca_v_1 channels.

**Figure 5:**
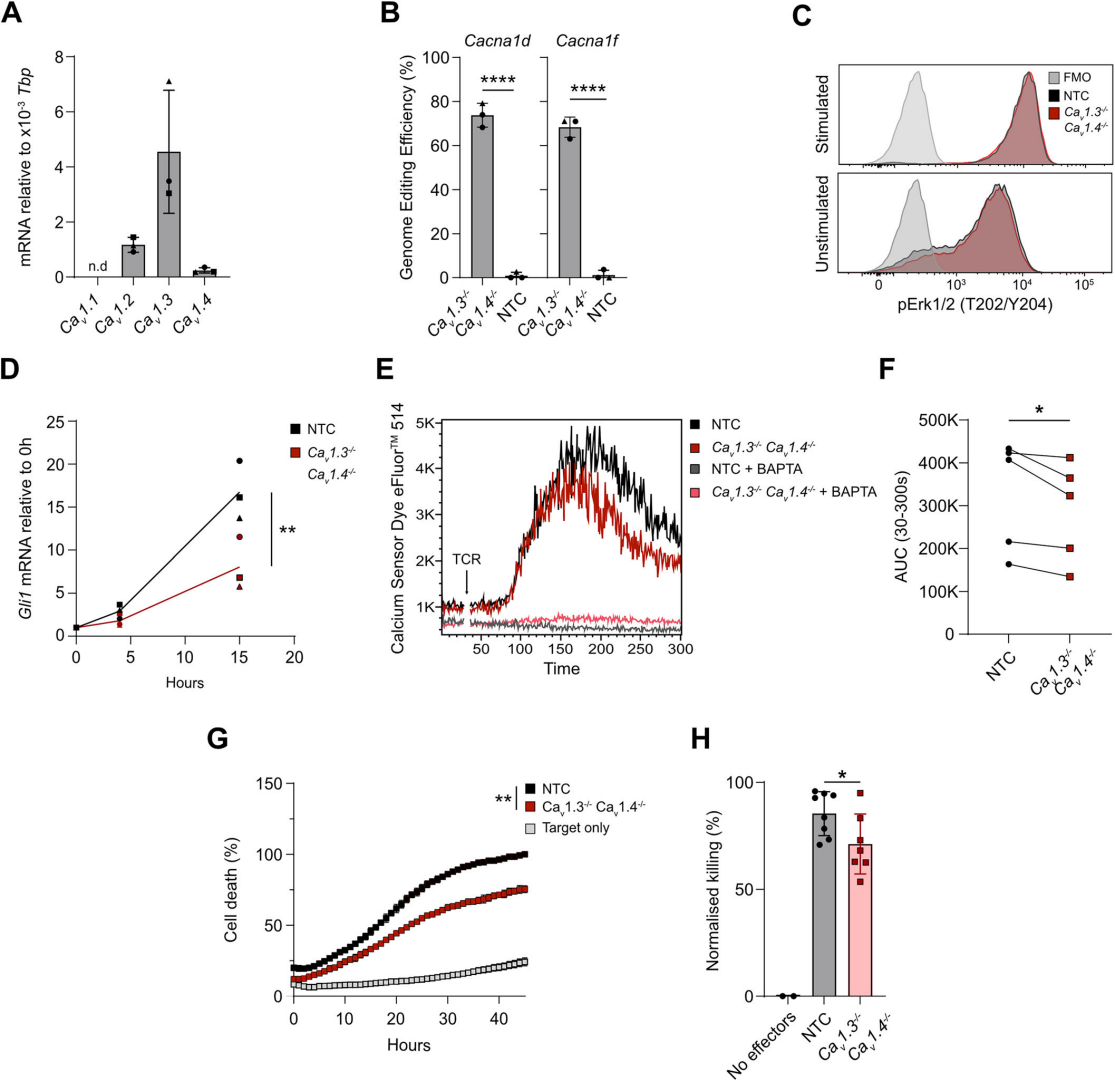
Ca_v_1.3 and Ca_v_1.4 mediate TCR-induced Ca^2+^ flux and Gli1 induction and are important for CTL killing in vitro and in vivo. (**A**) RNA was extracted from murine CTLs on day 8 post stimulation mRNA expression of Ca_v_1 isoforms was determined by qRT-PCR. Data is normalised to *Tbp* as a reference gene. Similar results were obtained when *B2m* was used as a reference gene. *n* = 3 biological replicates from 1 independent experiment. Symbols indicate biological replicates. Bars represent the mean; error bars indicate SD. (**B**–**H**) *OT-I* CD8^+^ T cells were electroporated with RNP complexes on day 2/3 post stimulation to generate *Ca*_*v*_*1.3/1.4*^*-/-*^ (double KO) or non-targeting control (NTC) CTLs. (**B**) PCR amplification of the edited loci was performed prior to a T7EI mismatch cleavage assay to quantify genome editing efficiency on day 8/9 post stimulation. *n* = 3 biological replicates from 3 independent experiments. *p* values were calculated using an unpaired two-tailed Student’s t test (*p* < 0.0001). Symbols indicate biological replicates. Bars represent the mean; error bars indicate SD. (**C**,** D**) CTLs were restimulated on day 8/9 with 10 µg/ml cross-linked soluble anti-CD3ε for flow cytometric analysis of pErk (C) or restimulated with plate-bound anti-CD3ε for mRNA expression of *Gli1* (D). *p* values were calculated using a two-way ANOVA (*p* = 0.0019). *n* = 3 biological replicates from 3 independent experiments. Symbols indicate biological replicates. (**E**) On day 5-7 CTLs were loaded with Calcium Sensor Dye eFluor^TM^ 514 and calcium (Ca^2+^) flux was analysed by flow cytometry. After 30 s, the TCR was cross-linked. The Ca^2+^ chelator BAPTA serves as a negative control. Representative flow cytometry plot is shown. (**F**) Quantification of the area under the curve (AUC) of Ca^2+^ flux of the period after TCR cross-linking (between 30s-300s). *p* values were calculated using a paired two-tailed Student’s t test (*p* = 0.0472). *n* = 5 biological replicates from 3 independent experiments. (**G**) On day 6 post stimulation, CTLs were subjected to an Incucyte cytotoxicity assay with MC38-OvaN4 target cells at an effector:target ratio of 0.5:1. *n* = 2 biological replicates from 2 independent experiments normalised to killing of NTC cells at 45 h time point. Error bars indicate SD. *p* values were calculated using a two-way ANOVA (*p* = 0.0029). (**H**) *Rag2* KO mice were injected with 1.6 × 10^6^ Celltracker^TM^ Violet BMQC-labelled, untransduced EL4 (EL4 UT) and 1.6 × 10^6^ GFP-expressing EL4-OvaN4 cells intraperitoneally (ip). Two hours post tumour cell injection 0.5 × 10^6^ NTC or *Cav1.3/1.4* KO CTLs were adoptively transferred via *i.p*. injection. At endpoint, 20 h post CTL injection, cells were collected from the peritoneal cavity and analysed by flow cytometry. Bar chart showing the ratio between EL4 UT and EL4-OvaN4 normalised to the ratio of PBS (no effector) injected mice. *n* = 2 (no effector), *n* = 8 (NTC), *n* = 7 (Ca_v_1.3/1.4^-/-^) biological replicates from 2 independent experiments. *p* values were calculated using an unpaired two-tailed Student’s t test (*p* = 0.0414). Bars represent the mean; error bars indicate SD. *p < 0.05, **p < 0.01, ****p < 0.0001. Source data are available online for this figure.

First, we assessed that Ca_v_1 channels are expressed in murine CD8^+^ T cells (Erdogmus et al, 2022). We validated our qRT-PCR probes using tissues with reported high Ca_v_ family expression as positive controls (Fig. EV3A) and observed that Ca_v_1.2, Ca_v_1.3 and Ca_v_1.4 are expressed in murine CTLs (Fig. 5A) and Ca_v_1.3 mRNA is upregulated upon TCR stimulation (Fig. EV3B). To test whether the channels were functionally important for Gli1 activation we generated CRISPR knockouts of the Ca_v_1 α_1_ subunit coding genes (*Cacna1c/d/f*) expressed in primary murine CD8^+^ T *OT-I* cells. Knockout of individual genes did not show a significant decrease in Gli1 induction (Fig. EV3C) indicating that there may be a degree of redundancy between individual Ca_v_1 family channels. However, knockout of Ca_v_1.3 and Ca_v_1.4 led to the most reduction in Gli1 levels. Thus, we next performed a double KO of *Cacna1d* and *Cacna1f*, which encode Ca_v_1.3 and Ca_v_1.4, respectively. Double KO T cells presented with high editing efficiency (Fig. 5B), normal cell proliferation (Fig. EV3D), and intact MAPK signalling (Fig. 5C). Notably, double KO CTLs showed impaired upregulation of *Gli1* upon TCR stimulation (Fig. 5D), significantly reduced Ca^2+^ flux upon TCR engagement (Fig. 5E,F) and diminished killing capacity of MC38-OvaN4 tumour cells in vitro (Fig. 5G), phenocopying the data obtained from Ca_v_ inhibitor experiments (Figs. 3D–H and 4A,D,E). To determine whether Ca_v_1.3 and Ca_v_1.4 were also important for CTL killing in vivo we transferred double KO *OT-I *T cells into EL4-Ova tumour bearing mice and observed significantly reduced specific tumour cell killing in vivo (Fig. 5H).

Taken together, we have shown that Ca_v_1 channels are expressed in primary murine T cells and are functional mediators of TCR-induced Ca^2+^ flux. The channels act as the main inducers of Gli1 in naive CD8^+^ T cells and in CTLs act independently of MAPK signalling to induce Gli1. Genetic deletion of *Ca*_*v*_*1.3* and *Ca*_*v*_*1.4* leads to diminished Ca^2+^ flux and Gli1 induction upon TCR activation as well as tumour cell killing confirming the data obtained with Ca_v_1 inhibitor nifedipine.

### Ca_v_1 channels are important for killing and localise to the plasma membrane and intracellular vesicles in human CD8^+^ T cells

**Figure 6:**
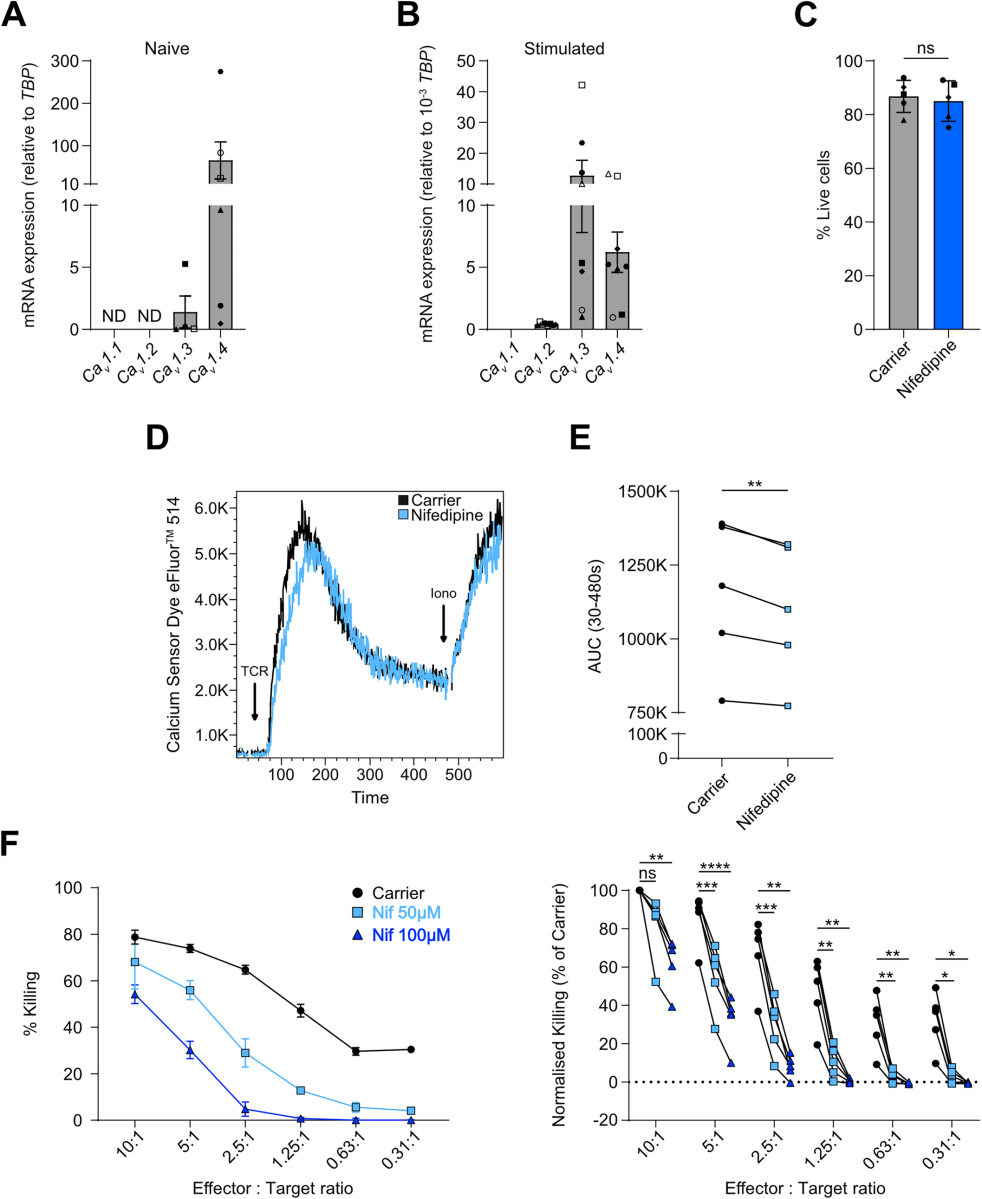
Ca_v_1 family channels are expressed in human T cells and control CTL killing. (**A**,** B**) Naive human CD8^+^ T cells, freshly isolated from peripheral blood of healthy donors (**A**), or human CTLs on day 13–26 post activation (**B**) were processed for qRT-PCR analysis of human Ca_v_1.1 (*CACNA1S*), Ca_v_1.2 (*CACNA1C*), Ca_v_1.3 (*CACNA1D*) and Ca_v_1.4 (*CACNA1F*) channel expression. Values were normalised to *TBP* as a reference gene. Similar results were obtained when *ACTB* was used as a reference gene. ND: not detected. Bars represent the mean; error bars indicate SEM. Each symbol indicates a different healthy donor. (**A**) *n* = 7 (**B**) *n* = 8 biological replicates from 2 independent experiments. (**C**) Naive human CD8^+^ T cells were isolated from PBMCs of healthy donors and stimulated with human T-activator CD3/CD28 beads for 3 days in the presence of nifedipine or carrier control before flow cytometric analysis for cell viability. Bars represent the mean; error bars indicate SD. Statistical significance was assessed by a paired two-tailed Student’s t test. *n* = 5 biological replicates. Each symbol indicates a different healthy donor. (**D**,** E**) Human CD8^+^ T cells were isolated from peripheral blood of healthy donors and CTLs were generated. On day 12–15 post stimulation CTLs were loaded with Calcium Sensor Dye eFluor^TM^ 514 and Ca^2+^ flux was assessed by flow cytometry in the presence or absence of 50 μM nifedipine. After 30 s, TCR cross-linking was induced by antibody cross-linking. After 8 min, ionomycin was added to measure maximal Ca^2+^ flux. (**D**) Representative flow cytometry plot is shown. (**E**) Quantification of the AUC of Ca^2+^ flux of the period after TCR cross-linking (between 30 s–480 s). *n* = 5 biological replicates from 4 independent experiments. *p* values were calculated using a paired two-tailed Student’s t test (*p* = 0.01). (**F**) T cells were stimulated for 3 days and again restimulated on d10 for 3 days. On d14–d15, CD8^+^ CTLs were co-cultured with P815 target cells in the presence of the indicated concentrations of nifedipine or carrier control at the indicated effector to target ratios and subjected to an LDH cytotoxicity assay. Left panel: representative donor shown. Right panel: data points represent mean of three technical replicates; error bars indicate SD, *n* = 5 biological replicates from 3 independent experiments. *p* values were calculated using a two-way ANOVA with Tukey’s multiple comparisons test (10:1 *p* = 0.0079, 5:1 *p* = 0.0008 & *p* < 0.0001, 2.5:1 *p* = 0.007 & *p* = 0.0016, 1.25:1 *p* = 0.0029 & *p* = 0.0075, 0.625:1 *p* = 0.0153 & *p* = 0.0179, 0.3125:1 *p* = 0.0109 & *p* = 0.0165). *p < 0.05, **p < 0.01, ***p < 0.001, ****p < 0.0001. ns = not significant. Source data are available online for this figure.

To interrogate whether Ca_v_1 channels are also expressed in human CD8^+^ T cells we profiled the expression of Ca_v_1 α_1_ subunit coding genes (*CACNA1S/C/D/F* corresponding to Ca_v_1.1, Ca_v_1.2, Ca_v_1.3, and Ca_v_1.4, respectively) in naive and activated CD8^+^ (Fig. 6A,B) T cells from multiple healthy donors. Ca_v_1.3 and Ca_v_1.4, the two subunits responsible for Gli1 induction in murine T cells were readily detected in human T cells, while Ca_v_1.1 was not expressed and low expression of Cav1.2 could only be detected in activated human T cells.

Next, we wanted to know whether Ca_v_1 channels are implicated in the effector functions of human CD8^+^ T cells. The concentration of nifedipine used had no effect on CTL cell viability (Fig. 6C), but Ca^2+^ flux in CTLs following TCR cross-linking was significantly reduced upon Ca_v_1 channel blockade (Fig. 6D,E). To measure tumour cell killing in vitro, we treated human CTLs from multiple healthy donors with nifedipine or carrier control before subjecting them to a killing assay. While carrier-treated human CTLs were able to efficiently kill tumour targets, nifedipine-treated CTLs from all donors tested showed a marked dose-dependent decrease in killing ability (Fig. 6F).

**Figure 7:**
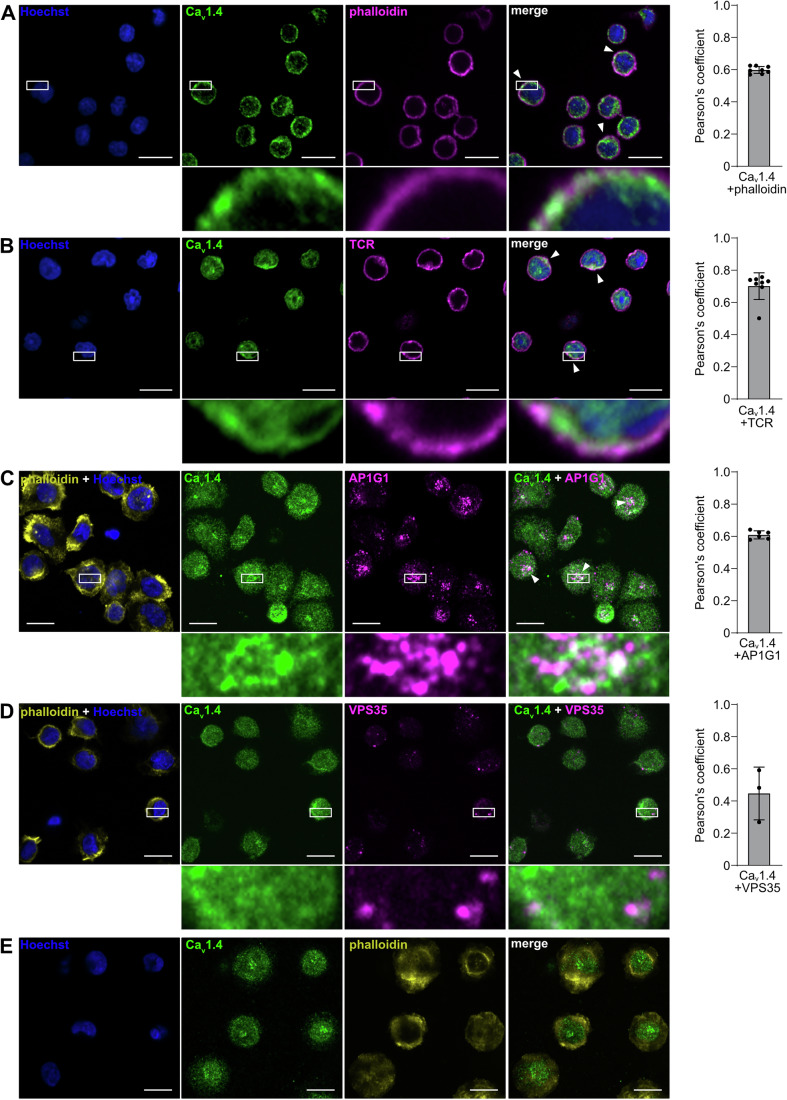
Ca_v_1.4 colocalises with the TCR at the plasma membrane and the endosomal marker AP1G1 in human CTLs. Human CD8^+^ T cells were stimulated with soluble anti-CD3/CD28/CD2 antibodies for 72 h for CTL generation. Between day 9 and 11 of culture, CTLs were fixed and stained with (**A**) phalloidin and antibodies against Ca_v_1.4, (**B**) antibodies against Ca_v_1.4 and TCR, (**C**) phalloidin and antibodies against Ca_v_1.4 and AP1G1, (**D**) phalloidin and antibodies against Ca_v_1.4 and VPS35, or (**E**) phalloidin and antibodies against Ca_v_1.4. Nuclei were stained with Hoechst 33342. For (**E**), CTLs were plated on anti-CD3ε-coated wells for 4 min prior to fixation to generate immune synapses. (**A**) *n* = 8 biological replicates, (**B**) *n* = 3 biological replicates, (**C**,** D**) *n* = 2 biological replicates, (**E**) *n* = 4 biological replicates. Single Z-stack is shown. Representative donor shown. Scale bar = 10 μm. White arrowheads indicate areas of colocalisation between Ca_v_1.4 and phalloidin (**A**), Ca_v_1.4 and TCR (**B**), Ca_v_1.4 and AP1G1 (**C**). White box indicates the region shown at higher magnification in (**A**–**D**). Bar graphs show the Pearson’s correlation coefficient between Ca_v_1.4 and the indicated markers. Bars represent the mean; error bars indicate SD. (**A**) *n* = 562 cells, from 8 images. (**B**) *n* = 568 cells, from 8 images. (**C**) *n* = 132 cells, from 6 images. (**D**) *n* = 74 cells, from 3 images. Source data are available online for this figure.

Ca_v_1 channels have been argued to localise to the plasma membrane in T cells (Omilusik et al, 2011) but have never been localised by immunofluorescence. Since we detected high Ca_v_1.4 expression in naive and activated human T cells, we explored the subcellular localisation of Ca_v_1.4 by confocal microscopy. First, we validated the specificity of the Ca_v_1.4 antibody in HEK293T cells overexpressing a HA-tagged human Ca_v_1.4 construct (Fig. EV3E,H). Having shown specificity of the Ca_v_1.4 antibody, we stained human CD8^+^ T cells from different healthy donors and observed Ca_v_1.4 staining at the plasma membrane as well as on intracellular vesicles in all donors (Fig. 7A). Interestingly, we found Ca_v_1.4 in close juxtaposition with the TCR on the plasma membrane of human CD8^+^ T cells at steady state, indicating that indeed the TCR in T cells might be able to directly activate the channel (Fig. 7B). To further characterise the intracellular localisation of Ca_v_1.4, we stained for markers of vesicular transport: (I) AP-1 complex subunit gamma-1 (AP1G1), which localises to post-golgi compartments (TGN/endosomes) (II) VPS35, a core component of the retromer complex critical for retrieval of transmembrane proteins from endosomes to the Golgi-network or the cell surface. Ca_v_1.4 showed significant colocalisation with AP1G1 in T cells (Fig. 7C) and also HEK293T cells overexpressing Ca_v_1.4 (Fig EV3F,H), but did not colocalise with VPS35-positive vesicles in either human T or HEK293T cells (Figs. 7D and EV3G,H), indicating that Ca_v_1.4 localises to endosomes but might not be recycled to the plasma membrane at steady state.

To understand if Ca_v_1.4 localisation changes upon immune synapse formation, we generated immune synapses on glass coated with anti-CD3ε antibodies (Fig. 7E). Notably, Ca_v_1.4 accumulated in clusters at the central actin-depleted signalling region of the immune synapse. This suggests that Ca_v_1.4 channels are positioned ideally for active involvement in TCR-driven signalling at the immune synapse.

Taken together, we have shown that, similarly to murine CD8^+^ T cells, human CD8^+^ T cells express Ca_v_1 channels and depend on Ca_v_1 channels for target cell killing. Ca_v_1.4 channels in human T cells are located in close proximity to the TCR complex on the plasma membrane and on endosomes and cluster at the centre of the immune synapse, indicating that these channels are dynamically recruited to the site of TCR engagement, where they can be activated by PKC.

### Small molecule Ca_v_1 agonist increases killing capacity of mouse and human CTLs

**Figure 8:**
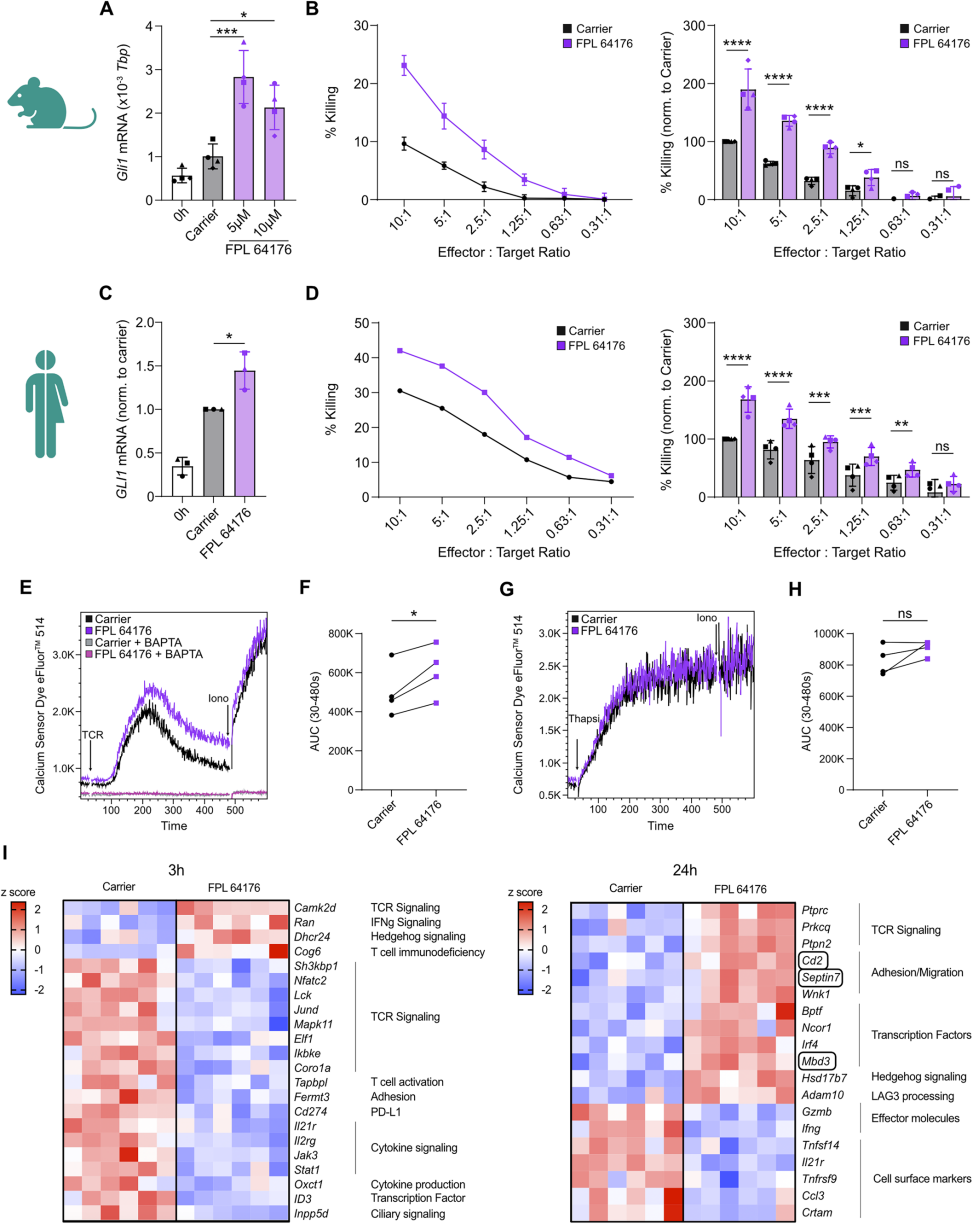
L-type voltage-gated Ca^2+^ channel agonist FPL 64176 enhances Gli1 induction and killing capacity of murine and human CD8^+^ T cells by maintaining an increased Ca^2+^ flux. (**A**) Murine *OT-I* CTLs were restimulated on day 9/10 for 3 h with plate-bound anti-CD3ε in the presence of the indicated concentrations of FPL 64176 or carrier control before RNA was extracted for qRT-PCR analysis. *n* = 4 biological replicates from 2 independent experiments. Data is normalised to *Tbp* as a reference gene. Similar results were obtained when *CD3ε* was used as a reference gene. Bars represent the mean; error bars indicate SD. *p* values were calculated using a one-way ANOVA with Tukey’s multiple comparison test (Carrier vs 5 µM *p* = 0.0009, Carrier vs 10 µM *p* = 0.0201). (**B**) On day 7 post stimulation, murine CTLs were co-cultured with Ova-pulsed EL4 tumour target cells in the presence of 10 µM FPL 64176 or carrier control at the indicated effector to target ratios. LDH cytotoxicity assay was performed after 3 h. Representative assay (left, data points represent mean of three technical replicates; error bars indicate SD) and quantification of *n* = 4 biological replicates from 2 independent experiments normalised to killing of carrier-treated cells at a 10:1 effector:target ratio (right, bars represent the mean; error bars indicate SD) are shown. *p* values were calculated using a two-way ANOVA with Sidak’s multiple comparison test (10:1 *p* < 0.0001, 5:1 *p* < 0.0001, 2.5:1 *p* < 0.0001, 1.25:1 *p* = 0.0406). (**C**,** D**) Human CD8 + T cells were isolated from peripheral blood of healthy donors and CTLs were generated. (**C**) Human CTLs were restimulated on day 15–20 for 24 h with plate-bound anti-CD3ε in the presence of 10 µM of FPL 64176 or carrier control before RNA was extracted for qRT-PCR analysis. *n* = 3 biological replicates from 2 independent experiments. Data is normalised to *TBP* as a reference gene. Similar results were obtained when *CD3ε* was used as a reference gene. Bars represent the mean; error bars indicate SD. *p* values were calculated using a one-way ANOVA with Tukey’s multiple comparison test (*p* = 0.0171). (**D**) On day 16–24, CD8^+^ T cells were co-cultured with P815 target cells at indicated effector to target ratios for 3 h and subjected to a flow-cytometry-based cytotoxicity assay in the presence of 10 µM FPL64176 or carrier control. Representative killing assay (left) and quantification of *n* = 4 biological replicates from 2 independent experiments normalised to killing of carrier-treated cells at a 10:1 effector:target ratio (right) are shown. Bars represent the mean; error bars indicate SD. *p* values were calculated using a two-way ANOVA with Sidak’s multiple comparison test (10:1* p* < 0.0001, 5:1 *p* < 0.0001, 2.5:1 *p* < 0.0004, 1.25:1 *p* = 0.0003, 0.625:1 *p* = 0.0092). (**E**–**H**) On day 6–8 poststimulation murine CTLs were loaded with Calcium Sensor Dye eFluor^TM^ 514 and Ca^2+^ flux was analysed by flow cytometry in the presence or absence of 10 µM FPL 64176. After 30 s, TCR cross-linking was induced with IgG antibodies (**E**,** F**) or store-operated Ca^2+^ entry was induced by 1 µM thapsigargin (**G**, **H**). After 8 min, ionomycin was added to measure maximal Ca^2+^ flux. Conditions with the Ca^2+^ chelator BAPTA serve as negative controls. Representative flow cytometry plots shown (**E**,** G**). (**F**) Quantification of the area under the curve (AUC) of Ca^2+^ flux of the period after TCR cross-linking (between 30 s and 480 s). *n* = 4 biological replicates from 4 independent experiments. *p* values were calculated using a paired two-tailed Student’s t test (*p* = 0.0295). (**H**) Quantification of the AUC of Ca^2+^ flux of the period after thapsigargin addition (between 30 s and 480 s). *n* = 4 biological replicates from 4 independent experiments. *p* values were calculated using a paired two-tailed Student’s t test (*p* = 0.1506). (**I**) Murine naive CD8^+^ T cells were stimulated for 3 h and 24 h with plate-bound anti-CD3ε and anti-CD28 antibodies in the presence of 10 mM FPL 64176 or carrier control before RNA was extracted for RNA-Seq analysis. Heatmap depicting a selection of biologically relevant, statistically significant differentially downregulated and upregulated genes, respectively. *n* = 6 biological replicates. *p < 0.05, **p < 0.01, ****p < 0.0001. Symbols indicate biological replicates or individual human donors. ns = not significant. Boxes indicate genes with one of 11 Gli consensus sequences as shown in Appendix Table S8 (Winklmayr et al, 2010). Source data are available online for this figure.

**Figure EV4:**
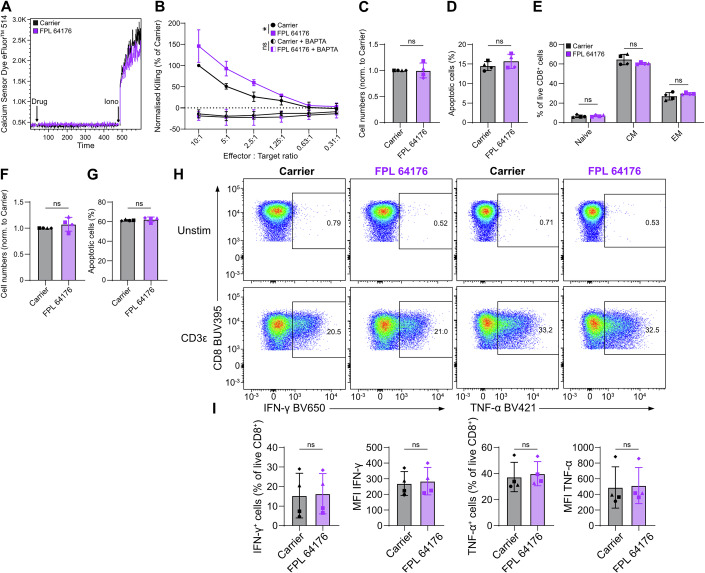
Phenotypic characterisation of FPL 64176-treated murine and human CTLs. (**A**–**I**) FPL 64176 does not lead to a Ca^2+^ response in the absence of TCR stimulation, enhances CTL killing via extracellular Ca^2+^ influx, and does not affect CD8^+^ T cell viability, differentiation, proliferation or cytokine production. (**A**) Murine CTLs on day 6–8 post stimulation were loaded with Calcium Sensor Dye eFluor^TM^ 514, stained with anti-CD8 and anti-CD3e and calcium (Ca^2+^) flux was analysed by flow cytometry. After 30 s, FPL 64176 or carrier solution was added to a final concentration of 10 µM. After 8 min, ionomycin was added to measure maximal Ca^2+^ flux. Data shown is representative of *n* = 4 biological replicates from 4 independent experiments. (**B**) On day 7–8 post stimulation, murine CTLs were co-cultured with ovalbumin-pulsed EL-4 target cells at the indicated effector to target ratios in the presence of 10 µM FPL64176 and/or 1.25 mM BAPTA and their respective carrier controls and subjected to an LDH cytotoxicity assay for 3 h. *n* = 4 biological replicates from 4 independent experiments normalised to killing of carrier-treated cells at a 10:1 effector:target cell ratio. Error bars indicate SD. *p* values were calculated using a two-way ANOVA with E:T ratio and treatment as factors with Dunnett’s multiple comparisons test comparing each treatment condition (*p* = 0.0195). (**C**–**E**) Naive CD8^+^ T cells were stimulated with plate-bound anti-CD3/CD28 antibodies for 24 h in the presence of 10 µM FPL64176 or carrier control before flow cytometric analysis. Bars represent the mean; error bars indicate SD. (**C**) Quantitative analysis of live cell numbers normalised to carrier *n* = 4 biological replicates from 2 independent experiments. Statistical significance was assessed using a two-tailed one sample t test. (**D**) Percentage of apoptotic cells as detected by Apotracker™ Green staining *n* = 4 biological replicates from 2 independent experiments. Statistical significance was assessed using a two-tailed paired Student’s t test. (**E**) Analysis of T cell subsets based on the expression of CD44 and CD62L: CD44^-^ CD62L^+^ (naive), CD44^+^ CD62L^+^ (CM = central memory), and CD44^+^ CD62L^-^ (EM = effector memory). *n* = 4 biological replicates from 2 independent experiments. Statistical significance was assessed using a two-way ANOVA with Sidak’s multiple comparison test. (**F**, **G**) CTLs were restimulated on day 10 with plate-bound anti-CD3ε antibody for 24 h in the presence of 10 µM FPL64176 or carrier control before flow cytometric analysis. Quantitative analysis of live cell numbers normalised to carrier condition (**F**) and percentage of apoptotic cells as detected by Apotracker™ Green staining (**G**) are shown. *n* = 4 biological replicates from 2 independent experiments. Bars represent the mean; error bars indicate SD. Statistical significance was assessed using a two-tailed one sample t test (**F**) or two-tailed paired Student’s t test (**G**). (**H**, **I**) Between day 11 and 13 post stimulation, human CTLs were left unstimulated or were re-stimulated with 1 µg/mL plate-bound anti-CD3ε antibodies for 4.5 h in the presence of 10 µM FPL64176 or carrier control. IFN-γ and TNF-α production was assessed by intracellular cytokine staining. Representative flow cytometry plots (top) and quantification of percentages and median fluorescence intensity (MFI) (bottom) are shown. *n* = 4 individual healthy donors from 2 independent experiments. Symbols indicate individual healthy donors. Bars represent the mean and error bars indicate SD. Statistical significance was assessed using a two-tailed paired Student’s t test. *p < 0.05. ns = not significant.

CD8^+^ T cell function is often impaired in the tumour microenvironment, which represents a major challenge for CAR T cell therapy (Majzner and Mackall, 2019). After having found that Ca_v_1 channel-mediated Gli1 induction is required for effective CD8^+^ T cell killing, we next sought to investigate whether agonists of Ca_v_1 channels would further enhance Gli1 induction and thereby amplify tumour cell killing. For these experiments we stimulated both murine (Fig. 8A) and human (Fig. 8C) CTLs in the presence of the highly selective and potent Ca_v_1 agonist, FPL 64176 (Baxter et al, 1993) or carrier control and observed a significant increase in Gli1 mRNA induction. Strikingly, in the presence of Ca_v_1 agonist FPL 64176 the killing capacity of both murine (Fig. 8B) and human (Fig. 8D) CTLs was enhanced nearly 100% in a TCR- and Ca^2+^-dependent manner (Fig. EV4A,B).

To investigate whether treatment with FPL 64176 had indeed resulted in an increased, TCR-induced Ca^2+^ flux in CTLs we measured Ca^2+^ fluxes upon both, TCR and thapsigargin stimulation. Thapsigargin leads to store-operated calcium entry (SOCE) by inhibiting the reuptake of Ca^2+^ into the ER without engagement of the TCR and its associated pathways. CTLs in the presence of FPL 64176 demonstrated a significantly increased Ca^2+^ flux after stimulation (Fig. 8E,F) while thapsigargin-mediated Ca^2+^ flux was unchanged (Fig. 8G,H), indicating that FPL 64176-mediated Ca^2+^ flux is TCR-dependent but independent of SOCE.

Since the Gli1 target gene programme in other cell types include cell cycle and anti-apoptosis genes, we further investigated whether FPL 64176 treatment could also increase proliferation, decrease apoptosis and influence the memory phenotype of CD8^+^ T cells. However, no significant effect of FPL 64176 treatment on cell numbers, apoptosis or memory phenotype was observed in vitro (Fig. EV4C–G). Given the established role of Ca^2+^ signalling in regulating effector cytokine production in T cells (Trebak and Kinet, 2019), we also assessed IFN-γ and TNF-α expression by human CTLs upon restimulation. No significant differences in cytokine production were detected in the presence of FPL 64176 (Fig. EV4H,I) supporting our data that Hh signalling via Gli1 does not affect cytokine production in CTLs (de la Roche et al, 2013).

To elucidate possible targets downstream of the Ca_v_1-Gli1 axis we performed RNA-Seq on naive murine T cells upon TCR engagement in the presence of FPL 64176 or carrier control at 3 h to assess early response genes and at 24 h to assess late response genes (Fig. 8I; Appendix Figs. S1 and S2). At the 3 h timepoint a number of FPL 64176-modulated genes involved TCR signalling including upregulation of Calcium/Calmodulin-dependent protein kinase II (Lin et al, 2005) and Protein Kinase C theta (Isakov and Altman, 2012), while downregulating key negative regulators of TCR signalling such as IκB Kinase ε (Zhang et al, 2016) and Coronin 1 (Jayachandran et al, 2019). FPL 64176 was also able to downregulate negative regulators of T cell function such as *Tapbpl* (Lin et al, 2021). At the 24 h timepoint, the number of genes upregulated by FPL 64176 further increased and included genes involved in T cell adhesion/co-stimulatory signalling such as *Cd2* (Zhu et al, 2006) and cytoskeletal rearrangement during migration such as *Septin7* (Zhovmer et al, 2024) and *Wnk1* (Kochl et al, 2016). Target genes also included transcription factors required for T cell activation and effector function such as Mbd family members (Kersh, 2006) as well as transcription factors regulating T cell survival and effector differentiation including Bptf (Wu et al, 2016), Ncor1 (Hainberger et al, 2020) and Irf4 (Man et al, 2013; Yao et al, 2013). Interestingly, both at early and late timepoints FPL 64176 upregulated cholesterol biosynthesis genes including hydroxysteroid 17β dehydrogenase 7 (Stottmann et al, 2011) and 24 dehydrocholesterol reductase (Qiu et al, 2020) which have both been shown to be bona fide activators of the Hedgehog pathway. Critically, at the 24 h timepoint a large number of FPL 64176 regulated genes harbour described canonical Gli1 binding sites (Winklmayr et al, 2010) around their transcriptional start site including *Septin7*, *Cd2* and *Mbd3* (Appendix Table S8, Appendix Table S9).

Taken together, we have discovered a unique way to specifically increase CTL killing without affecting survival, proliferation or differentiation phenotype of cytotoxic T cells by activating Ca_v_1 channels.

### Small molecule Ca_v_1 agonist FPL 64176 enhances killing capacity of cytotoxic CD4^+^ T cells and human γδ T cells

To investigate whether the previously unknown Ca_v_1-Gli signalling axis we have discovered was active in only CD8 T cells or might be important for cytotoxic lymphocytes in general, we explored Gli-dependency and FPL 64176 effects in killing capacity of cytotoxic CD4^+^ T and γδ T cells.

**Figure 9:**
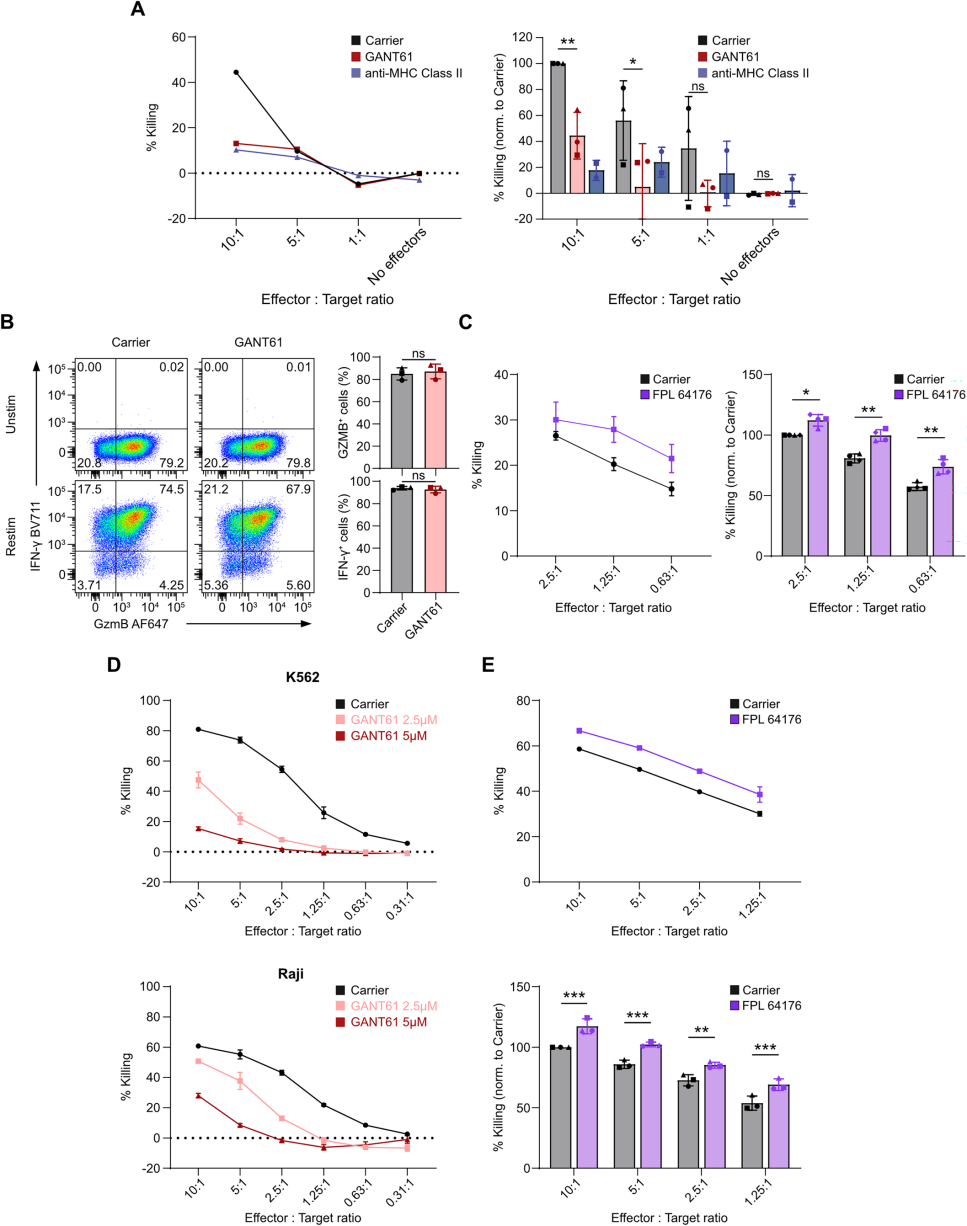
Gli1 controls killing in cytotoxic CD4^+^ T cells with L-type voltage-gated Ca^2+^ channel agonism able to enhance the killing capability of other cytotoxic T lymphocytes. (**A**, **B**) Cytotoxic CD4^+^ T cells were generated from *OT-II* mice and treated with the Gli inhibitor GANT61 at 5 µM or carrier control 24 h prior to a killing assay of Ova-pulsed B cell targets on day 7. (**A**) Pooled killing assays including anti-MHC Class II negative control. *n* = 2–3 biological replicates from 2–3 independent experiments. Bars represent the mean; error bars shown are SD. *p* values were calculated using a two-way ANOVA with Tukey’s multiple comparison test (10:1 *p* = 0.008, 5:1 *p* = 0.0135). (**B**) IFN-γ and Granzyme B levels were assessed by flow cytometry at day 8 in unstimulated (top panel) or restimulated CD4^+^ T cells (bottom panel). Quantification of steady-state Granzyme B levels and IFN-γ levels upon restimulation are shown on the right. Data shown is pooled from *n* = 3 biological replicates from 3 independent experiments. Bars represent the mean; error bars shown are SD. Statistical significance was assessed by a paired two-tailed Student’s t test. (**C**) On day 7, CD4^+^ T cells were co-cultured with P815 target cells at indicated effector to target ratios and subjected to a flow-cytometry-based cytotoxicity assay in the presence of 10 µM FPL 64176 or carrier control at 20 h. Top panel shows a representative killing assay (Data points represent mean of two technical replicates; error bars indicate SD). Bottom panel shows quantification of *n* = 4 biological replicates from 2 independent experiments normalised to killing of carrier-treated cells at a 2.5:1 effector:target ratio. Bars represent the mean; error bars shown are SD. p values were calculated using a two-way ANOVA with Šídák’s multiple comparisons test (2.5:1 *p* = 0.007, 1.25:1 *p* = 0.0007, 0.625:1 *p* = 0.0017). (**D**) On day 19, human Vγ9Vδ2^+^ T cells were co-cultured with zoledronate-pulsed K562 and Raji target cells at the indicated effector to target ratios and subjected to an LDH cytotoxicity assay in the presence of the indicated concentrations of GANT61 or carrier control at 5 h. Representative killing assays from *n* = 2 biological replicates from 2 independent experiments. Data points represent mean of two technical replicates; error bars indicate SD. (**E**) On day 23–24, human Vγ9Vδ2^+^ T cells were co-cultured with P815 target cells at indicated effector to target ratios and subjected to a flow-cytometry-based cytotoxicity assay in the presence of 10 µM FPL 64176 or carrier control at 4.5 h. Top panel shows a representative killing assay. Data points represent mean of two technical replicates; error bars indicate SD. Bottom panel shows quantification of *n* = 3 biological replicates from 2 independent experiments normalised to killing of carrier-treated cells at a 10:1 effector:target ratio. Bars represent the mean; error bars shown are SD. *p* values were calculated using a two-way ANOVA with Šídák’s multiple comparisons test (10:1 *p* = 0.0005, 5:1 *p* = 0.0006, 2.5:1 *p* = 0.0028, 1.25:1 *p* = 0.001). Symbols indicate biological replicates or individual human donors. *p < 0.05, **p < 0.01, ***p < 0.001. ns = not significant. Source data are available online for this figure.

Cytotoxic CD4^+^ T cell kill via granzyme/perforin release and Fas/FasL interactions like CD8^+^ CTLs but are MHC Class II restricted (Takeuchi and Saito, 2017). The cells have been shown to develop in vivo during cytomegalovirus infection (van Leeuwen et al, 2004) and are critical for the clearance of Influenza A infection (Brown et al, 2012) and tumour control in a B16 melanoma model (Quezada et al, 2010). After generation of cytotoxic CD4^+^ T cells in vitro, we treated the cells with the potent Gli antagonist GANT61 and abrogated the killing ability of these cells (Fig. 9A) without affecting IFN-γ and GzmB production indicating that cytotoxic CD4^+^ T cells rely on Gli1 for killing (Fig. 9B). Importantly, when cytotoxic CD4^+^ T cells were treated with FPL 64176 we observed a consistent enhancement killing capacity of 10–30% (Fig. 9C).

**Figure EV5:**
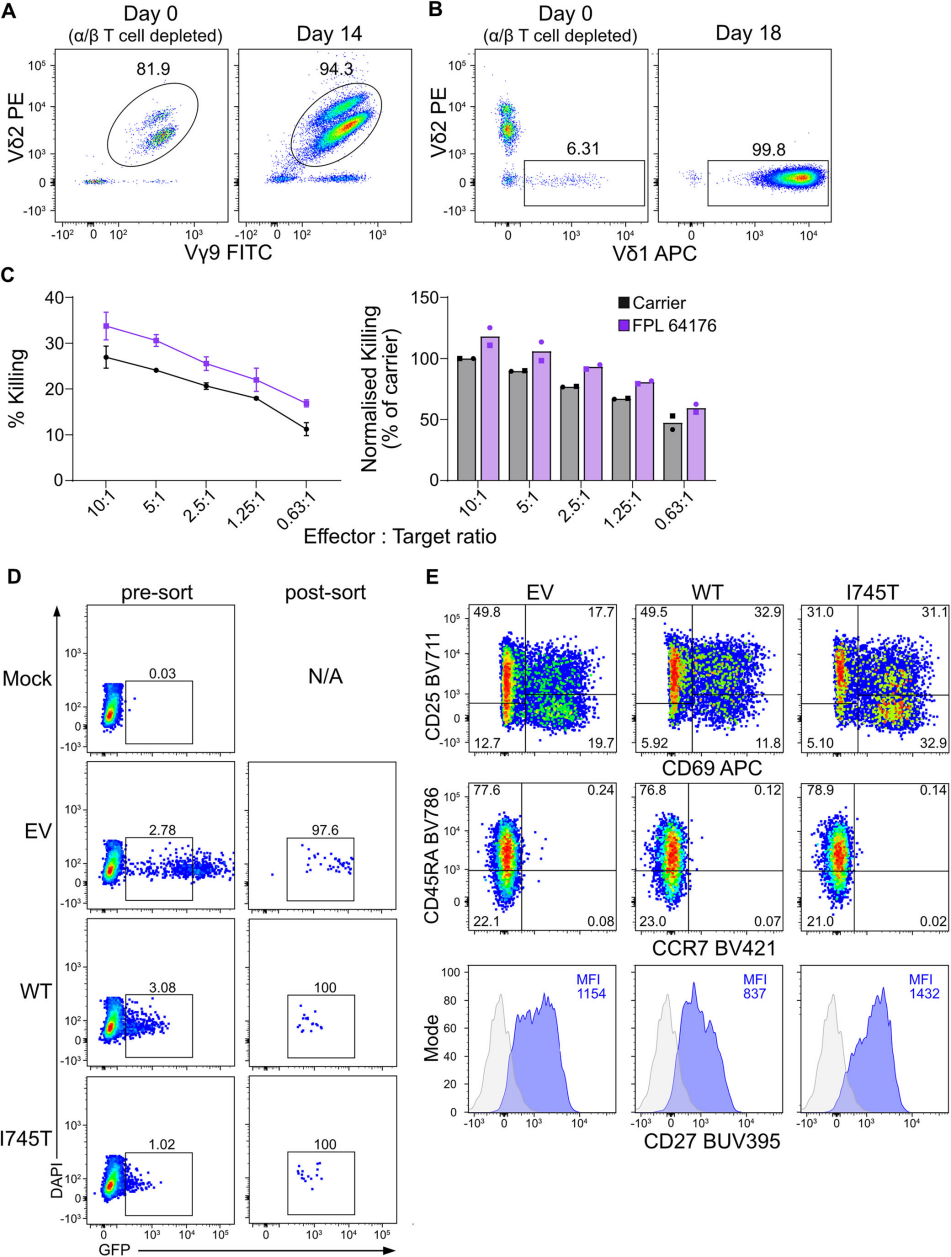
Purity of expanded human Vγ9Vδ2^+^ and Vδ1^+^ T cells, effect of FPL 64176 on the killing capacity of Vδ1^+^ cells, purity and characterisation of human CD8^+^ T cells expressing Ca_v_1.4 constructs. (**A**–**C**) L-type voltage-gated Ca^2+^ channel agonist FPL 64176 enhances the killing capability of Vδ1^+^ T lymphocytes. (**A**) Human Vγ9Vδ2^+^ cells were expanded from αβ T cell-depleted PBMCs using 1 µM zoledronate before checking purity by flow cytometry on day 14. (**B**) Human Vδ1^+^ cells were expanded from αβ T cell-depleted PBMCs using anti-CD3ε antibodies and cytokines before checking purity by flow cytometry on day 18. (**C**) On day 22, human Vδ1^+^ T cells were co-cultured with P815 target cells at indicated effector to target ratios for 3 h and subjected to a flow cytometry-based cytotoxicity assay in the presence of 10 µM FPL 64176 or carrier control. Left panel shows a representative killing assay. Data points represent mean of two technical replicates; error bars indicate SD. Right panel shows quantification of *n* = 2 biological replicates from 1 independent experiment normalised to killing of carrier-treated cells at a 10:1 effector:target ratio. Bars represent the mean. (**D**,** E**) Sorting of human CD8^+^ T cells expressing Ca_v_1.4 constructs and characterization of activation and differentiation markers. (**D**) Human CTLs were electroporated with PiggyBac vectors encoding GFP only (EV), human Ca_v_1.4 (WT), or human Ca_v_1.4 harbouring the gain-of-function mutation I745T (I745T) 48 h post stimulation and sorted between d17–28 of CTL culture based on GFP expression levels. Representative flow cytometry plots pre- and post-sorting are shown. *n* = 7 biological replicates from 5 independent experiments. (**E**) Sorted human CTLs expressing EV, WT, or I745T were restimulated for 72 h on day 19–20 of culture using plate-bound anti-CD3ε before being analysed by flow cytometry for activation and differentiation markers on day 28. Top Expression of CD25 and CD69 (top panel), CCR7 and CD45RA (middle panel) and CD27 (bottom panel) are shown. FMO control is shown in grey. Data shown is from *n* = 2 healthy donors.

γδ T cells are unconventional T lymphocytes that share many features with their αβ counterparts including cytotoxic effector functions and clonal expansion but lack the need for classic MHC-mediated antigen presentation (Bigby et al, 1993). Instead, they can respond to infected or tumour cells in an innate fashion via various receptor-ligand interactions (Shafi et al, 2011). The most abundant subpopulations in humans are Vγ9Vδ2^+^ and Vδ1^+^ cells. Both subsets have been shown to be important in anti-tumour immunity (de Vries et al, 2023; Nguyen et al, 2022) and their promising therapeutic potential in cancer immunotherapy is being actively explored. After isolation and expansion of Vγ9Vδ2^+^ cells to high purity (Fig. EV5A), we asked whether the cells depend on GLI for their killing potential. Strikingly, Vγ9Vδ2^+^ killing of zoledronate-pulsed K562 and Raji target cells was diminished in a dose-dependent fashion in the presence of the GLI inhibitor GANT61 (Fig. 9D). To investigate whether target cell killing could be amplified by Ca_v_1 agonism, we assessed P815 target cell killing in the presence of FPL 64176. FPL 64176 robustly led to a 17–30% increase in tumour cell killing (Fig. 9E). In addition, we isolated and expanded highly pure Vδ1^+^ T cells (Fig. EV5B) and showed that also in these cells FPL 64176 treatment led to a 18–25% increase in tumour cell killing (Fig. EV5C).

This data indicates that Ca_v_1-Gli-signalling axis is not only required for optimal killing of cytotoxic CD8^+^ T cells but also plays an important role in the killing capacity of cytotoxic CD4^+^ T cells as well as Vγ9Vδ2^+^ and Vδ1^+^ T cells and killing capacity can be amplified by Ca_v_1 agonist FPL 64176.

### Human CD8^+^ CTLs expressing a Ca_v_1.4 gain-of-function variant have enhanced killing ability

**Figure 10:**
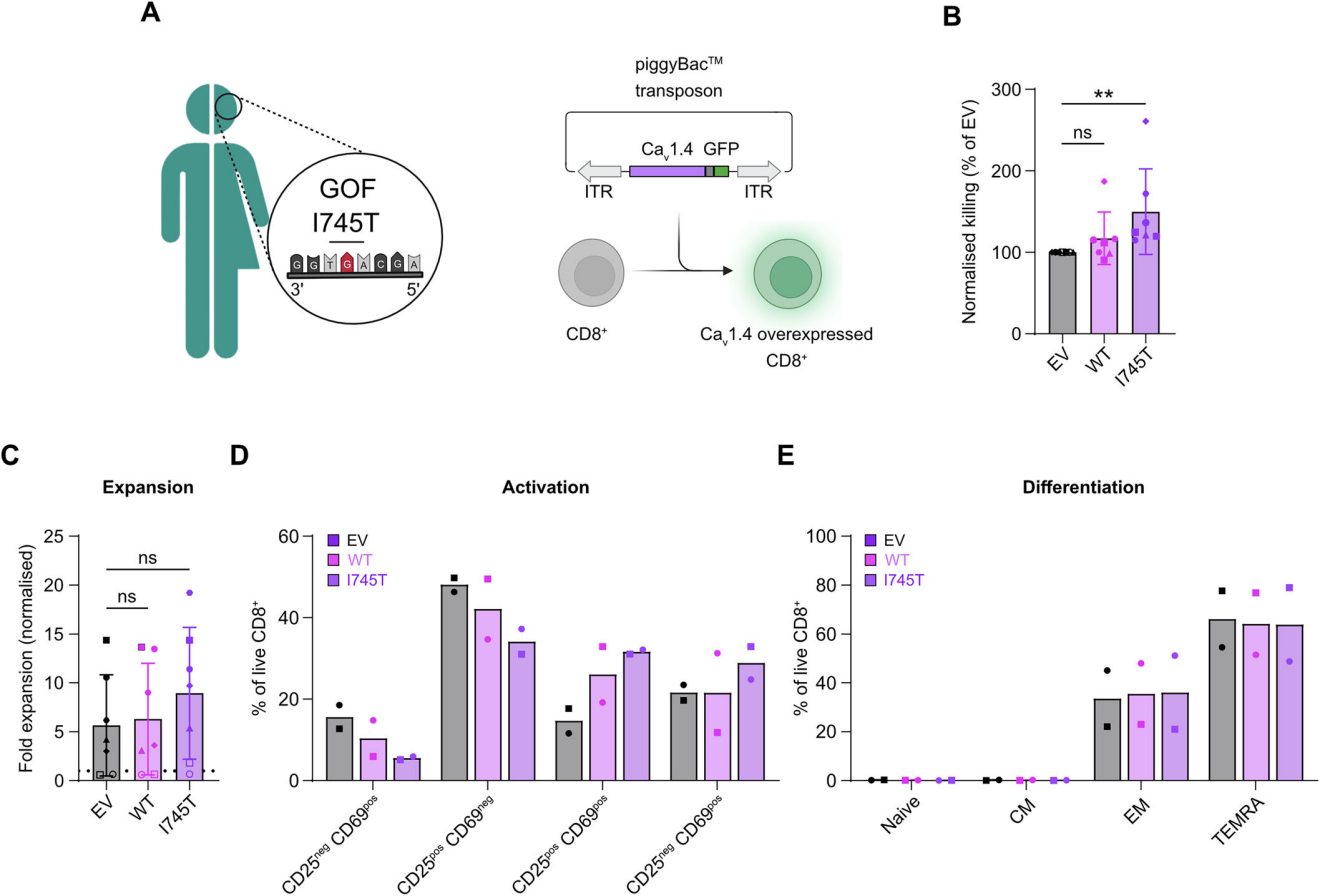
Overexpression of a gain-of-function Ca_v_1.4 construct leads to increased killing ability of human CD8^+^ T cells without affecting their expansion, activation and differentiation characteristics. (**A**) Schematic overview of Ca_v_1.4 construct overexpression in human CD8^+^ T cells based on the gain-of-function mutation I745T identified in Hope et al, 2005. Ca_v_1.4 constructs were introduced into human CTLs via electroporation using the PiggyBac transposon system. Created with BioRender.com. (**B**) On day 26–34, human CTLs cells expressing Ca_v_1.4 constructs were co-cultured with P815 target cells for 3 h at 5:1 effector to target ratio and subjected to a flow cytometry-based cytotoxicity assay. *n* = 7 biological replicates from 5 independent experiments normalised to killing of empty vector (EV) construct expressing cells. Bars represent the mean; error bars shown are SD. *p* values were calculated using a Friedman test with Dunn’s multiple comparisons test (*p* = 0.0099). (**C**) Human CTLs expressing Cav1.4 constructs were sorted based on GFP expression and restimulated with plate-bound anti-CD3ε antibodies for 48–72 h and counted again on day 8–12 after sorting. *n* = 7 biological replicates from 5 independent experiments normalised to the number of cells obtained on the day of sorting. Bars represent the mean; error bars shown are SD. Statistical significance was assessed using a one-way ANOVA with Tukey’s multiple comparisons test. (**D, E**) Sorted Human CTLs expressing Cav1.4 constructs were analysed by flow cytometry for activation and differentiation markers 8–9 days post restimulation with plate-bound anti-CD3ε for 72 h. (**D**) Quantitative analysis of percentage of cells expressing activation markers CD25 and CD69. *n* = 2 biological replicates. Symbols indicate individual human donors. Bars represent the mean. ** (**E**) Flow cytometric analysis of the percentage of cells expressing differentiation markers CCR7 and CD45RA. Naive = CCR7^+^ CD45RA^+^, CM = CCR7^+^ CD45RA^-^, EM = CCR7^-^ CD45RA^-^, TEMRA = CCR7^-^ CD45RA^+^. *n* = 2 biological replicates. Symbols indicate individual human donors. Bars represent the mean. **p < 0.01. ns = not significant. Source data are available online for this figure.

While treatment of T cells with the Ca_v_1 agonist FPL 64176 leads to short-lived effects, we wanted to generate T cells with permanently enhanced killing capacity. To do this, we used the PiggyBac transposon system to stably introduce either GFP alone (EV), human Ca_v_1.4 (WT) or Ca_v_1.4 harbouring a gain-of-function mutation (I745T) into human CD8^+^ CTLs (Fig. 10A). The I745T mutation was first identified in a New Zealand family and causes severe congenital stationary night blindness type-2 (Hope et al, 2005). Functionally, the mutation reduces both the energy required to open the channel as well as the inactivation kinetics (Hemara-Wahanui et al, 2005).

Human CD8^+^ CTLs expressing the PiggyBac constructs were sorted based on GFP expression (Fig. EV5D) and killing capacity was tested in vitro against P815 target cells. While CTLs expressing the WT Ca_v_1.4 did not show a significant difference in their killing capacity compared to EV control cells, expression of the I745T mutation led to a robust increase in target cell killing in all donors tested ranging from 10 to 280% (Fig. 10B). Comparisons between EV, WT, and I745T CTLs revealed no significant differences in differentiation characteristics. However, a trend towards higher proliferative capacity and activation in I745T CTLs was observed (Figs. 10C–E and EV5E).

## Discussion

In this manuscript we have uncovered a novel non-canonical Hh signalling pathway whereby a Ca_v_1 channel-mediated Ca^2+^ flux can induce Gli1. We show that the Ca_v_1-Gli1 axis is functionally important for CTL killing, providing a direct mechanistic link between Ca_v_1 channels and CTL killing.

There is currently great scientific interest to unmask the roles of Ca_v_1 channels in T cell biology. Constitutive knockout of Ca_v_1.4 or the Ca_v_ channel regulatory β3 subunit leads to signalling defects in thymocytes and apoptosis of peripheral T cells, respectively (Jha et al, 2009; Omilusik et al, 2011). Constitutive knockouts make it challenging to draw conclusions about the role of Ca_v_1 channels in mature peripheral T cells.

Early research suggested that all four family members of the L-type Ca^2+^ channels (Ca_v_1.1, Ca_v_1.2, Ca_v_1.3, and Ca_v_1.4) are expressed in murine T cells (Badou et al, 2013) but recently this was questioned by analysis of publicly available RNA-Seq and preliminary protein electrophoresis data (Erdogmus et al, 2022). Using validated qRT-PCR probes we demonstrate that apart from *Ca*_*v*_*1.1*, murine CTLs express all other alpha subunits of L-type Ca^2+^ channels. In addition, we find a similar expression pattern in human CD8^+^ T cells. We show that Ca_v_1.3 and Ca_v_1.4 are involved in the induction of Gli1 in CD8^+^ T cells. While it had been argued that Ca_v_1.4 resides on the plasma membrane little is known about the cellular localisation of Ca_v_ channels (Omilusik et al, 2011). Here we find that Ca_v_1.4 channels in primary human CD8^+^ T cells are localised on endosomes and reside at the plasma membrane in close proximity to the TCR complex. Upon TCR-induced immune synapse formation Ca_v_1.4 channels cluster at the center of the immune synapse in keeping with the hypothesis that Ca_v_1 channels might be directly regulated by the TCR upon stimulation.

Earlier work suggested that Ca_v_1 channels may contribute to TCR-induced Ca^2+^ signalling and effector function in T cells based on pharmacological inhibition with the pan-Ca_v_1 inhibitor nifedipine and a *Ca*_*v*_*1.4* constitutive KO model (Colucci et al, 2009; Kotturi et al, 2003; Omilusik et al, 2011). We have used both nifedipine and CRISPR-Cas9 editing of *Ca*_*v*_*1* cannels in primary CTLs to confirm the role of Ca_v_1 channels in TCR-mediated Gli1 induction and killing. Throughout, nifedipine showed a stronger effect compared to the double knockout of *Ca*_*v*_*1.3/1.4*. This is likely due to the following reasons. First, nifedipine blocks all Ca_v_1 family members, whereas our editing approach only targeted the two isoforms, which produced the strongest reduction of *Gli1* in our hands. Second, our editing may be incomplete, allowing residual protein expression. Third, we have shown that mature CTLs also express Ca_v_1.2, which raises the possibility of functional redundancy and compensation by Ca_v_1.2. Lastly, dihydropyridines such as nifedipine have been suggested to influence Ca^2+^ responses besides plasma membrane flux, such as store-dependent Ca^2+^ entry or intracellular Ca^2+^ handling (Kotturi et al, 2003).

In the literature links between Ca^2+^ and Hh signalling have been reported. In neuronal cells canonical Hh signalling can induce downstream Ca^2+^ spikes via Ca_v_ and TRPC1 channels (Belgacem and Borodinsky, 2011). Furthermore, Smo has been shown to reprogramme metabolism of adipocytes by inducing extracellular Ca^2+^ flux via Ca_v_1 channels (Teperino et al, 2012). Here we show the “reverse” in T cells, where Ca^2+^ signalling is capable of being an activator/inducer of Hh signalling. Our work uncovers Ca^2+^ signalling via Ca_v_1 channels as a previously unknown mechanism of non-canonical Hh signalling. The pathway operates independently of canonical signalling via Ptch/Smo in T cells but synergises with the canonical pathway to ensure optimal tumour cell killing.

The literature supports the notion that our proposed Ca_v_1-Gli axis may be active in other cells. Humans with mutations in Ca_v_1 channels phenotypically display cardiac arrhythmias and signs of autistic spectrum disorder (consistent with the critical roles of Ca_v_1 channels in the heart and brain) as well as signs of syndactyly (Splawski et al, 2004), a phenotype which has been robustly linked to inactivating mutations in the Hh pathway (Ahmed et al, 2017). Furthermore, studies in mice showed that knockdown of Ca_v_1 channels leads to abnormalities in skeletal development (Atsuta et al, 2019), highly reminiscent of the defects observed in *Ihh* KO mice (St-Jacques et al, 1999). These studies suggest that loss-of-function phenotypes of Ca_v_1 channels phenotypically mimic known Hh loss-of-function phenotypes, consistent with the hypothesis that a Ca_v_-induced mechanism of Gli activation is operational in other cell types.

Interestingly, we uncover that in CTLs MAPK activation downstream of the TCR contributes to Gli1 transcription which has been shown in other cell types and cancers (Rovida and Stecca, 2015). Of interest is the difference of Gli1 regulation between naive CD8^+^ T cells, that seem to mainly rely on Ca_v_1 signalling, and CTLs, that also employ MAPK signalling. A possible explanation for this could be that naive CD8^+^ T cells have much longer contact times with an APC and form more stable synapses in vivo when they are stimulated than CTLs, which have shorter contact times with their target cells and have been shown to often require multiple rounds of contact in vivo to efficiently kill target cells (Halle et al, 2016). Therefore, CTLs may have evolved two pathways to ensure appropriate levels of Gli1 activation.

Regarding the activation of Ca_v_1 channels in T cells, it is tempting to speculate that this is PKC-driven because of two observations. First, Ca_v_1 channels in T cells have been suggested to lack the ability to respond to depolarization and instead become activated upon TCR engagement (Badou et al, 2006) which is further supported by our data showing colocalisation between Ca_v_1.4 and the TCR on human CD8^+^ T cell and that Ca_v_ agonism depends on TCR engagement. And second, PKC is a well-known activator of Ca_v_1 channels in excitable cell types and has been hypothesized as the link between TCR and Ca_v_ channel activation in T cells (Robert et al, 2013; Strauss et al, 1997). This explains why we find that PMA (a PKC agonist) is able to drive Gli1 induction (Fig. 2B), particularly in naive CD8^+^ T cells where this effect cannot simply be explained by PKC-related induction of MAPK signalling (which has no effect on Gli1 induction). Notably, we show that PKC-induced Gli1 is abrogated when Ca_v_1 channels are inhibited and Ca_v_1 clusters at the immune synapse upon TCR activation supporting a critical role for PKC to activate Ca_v_1 channels in T cells. The requirement for PKC for Ca_v_1 activation and the different nature of the Ca^2+^ flux induced by ionomycin might also explain why ionomycin administration alone cannot induce Gli1.

An open question remains how a localised Ca^2+^ flux can induce Gli1. While Gli proteins lack canonical Ca^2+^-binding motifs, studies suggest that Ca^2+^ can modulate Gli activity indirectly via Ca^2+^-sensitive mediators of the Gli code. Examples include cAMP/PKA axis within the site of Gli processing in ciliated cells, modulating the balance of Gli activator and repressor forms (Shim et al, 2023). Additionally, Ca^2+^-dependent kinases like PKC may regulate Gli post-translational modifications, nuclear localisation, or stability (Montagnani and Stecca, 2019).

Taken together, we show that Gli1 is important for CD8^+^ T cell killing of tumour cells and that Gli1 activation is intricately coupled to CD8^+^ T cell activation downstream of the TCR via Ca_v_1 channels. Furthermore, we demonstrate that the Ca_v_1-Gli1 signalling arm is active not just in CD8^+^ T cells but in other cytotoxic lymphocytes including cytotoxic CD4^+^ T cells and γδ T cells. This is in keeping with clinical observations linking patient Ca_v_1 channel mutations to immunodeficiencies (Fenninger et al, 2019; Splawski et al, 2004). For example, patients with Timothy syndrome, who harbour a missense mutation in the *CACNA1S* gene, have dysfunctions in several organ systems including the immune system and nearly 50% of patients show recurrent infections (Splawski et al, 2004). And Ca_v_1.4-deficient patients with X-linked incomplete congenital stationary night blindness (harbouring a single point mutation R625X (p.Arg625Ter) leading to a premature stop codon in the *CACNA1F* gene) present with recurrent viral infections and pneumonia (Fenninger et al, 2019). On the other hand, nifedipine treatment has been shown to improve graft survival in renal transplantation patients (Shin et al, 1997) further underscoring the clinical importance of the Ca_v_1-Gli1-cytotoxicity axis.

The regulation of CTL killing is not only interesting from a cell biological aspect but is also critical for understanding and improving cellular CTL therapy such as CAR-T cell therapy in the clinic. Our work advances the field 2-fold. First, Gli inhibitor Arsenic Trioxide (ATO) is currently in clinical trials for the treatment of various cancers including advanced Basal Cell Carcinoma and Acute Myeloid Leukemia (Infante et al, 2015). Our data suggests that ATO may inhibit the CD8^+^ anti-tumour response and might explain why Gli1 inhibitors have had little success in the clinic so far. Second, our observation that Ca_v_1 agonists can enhance CTL killing provide a therapeutic entry point to either use Ca_v_1 agonists or Ca_v_1 activating mutations as another way to potentially amplify killing potential of CTLs in adoptive T cell therapy. Ca^2+^ signalling culminating in NFAT can lead to T cell exhaustion (Martinez et al, 2015). By contrast the unique, pharmacologically tractable Ca^2+^ flux via Ca_v_1 channels described here culminates in Gli1 upregulation and enhances killing potential without affecting characteristics associated with differentiation and proliferation.

## Methods

**Table:** Reagents and tools table

Reagent/Resource	Reference or Source	Identifier or Catalog Number
**Experimental models**		
C57BL/6J mice	Charles River Inc., UK	RRID:IMSR_JAX:000664
*OT-I* mice	The Jackson Laboratory, USA	RRID:IMSR_JAX:003831
*OT-II* mice	Charles River Inc., UK	RRID:IMSR_JAX:004194
*Rag2* KO mice	Gift from Dr. Suzanne Turner	RRID:IMSR_JAX:008449
*Rag2* KO *OT-I* mice	This study	
*Rag1 * KO *OT-I* mice	Gift from Dr. Gillian Griffiths (University of Cambridge)	
*Gli1-eGFP* mice	Gift from Dr. Alexandra Joyner (Brownell et al, 2011)	
*Gli1-eGFP* *OT-I* mice	This study	
*dLckCre* mice	Gift from Dr. Randall Johnson (University of Cambridge)	
*ROSA26tdTom* mice	Gift from Dr. Douglas Winton (University of Cambridge)	
GzmBER^T2^*Cre/ROSA26EYFP*	Gift from Dr. Douglas Thomas Fearon (Cold Spring Harbor Laboratory) (Bannard et al, 2009)	
*Smo*^*fl/fl*^ mice	The Jackson Laboratory, USA	RRID:IMSR_JAX:004526
*Ihh*^*fl/+*^ mice	The Jackson Laboratory, USA	RRID:IMSR_JAX:024327
*GzmBER* ^*T2*^ *Cre/ROSAEYFP/Smo* ^*fl/fl*^	This study	
*dLckCre/ROSA26tdTom/Ihh* ^*fl/f*^	This study	
Human PBMCs	NHS Blood & Transplant	
HEK293T cell line	ATCC	CRL-3216
MC38 cell line	Kerafast	ENH204-FP
MC38-OvaT4 cell line	This study	Derived from MC38 by retroviral transduction
MC38-OvaN4 cell line	This study	Derived from MC38 by retroviral transduction
P815 cell line	ATCC	TIB-64
EL4 cell line	ATCC	TIB-39
EL4-OvaN4 cell line	This study	Derived from EL4 by retroviral transduction
K562 cell line	ATCC	CCL-243
RAJI cell line	ATCC	CCL-86
**Recombinant DNA**		
pMig-OvaT4	Gift from Dr. Dietmar Zehn (Technical University of Munich) (Zehn et al, 2009)	
pMig-OvaN4	Gift from Dr. Dietmar Zehn (Technical University of Munich) (Zehn et al, 2009)	
pCL-Eco: retroviral packaging plasmid	Gift from Dr. Gillian Griffiths (University of Cambridge)	
pMig-eGFP-Thy1.1	This study	
pMig-SmoeGFP-Thy1.1	This study	
pMig-SmoM2eGFP-Thy1.1	This study	
pMig-Ihh-Thy1.1	This study	
pcDNA3.1 +/Ca_v_1.4-C-(K)-DYK	Genscript	OHu21305
pSpCas9(BB)-T2A-GFP	Addgene	48138, RRID:Addgene_48138
pPBCAG-rtTAM2-IH	Gift from Dr. Marc de la Roche (University of Cambridge)	
pB-GFP-IRES-Hygro	This study	
pB-CACNA1F(WT)-T2A-GFP-IRES-Hygro	This study	
pB-CACNA1F(I745T)-T2A-GFP-IRES-Hygro	This study	
pHR-SIN/pHR-mScarlet backbone	Gift from Dr. Marc de la Roche (University of Cambridge)	
pHR-CACNA1F(WT)-HA-T2A-mScarlet	This study	
GFP-VIVIT plasmid	Addgene (gift from Dr. Anjana Rao)	11106; RRID: Addgene_11106
IκBα dominant-negative plasmid	Gift from Dr. Felix Randow (MRC Laboratory of Molecular Biology, Cambridge)	
**Antibodies**		
anti-CD3ε (murine)	eBioscience	16-0033-86
anti-CD28 (murine)	eBioscience	16-0281-86
anti-CD3ε (human, clone UCHT1)	BioLegend	300438
anti-CD3ε (human, clone OKT3)	BioLegend	317326
goat anti-hamster IgG	Thermo Fisher	31115
goat anti-mouse IgG	Thermo Fisher	31160
MHC Class II blocking antibody (clone M5/114.15.2)	BioLegend	107656
TruStain FcX Fc block	BioLegend	101320
Human TruStain FcX Fc block	BioLegend	422302
Antibodies used for flow cytometry	Various manufacturers	See Appendix Table S3
Antibodies used for immunofluorescence staining	Various manufacturers	See Appendix Table S6
Antibodies used for western blotting	Various manufacturers	See Appendix Table S7
**Oligonucleotides and other sequence-based reagents**		
Alt-R tracrRNA	Integrated DNA Technologies	1072534
Alt-R CRISPR-Cas9 crRNAs	Integrated DNA Technologies	See Appendix Table S1
Primers for genome editing assay	Sigma	See Appendix Table S2
TaqMan probes	Thermo Fisher	See Appendix Table S5
I745T forward mutagenesis primer	This study	TTGTCCACAGCAGTGGCAAGAAACACGTTCAACAGG
I745T reverse mutagenesis primer	This study	CCTGTTGAACGTGTTTCTTGCCACTGCTGTGGACAA
**Chemicals, Enzymes and other reagents**		
RPMI 1640	Gibco	21875034
IMDM	Gibco	12440061
ImmunoCult Cell Culture Medium	STEMCELL Technologies	10981
CTS OpTmizer T Cell Expansion SFM	Gibco	A1048501
DMEM	Gibco	21885-025
RPMI without phenol red	Gibco	11835-063
Opti-MEM	Gibco	31985070
Heat-inactivated batch-tested FCS	Biosera	1001-500 ml
β-mercaptoethanol	Gibco	31350-010
Penicillin/Streptomycin	Gibco	15140-122
Sodium pyruvate	Gibco	11360039
HEPES	Sigma	H0887
MEM Non-Essential Amino Acid Solution	Gibco	11140-035
Human plasma	Life Science Production	P-105ACT-US
L-glutamine	Gibco	25030024
Murine IL-2	PeproTech	212-12
Human IL-2	PeproTech	200-02-100
Human IL-2	Miltenyi Biotec	130-097-746
Human IL-15	Miltenyi Biotec	130-095-765
Human IL-4	PeproTech	200-04
Human IFN-γ	PeproTech	300-02
Human IL-21	PeproTech	200-21
Human IL-1β	PeproTech	200-01B
Human T-Activator CD3/CD28 Dynabeads	Thermo Fisher	111.31D
ImmunoCult Human CD3/CD28/CD2 T Cell Activator	STEMCELL Technologies	10970
Ova_257-264_ peptide	AnaSpec	60193-5-ANA
Ova_323-339_ peptide	AnaSpec	AS-27024
NEB 5-alpha competent *E. coli*	New England Biolabs	C2987H
Q5 High-Fidelity DNA Polymerase	New England Biolabs	M0491L
Gibson Assembly Master Mix	New England Biolabs	E2611
T4 DNA Ligase	New England Biolabs	M0202S
Restriction enzyme XhoI	New England Biolabs	R0146S
Restriction enzyme NotI	New England Biolabs	R3189S
Restriction enzyme MluI	Thermo Fisher	FD0564
Restriction enzyme NotI	Thermo Fisher	FD0596
CFSE	Invitrogen/Thermo Fisher	C34554
CellTracker Violet BMQC dye	Thermo Fisher	C10094
eFluor780 viability dye	eBioscience	65-0865-18
eBioscience Calcium Sensor Dye eFluor 514	Thermo Fisher	65-0859-70
DAPI	Thermo Fisher	D9542
Apotracker Green	BioLegend	427403
Hoechst 33342	Invitrogen/Fisher	H3570
Lipofectamine 2000	Invitrogen	11668019
Zoledronate	Sigma	SML0223
LPS	Sigma	L4391
4-hydroxytamoxifen (4-OHT)	Tocris	3412
Small molecule inhibitors used in study	Various manufacturers	See Appendix Table S4
DMSO	Life Technologies	D12345
Bovine serum albumin (BSA)	Sigma	A3912
Paraformaldehyde (16% solution)	CN Technical Services/EMS	15710-S
0.45 µm PVDF membrane filter	Merck Millipore	SLHVM33RS
Protamine sulphate	Sigma	1101230005
Protease inhibitor	Pierce	88666
Protein standard	Bio-Rad	161-0394
NuPAGE 4–12% Bis/Tris gel	Thermo Fisher	NP0335BOX
NuPAGE MOPS running buffer	Thermo Fisher	NP0001
NuPAGE transfer buffer	Thermo Fisher	NP0006-01
Methanol	Honeywell	32213-2.5L
SuperSignal West Pico Plus substrate	Thermo Fisher	34580
ProLong Diamond Antifade Mountant	Fisher	P36961
Triton X-100	Alfa Aesar	A16046.AE
**Software**		
FlowJo	BD/Tree Star	v10.4
GraphPad Prism	GraphPad	v7
Imaris	Bitplane/Oxford Instruments	v10.2
ImageQuant TL	Cytiva	v10.2
IDEAS	Millipore Sigma	
Incucyte software	Sartorius	v2022B
Salmon	(Patro et al, 2017)	v1.9.0
DESeq2	(Love et al, 2014)	v1.26.0
GSEA	Broad Institute (Subramanian et al, 2005)	v3
IGV	Broad Institute (Robinson et al, 2011)	
**Other**		
Murine naive CD8+ T Cell Isolation Kit	Miltenyi Biotec	130-096-543
Murine naive CD4+ T Cell Isolation Kit	Miltenyi Biotec	130-104-453
MojoSort Human CD8+ Naive T Cell Isolation Kit	BioLegend	480045
HumanCD8+ T Cell Isolation Kit	Miltenyi Biotec	130-096-495
EasySep Human TCR Alpha/Beta Depletion Kit	STEMCELL Technologies	17847
EasySep Biotin Positive Selection Kit	STEMCELL Technologies	17683
DNeasy Blood & Tissue Kit	Qiagen	69506
SequalPrep Long PCR Kit with dNTPs	Invitrogen	A10498
Alt-R Genome Editing Detection Kit	Integrated DNA Technologies	1075932
EasySep PE Positive Selection Kit	STEMCELL Technologies	17684
EasySep Release Mouse PE Positive Selection Kit	STEMCELL Technologies	17656
Amaxa Mouse T Cell Nucleofector Kit	Lonza	VVPA-1006
CytoTox 96 Non-radioactive Cytotoxicity Assay	Promega	G1780
RNAqueous-Micro Total RNA Isolation Kit	Thermo Fisher	Catalog number not specified
Single Cell RNA Purification Kit	Norgen Biotek	51800
Maxwell RSC simplyRNA Cells Kit	Promega	AS1390
PureLink RNA Mini Kit	Thermo Fisher	12183025
One-Step qRT-PCR Kit (SuperScript III Platinum)	Thermo Fisher	11732088
Qubit RNA HS Assay Kit	Thermo Fisher	Q32852
High Sensitivity RNA ScreenTape	Agilent	5067-5579
RNA ScreenTape	Agilent	5067-5576
D5000 ScreenTape	Agilent	5067-5588/5589
Illumina Stranded mRNA Prep	Illumina	1000000124518 v02
QIAprep Spin Miniprep Kit	Qiagen	27104
HiSpeed Plasmid Maxi Kit	Qiagen	12662
QuikChange II XL Site-Directed Mutagenesis Kit	Agilent	200522
Neon Transfection System 100 µL tips	Thermo Fisher	MPK10096
SepMate PBMC Isolation Tubes	STEMCELL Technologies	86450
BD Cytofix/Cytoperm Plus Fixation Buffer	BD Biosciences	554715
BD Phosflow Lyse/Fix Buffer	BD Biosciences	558049
BD Phosflow Perm/Wash I Buffer	BD Biosciences	557885
EasyEights magnet	STEMCELL Technologies	18103
Lympholyte	Cedarlane	CL5035
4200 TapeStation	Agilent	
CLARIOstar microplate reader	BMG Labtech	
Incucyte S3 System	Sartorius	
BD LSRII flow cytometer	BD Biosciences	
BD LSR Fortessa flow cytometer	BD Biosciences	
BD FACSymphony A5 cell analyser	BD Biosciences	
BD FACSAria IIu cell sorter	BD Biosciences	
Neon electroporation system	Thermo Fisher	
Nucleofector 2b Device	Lonza	Program X-001
Nanodrop spectrophotometer	Labtech	ND-1000
QuantStudio 6 Flex Real-Time PCR System	Thermo Fisher	
Andor Dragonfly 500 spinning-disc confocal microscope	Oxford Instruments	
Amnis ImageStream imaging cytometer	Millipore Sigma	
NovaSeq sequencer	Illumina	paired-end 50 bp sequencing

### Mice

*Rag2* KO were a generous gift from Suzanne Turner (University of Cambridge) and *OT-I* mice were purchased from the Jackson Laboratory (C57BL/6-Tg(TcraTcrb)1100Mjb/j, Stock no. 003831). *OT-I Rag2* KO mice were generated from these. *OT-I Rag1KO* mice were a generous gift from Gillian Griffiths (University of Cambridge). *OT-II* mice were purchased from Charles River Inc., UK. *Gli1-eGFP* mice were a generous gift from Alexandra Joyner (Sloan Kettering Institute) (Brownell et al, 2011) and were backcrossed onto the C57BL/6J background (The Jackson Laboratory, Bar Harbor, ME) for more than 11 generations. *Gli1-eGFP* *OT-I* mice were generated from these. *dLckCre* and *ROSA26tdTom* mice were a generous gift from Randall Johnson and Douglas Winton (University of Cambridge), respectively. *GzmBER*^*T2*^*Cre/ROSA26EYFP* mice were a generous gift from D. T. Fearon (Cold Spring Harbor Laboratory) (Bannard et al, 2009) and were backcrossed onto the C57BL/6J background (The Jackson Laboratory, Bar Harbor, ME) for more than 11 generations. *Smo*^*f/f*^ (Smo^tm2Amc^/J, Stock no. 004526*)* and *Ihh*^*f/+*^ (*Ihh*^*tm1Blan*^/J, Stock no. 024327) mice were purchased from The Jackson Laboratory. *Smo*^*fl/fl*^ mice were back-crossed to the C57BL/6 J background (Charles River Inc., UK) for more than 10 generations and crossed to GzmBER^T2^*Cre/ROSA26EYFP* mice to generate *GzmBER*^*T2*^*Cre/ROSAEYFP/Smo*^*fl/fl*^ mice. *Ihh*^*fl/fl*^ mice were crossed onto *dLckCre/ROSA26tdTom* mice to generate *dLckCre/ROSA26tdTom/Ihh*^*fl/fl*^ mice.

Mice were maintained at the CRUK Cambridge Institute/University of Cambridge, genotyped using Transnetyx, and used at 6–8 weeks of age. All housing and procedures were performed in strict accordance with the United Kingdom Home Office Regulations.

### In vivo mouse experiments

*Rag2* KO mice were injected subcutaneously with 100 µl of 0.5 × 10^6^ MC38-OvaT4 cell suspension in PBS in the flank. For injection of CTLs, naive murine CD8^+^ T cells were isolated from spleen and peripheral lymph nodes of donor *Gli1-eGFP OT-I* mice using MACS isolation. Cells were stimulated with plate-bound anti-CD3ε (1 µg/ml) and anti-CD28 (2 µg/ml) for 48 h and media was changed every day thereafter. To minimise allocation bias, on day 13 post tumour cell injection, mice were randomly assigned to two groups with comparable mean tumour size. Then, recipient mice received 4 × 10^6^ CTLs, either wild type or knockout for *Gli1*, 7–8 days post activation intravenously via tail vein injection. Mice were weighed and tumours measured at least three times per week and harvested on day 27 post injection.

For in vivo killing assays, untransduced EL4 cells (EL4-UT) were labelled with 2 µM CellTracker^TM^ Violet BMQC dye (Thermo Fisher, Cat no. C10094) in serum-free RPMI media for 30 min at 37 °C in a humidified incubator. EL4-UT and GFP-expressing EL4-OvaN4 were washed, resuspended in ice-cold PBS at 32 × 10^6^/mL and mixed at a ratio of 1:1. *RAG2* KO mice were injected intraperitoneally with 100 µL of EL4-OvaN4/EL4-UT mixture. 2 h post-injection of target cells, mice received 0.5 × 10^6^ of CTLs, either NTC or *Ca*_*v*_*1.3/1.4* KO via intraperitoneal injection. Mice were sacrificed 20 h post-CTL injection and the cellular content of their intraperitoneal cavity was collected by injecting 5 mL of ice-cold PBS and gently massaging the area followed by collection of the peritoneal fluids. Cells were then stained with a viability dye eFluor780 and subjected to flow cytometric analysis. The ratio of EL4-OvaN4 to EL4-UT was calculated by dividing the percentages of GFP^+^ (EL4-OvaN4) with CellTracker^TM^ Violet BMQC^+^ (EL4-UT) cells. This was then normalised to the ratio observed in PBS only (no effector) control mice.

### Cell culture

Purified naive murine CD8^+^ T cells were cultured at 1 × 10^6^ cells/ml in complete RPMI media: RPMI (Gibco, Cat no. 21875034) supplemented with 10% heat-inactivated batch-tested FCS (Biosera, Cat no. 1001-500ml), 50 µM β-Mercaptoethanol (50 mM, Gibco, Cat no. 31350-010), 100 U/ml Penicillin/Streptomycin (10,000 U/ml, Gibco, Cat no. 15140-122), 1 µM Sodium Pyruvate (Gibco, Cat no. 11360039), 10 µM HEPES (Sigma, Cat no. H0887) and 10 ng/ml murine IL-2 (Peprotech, Cat no. 212-12).

Murine CD4^+^ T cells were maintained at 1 × 10^6^/ml in complete IMDM media: (Gibco, Cat no. 12440061) supplemented with 5% heat-inactivated batch-tested FCS, 10 µM β-Mercaptoethanol, 100 U/ml Penicillin/Streptomycin and 40 ng/ml human IL-2 (Peprotech, Cat no. 200-02-100).

Human CD8^+^ T cells were cultured at 0.25–0.4 × 10^6^ cells/ml in ImmunoCult cell culture media (Stemcell Technologies, Cat no. 10981) supplemented with 100 U/ml Penicillin/Streptomycin and 100 IU/ml human IL-2 (Miltenyi, Cat no. 130-097-746).

Human Vγ9Vδ2^+^ T cells were cultured in complete Vγ9Vδ2^+^ T cell media: RPMI supplemented with 10% heat-inactivated batch-tested FCS, 1 µM Sodium Pyruvate, 20 µM HEPES, 1x MEM Non-Essential Amino Acid Solution (Gibco, Cat no. 11140-035), 100 U/ml Penicillin/Streptomycin, 100 IU/mL human IL-2 and 120 IU/mL human IL-15 (Miltenyi, Cat no. 130-095-765).

Human Vδ1 + T cells were cultured in complete Vδ1 + T cell media: CTS™ OpTmizer™ T Cell Expansion SFM (Gibco, Cat no. A1048501) supplemented with 2.5% human plasma (Life Science Production, Cat no. P-105ACT-US), 2 mM L-glutamine (Gibco, Cat no. 25030024) and 100 U/ml Penicillin/Streptomycin.

HEK 293T, MC38-OvaT4 and N4 as well as P815 cells were cultured in DMEM (Gibco, Cat no. 21885-025) supplemented with 10% heat-inactivated FCS. EL-4, K562, and RAJI cells were cultured in RPMI supplemented with 10% heat-inactivated FCS.

All cells were grown in a humidified incubator at 37 °C and 5% CO_2_ and cell lines were STR profiled and tested mycoplasma negative (MycoProbe^®^ Mycoplasma Detection Kit, R&D systems).

### T cell isolation and activation

#### Mouse

Spleens were harvested and cell suspensions made. Naive murine CD8^+^ T cells were isolated using negative selection (Naive CD8^+^ T Cell Isolation Kit, Cat no. 130-096-543, Miltenyi Biotec) according to the manufacturer’s instructions. The purity of the sorted populations was above 95%. CD8^+^ T cells were stimulated with plate-bound anti-CD3ε (1 µg/ml, eBioscience, Cat no. 16-0033-86) and anti-CD28 (2 µg/ml, eBioscience, Cat no. 16-0281-86) antibody.

For CTL generation, a splenocyte suspension from * Rag1 * or *Rag2* KO *OT-I*  mice was stimulated with 10 nM Ova_257-264_ peptide (SIINFEKL, AnaSpec, Cat no. 60193-5-ANA) or with plate-bound anti-CD3ε (1 µg/ml) and anti-CD28 (2 µg/ml) for 48 h and cultured for up to 10 days. Cells were restimulated with plate-bound anti-CD3ε (2.5 μg/ml) at day 9/10 post stimulation.

For cytotoxic CD4^+^ T cells, a single cell suspension was generated from an *OT-II* C57B6/J spleen. Cells were cultured at 2 × 10^6^/ml in complete IMDM in the presence of 4 ng/ml murine IL-12, 40 ng/ml human IL-2 and stimulated with soluble 1 µg/ml Ova_323-339_ (Anaspec, Cat no. AS-27024). After 48 h of stimulation the cells were washed and maintained in complete IMDM + 40 ng/ml human IL-2. On day 3 post-stimulation, cells were purified on a lympholyte gradient as previously described. B cells and CD8 + T cells were subsequently depleted by incubating the cell suspension with 1:800 biotinylated anti-CD8 and 1:800 biotinylated anti-CD19 antibodies for 10 min. Contaminating CD8 + T cell and B cell fractions were depleted using the EasySep™ Biotin Positive Selection Kit (StemCell Technologies, Cat no. 17683) according to the manufacturer’s instructions with one round of selection, collecting only the non-bound CD19- CD8- fraction. Purity was assessed by flow cytometry and was regularly over 98%. Cells were maintained at 1 × 10^6^/ml in complete IMDM + 40 ng/ml human IL-2. Alternatively, naive murine CD4^+^ T cells were isolated using negative selection (Naive CD4^+^ T Cell Isolation Kit, Cat no. 130-104-453, Miltenyi Biotec) according to the manufacturer’s instructions. The purity of the sorted populations was above 95%. CD4^+^ T cells were stimulated with plate-bound anti-CD3ε (1 µg/ml) and anti-CD28 (2 µg/ml) for 48 h and kept thereafter at 1 × 10^6^/ml in complete IMDM + 40 ng/ml human IL-2.

#### Human

PBMCs were isolated from Buffy Coats or Leukopaks of healthy donors using SepMate PBMC Isolation Tubes (Stemcell Technologies, Cat no. 86450) according to the manufacturer’s instructions.

Naive CD8^+^ cells were obtained using the MojoSort Human CD8^+^ Naive T Cell Isolation Kit (Biolegend, Cat no. 480045). Naive CD8^+^ cells were plated with Human T-Activator CD3/CD28 Dynabeads (Thermofisher, Cat no. 111.31D) at 25 µl of Dynabeads per 1 × 10^6^ CD8^+^ cells in complete ImmunoCult cell culture media (see above). On day 3, the beads were magnetically removed. When indicated, the cells were re-stimulated on d10 with 12.5 µl of Dynabeads per 1 × 10^6^ CD8^+^ cells for three additional days. On d14, the beads were removed.

Alternatively, CD8^+^ T cells were isolated using negative selection (CD8^+^ T Cell Isolation Kit, Cat no. 130-096-495, Miltenyi Biotec) and were stimulated with ImmunoCult™ Human CD3/CD28/CD2 T Cell Activator (Stemcell Technologies, Cat no. 10970) at 25 μl/mL of CD8^+^ cell suspension for 72 h. When indicated, the cells were re-stimulated between day 11–20 with either 1 µg/mL anti-CD3ε antibody (clone UCHT1, Biolegend, Cat no. 300438) or with 12.5 μl/mL of ImmunoCult™ Human CD3/CD28/CD2 T Cell Activator, respectively, in the presence of the indicated concentrations of FPL 64176 or carrier control.

Vγ9Vδ2^+^ T cells were expanded from PBMCs following a modified expansion protocol (Peters et al, 2020). Briefly, PBMCs were depleted of αβ T cells using the EasySep^TM^ Human TCR Alpha/Beta Depletion Kit (Stemcell Technologies, Cat no. 17847) followed by plating at 1 × 10^6^/ml with 1 µM zoledronate (Sigma, Cat no. SML0223), human IL-2 and IL-15 in 6-well plates with 5 ml cell suspension per well. Cells were supplemented with human IL-2 and IL-15 every 2–3 days to a final concentration of 100 IU/mL and 120 IU/mL, respectively, during the whole course of the expansion period. Cells were split on day 6–8 and day 14–16 by collecting the cells, adding 0.5 × volume of fresh cell culture medium, supplementing with human IL-2 and IL-15 to a final concentration of 100 IU/mL and 120 IU/mL, respectively, and re-seeding the cells at 1 × 10^6^/ml in 6-well plates with 5 ml cell suspension per well. Cells were maintained until day 24 of culture. Purity was assessed by flow cytometry on day 14 and was regularly over 90%.

Vδ1^+^ T cells were expanded from PBMCs following an optimised expansion protocol (Sanchez Martinez et al, 2022). Briefly, PBMCs were depleted of αβ T cells using the EasySep^TM^ Human TCR Alpha/Beta Depletion Kit followed by plating at 1 × 10^6^/ml with 140 ng/ml soluble anti-CD3ε antibody (clone OKT3, Biolegend, Cat no. 317326), 100 ng/ml human IL-4 (Peprotech, Cat no. 200-04), 70 ng/ml human IFN-γ (Peprotech, Cat no. 300-02), 7 ng/ml human IL-21 (Peprotech, Cat no. 200-21) and 15 ng/ml human IL-1β (Peprotech, Cat no. 200-01B) in 6-well plates with 5 ml cell suspension per well. At day 7, cell cultures were supplemented with 1 µg/mL soluble anti-CD3ε antibody, 13 ng/ml human IL-21 and 100 IU/ml human IL-15. Cells were split on day 11 by collecting the cells, adding 0.5 × volume of fresh cell culture medium supplemented with 1 µg/ml soluble anti-CD3ε antibody and 100 IU/mL human IL-15 and re-seeding the cells at 1 × 10^6^/ml in 6-well plates with 5 ml cell suspension per well. On day 18, cell cultures were collected and stained with 1:100 anti-Vδ1 APC antibodies for 30 min at 4 °C and Vδ1^+^ T cells were enriched by positive selection with anti-APC microbeads (Miltenyi Biotec, Cat no. 130-090-855) according to the manufacturer’s instructions. Purity was assessed immediately by flow cytometry and was regularly over 99%. Cells were maintained until day 24 of culture.

### CRISPR knockout of primary CD8^+^ T cells

Ribonucleoprotein (RNP) complexes were assembled following the manufacturer’s instructions using Alt-R^®^ CRISPR-Cas9 crRNA (IDT, Appendix Table S1) and tracrRNA (IDT, Cat no. 1072534). CD8^+^ T cells were isolated from * Rag2* KO *OT-I* spleens and stimulated with plate-bound anti-CD3ε/CD28 antibodies in complete RPMI media. After 48–72 h of stimulation, cells were washed twice in pre-warmed PBS (Gibco) prior to resuspension in 80 µl Buffer T (10^6^ cells/electroporation reaction). The suspension was briefly mixed with the RNP complex solution prior to electroporation with the Neon™ electroporation system in 100 µl electroporation tips (Thermo, Cat no. MPK10096) with three pulses of 1600 V each with a pulse width of 10 ms. Cells were left to recover in complete RPMI in the absence of antibiotics for 20 min. Cells were then centrifuged at 480 × *g* for 5 min and returned into culture in complete RPMI media (without antibiotics). Antibiotics were re-added after six hours at the indicated concentration.

### Genome editing efficiency assay

Genomic DNA was extracted from T cells using a DNeasy Blood & Tissue kit (Qiagen, Cat no. 69506). PCR amplification of the region of gDNA containing the CRISPR editing locus was performed using the SequalPrep Long PCR Kit with dNTPs (Invitrogen, Cat no. A10498) according to the manufacturer’s instructions. Primers (Sigma) used are detailed in Appendix Table S2. A T7 endonuclease I mismatch cleavage assay was performed using the Alt-R® Genome Editing Detection Kit (IDT, Cat no. 1075932) per the manufacturer’s instructions. Quantification of cleaved bands was performed on a capillary electrophoresis system (4200 Tapestation, Agilent) using D5000 screentape (Agilent, Cat no. 5067-5588/9).

### In vitro killing assay

#### LDH-based killing assays

CD8^+^ T cell cytotoxicity was assessed in murine, human CTLs that were generated as previously described and used on day 6/7 or day 12–29 post-stimulation, respectively. The CytoTox 96^®^ Non-radioactive Cytotoxicity Assay kit (Promega, Cat no. G1780) was used according to the manufacturer’s instructions. EL4 cells were used as target cells for murine *OT-I *CTLs and pulsed for 1 h at 37 °C with 1 µM SIINFEKL while P815 cells were used as target cells for human CTLs and pulsed for 1 h at 37 °C with 0.5–1 µg/ml anti-CD3 antibody (clone UCHT1, Biolegend, Cat no. 300438) after which they were washed and resuspended at 1 × 10^5^ cells/ml in killing assay media (RPMI without phenol red, Gibco, Cat no. 11835-063, 2% FCS) in a round-bottom 96-well plate. T cells were washed, resuspended in killing assay media (RPMI without phenol red + 2% FCS) and plated at the indicated effector:target ratios. Plates were incubated at 37 °C prior to collection of supernatant at the designated timepoints. Absorbance was measured at 490 nm using a CLARIOstar microplate reader (BMG Labtech).

Human Vγ9V2δ^+^ T cell cytotoxicity against K562 and Raji targets were determined using CytoTox 96^®^ Non-radioactive Cytotoxicity Assay kit according to the manufacturer’s instructions. K562 and Raji cells were incubated with 50 µM zoledronate for 18 h before the assay after which they were washed and resuspended at 1 × 10^5^ cells/ml in killing assay media in a round-bottom 96-well plate. Vγ9V2δ^+^ T cells were washed, resuspended in killing assay media (RPMI without phenol red + 2% FCS) and plated at the indicated effector:target ratios. Plates were incubated at 37 °C prior to collection of supernatant at the designated timepoints. Absorbance was measured at 490 nm using a CLARIOstar microplate reader (BMG Labtech). All killing assays contained “target cell only” controls (in the presence or absence of small molecule controls when applicable).

#### Flow cytometry-based killing assays

In addition, a flow cytometry-based killing assay was used to measure human CD8^+^ CTL, Vγ9Vδ2, Vδ1, and murine CD4^+^ CTL cytotoxicity when FPL-64176 was used. Here, P815 targets cells were labelled for 45 min at 37 °C with CFSE (1:30,000, Invitrogen, Cat no. C34-554). Following quenching and washing, target cells were resuspended at 2.5 × 10^5^ cells/ml in killing assay media and pulsed for 1 h at 37 °C with 0.05 µg/mL anti-CD3 antibody (clone UCHT1, Biolegend, Cat no. 300438) before plating in a round-bottom 96-well plate. CTLs were washed, resuspended in killing assay media, and plated at the indicated effector to target ratios. Plates were incubated at 37 °C for the indicated timepoints and samples were stained with fixable viability dye eFluor780 (eBioscience, Cat no. 65-0865-18) as previously described. Samples were fixed with 2% paraformaldehyde for 10 min at room temperature and washed before flow-cytometry analysis. Specific lysis was calculated by subtracting the percentage of dead CFSE^+^ target cells in control wells (target cells alone) from the percentage of dead CFSE^+^ target cells in test wells (target cells + effector cells).

On day 5 after cytotoxic *OT-II* CD4 + T cell stimulation, B cells were purified from the spleen of a C57B6/J mouse using MACS positive selection (Miltenyi, Cat no. 130-121-301) per the manufacturer’s instructions. Details of the MACS immune cell isolation are described above. Purity was routinely over 95%. Cells were cultured at 2 × 10^6^/ml for 48 h in complete RPMI in the presence of 25 µg/ml LPS (Sigma, Cat no. L4391).

The cytotoxic CD4^+^ killing assay was performed on day 7 post-stimulation. B cell targets were labelled with CFSE (Thermo, Cat no. C34554). Half of the B cells were labelled with low-dose 0.25 µM CFSE and the other half with high-dose 1.25 µM CFSE per the manufacturer’s instructions. Cells were resuspended at 5 × 10^6^/ml and the B cells labelled with high-dose CFSE were pulsed with 10 µM Ova_323-339_ for 1 h at 37 °C. During this time T cells were washed, resuspended at 1 × 10^6^/ml pre-incubated with the indicated concentration of small-molecule inhibitor at 37 °C. After pulsing, B cells were washed 3 times, counted and mixed in a 1:1 ratio (high-dose CFSE + Ova pulsed targets: low dose CFSE + not pulsed). 20,000 B cells were plated per well of a 96-well round-bottom plate. Effector T cells were plated at the indicated effector:target ratios including a target only condition. Drugs were supplemented as indicated and the plate was left to incubate for 14 h in a humidified incubator at 37 °C. After this, cells were stained with eFluor780 and acquired on a flow cytometer. Cytotoxicity was calculated using the following formula:

$${{{\mathrm{Cytotoxicity}}}}=100 \, x \, (1-\frac{\frac{{Unloaded}}{{Loaded}}{no}\,T\,{cells}}{\frac{{Unloaded}}{{Loaded}}{Experimental}})$$

An MHC Class II blocking antibody (clone: M5/114.15.2, Biolegend Cat. No. 107656) was included as a negative control in some experiments. In these conditions, 20 µg/ml MHC-Class II blocking antibody was added to the cells prior to the 14 h incubation. All killing assays contained “target cell only” controls (in the presence or absence of small molecule controls when applicable).

#### Incucyte killing assays

Incucyte killing assay with NTC *Ca*_*v*_*1.3/1.4* double KO CD8^+^ CTLs were performed on an Incucyte S3 System (Sartorius) on day 6 post-stimulation. Briefly, 8000 MC38-OvaN4 tumour cells were plated on 96-well flat bottom plates overnight. The next morning, NTC or *Ca*_*v*_*1.3/1.4* double KO CTLs were washed and resuspended in RPMI1640 media + 10% FCS and 4000 cells were plated out and imaged immediately in the presence of 1:800 Apotracker^TM^ Green (Biolegend, Cat no. 427403). Plates were scanned in brightfield and green channels every 30 min and analysed using the Incucyte v2022B software (Sartorius) to analyse green fluorescence (green total integrated intensity—green TII). Cell death was calculated by normalising green TII to the 45 h time point in the NTC + target condition.

### Flow cytometry

#### Surface staining

Cells were stained with fixable viability dye eFluor780 (eBioscience, Cat no. 65-0865-18) or DAPI (Thermo Fisher, Cat no. D9542, 1:3000 in PBS), washed, and incubated with Fc block (1:100; Biolegend TruStain fcX, Cat no. 101320) and fluorophore-conjugated antibodies at the appropriate concentrations (Appendix Table S3) for 20 min at 4 °C.

#### Intracellular staining

Following surface staining, cells were fixed with BD Cytofix/Cytoperm Plus Fixation Buffer (BD Biosciences, Cat no. 554715) as per manufacturer’s instructions and stained with fluorophore-conjugated antibodies at the appropriate concentrations (Appendix Table S3) for 30 min at 4 °C. Prior to analysis, cells were washed once in permeabilization buffer and once in FACS buffer.

#### pErk staining

Naive CD8^+^ T cells or CTLs were isolated/maintained as described previously. Cells were pre-incubated for 30 min at 37 °C with 10 µM MEK1/2 inhibitor U0126 (Cambridge Biosciences, Cat no. SM106-5) or carrier control (DMSO). Cells were stimulated by cross-linking the TCR using 10 µg/ml soluble anti-CD3ε and 5 µg/ml soluble anti-CD28 with the addition of goat-anti-hamster IgG (1:300) for 2 min in a 37 °C waterbath. Cells were fixed in 2% PFA (EMS, Cat no. 15710-S), washed and incubated in BD Phosflow Lyse/Fix Buffer (BD, Cat no. 558049) for 10 min at 37 °C. Cells were washed with BD Phosflow Perm/Wash I Buffer (BD, Cat no. 557885) and stained with primary anti-pErk antibody (30 min, 4 °C) and then secondary anti-Rabbit Alexa Fluor 647 and anti-CD8a PE antibodies at the indicated concentrations (Appendix Table S3) (20 min, 4 °C).

#### Ca^2+^ flux assays

Intracellular calcium flux was measured in murine and human CTLs on day 6–8 and 10–16 of culture, respectively. Cells were resuspended in phenol-red free RPMI media containing 2% HI-FCS and 20 mM HEPES and loaded with 3 µM eBioscienceTM Calcium Sensor Dye eFluorTM 514 (Thermo Fisher, Cat no. 65-0859-70) for 30 min in the incubator at 37 °C. In the case of murine CTLs, for the last 15 min, 1 µg/ml anti-CD3ε antibody (clone 145-2C11, Biolegend, Cat no. 100340) and 1:200 anti-CD8a BUV737 (Appendix Table S3) antibody were added together with the indicated drugs or carrier controls. In the case of human CTLs, for the last 15 min, 1 µg/ml anti-CD3ε antibody (clone UCHT1, Biolegend, Cat no. 300438) and 1:200 anti-CD8a BUV737 (Appendix Table S3) antibody were added together with the indicated drugs or carrier controls. Afterwards cells were kept on ice prior to flow cytometric analysis. Each sample was equilibrated at 37 °C for 10 min and then kept at room temperature for 2 min before running on the flow cytometer. First, 30 s of baseline was recorded before calcium flux was induced by cross-linking of the TCR with 10 µg/ml goat anti-hamster IgG (Thermo Fisher, Cat no. 31115) or goat anti-mouse IgG (Thermo Fisher, Cat no. 31160) antibodies in the case of murine and human CTLs, respectively. SERCA was blocked using 1 µM thapsigargin (Thermo Fisher, Cat no. T7458). 1 µg/ml ionomycin (Sigma, Cat no. I9657) was used sometimes as a positive control. Induction of calcium flux in the presence of 1.25 mM BAPTA was sometimes used as a negative control. Figures showing Ca^2+^ kinetics and their quantification depict the median of calcium sensor dye fluorescence.

Flow cytometric analyses were conducted on a BD LSRII or BD LSR Fortessa or BD FACSymphony A5 cell analyser, and data was analysed using FlowJo software (Tree Star Inc., version 10.4). Ca^2+^ flux analyses were performed on the FlowJo Kinetics platform.

### In vitro tamoxifen treatment

In vitro tamoxifen was given as 4-hydroxytamoxifen (4-OH-Tamoxifen or OHT) (Tocris, Cat no. 3412) solubilized in DMSO (Life Technologies, Cat no. D12345) in order to activate CreER^T2^ recombinase in vitro. Stock solutions were made at 100 mM and kept at −20 °C protected from light. Cells were treated at a concentration of 300 nM, diluted in complete RPMI (Gibco), and fresh 4-OHT was added daily for the duration of culture. Control-treated cells received DMSO in complete RPMI.

### Small molecule treatment of T cells

For small molecule treatment studies, naive or activated T cells were pre-incubated for 1 h (2 h in the case of BTP2 (Zitt et al, 2004), and 18 h for GANT61) prior to the assay. Doses of Ca^2+^ channel blockers were chosen based on doses established for use in T cells in the literature (see Appendix Table S4). All inhibitors were reconstituted in DMSO (Life Technologies, Cat no. D12345) or EtOH according to the manufacturer’s instructions and a carrier control was included for all experiments. For all the small molecules used in the study, see Appendix Table S4.

### qRT-PCR

Cells harvested for RNA extraction were washed twice in ice-cold PBS, snap-frozen as dry pellets, and stored at −80 °C. RNA was extracted using the RNAqeous™-Micro Total RNA Isolation Kit (murine cells) or the Single Cell RNA Purification Kit (Norgen Biotek Corp., Cat no. 51800) (human cells) or the Maxwell® RSC simplyRNA Cells Kit (Promega, Cat no. AS1390) (murine & human cells) according to the manufacturer’s instructions. RNA concentration was measured with a Nanodrop spectrophotometer (Labtech ND-1000) and samples were stored at −80 °C if not used immediately.

Reactions for qRT-PCR were set up using the One-Step qRT-PCR Kit (Thermo Fisher SuperScript III Platinum, cat no. 11732088) according to the manufacturer’s instructions using the Taqman probes indicated in Appendix Table S5. Each sample was run in triplicate with *TBP* and/or *CD3ε* used as housekeeping genes. In addition, each experiment included a non-template control and primer/probes were validated by no RT controls. Samples were run on a QuantStudio 6 Flex Real-Time PCR System (Thermo Fisher). Levels of Gli1 mRNA were determined using probe (Mm00494654_m1) and confirmed in some of the experiments with a second probe (Mm00494645_m1).

Expression of the gene transcript of interest was calculated with the ΔCt method (Schmittgen and Livak, 2008). The cycle threshold (Ct) value from the gene of interest was subtracted from the housekeeping gene and transformed with a factor of 2^(−ΔCt) to give the fold expression relative to the housekeeping gene.

### Immunofluorescence

HEK293T cells underwent immunofluorescence staining after 18 h of transfection with the human HA-tagged Ca_v_1.4 construct (containing an mScarlet reporter) while primary human CTLs at day 9–11 post-stimulation were plated onto glass slides at 4 × 10^6^/ml and allowed to adhere to the glass for 10 min at 37 °C in an incubator. To generate immune synapses, glass cover slips were coated with 1 μg/ml plate-bound anti-CD3ε antibodies (clone UCHT1, Biolegend, Cat no. 300438) for 1 h at 37 °C. After incubation, antibody solution was removed and CTLs were plated and allowed to form synapses for 5 min at 37 °C in an incubator. Cells were fixed with 4% PFA (16% PFA solution, CN Technical Services, cat no. 15710-s, 1x PBS) for 10 min at room temperature. Slides were washed 5 times with PBS and blocked with blocking buffer (PBS + 1% bovine serum albumin (BSA, Sigma, cat no. A3912; 50 g lyophilized powder) + 0.1% Triton X-100 (Alfa Aesar)) for 30 min at room temperature. Blocking buffer was aspirated and blocking buffer containing the respective primary antibodies (Appendix Table S6) was added. After staining for one hour at room temperature slides were washed 5 times with PBS + 0.1% Triton X-100. Secondary antibodies with phalloidin (Appendix Table S6) were added in blocking buffer (PBS + 1% BSA + 0.1% Triton X-100) and slides were incubated for 30 min light-protected at room temperature. After incubation, slides were washed 5 times with PBS + 0.1% Triton X-100 and stained with Hoechst (Hoechst 33342, trihydrochloride, trihydrate Invitrogen/Fisher, cat no. H3570, 1:30,000 dilution) prepared in PBS for 5 min light-protected at room temperature. Slides were washed five times with PBS and mounted with ProLong Diamond Antifade Mountant (Fisher, cat no. P36961). For Ca_v_1.4 antibody, similar results were obtained when another primary rabbit anti-Cav1.4 antibody (from Novus Biologicals, Cat no. NBP1-30667, Appendix Table S6) was used.

Confocal spinning disc microscopy was performed on an Andor Dragonfly 500 (Oxford Instruments) and images were processed using Imaris software (Bitplane/Oxford Instruments).

Colocalisation analysis was performed in Imaris 10.2 (Oxford Instrument). For each field of view, a region of interest (ROI) was created from the Ca_v_1.4 channel using a low threshold setting to select the cells. The colocalisation threshold was then calculated in the ROI using the method by Costes et al (2004). We report the Pearson correlation coefficients in the ROI calculated on the whole 3D data for each field of view. For a perfect colocalisation between the channels, the coefficient would be 1.0 whereas a random arrangement would result in a value close to zero.

### Western blot

Cells were lysed in ice-cold RIPA buffer (150 mM NaCl, 50 mM Tris-HCl pH 8.0, 1 mM MgCl_2_, 2% Triton) with protease inhibitor (Pierce, Cat. No. 88666). Samples were heated at 65 °C for 15 min and loaded together with a protein standard (Bio Rad, Cat no. 161-0394), onto a NuPAGE 4–12% gradient Bis/Tris Acrylamide gel (Thermo Fisher, Cat no. NP0335BOX). PAGE was run in Nu-PAGE MOPS running buffer (Thermo Fisher, Cat no. NP0001). Western Blotting was performed using wet transfer in Nu-PAGE Transfer Buffer (Thermo Fisher, Cat no. NP0006-01) + 10% Methanol (Honeywell, Cat no. 32213-2.5L). Membranes were blocked with 5% (w/v) nonfat dry milk (Marvel Original, Dried Skimmed Milk) in TBS before incubation with primary and secondary antibodies (Appendix Table S7). Membranes were developed with SuperSignal West Pico Plus Chemiluminescent Substrate (Thermo Fisher, Cat no. 34580).

Quantification of murine Gli1 protein levels were carried out using the ImageQuant^TM^ TL analysis software (Cytiva, version 10.2). Protein levels were normalised to the respective loading control in each experiment and then normalised to carrier control.

### Retroviral transduction

Retroviral transduction was used to generate primary CD8^+^ T cells overexpressing core Hedgehog components and to generate MC38 or EL4 tumour cells stably expressing the T4 or N4 Ova peptide (MC38-OvaT4, EL4-OvaN4), respectively. pMig-OvaT4 and pMig-OvaN4 were a generous gift from Dietmar Zehn (Technical University of Munich) (Zehn et al, 2009).

#### Generation of retrovirus

One day prior to the generation of retrovirus, HEK 293T cells were seeded in a six well plate resulting in 75% confluency the next day. Media was replaced with 2 ml fresh DMEM per well prior to transfection with retroviral plasmids. HEK 293T cells were transfected with 1.3 µg of packaging plasmid pCL-Eco (generous gift from Gillian Griffiths, Cambridge, UK) and 1.9 µg pMig vector—either 5’LTR-eGFP-IRES-rThy1.1(CD90.1)-3’LTR (referred to as “empty vector”), 5’LTR-Smo-C terminal eGFP fusion-STOP-IRES-rThy1.1(CD90.1)-3’LTR (referred to as “SmoM2”) or 5’LTR-Ihh-STOP-IRES-rThy1.1(CD90.1)-3’LTR (referred to as “Ihh”) or 5’LTR-OvaT4-eGFP-3’LTR (referred to as “OvaT4”) or 5’LTR-OvaN4-eGFP-3’LTR (referred to as “OvaN4”)—prepared in Opti-MEM™ media (Gibco, Cat no. 31985070). Concurrently, a 1:25 dilution of Lipofectamine™ 2000 (Invitrogen, Cat no. 11668019) was prepared in Opti-MEM™ media. The DNA-Opti-MEM™ solution was mixed with the Lipofectamine™2000-Opti-MEM™ solution at a ratio of 1:1 and incubated for 5 min at room temperature after which 400 µl of the mixture was added drop-wise to the HEK-293T cells. Media was replaced 18 h after transfection and collected 48 h post transfection. The retroviral supernatant was passed through a 0.45 µm PVDF membrane filter (Merck Millipore, Cat no. SLHVM33RS) for immediate use.

#### Retroviral transduction

Naive CD8^+^ T cells were isolated from C57BL/6 spleens and stimulated with plate-bound anti-CD3ε/CD28 antibodies as described previously. After 24 h of stimulation, T cell media was partially withdrawn and replaced by retroviral supernatant in a 1:2 ratio supplemented with protamine sulphate (Sigma, Cat no. 1101230005) at a final concentration of 9 µg/ml and IL-2 to maintain the standard CD8^+^ T cell expansion conditions as described above. Cells were then centrifuged at 680 × *g* for 15 min at 32 °C and subsequently placed in a humidified cell culture incubator. After 48 h cells were centrifuged, retroviral supernatant was removed and the cells subsequently cultured in complete RPMI + IL-2.

MC38 colorectal cancer cells were thawed and plated at 0.25 × 10^6^ per well of a 6-well plate in 3 ml media. After 16 h, 2 ml of media was removed from the cells and 2 ml fresh retroviral supernatant was added in the presence of 9 µg/ml protamine sulphate and spinfection (500 × *g* for 15 min at 32 °C) was performed. Cells were incubated for 72 h, after which they were washed and cultured in DMEM + 10% FCS.

5 × 10^6^ EL4 cells were thawed and plated in 1 mL per well in a 6-well plate. Cells were immediately transduced with 2 mL of fresh retroviral supernatant containing protamine sulfate at a final concentration of 6 μg/mL. Cells were then centrifuged at 680 × *g* for 15 min at 32 °C and incubated at 37 °C in a humidified incubator for 72 h.

### Cell sorting

#### Magnetic sorting of retrovirally transduced cells

For downstream applications such as qRT-PCR, retrovirally-transduced cell populations were enriched for transduced cells measured by the presence Thy1.1 expression. T cell populations were subjected to magnetic sorting at day 3 or 4 after initial stimulation. Thy1.1^+^ cells were positively enriched using the EasySep™ PE Positive Selection Kit (Stem Cell Technologies, Cat no. 17684) or EasySep™ Release Mouse PE Positive Selection Kit (Stem Cell Technologies, Cat no. 17656) according to the manufacturer’s instructions. Cells were labelled with a PE-conjugated Thy1.1 antibody (Appendix Table S3) at a final concentration of 0.25 mg/ml in MACS buffer. All steps were performed in MACS buffer and all incubations were performed at room temperature. PE positive cells were enriched using an EasyEights™ magnet (Stem Cell Technologies, Cat no. 18103). Three rounds of separation were performed in total and cells were washed in complete media prior to maintenance in culture with complete RPMI + IL-2. Purity of the sort was routinely over 95% as measured by FACS.

#### Fluorescence-activated cell sorting

Retrovirally transduced MC38-OvaT4 and EL4-OvaN4 cells were sorted by FACS on day 10 and day 9, respectively, following retroviral transduction based on GFP expression. Briefly, cells were trypsinised (MC38) or collected (EL4), washed and resuspended in MACS buffer containing DAPI (100 ng/ml) at 5 × 10^6^/ml concentration and passed through a 0.45 µm membrane filter before sorting DAPI^-ve^, GFP^+ve^ cells on a BD FACSAria IIu cell sorter. Purity of the sorted cells was above 98%.

Electroporated day 20–24 human CD8^+^ T cells containing Ca_v_1.4 constructs and empty vector were sorted by FACS based on GFP expression. Briefly, cells were collected, stained with anti-CD8a BUV737 antibodies for 20 min at 4 °C and resuspended in MACS buffer containing DAPI at 5 × 10^6^/ml concentration. Cells were passed through a 0.45 µm filter before sorting DAPI^-ve^, CD8^+ve^, GFP^+ve^ cells on a BD FACSAria IIu cell sorter. Purity of the sorted cells was above 97%. Sorted CD8^+^ T cells were washed twice and resuspended at 1 × 10^6^/ml concentration in Immunocult media with human IL-2 and Penicillin/Streptomycin. Cells were seeded on 96-well round-bottom plates coated with 1 µg/ml anti-CD3ε antibodies and restimulated for 72 h.

### Nucleofection of CTLs

The GFP-VIVIT plasmid was a gift from Anjana Rao (Addgene Plasmid # 11106; http://n2t.net/addgene:11106; RRID:Addgene_1106) which contains a fusion of MAGPHPVIVITGPHEE to the N-terminus EGFP, resulting in selective inhibition of the interaction between calcineurin and NFAT (Aramburu et al, 1999). The I*κ*Bα dominant negative (“I*κ*B DN”) plasmid was a generous gift from Felix Randow (Laboratory of Molecular Biology, Cambridge). The plasmid contains an I*κ*Bα which is resistant to proteosomal degradation due to two amino acid substitutions (S32 → A32 and S36 → A36) resulting in a dominant-negative phenotyping inhibiting NF-*κ*B induced transcription (Brockman et al, 1995).

Splenocytes from * Rag2* KO *OT-I* mice were stimulated with 10 nM Ova (SIINFEKL) peptide for 48 h and subsequently washed daily (resuspended at 1 × 10^6^ cells/ml). On day 6 CTLs were subjected to lympholyte treatment (Cedarlane, CL5035) per the manufacturer’s instructions. On day 7 cells were nucleofected using the Amaxa Mouse T Cell Nucleofector Kit (Lonza, Cat no. VVPA-1006) per the manufacturer’s instructions with 5 × 10^6^ cells and 1.5 µg plasmid DNA per cuvette. Cells were electroporated using a Nucleofector 2b Device (Lonza) on programme X-001. Cells were restimulated 18 h post nucleofection on day 8.

### Lipofection of HEK293T with HA-tagged human Ca_v_1.4

0.4 × 10^6^ HEK293T cells per well were seeded in 6-well plates on round coverslips. The next day, cells were transfected by first preparing 1.5 µg pHR’-CACNA1F(WT)-HA-T2A-mScarlet plasmid in Opti-MEM™ media. Concurrently, a 1:25 dilution of Lipofectamine™ 2000 was prepared in Opti-MEM™ media. The DNA-Opti-MEM™ solution was mixed with the Lipofectamine™2000-Opti-MEM™ solution at a ratio of 1:1 and incubated for 5 min at room temperature after which 400 µl of the mixture was added dropwise to HEK-293T cells. 18 h post transfection cells were used for immunofluorescence staining.

### Generation of Ca_v_1.4 constructs

#### Site-directed mutagenesis

pcDNA3.1 + /C-(K)-DYK containing the human Ca_v_1.4 sequence (Cat no. OHu21305) was purchased from GenScript. As the plasmid from Genscript was isolated from the dam^-ve^ strain ER2925, the plasmid was first transformed into dam^+ve^ NEM® 5-alpha Competent *E. coli* (Cat no. C2987H) as per the manufacturer’s instructions and grown at 37 °C. Transformation reactions were plated out on LB agar plates containing 100 µg/ml Ampicillin and grown overnight. Single colonies were picked and cultured in 7 ml LB in the presence of 100 µg/ml Ampicillin overnight. Plasmid DNA was isolated using a QiaPrep Spin Miniprep Kit (Qiagen, Cat No. 27104). Plasmids were subjected to diagnostic restriction digest on an agarose gel and appropriate working stocks were generated from 250 ml bacterial cultures using the HiSpeed Plasmid Maxi Kit (Qiagen, Cat no. 12662).

Site-directed mutagenesis was performed to introduce the Ca_v_1.4 gain-of-function mutation I745T (Hemara-Wahanui et al, 2005; Hope et al, 2005) using the QuikChange II XL Site-Directed Mutagenesis Kit (Agilent, Cat no. 200522) according to the manufacturer’s instructions. The primers (5’ → 3’) used were: I745T forward (TTGTCCACAGCAGTGGCAAGAAACACGTTCAACAGG) and I745T reverse (CCTGTTGAACGTGTTTCTTGCCACTGCTGTGGACAA). Transformation reactions were plated out on LB agar plates containing 100 µg/ml Ampicillin and grown overnight. Single colonies were picked and cultured in 7 ml LB in the presence of 100 µg/ml Ampicillin overnight. Plasmids were isolated using a QiaPrep Spin Miniprep Kit and subjected to diagnostic restriction digest on an agarose gel and appropriate working stocks were generated from 250 ml bacterial cultures using the HiSpeed Plasmid Maxi Kit (Qiagen, Cat no. 12662). Presence of the mutation was confirmed by Sanger sequencing (Source Bioscience).

#### Gibson assembly

The wild-type (WT) or I745T Ca_v_1.4 insert from pcDNA3.1 + /C-(K)-DYK and the T2A-eGFP insert from pSpCas9(BB)-T2A-GFP (PX458, Addgene, Cat no. 48138) were PCR amplified using the Q5^®^ High-Fidelity DNA Polymerase (New England Biolabs, Cat no. M0491L). The backbone was generated by restriction digest from pPBCAG-rtTAM2-IH (a generous gift from Marc de la Roche, University of Cambridge) using XhoI (New England Biolabs, Cat no. R0146S) and NotI (New England Biolabs, Cat no. R3189S). Gibson assembly using the Gibson Assembly^®^ Master Mix (New England Biolabs, Cat no. E2611) was then used to generate pB-CACNA1F(WT)-T2A-GFP-IRES-Hygro (referred to as “WT”) and pB-CACNA1F(I745T)-T2A-GFP-IRES-Hygro (referred to as “I745T”). The pB-GFP-IRES-Hygro (referred to as “EV”) was generated using ligation reaction with the T4 DNA Ligase (New England Biolabs, Cat no. M0202S).

The WT Ca_v_1.4 insert from pcDNA3.1 + /C-(K)-DYK and the mScarlet reporter protein were PCR amplified using the Q5^®^ High-Fidelity DNA Polymerase (New England Biolabs, Cat no. M0491L), with PCR primers adding an HA-tag to the C-terminus of Ca_v_1.4 and a T2A sequence between the Ca_v_1.4-HA protein and the mScarlet reporter. The backbone was generated by restriction digest from a pHR-SIN lentiviral plasmid using Mlu-I (Thermo Fisher: FD0564) and Not-I (Thermo Fisher: FD0596). Gibson assembly using the Gibson Assembly^®^ Master Mix (New England Biolabs, Cat no. E2611) was used to generate the final pHR-CACNA1F(WT)-HA-T2A-mScarlet construct. Correct cloning was confirmed via Sanger sequencing and Oxford Nanopore sequencing.

### Electroporation of human CD8^+^ T cells with Ca_v_1.4 constructs

Electroporation of human CD8^+^ cells with Ca_v_1.4 constructs and empty vector was performed on day 2–3 following stimulation. Cells were washed twice in pre-warmed PBS prior to resuspension in 80 µl Buffer T (4 × 10^6^ cells/electroporation reaction). 30 µg of Ca_v_1.4 construct and 30 µg Piggybac transposase were added to the cell suspension prior to electroporation with the Neon™ electroporation system in 100 µl electroporation tips with three pulses of 1700 V each with a pulse width of 10 ms. Cells were left to recover in Immunocult cell culture media in the absence of antibiotics for 24 h. Cells were then centrifuged at 400 × *g* for 5 min and returned into culture in Immunocult media with antibiotics at the indicated concentrations and cultured as normal.

### ImageStream analysis of NF-*κ*B nuclear translocation

On day 8, 18 h after nucleofection, CTLs were counted and plated onto a 24-well plate pre-coated overnight with 2.5 µg/ml anti-CD3ε antibody. After 1 h of stimulation, cells were harvested and washed once in PBS prior to staining with eFluor780 for 10 min light-protected at room temperature. Cells were subsequently washed in perm wash buffer—made up of 2% FCS, 0.1% Sodium Azide, 0.1% Triton X-100 (Alfa Aesar, Cat no. A16046.AE) in PBS prior to fixation in 4% PFA for 10 min light-protected at room temperature. Cells were washed twice in perm wash buffer before staining in 100 µl perm wash buffer containing anti-p65 APC antibody (Appendix Table S4) for 30 min light-protected at room temperature. Cells were washed twice in perm wash buffer, subsequently resuspended in 50 µl perm wash buffer and transferred to a 1.5 ml Eppendorf tube. DAPI was added (0.5 mg/ml) immediately prior to acquisition on an Amnis ImageStream (Millipore Sigma) imaging cytometer. Data was analysed with IDEAS Software (Millipore Sigma).

### RNA-Seq experiments

#### Naive CD8^+^ treatment with FPL 64176

24-well plates were coated overnight with 350 µl PBS solution containing plate-bound anti-CD3ε (1 µg/ml) and anti-CD28 (2 µg/ml) antibodies. Naive murine CD8^+^ T cells were isolated using negative selection (Naive CD8^+^ T Cell Isolation Kit, Cat no. 130-096-543, Miltenyi Biotec) from 6 * Rag1 * KO *OT-I* mice according to the manufacturer’s instructions. Cells were counted, washed and resuspended at 1 × 10^6^/ml in complete T cell media in the presence of 10 µM FPL 64176 or carrier control and incubated in a humidified incubator for 50 min. Following pre-incubation, PBS antibody coating was removed from the wells and 500 µl cells in carrier- or FPL 64176-containing media were plated on the stimulation plates. Following 3 h and 24 h of stimulation, cells harvested for RNA extraction were washed twice in ice-cold PBS, snap-frozen as dry pellets, and stored at −80 °C.

RNA was extracted using the Purelink^TM^ RNA Mini Kit (Thermo, Cat no. 12183025) according to the manufacturer’s instructions. RNA concentration was measured with the Qubit^TM^ RNA HS Assay kit (Thermo, Cat no. Q32852). RNA integrity was measured with a capillary electrophoresis system (4200 Tapestation, Agilent). 3 h samples were measured using a High Sensitivity RNA ScreenTape (Agilent, Cat no. 5067-5579) while 24 h samples with a regular RNA ScreenTape (Agilent, Cat no. 5067-5576) after which they were stored at –80 °C. Each sample had a RIN value of >8.

Library preparation was conducted according to the Illumina Stranded mRNA Prep reference guide (Illumina, Cat no. 1000000124518 v02). PCR cycles were adjusted based on RNA input (12 and 11 cycles for 3 h and 24 h samples, respectively). A pool of 24 libraries with paired-end and 50 bp-long reads was sequenced on NovaSeq. The average number of reads per sample was approximately 25 million. There are six replicates for each sample group. The reads were quantified against the mouse genome assembly GRCm39 using Salmon (version 1.9.0) (Patro et al, 2017). The DEseq2 package (version 1.26.0) (Love et al, 2014) was used for differential expression analysis. GSEA preranked analysis performed using GSEA software (version 3) (Subramanian et al, 2005).

Gli consensus sequences were extracted from Winklmayr et al (2010). A total of 11 Gli1 binding sequences were searched 2000 bp upstream and 200 bp downstream of transcription start sites for 79 differentially expressed genes.

### Analysis of Chip-Seq data

Coverage bigwig files were downloaded from GEO (GSE54191). The Integrative Genomics Viewer (IGV) was used for coverage analysis (Robinson et al, 2011).

### Statistics

Statistical analysis was performed using Prism 7 software (GraphPad Inc.). Details of the respective statistical tests used are noted in the figure legends. *p* < 0.05 was considered significant. Sample sizes were selected based on prior experience with similar studies in the same model, consultation with the Institute biostatistician, and the genotypes available at study initiation in order to provide sufficient statistical power while adhering to the 3Rs (Reduction) principle and the animal numbers approved by the Animal Welfare & Ethics Review Body of the Cancer Research UK – Cambridge Institute. Sample blinding was not applied.

### Study approval

Regarding animal experiments, all housing and procedures were performed in strict accordance with the United Kingdom Home Office Regulations and the Animal Welfare & Ethics Review Body of the Cancer Research UK – Cambridge Institute.

Healthy Donor samples were acquired in the form of Buffy Coats from NHS Blood and Transplant (NHSBT, Cambridge) or Quarter Leukopaks by Cambridge Bioscience (Cat no. LKP1DC4ACD50-X5XX) and processed under appropriate ethics (Research into Altered Lymphocyte Function in Health and Disease, REC reference: 17/YH/0304).

### Graphics

Figure 10 and synopsis image were created with BioRender.com.

## Supplementary information

Appendix

Peer Review File

Source data Fig. 1

Source data Fig. 2

Source data Fig. 3

Source data Fig. 4

Source data Fig. 5

Source data Fig. 7

Source data Fig. 8

Source data Fig. 9

Source data Fig. 10

Source data Fig. 6

Source data for Expanded View and Appendix

Expanded View Figures

## Data Availability

The datasets produced in this study are available in the following databases: RNA-Seq data: Gene Expression Omnibus GSE305142. The source data of this paper are collected in the following database record: biostudies:S-SCDT-10_1038-S44319-026-00810-8.

## References

[CR1] Adachi K, Davis MM (2011) T-cell receptor ligation induces distinct signaling pathways in naive vs. antigen-experienced T cells. Proc Natl Acad Sci USA 108:1549–1554 21205892 10.1073/pnas.1017340108 PMC3029746

[CR2] Ahmed H, Akbari H, Emami A, Akbari MR (2017) Genetic overview of syndactyly and polydactyly. Plast Reconstr Surg Glob Open 5:e1549 29263957 10.1097/GOX.0000000000001549 PMC5732663

[CR3] Aramburu J, Yaffe MB, Lopez-Rodriguez C, Cantley LC, Hogan PG, Rao A (1999) Affinity-driven peptide selection of an NFAT inhibitor more selective than cyclosporin A. Science 285:2129–2133 10497131 10.1126/science.285.5436.2129

[CR4] Atsuta Y, Tomizawa RR, Levin M, Tabin CJ (2019) L-type voltage-gated Ca(2+) channel CaV1.2 regulates chondrogenesis during limb development. Proc Natl Acad Sci USA 116:21592–21601 31591237 10.1073/pnas.1908981116 PMC6815189

[CR5] Badou A, Jha MK, Matza D, Flavell RA (2013) Emerging roles of L-type voltage-gated and other calcium channels in T lymphocytes. Front Immunol 4:243 24009608 10.3389/fimmu.2013.00243 PMC3757574

[CR6] Badou A, Jha MK, Matza D, Mehal WZ, Freichel M, Flockerzi V, Flavell RA (2006) Critical role for the beta regulatory subunits of Cav channels in T lymphocyte function. Proc Natl Acad Sci USA 103:15529–15534 17028169 10.1073/pnas.0607262103 PMC1622857

[CR7] Bannard O, Kraman M, Fearon DT (2009) Secondary replicative function of CD8+ T cells that had developed an effector phenotype. Science 323:505–509 19164749 10.1126/science.1166831 PMC2653633

[CR8] Baxter AJ, Dixon J, Ince F, Manners CN, Teague SJ (1993) Discovery and synthesis of methyl 2,5-dimethyl-4-[2- (phenylmethyl)benzoyl]-1H-pyrrole-3-carboxylate (FPL 64176) and analogues: the first examples of a new class of calcium channel activator. J Med Chem 36:2739–2744 7692047 10.1021/jm00071a004

[CR9] Belgacem YH, Borodinsky LN (2011) Sonic hedgehog signaling is decoded by calcium spike activity in the developing spinal cord. Proc Natl Acad Sci USA 108:4482–4487 21368195 10.1073/pnas.1018217108 PMC3060219

[CR10] Bertin S, Aoki-Nonaka Y, de Jong PR, Nohara LL, Xu H, Stanwood SR, Srikanth S, Lee J, To K, Abramson L et al (2014) The ion channel TRPV1 regulates the activation and proinflammatory properties of CD4(+) T cells. Nat Immunol 15:1055–1063 25282159 10.1038/ni.3009 PMC4843825

[CR11] Bigby M, Markowitz JS, Bleicher PA, Grusby MJ, Simha S, Siebrecht M, Wagner M, Nagler-Anderson C, Glimcher LH (1993) Most gamma delta T cells develop normally in the absence of MHC class II molecules. J Immunol 151:4465–4475 8409413

[CR12] Briscoe J, Therond PP (2013) The mechanisms of Hedgehog signalling and its roles in development and disease. Nat Rev Mol Cell Biol 14:416–429 23719536 10.1038/nrm3598

[CR13] Brockman JA, Scherer DC, McKinsey TA, Hall SM, Qi X, Lee WY, Ballard DW (1995) Coupling of a signal response domain in I kappa B alpha to multiple pathways for NF-kappa B activation. Mol Cell Biol 15:2809–2818 7739562 10.1128/mcb.15.5.2809 PMC230512

[CR14] Brown DM, Lee S, Garcia-Hernandez Mde L, Swain SL (2012) Multifunctional CD4 cells expressing gamma interferon and perforin mediate protection against lethal influenza virus infection. J Virol 86:6792–6803 22491469 10.1128/JVI.07172-11 PMC3393557

[CR15] Brownell I, Guevara E, Bai CB, Loomis CA, Joyner AL (2011) Nerve-derived sonic hedgehog defines a niche for hair follicle stem cells capable of becoming epidermal stem cells. Cell Stem Cell 8:552–565 21549329 10.1016/j.stem.2011.02.021 PMC3089905

[CR16] Brownlie RJ, Zamoyska R (2013) T cell receptor signalling networks: branched, diversified and bounded. Nat Rev Immunol 13:257–269 23524462 10.1038/nri3403

[CR17] Cabral MD, Paulet PE, Robert V, Gomes B, Renoud ML, Savignac M, Leclerc C, Moreau M, Lair D, Langelot M et al (2010) Knocking down Cav1 calcium channels implicated in Th2 cell activation prevents experimental asthma. Am J Respir Crit Care Med 181:1310–1317 20167851 10.1164/rccm.200907-1166OC

[CR18] Chen XL, Chinchilla P, Fombonne J, Ho L, Guix C, Keen JH, Mehlen P, Riobo NA (2014) Patched-1 proapoptotic activity is downregulated by modification of K1413 by the E3 ubiquitin-protein ligase Itchy homolog. Mol Cell Biol 34:3855–3866 25092867 10.1128/MCB.00960-14 PMC4187711

[CR19] Colucci A, Giunti R, Senesi S, Bygrave FL, Benedetti A, Gamberucci A (2009) Effect of nifedipine on capacitive calcium entry in Jurkat T lymphocytes. Arch Biochem Biophys 481:80–85 18950601 10.1016/j.abb.2008.10.002

[CR20] Costes SV, Daelemans D, Cho EH, Dobbin Z, Pavlakis G, Lockett S (2004) Automatic and quantitative measurement of protein-protein colocalization in live cells. Biophys J 86:3993–4003 15189895 10.1529/biophysj.103.038422 PMC1304300

[CR21] Crompton T, Outram SV, Hager-Theodorides AL (2007) Sonic hedgehog signalling in T-cell development and activation. Nat Rev Immunol 7:726–735 17690714 10.1038/nri2151

[CR22] de la Roche M, Asano Y, Griffiths GM (2016) Origins of the cytolytic synapse. Nat Rev Immunol 16:421–432 27265595 10.1038/nri.2016.54

[CR23] de la Roche M, Ritter AT, Angus KL, Dinsmore C, Earnshaw CH, Reiter JF, Griffiths GM (2013) Hedgehog signaling controls T cell killing at the immunological synapse. Science 342:1247–1250 24311692 10.1126/science.1244689 PMC4022134

[CR24] de Vries NL, van de Haar J, Veninga V, Chalabi M, Ijsselsteijn ME, van der Ploeg M, van den Bulk J, Ruano D, van den Berg JG, Haanen JB et al (2023) gammadelta T cells are effectors of immunotherapy in cancers with HLA class I defects. Nature 613:743–750 36631610 10.1038/s41586-022-05593-1 PMC9876799

[CR25] Erdogmus S, Concepcion AR, Yamashita M, Sidhu I, Tao AY, Li W, Rocha PP, Huang B, Garippa R, Lee B et al (2022) Cavbeta1 regulates T cell expansion and apoptosis independently of voltage-gated Ca(2+) channel function. Nat Commun 13:2033 35440113 10.1038/s41467-022-29725-3 PMC9018955

[CR26] Fenninger F, Han J, Stanwood SR, Nohara LL, Arora H, Choi KB, Munro L, Pfeifer CG, Shanina I, Horwitz MS et al (2019) Mutation of an L-type calcium channel gene leads to T lymphocyte dysfunction. Front Immunol 10:2473 31736943 10.3389/fimmu.2019.02473 PMC6833481

[CR27] Griffiths GM, Tsun A, Stinchcombe JC (2010) The immunological synapse: a focal point for endocytosis and exocytosis. J Cell Biol 189:399–406 20439993 10.1083/jcb.201002027 PMC2867296

[CR28] Hainberger D, Stolz V, Zhu C, Schuster M, Muller L, Hamminger P, Rica R, Waltenberger D, Alteneder M, Krausgruber T et al (2020) NCOR1 orchestrates transcriptional landscapes and effector functions of CD4(+) T cells. Front Immunol 11:579 32318068 10.3389/fimmu.2020.00579 PMC7147518

[CR29] Halle S, Keyser KA, Stahl FR, Busche A, Marquardt A, Zheng X, Galla M, Heissmeyer V, Heller K, Boelter J et al (2016) In vivo killing capacity of cytotoxic T cells is limited and involves dynamic interactions and T cell cooperativity. Immunity 44:233–245 26872694 10.1016/j.immuni.2016.01.010 PMC4846978

[CR30] Hanna J, Beke F, O’Brien LM, Kapeni C, Chen HC, Carbonaro V, Kim AB, Kishore K, Adolph TE, Skjoedt MO et al (2022) Cell-autonomous Hedgehog signaling controls Th17 polarization and pathogenicity. Nat Commun 13:4075 35835905 10.1038/s41467-022-31722-5 PMC9281293

[CR31] Hemara-Wahanui A, Berjukow S, Hope CI, Dearden PK, Wu SB, Wilson-Wheeler J, Sharp DM, Lundon-Treweek P, Clover GM, Hoda JC et al (2005) A CACNA1F mutation identified in an X-linked retinal disorder shifts the voltage dependence of Cav1.4 channel activation. Proc Natl Acad Sci USA 102:7553–7558 15897456 10.1073/pnas.0501907102 PMC1140436

[CR32] Hope CI, Sharp DM, Hemara-Wahanui A, Sissingh JI, Lundon P, Mitchell EA, Maw MA, Clover GM (2005) Clinical manifestations of a unique X-linked retinal disorder in a large New Zealand family with a novel mutation in CACNA1F, the gene responsible for CSNB2. Clin Exp Ophthalmol 33:129–136 15807819 10.1111/j.1442-9071.2005.00987.x

[CR33] Infante P, Alfonsi R, Botta B, Mori M, Di Marcotullio L (2015) Targeting GLI factors to inhibit the Hedgehog pathway. Trends Pharmacol Sci 36:547–558 26072120 10.1016/j.tips.2015.05.006

[CR34] Isakov N, Altman A (2012) PKC-theta-mediated signal delivery from the TCR/CD28 surface receptors. Front Immunol 3:273 22936936 10.3389/fimmu.2012.00273 PMC3425079

[CR35] Jayachandran R, Gumienny A, Bolinger B, Ruehl S, Lang MJ, Fucile G, Mazumder S, Tchang V, Woischnig AK, Stiess M et al (2019) Disruption of coronin 1 signaling in T cells promotes allograft tolerance while maintaining anti-pathogen immunity. Immunity 50:152–165.e158 30611611 10.1016/j.immuni.2018.12.011

[CR36] Jha MK, Badou A, Meissner M, McRory JE, Freichel M, Flockerzi V, Flavell RA (2009) Defective survival of naive CD8+ T lymphocytes in the absence of the beta3 regulatory subunit of voltage-gated calcium channels. Nat Immunol 10:1275–1282 19838200 10.1038/ni.1793 PMC2785134

[CR37] Kersh EN (2006) Impaired memory CD8 T cell development in the absence of methyl-CpG-binding domain protein 2. J Immunol 177:3821–3826 16951344 10.4049/jimmunol.177.6.3821

[CR38] Kochl R, Thelen F, Vanes L, Brazao TF, Fountain K, Xie J, Huang CL, Lyck R, Stein JV, Tybulewicz VL (2016) WNK1 kinase balances T cell adhesion versus migration in vivo. Nat Immunol 17:1075–1083 27400149 10.1038/ni.3495 PMC4994873

[CR39] Kotturi MF, Carlow DA, Lee JC, Ziltener HJ, Jefferies WA (2003) Identification and functional characterization of voltage-dependent calcium channels in T lymphocytes. J Biol Chem 278:46949–46960 12954628 10.1074/jbc.M309268200

[CR40] Kurachi M, Barnitz RA, Yosef N, Odorizzi PM, DiIorio MA, Lemieux ME, Yates K, Godec J, Klatt MG, Regev A et al (2014) The transcription factor BATF operates as an essential differentiation checkpoint in early effector CD8+ T cells. Nat Immunol 15:373–383 24584090 10.1038/ni.2834 PMC4000237

[CR41] Lin MY, Zal T, Ch’en IL, Gascoigne NR, Hedrick SM (2005) A pivotal role for the multifunctional calcium/calmodulin-dependent protein kinase II in T cells: from activation to unresponsiveness. J Immunol 174:5583–5592 15843557 10.4049/jimmunol.174.9.5583

[CR42] Lin Y, Cui C, Su M, Silbart LK, Liu H, Zhao J, He L, Huang Y, Xu D, Wei X et al (2021) Identification of TAPBPL as a novel negative regulator of T-cell function. EMBO Mol Med 13:e13404 33938620 10.15252/emmm.202013404 PMC8103088

[CR43] Love MI, Huber W, Anders S (2014) Moderated estimation of fold change and dispersion for RNA-seq data with DESeq2. Genome Biol 15:550 25516281 10.1186/s13059-014-0550-8 PMC4302049

[CR44] Majzner RG, Mackall CL (2019) Clinical lessons learned from the first leg of the CAR T cell journey. Nat Med 25:1341–1355 31501612 10.1038/s41591-019-0564-6

[CR45] Man K, Miasari M, Shi W, Xin A, Henstridge DC, Preston S, Pellegrini M, Belz GT, Smyth GK, Febbraio MA et al (2013) The transcription factor IRF4 is essential for TCR affinity-mediated metabolic programming and clonal expansion of T cells. Nat Immunol 14:1155–1165 24056747 10.1038/ni.2710

[CR46] Martinez GJ, Pereira RM, Aijo T, Kim EY, Marangoni F, Pipkin ME, Togher S, Heissmeyer V, Zhang YC, Crotty S et al (2015) The transcription factor NFAT promotes exhaustion of activated CD8(+) T cells. Immunity 42:265–278 25680272 10.1016/j.immuni.2015.01.006 PMC4346317

[CR47] McLellan JS, Zheng X, Hauk G, Ghirlando R, Beachy PA, Leahy DJ (2008) The mode of Hedgehog binding to Ihog homologues is not conserved across different phyla. Nature 455:979–983 18794898 10.1038/nature07358 PMC2679680

[CR48] Montagnani V, Stecca B (2019) Role of protein kinases in hedgehog pathway control and implications for cancer therapy. Cancers 11:449 30934935 10.3390/cancers11040449 PMC6520855

[CR49] Nguyen S, Chevalier MF, Benmerzoug S, Cesson V, Schneider AK, Rodrigues-Dias SC, Dartiguenave F, Lucca I, Jichlinski P, Roth B et al (2022) Vdelta2 T cells are associated with favorable clinical outcomes in patients with bladder cancer and their tumor reactivity can be boosted by BCG and zoledronate treatments. J Immunother Cancer 10:e004880 36002184 10.1136/jitc-2022-004880 PMC9413168

[CR50] Omilusik K, Priatel JJ, Chen X, Wang YT, Xu H, Choi KB, Gopaul R, McIntyre-Smith A, Teh HS, Tan R et al (2011) The Ca(v)1.4 calcium channel is a critical regulator of T cell receptor signaling and naive T cell homeostasis. Immunity 35:349–360 21835646 10.1016/j.immuni.2011.07.011

[CR51] Park HL, Bai C, Platt KA, Matise MP, Beeghly A, Hui CC, Nakashima M, Joyner AL (2000) Mouse Gli1 mutants are viable but have defects in SHH signaling in combination with a Gli2 mutation. Development 127:1593–1605 10725236 10.1242/dev.127.8.1593

[CR52] Patro R, Duggal G, Love MI, Irizarry RA, Kingsford C (2017) Salmon provides fast and bias-aware quantification of transcript expression. Nat Methods 14:417–419 28263959 10.1038/nmeth.4197 PMC5600148

[CR53] Peters C, Kouakanou L, Oberg HH, Wesch D, Kabelitz D (2020) In vitro expansion of Vgamma9Vdelta2 T cells for immunotherapy. Methods Enzymol 631:223–237 31948549 10.1016/bs.mie.2019.07.019

[CR54] Pietrobono S, Gagliardi S, Stecca B (2019) Non-canonical Hedgehog signaling pathway in cancer: activation of GLI transcription factors beyond smoothened. Front Genet 10:556 31244888 10.3389/fgene.2019.00556 PMC6581679

[CR55] Polizio AH, Chinchilla P, Chen X, Manning DR, Riobo NA (2011) Sonic Hedgehog activates the GTPases Rac1 and RhoA in a Gli-independent manner through coupling of smoothened to Gi proteins. Sci Signal 4:pt7 22114142 10.1126/scisignal.2002396 PMC5811764

[CR56] Qiu T, Cao J, Chen W, Wang J, Wang Y, Zhao L, Liu M, He L, Wu G, Li H et al (2020) 24-Dehydrocholesterol reductase promotes the growth of breast cancer stem-like cells through the Hedgehog pathway. Cancer Sci 111:3653–3664 32713162 10.1111/cas.14587 PMC7540995

[CR57] Quezada SA, Simpson TR, Peggs KS, Merghoub T, Vider J, Fan X, Blasberg R, Yagita H, Muranski P, Antony PA et al (2010) Tumor-reactive CD4(+) T cells develop cytotoxic activity and eradicate large established melanoma after transfer into lymphopenic hosts. J Exp Med 207:637–650 20156971 10.1084/jem.20091918 PMC2839156

[CR58] Robert V, Triffaux E, Savignac M, Pelletier L (2013) Singularities of calcium signaling in effector T-lymphocytes. Biochim Biophys Acta 1833:1595–1602 23266355 10.1016/j.bbamcr.2012.12.001

[CR59] Robinson JT, Thorvaldsdottir H, Winckler W, Guttman M, Lander ES, Getz G, Mesirov JP (2011) Integrative genomics viewer. Nat Biotechnol 29:24–26 21221095 10.1038/nbt.1754 PMC3346182

[CR60] Rovida E, Stecca B (2015) Mitogen-activated protein kinases and Hedgehog-GLI signaling in cancer: a crosstalk providing therapeutic opportunities? Semin Cancer Biol 35:154–167 26292171 10.1016/j.semcancer.2015.08.003

[CR61] Roychoudhuri R, Clever D, Li P, Wakabayashi Y, Quinn KM, Klebanoff CA, Ji Y, Sukumar M, Eil RL, Yu Z et al (2016) BACH2 regulates CD8(+) T cell differentiation by controlling access of AP-1 factors to enhancers. Nat Immunol 17:851–860 27158840 10.1038/ni.3441 PMC4918801

[CR62] Sahoo SS, Majhi RK, Tiwari A, Acharya T, Kumar PS, Saha S, Kumar A, Goswami C, Chattopadhyay S (2019) Transient receptor potential ankyrin1 channel is endogenously expressed in T cells and is involved in immune functions. Biosci Rep 39:BSR20191437 31488616 10.1042/BSR20191437 PMC6753326

[CR63] Samten B, Townsend JC, Weis SE, Bhoumik A, Klucar P, Shams H, Barnes PF (2008) CREB, ATF, and AP-1 transcription factors regulate IFN-gamma secretion by human T cells in response to mycobacterial antigen. J Immunol 181:2056–2064 18641343 10.4049/jimmunol.181.3.2056 PMC2587306

[CR64] Sanchez Martinez D, Tirado N, Mensurado S, Martinez-Moreno A, Romecin P, Gutierrez Aguera F, Correia DV, Silva-Santos B, Menendez P (2022) Generation and proof-of-concept for allogeneic CD123 CAR-Delta One T (DOT) cells in acute myeloid leukemia. J Immunother Cancer 10:e005400 36162920 10.1136/jitc-2022-005400 PMC9516293

[CR65] Sasaki N, Kurisu J, Kengaku M (2010) Sonic hedgehog signaling regulates actin cytoskeleton via Tiam1-Rac1 cascade during spine formation. Mol Cell Neurosci 45:335–344 20654717 10.1016/j.mcn.2010.07.006

[CR66] Schmittgen TD, Livak KJ (2008) Analyzing real-time PCR data by the comparative C(T) method. Nat Protoc 3:1101–1108 18546601 10.1038/nprot.2008.73

[CR67] Shafi S, Vantourout P, Wallace G, Antoun A, Vaughan R, Stanford M, Hayday A (2011) An NKG2D-mediated human lymphoid stress surveillance response with high interindividual variation. Sci Transl Med 3:113ra124 22133594 10.1126/scitranslmed.3002922 PMC3966512

[CR68] Shim S, Goyal R, Panoutsopoulos AA, Balashova OA, Lee D, Borodinsky LN (2023) Calcium dynamics at the neural cell primary cilium regulate Hedgehog signaling-dependent neurogenesis in the embryonic neural tube. Proc Natl Acad Sci USA 120:e2220037120 37252980 10.1073/pnas.2220037120 PMC10266006

[CR69] Shin GT, Cheigh JS, Riggio RR, Suthanthiran M, Stubenbord WT, Serur D, Wang JC, Rubin AL, Stenzel KH (1997) Effect of nifedipine on renal allograft function and survival beyond one year. Clin Nephrol 47:33–36 9021239

[CR70] Splawski I, Timothy KW, Sharpe LM, Decher N, Kumar P, Bloise R, Napolitano C, Schwartz PJ, Joseph RM, Condouris K et al (2004) Ca(V)1.2 calcium channel dysfunction causes a multisystem disorder including arrhythmia and autism. Cell 119:19–31 15454078 10.1016/j.cell.2004.09.011

[CR71] St-Jacques B, Hammerschmidt M, McMahon AP (1999) Indian hedgehog signaling regulates proliferation and differentiation of chondrocytes and is essential for bone formation. Genes Dev 13:2072–2086 10465785 10.1101/gad.13.16.2072 PMC316949

[CR72] Stottmann RW, Turbe-Doan A, Tran P, Kratz LE, Moran JL, Kelley RI, Beier DR (2011) Cholesterol metabolism is required for intracellular hedgehog signal transduction in vivo. PLoS Genet 7:e1002224 21912524 10.1371/journal.pgen.1002224 PMC3164675

[CR73] Strauss O, Mergler S, Wiederholt M (1997) Regulation of L-type calcium channels by protein tyrosine kinase and protein kinase C in cultured rat and human retinal pigment epithelial cells. FASEB J 11:859–867 9285484 10.1096/fasebj.11.11.9285484

[CR74] Subramanian A, Tamayo P, Mootha VK, Mukherjee S, Ebert BL, Gillette MA, Paulovich A, Pomeroy SL, Golub TR, Lander ES et al (2005) Gene set enrichment analysis: a knowledge-based approach for interpreting genome-wide expression profiles. Proc Natl Acad Sci USA 102:15545–15550 16199517 10.1073/pnas.0506580102 PMC1239896

[CR75] Takeuchi A, Saito T (2017) CD4 CTL, a cytotoxic subset of CD4(+) T cells, their differentiation and function. Front Immunol 8:194 28280496 10.3389/fimmu.2017.00194 PMC5321676

[CR76] Teperino R, Amann S, Bayer M, McGee SL, Loipetzberger A, Connor T, Jaeger C, Kammerer B, Winter L, Wiche G et al (2012) Hedgehog partial agonism drives Warburg-like metabolism in muscle and brown fat. Cell 151:414–426 23063129 10.1016/j.cell.2012.09.021

[CR77] Trebak M, Kinet JP (2019) Calcium signalling in T cells. Nat Rev Immunol 19:154–169 30622345 10.1038/s41577-018-0110-7 PMC6788797

[CR78] van Leeuwen EM, Remmerswaal EB, Vossen MT, Rowshani AT, Wertheim-van Dillen PM, van Lier RA, ten Berge IJ (2004) Emergence of a CD4+CD28- granzyme B+, cytomegalovirus-specific T cell subset after recovery of primary cytomegalovirus infection. J Immunol 173:1834–1841 15265915 10.4049/jimmunol.173.3.1834

[CR79] Weidinger C, Shaw PJ, Feske S (2013) STIM1 and STIM2-mediated Ca(2+) influx regulates antitumour immunity by CD8(+) T cells. EMBO Mol Med 5:1311–1321 23922331 10.1002/emmm.201302989 PMC3799488

[CR80] Winklmayr M, Schmid C, Laner-Plamberger S, Kaser A, Aberger F, Eichberger T, Frischauf AM (2010) Non-consensus GLI binding sites in Hedgehog target gene regulation. BMC Mol Biol 11:2 20070907 10.1186/1471-2199-11-2 PMC2830928

[CR81] Woehrle T, Yip L, Elkhal A, Sumi Y, Chen Y, Yao Y, Insel PA, Junger WG (2010) Pannexin-1 hemichannel-mediated ATP release together with P2X1 and P2X4 receptors regulate T-cell activation at the immune synapse. Blood 116:3475–3484 20660288 10.1182/blood-2010-04-277707 PMC2981474

[CR82] Wu B, Wang Y, Wang C, Wang GG, Wu J, Wan YY (2016) BPTF is essential for T cell homeostasis and function. J Immunol 197:4325–4333 27799308 10.4049/jimmunol.1600642 PMC5127169

[CR83] Xie J, Murone M, Luoh SM, Ryan A, Gu Q, Zhang C, Bonifas JM, Lam CW, Hynes M, Goddard A et al (1998) Activating Smoothened mutations in sporadic basal-cell carcinoma. Nature 391:90–92 9422511 10.1038/34201

[CR84] Yao S, Buzo BF, Pham D, Jiang L, Taparowsky EJ, Kaplan MH, Sun J (2013) Interferon regulatory factor 4 sustains CD8(+) T cell expansion and effector differentiation. Immunity 39:833–845 24211184 10.1016/j.immuni.2013.10.007 PMC3855863

[CR85] Zehn D, Lee SY, Bevan MJ (2009) Complete but curtailed T-cell response to very low-affinity antigen. Nature 458:211–214 19182777 10.1038/nature07657 PMC2735344

[CR86] Zhang J, Feng H, Zhao J, Feldman ER, Chen SY, Yuan W, Huang C, Akbari O, Tibbetts SA, Feng P (2016) IkappaB Kinase epsilon is an NFATc1 kinase that inhibits T cell immune response. Cell Rep 16:405–418 27346349 10.1016/j.celrep.2016.05.083 PMC5293007

[CR87] Zhovmer AS, Manning A, Smith C, Nguyen A, Prince O, Saez PJ, Ma X, Tsygankov D, Cartagena-Rivera AX, Singh NA et al (2024) Septins provide microenvironment sensing and cortical actomyosin partitioning in motile amoeboid T lymphocytes. Sci Adv 10:eadi1788 38170778 10.1126/sciadv.adi1788 PMC13155490

[CR88] Zhu DM, Dustin ML, Cairo CW, Thatte HS, Golan DE (2006) Mechanisms of cellular avidity regulation in CD2-CD58-mediated T cell adhesion. ACS Chem Biol 1:649–658 17168569 10.1021/cb6002515

[CR89] Zitt C, Strauss B, Schwarz EC, Spaeth N, Rast G, Hatzelmann A, Hoth M (2004) Potent inhibition of Ca2+ release-activated Ca2+ channels and T-lymphocyte activation by the pyrazole derivative BTP2. J Biol Chem 279:12427–12437 14718545 10.1074/jbc.M309297200

