# Supplementary material for: L-type voltage-gated Ca2+ channels control T cell killing via non-canonical Hedgehog signalling
Source: EMBO Rep. 2026 Jun 8;27(13):3689–730. doi: 10.1038/s44319-026-00810-8 (PMC13354577; doi:10.1038/s44319-026-00810-8)
Supplement: Supplementary file 1 — Appendix [file 44319_2026_810_MOESM1_ESM.pdf]

Appendix

Table of contents:

**Appendix Figure S1.** RNA-Seq analysis of FPL 64176- and carrier-treated CD8+ T cells after 3h	p2
**Appendix Figure S2.** RNA-Seq analysis of FPL 64176- and carrier-treated CD8+ T cells after 24h	p3
**Appendix Table S1.** List of crRNA used for CRISPR in CD8+ T cells	p4
**Appendix Table S2.** PCR Primers used for genome editing efficiency assays	p5
**Appendix Table S3.** List of antibodies used for flow cytometry	p6
**Appendix Table S4.** Overview of small molecules used	p8
**Appendix Table S5.** Taqman probes (Thermo Fisher) used for qRT-PCR	p9
**Appendix Table S6.** Antibodies used for immunofluorescence staining	p10
**Appendix Table S7.** Antibodies used for Western Blotting	p11
**Appendix Table S8.** List of Gli1 binding sites used in analysis of RNA-Seq data	p12
**Appendix Table S9.** List of transcripts upregulated at 24h by FPL 641761 with Gli1 binding sites in their promoter regions	p13
**Appendix references**	p14

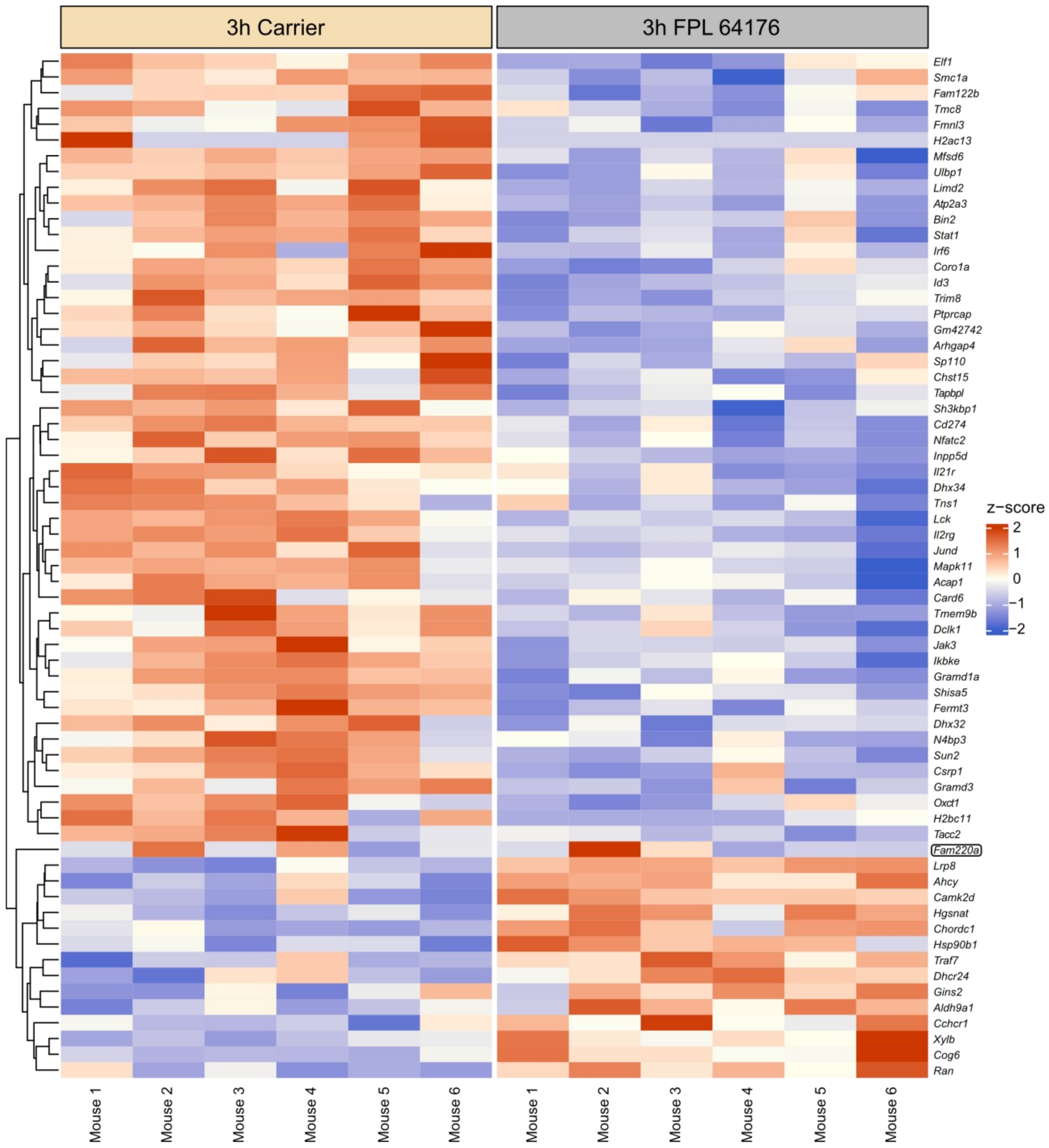

Appendix Figure S1. RNA-Seq analysis of FPL 64176- and carrier-treated CD8+ T cells after 3h
Naïve CD8⁺ T cells were isolated from *OTI Rag1KO* mice and stimulated for 3h with plate-bound anti-CD3ε and anti-CD28 in the presence of 10mM FPL 64176 or carrier control before RNA was extracted for RNA-Seq analysis. Heatmap depicting relative Z scores of statistically significant differentially downregulated and upregulated genes, respectively. The rows are scaled using the Z score. Euclidean distances and the complete clustering method are employed for the clustering of genes. n=6 biological replicates. Boxes indicate genes with one of 11 Gli consensus sequences (Winklmayr *et al*, 2010).

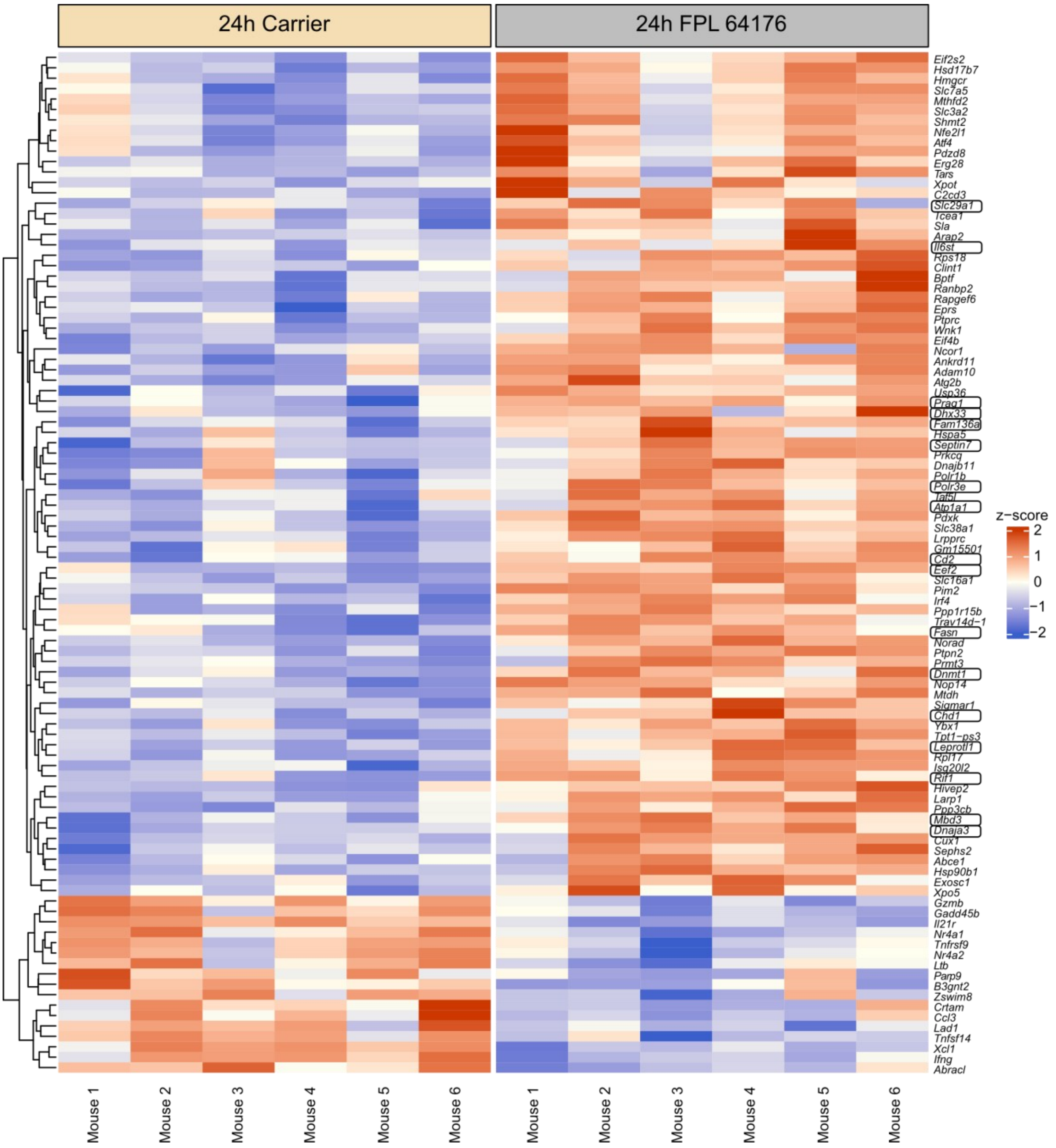

Appendix Figure S2. RNA-Seq analysis of FPL 64176- and carrier-treated CD8+ T cells after 24h
Naïve CD8⁺ T cells were isolated from *OTI Rag1KO* mice and stimulated for 24h with plate-bound anti-CD3ε and anti-CD28 in the presence of 10mM FPL 64176 or carrier control before RNA was extracted for RNA-Seq analysis. Heatmap depicting relative Z scores of statistically significant differentially downregulated and upregulated genes, respectively. The rows are scaled using the Z score. Euclidean distances and the complete clustering method are employed for the clustering of genes. n=6 biological replicates. Boxes indicate genes with one of 11 Gli consensus sequences (Winklmayr *et al*, 2010).

Appendix Table S1. List of crRNA used for CRISPR in CD8⁺ T cells

Guide	Sequence (5'→ 3')
Non-targeting control 1	Proprietary (IDT, Cat No. 1072544)
Non-targeting control 2	Proprietary (IDT, Cat No. 10725455)
Ca_v1.1#1 (*Cacna1s*)	AGCAAGAUGAUGGUCUCGAA
Ca_v1.1#2 (*Cacna1s*)	TGAAGAGGGTCAGCAACACG
Ca_v1.2#1 (*Cacna1c*)	CTACCGTCAGTTCCACACAG
Ca_v1.2#2 (*Cacna1c*)	CTACCGTCAGTTCCACACAG
Ca_v1.3#1 (*Cacna1d*)	GTCGAAGTGGTCTTAACACT
Ca_v1.3#2 (*Cacna1d*)	TCTGAACATGGTCTTCACAG
Ca_v1.4#1 (*Cacna1f*)	GTTCCTGGTGATTTTCACCG
Ca_v1.4#2 (*Cacna1f*)	TCCTGAGGAGACGTACACAT

Appendix Table S2. PCR Primers used for genome editing efficiency assays

Primer	Sequence (5'→3')
Cacna1d forward	TGGACACTTTGTTGGCATGA
Cacna1d reverse	AGTTTTACCAAGGGCAGAGC
Cacna1f forward	CCACAACTTGGTGAGGTCTA
Cacna1f reverse	GAGGTCGTAGCACCCTAAAT

Appendix Table S3. List of antibodies used for flow cytometry

Target	Clone	Reactivity	Fluorochrome	Dilution	Cat no.	Supplier
CCR7	G043H7	Human	PE/Cy7	1:50	353226	Biolegend
CD3ε	145-2C11	Mouse	BUV395	1:50	563565	BD
CD3ε	UCHT1	Human	BUV395	1:100	563546	BD
CD4	RM4-5	Mouse	BV605	1:200	100548	Biolegend
CD4	RM4-5	Mouse	APC	1:200	100516	Biolegend
CD4	RM4-5	Mouse	FITC	1:200	100510	Biolegend
CD4	RM4-5	Mouse	PE	1:200	100512	Biolegend
CD4	RPA-T4	Human	BV605	1:100	300556	Biolegend
CD8a	53-6.7	Mouse	BV605	1:200	100744	Biolegend
CD8a	53-6.7	Mouse	APC	1:200	100712	Biolegend
CD8a	53-6.7	Mouse	FITC	1:200	100706	Biolegend
CD8a	53-6.7	Mouse	PE	1:200	100707	Biolegend
CD8a	53-6.7	Mouse	BUV737	1:200	612759	BD
CD8	RPA-T8	Human	BV711	1:100	301044	Biolegend
CD8	HIT8a	Human	BUV737	1:100	741850	BD
CD27	LG.3A10	Human	PerCP/Cy5.5	1:50	124214	Biolegend
CD28	CD28.2	Human	BV785	1:50	302950	Biolegend
CD44	IM7	Mouse/Human	BV650	1:400	103049	Biolegend
CD44	IM7	Mouse/Human	BV785	1:400	103059	Biolegend
CD44	IM7	Mouse/Human	BV711	1:400	103057	Biolegend
CD44	IM7	Mouse/Human	PE/Cy7	1:400	103030	Biolegend
CD45	30-F11	Mouse	PE/Cy7	1:200	103114	Biolegend
CD45	30-F11	Mouse	AF700	1:400	103128	Biolegend
CD45	HI30	Human	FITC	1:100	304006	Biolegend
CD45RO	UCHL-1	Human	APC	1:100	304210	Biolegend
CD62L	MEL-14	Mouse	BV421	1:200	104436	Biolegend
CD62L	MEL-14	Mouse	BV510	1:200	104441	Biolegend
CD90.1	HIS51	Mouse/Rat	APC	1:200	17-0900-82	BD
CD90.1	HIS51	Mouse/Rat	PE	1:200	12-0900-83	Thermo
CD95	DX2	Human	BV421	1:50	305624	Biolegend
PD1	RMP1-30	Mouse	BV421	1:50	109121	Biolegend
TIM3	RMT3-23	Mouse	BV605	1:100	119721	Biolegend
GzmB	GB11	Mouse/Human	Alexa Fluor 647	1:200	515406	Biolegend
IFN-γ	XMG1.2	Mouse	BV711	1:100	505835	Biolegend
IFN-γ	4S.B3	Human	BV650	1:100	563416	BD

TNF-α	Mab11	Human	BV421	1:100	502932	Biolegend
IL-2	JES6-5H4	Mouse	PE	1:100	503808	Biolegend
TCRβ	H57-597	Mouse	BV711	1:200	109243	Biolegend
TCRγδ	GL3	Mouse	Alexa Fluor 488	1:200	118128	Biolegend
pErk	197G2	Mouse/Human	none	1:200	4377	Cell Signaling
p65	532301	Mouse/Human	APC	1:20	IC5078A	R&D Systems
Vγ9	IMMU360	Human	FITC	1:100	IM1463	Beckman Coulter
Vδ2	B6	Human	PE	1:100	331408	Biolegend
Vδ1	TS8.2	Human	APC	1:100	17-5679-42	Thermo
Rabbit IgG	Polyclonal	Rabbit	Alexa Fluor 647	1:500	A-21245	Thermo Fisher

Appendix Table S4. Overview of small molecules used.

Small molecule	Dose(s)	Stock concentration	Manufacturer	Cat no.
GANT61	2.5, 5μM	5mM	APExBIO	A1615
PMA	1, 5, 10, 50ng/ml	1mg/ml	Sigma	P1585
Ionomycin	10, 50, 100, 1000ng/ml	1mg/ml	Sigma	I9657
U0126[23]	10μM	Reconstituted freshly	Cambridge Bioscience	SM106-5
BAPTA-AM[45]	1.25, 2.5, 5, 10, 25μM	Reconstituted freshly	Thermo Fisher	B1205
BAPTA Tetrasodium Salt	1.25mM	Reconstituted freshly	Thermo Fisher	B1214
EGTA	1.25mM	0.5M	Sigma	E4378
Cyclopamine	2.5μM	5mM	Selleckchem	S1146
TNP-ATP[46]	30μM	10mM	Tocris	2464/5
BCTC[85]	1μM	10mM	Tocris	3875/10
BTP2 (YM-58483)[77]	200nM	5mM	Tocris	3939/10
HC030031[48]	100μM	100mM	Tocris	2896/10
nifedipine[49]	10, 50, 100μM	100mM	Tocris	1075/100
FPL 64176	5, 10μM	25, 20mM	Tocris	1403
Thapsigargin	1μM	1mM	Thermo Fisher	T7458

Appendix Table S5. Taqman probes (Thermo Fisher) used for qRT-PCR

Probe	Cat no.	Exon Boundary
CD3ε	Mm00599684_g1	6-7
Tbp	Mm00446973_m1	4-5
Tbp	Mm00446971_m1	2-3
Ihh	Mm01259021_m1	1-2
Smo	Mm01162710_m1	8-9
Gli1	Mm00494654_m1	11-12
Gli1	Mm00494645_m1	2-3
Cacna1f	Mm01352612_m1	13-14
Cacna1d	Mm01209927_g1	48-49
Cacna1s	Mm00489257_m1	9-10
Cacna1c	Mm01188822_m1	26-27
b2m	Mm00437762_m1	1-2
CACNA1F	Hs00913730_m1	46-47
CACNA1D	Hs00167753_m1	46-47
CACNA1S	Hs00163885_m1	2-3
CACNA1C	Hs00167681_m1	3-4
TBP	Hs00427620_m1	2-3
GLI1	Hs01110766_m1	8-9
ACTB	Hs99999903_m1	1-1

Appendix Table S6. Antibodies used for immunofluorescence staining

Target	Fluorophore	Dilution	Cat no.	Host species	Manufacturer
$Ca_v1.4$	Primary	1:25-1:100	LS-C94032	Rabbit	Stratech
$Ca_v1.4$	Primary	1:25	NBP1-30667	Rabbit	Novus Biological
CD3ε	Primary/BV510	1:50	300448	Mouse	BD
AP1G1	Primary	1:1000	A4200	Mouse	Sigma
VPS35	Primary	1:1000	sc-374372	Mouse	Santa Cruz
Phalloidin	AF568	1:150	A12380	N/A	Thermo Fisher
Phalloidin	AF647	1:100	A30107	N/A	Thermo Fisher
Rabbit IgG	AF488	1:400	A11034	Goat	Thermo Fisher
Mouse IgG	AF647	1:400	A31571	Donkey	Thermo Fisher

Appendix Table S7. Antibodies used for Western Blotting

Target	Clone	Reactivity	Type	Dilution	Cat no.	Supplier
Gli1	388516	Mouse/Human	Primary	1:1’000	MAB3324	Biotechne
α-tubulin	Monoclonal	Mouse anti-human/mouse	Primary	1:2’000	3873S	Cell Signaling
β-tubulin	Polyclonal	Rabbit anti-human/rat/mouse	Primary	1:2’000	2146	Cell Signaling
Rat IgG	Polyclonal	Goat anti-rat	Secondary (HRP conj.)	1:3’000	7077S	Cell Signaling
Rabbit IgG	Polyclonal	Goat anti-rabbit	Secondary (HRP conj.)	1:10’000	P0448	Dako

Appendix Table S8. List of Gli1 binding sites used in analysis of RNA-Seq data

***Gli1* binding sequences**
GACCACCCA
AACCACCCA
GAACACCCA
GACCACACA
GACCACCAA
GACCCCCCA
GGCCACCCA
GACCGCCCA
TACCACCCA
GACCTCCCA
GACCACCTA

Appendix Table S9. List of transcripts upregulated at 24h by FPL 641761 with Gli1 binding sites in their promoter regions

Gli1 Binding Site	Gene	Match Count	Match Strand
GACCACCCA	*Leprotl1*	1	Forward
AACCACCCA	*Dhx33*	1	Forward
GAACACCCA	*Septin7*	1	Forward
GAACACCCA	*Prag1*	1	Forward
GACCCCCCA	*Polr3e*	1	Forward
GACCCCCCA	*Mbd3*	1	Forward
GGCCACCCA	*Fasn*	1	Forward
GGCCACCCA	*Chd1*	1	Forward
GGCCACCCA	*Il6st*	1	Forward
GACCTCCCA	*Cd2*	1	Forward
GACCACCTA	*Rps18*	1	Forward
GAACACCCA	*Atp1a1*	1	Reverse
GACCACCAA	*Cux1*	1	Reverse
GACCCCCCA	*Fam136a*	1	Reverse
GGCCACCCA	*Rif1*	1	Reverse
GACCGCCCA	*Pdzd8*	1	Reverse
TACCACCCA	*Dnaja3*	1	Reverse
TACCACCCA	*Slc29a1*	1	Reverse
TACCACCCA	*Dnmt1*	1	Reverse
GACCTCCCA	*Mthfd2*	1	Reverse
GACCTCCCA	*Chd1*	1	Reverse
GACCACCTA	*Eef2*	1	Reverse

Appendix references

Winklmayr, M. *et al.* Non-consensus GLI binding sites in Hedgehog target gene regulation. *BMC Mol Biol* **11**, 2 (2010).
