# Supplementary figures and images for: L-type voltage-gated Ca2+ channels control T cell killing via non-canonical Hedgehog signalling
Source: EMBO Rep. 2026 Jun 8;27(13):3689–730. doi: 10.1038/s44319-026-00810-8 (PMC13354577; doi:10.1038/s44319-026-00810-8)

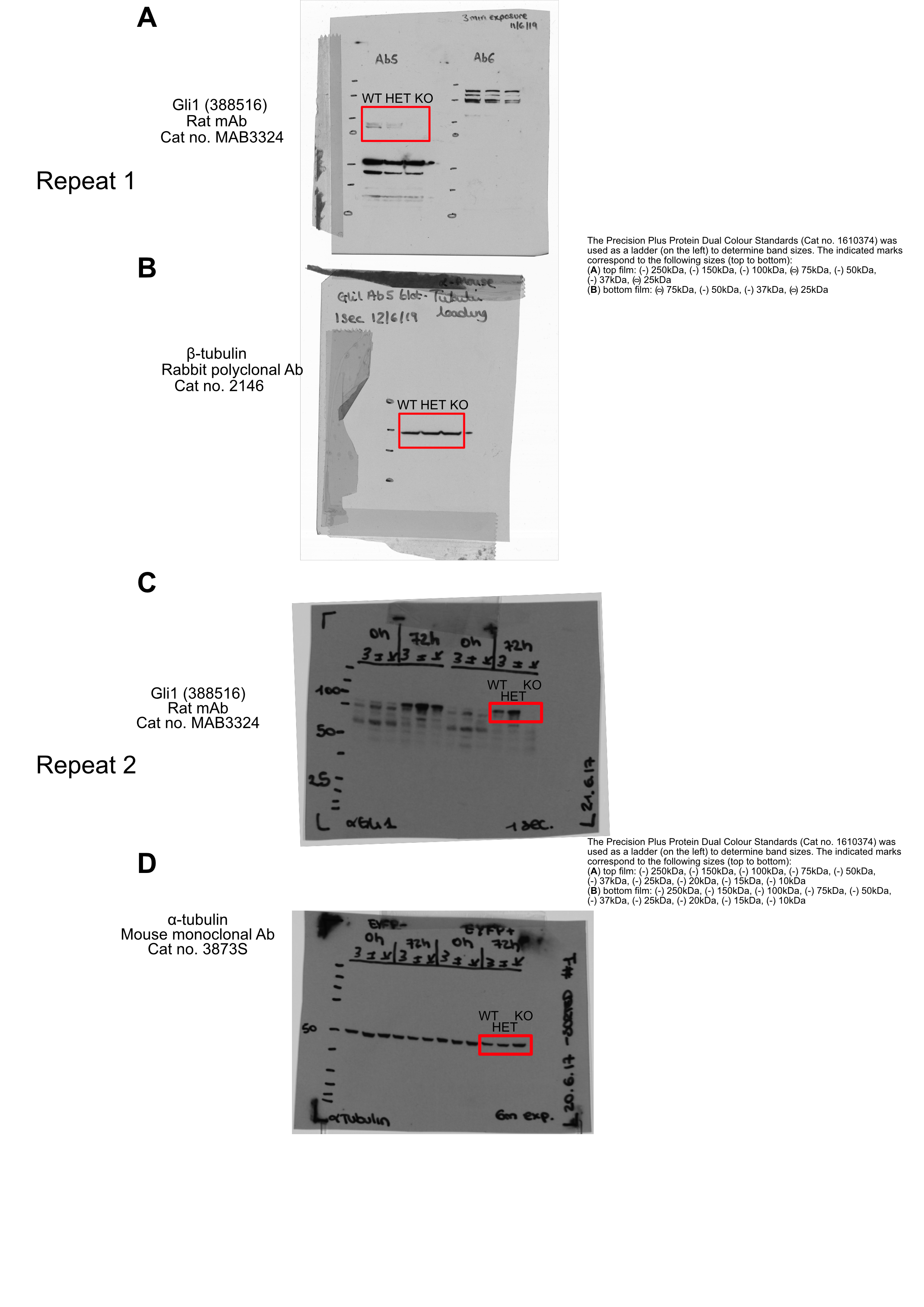

Supplement: Supplementary file 3 — Source data Fig. 1 [file 44319_2026_810_MOESM3_ESM.zip › Figure_1/1A/Western_Gli1_+tubulin_n=2.tiff]

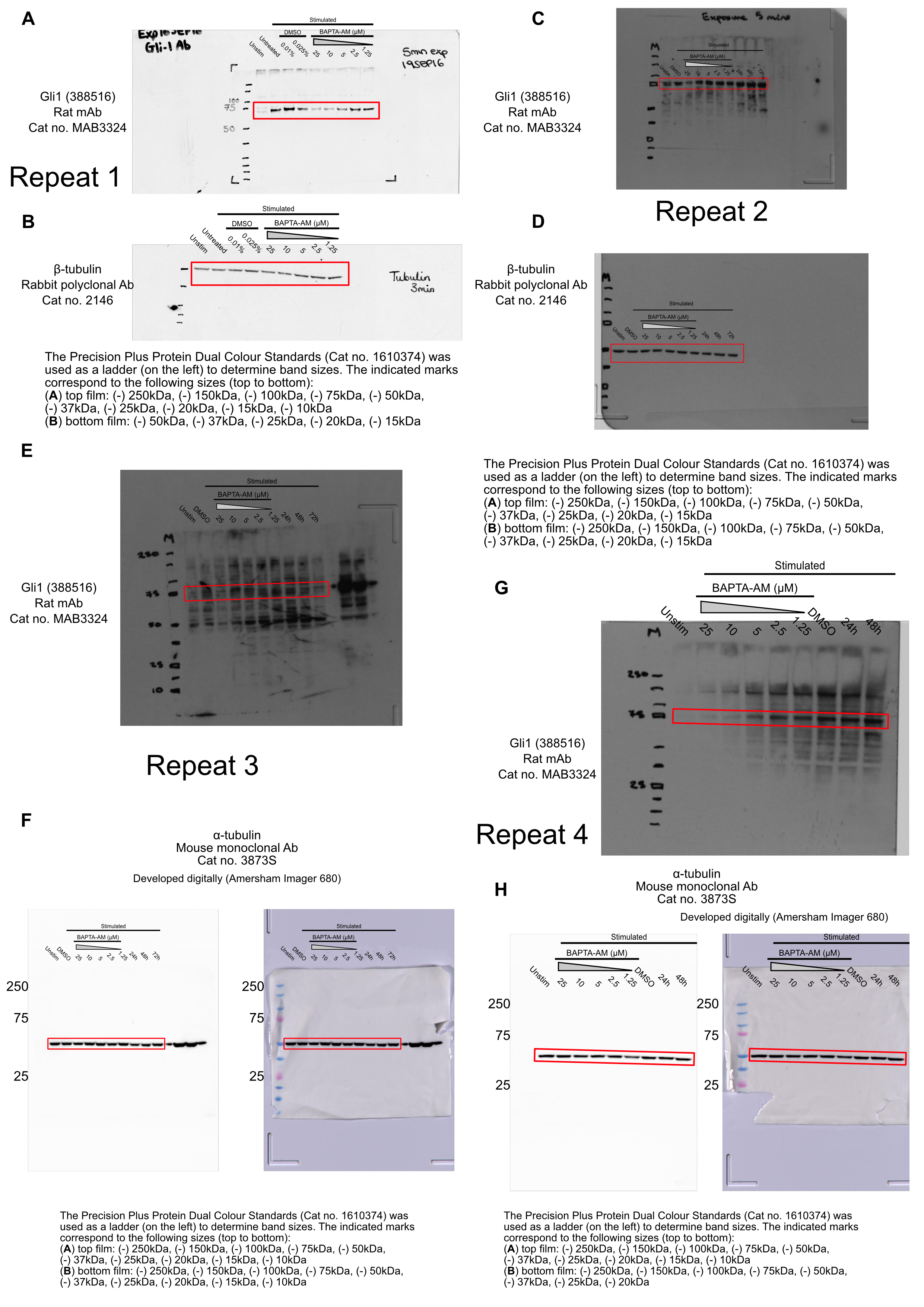

Supplement: Supplementary file 5 — Source data Fig. 3 [file 44319_2026_810_MOESM5_ESM.zip › Figure_3/3A/Western_Gli1+tubulin_n=4.tiff]

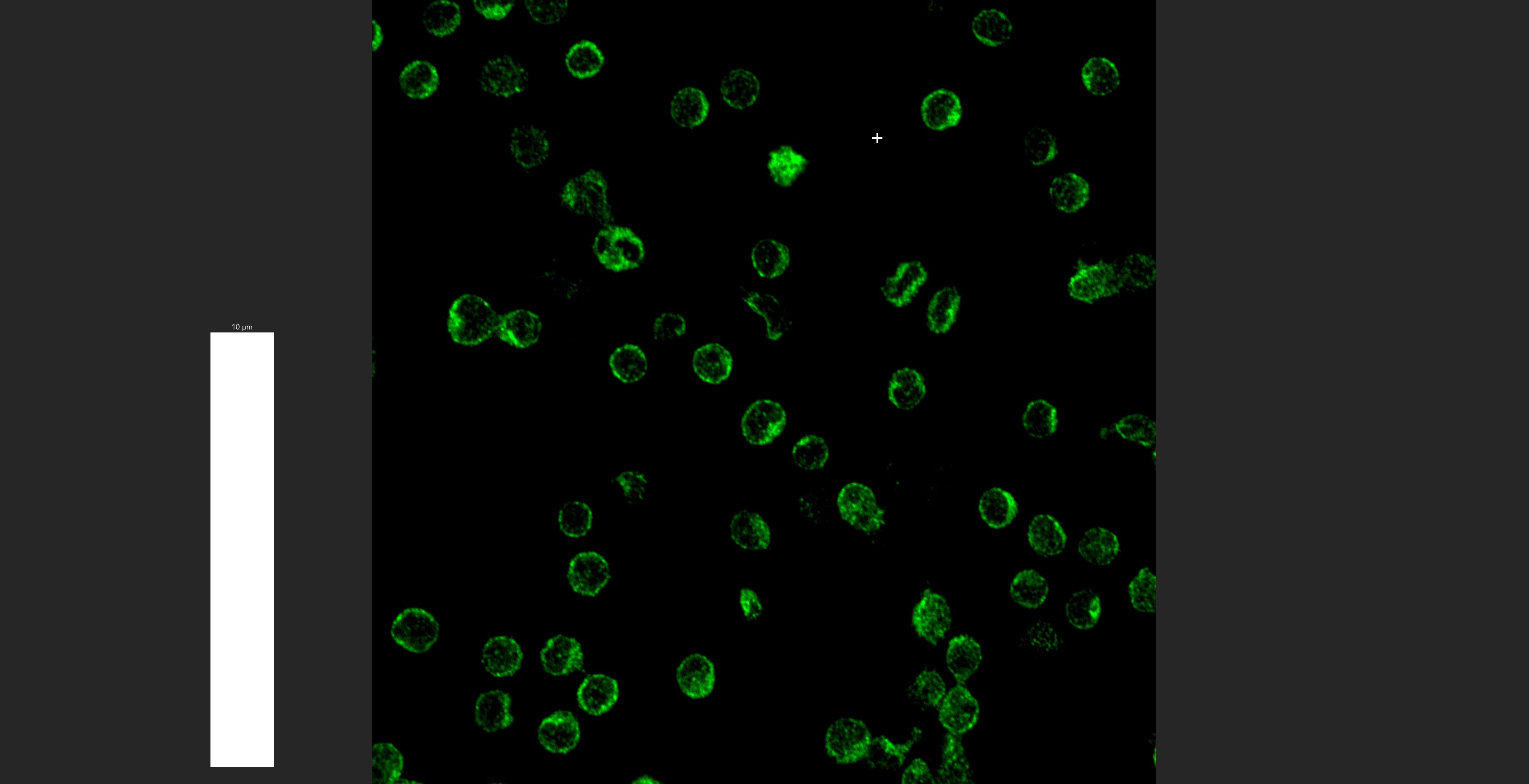

Supplement: Supplementary file 8 — Source data Fig. 7 [file 44319_2026_810_MOESM8_ESM.zip › Figure_7/7A/IF_Cav1_4.tif]

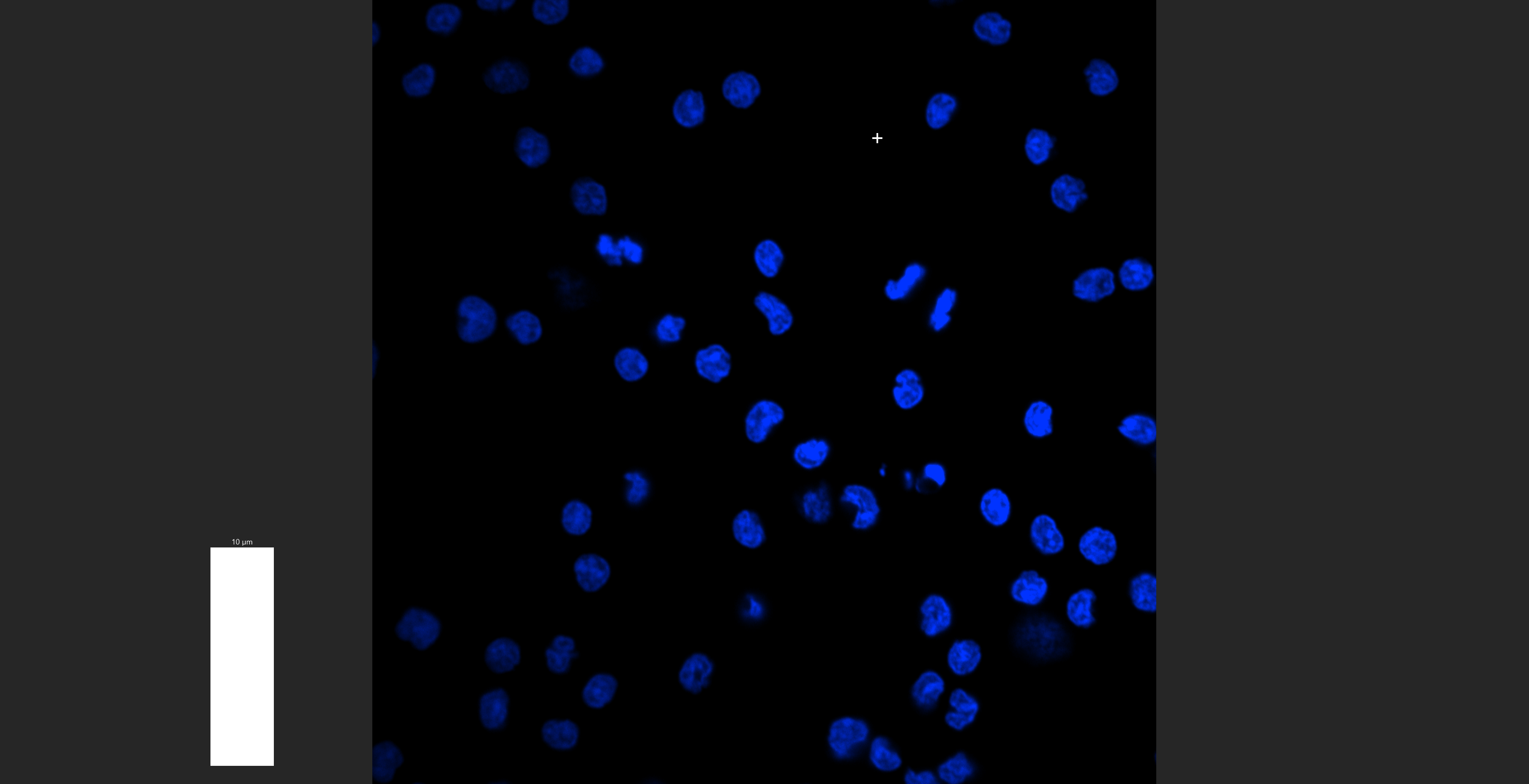

Supplement: Supplementary file 8 — Source data Fig. 7 [file 44319_2026_810_MOESM8_ESM.zip › Figure_7/7A/IF_DAPI.tif]

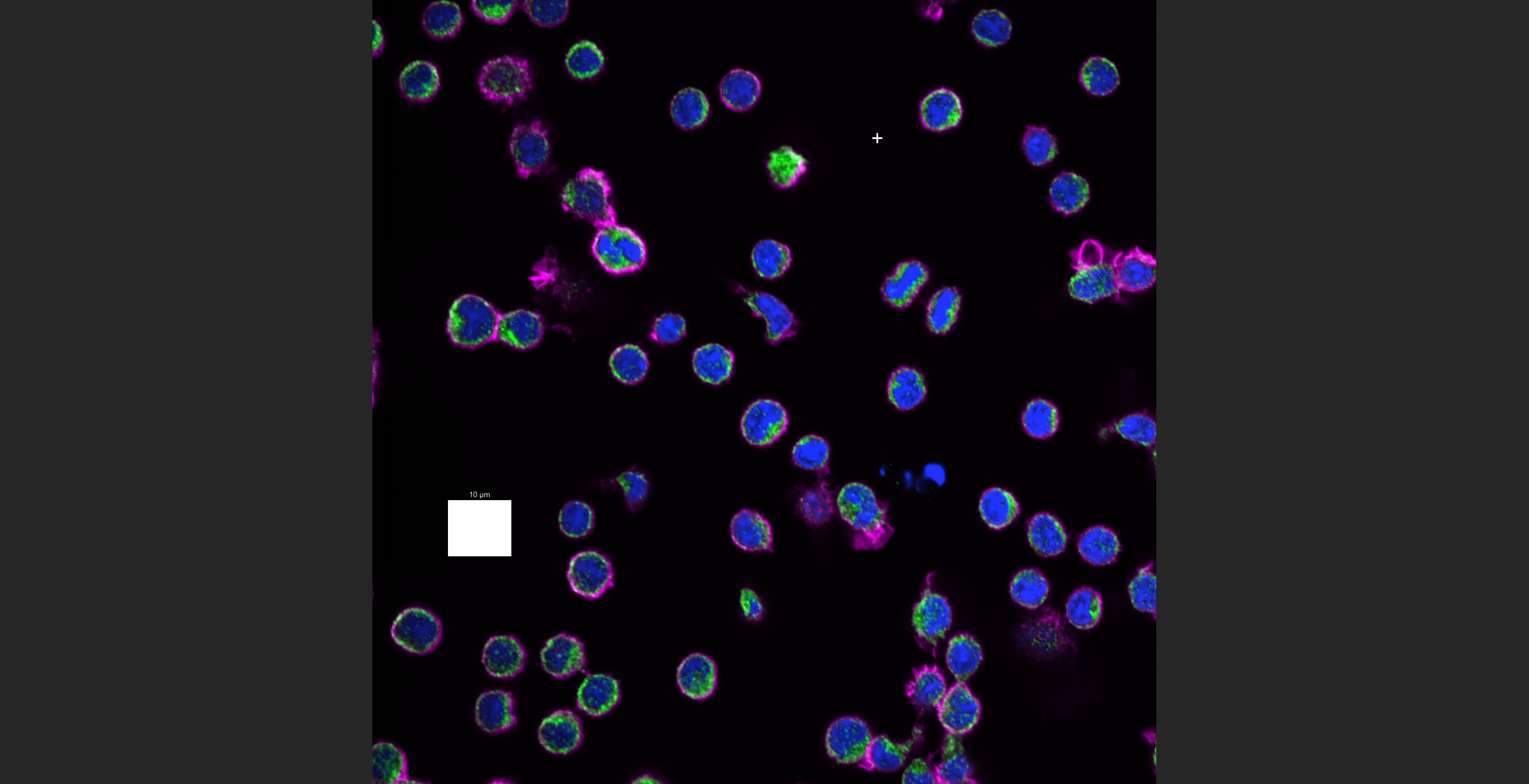

Supplement: Supplementary file 8 — Source data Fig. 7 [file 44319_2026_810_MOESM8_ESM.zip › Figure_7/7A/IF_merge.tif]

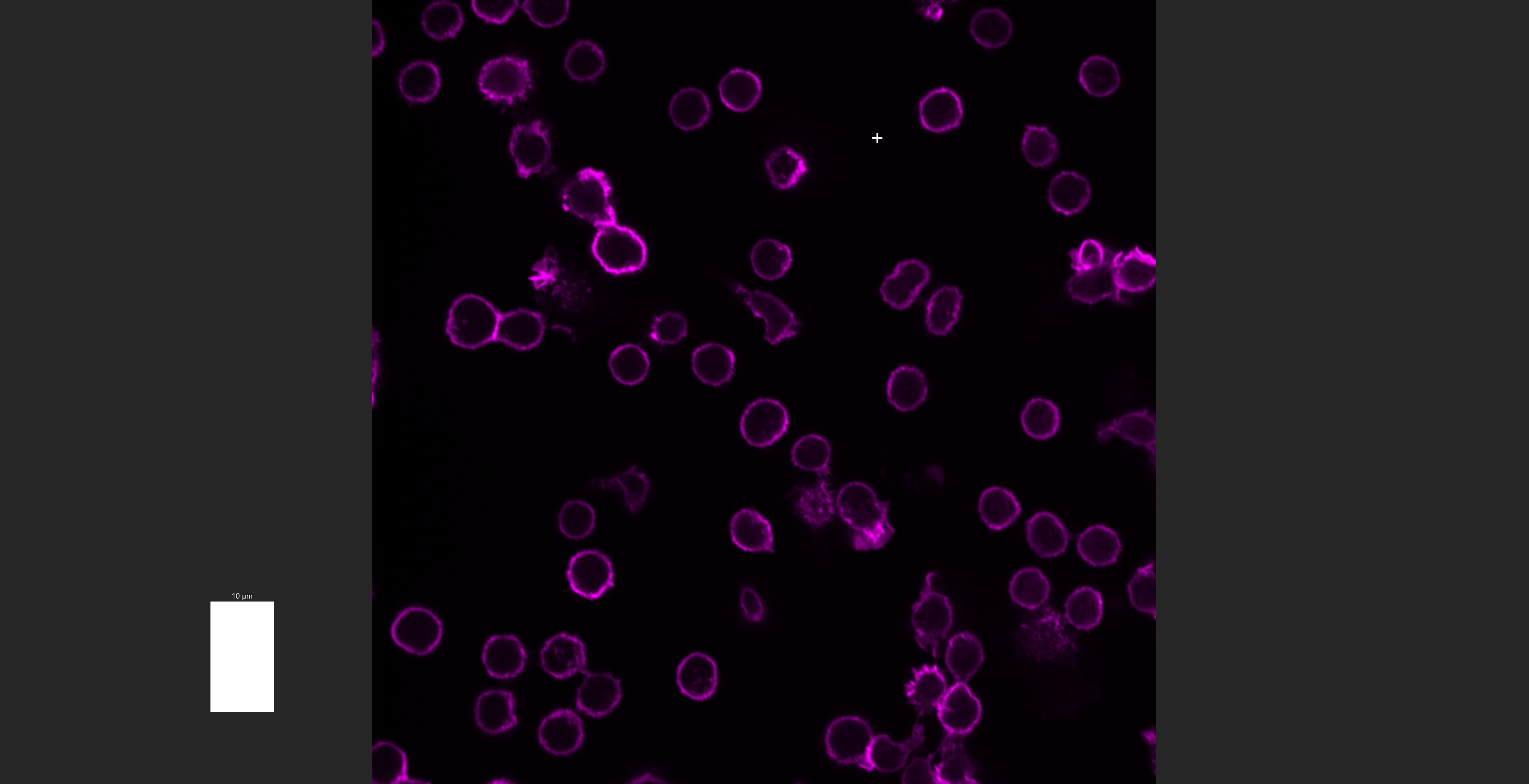

Supplement: Supplementary file 8 — Source data Fig. 7 [file 44319_2026_810_MOESM8_ESM.zip › Figure_7/7A/IF_phalloidin.tif]

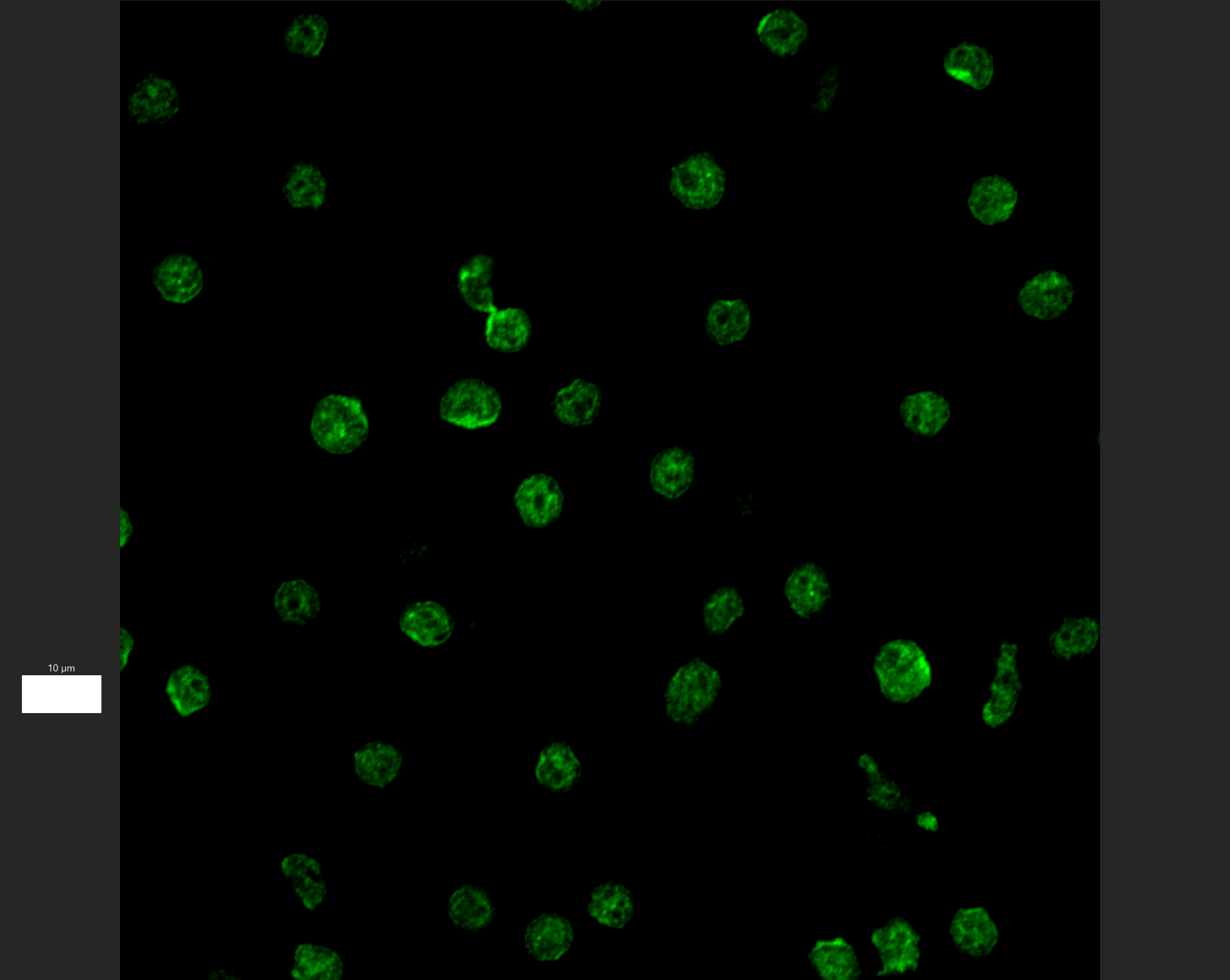

Supplement: Supplementary file 8 — Source data Fig. 7 [file 44319_2026_810_MOESM8_ESM.zip › Figure_7/7B/IF_Cav1_4.tif]

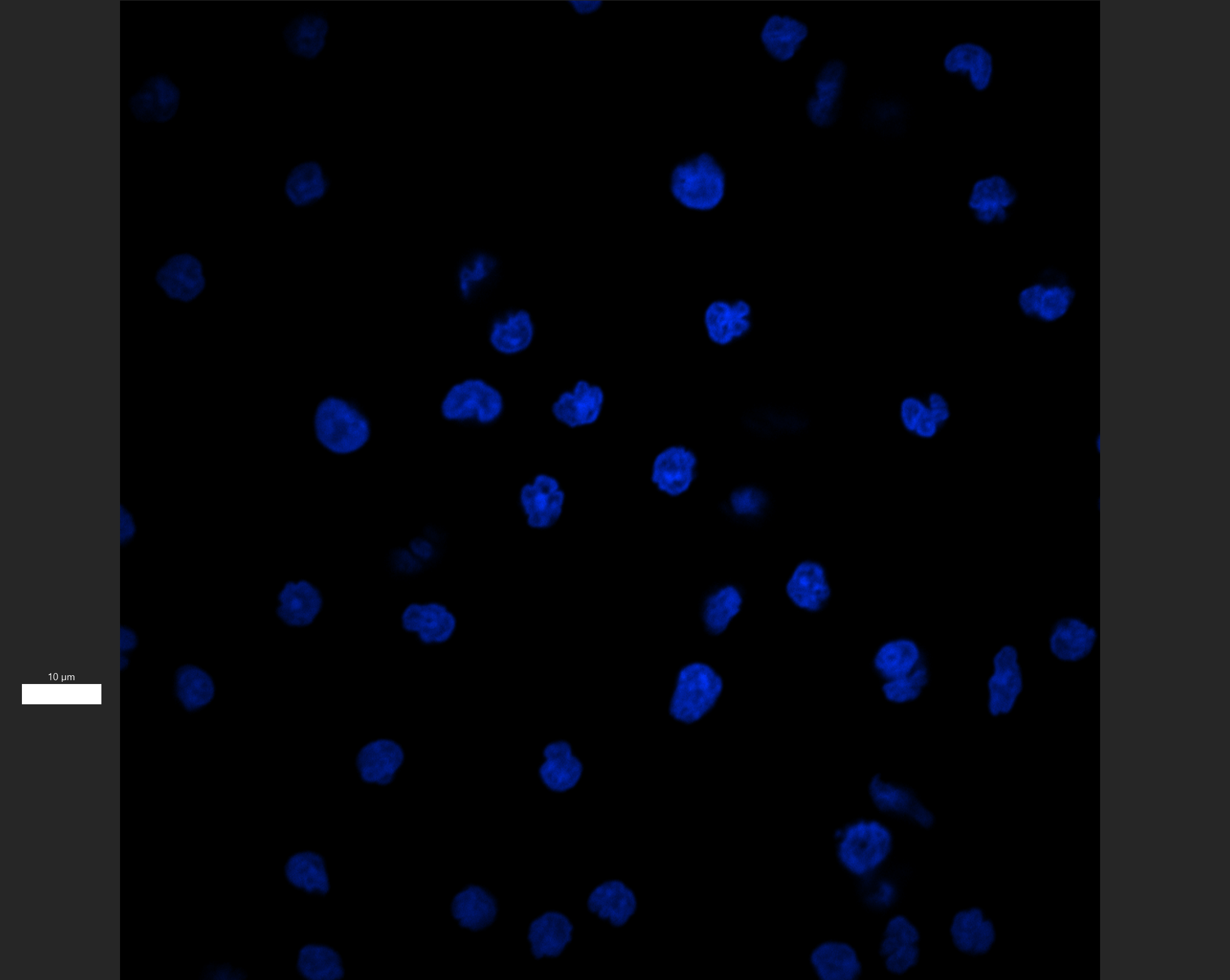

Supplement: Supplementary file 8 — Source data Fig. 7 [file 44319_2026_810_MOESM8_ESM.zip › Figure_7/7B/IF_DAPI.tif]

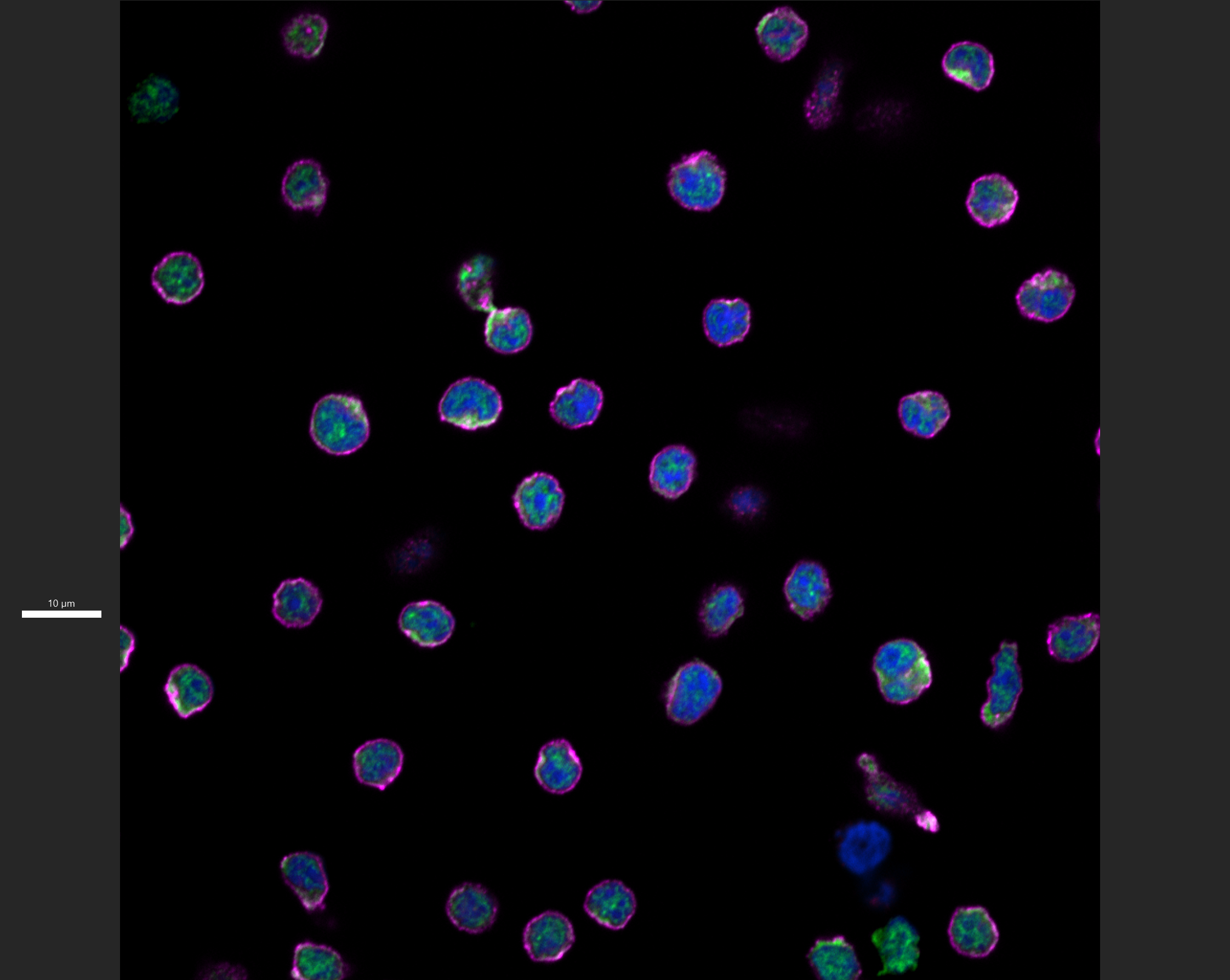

Supplement: Supplementary file 8 — Source data Fig. 7 [file 44319_2026_810_MOESM8_ESM.zip › Figure_7/7B/IF_merge.tif]

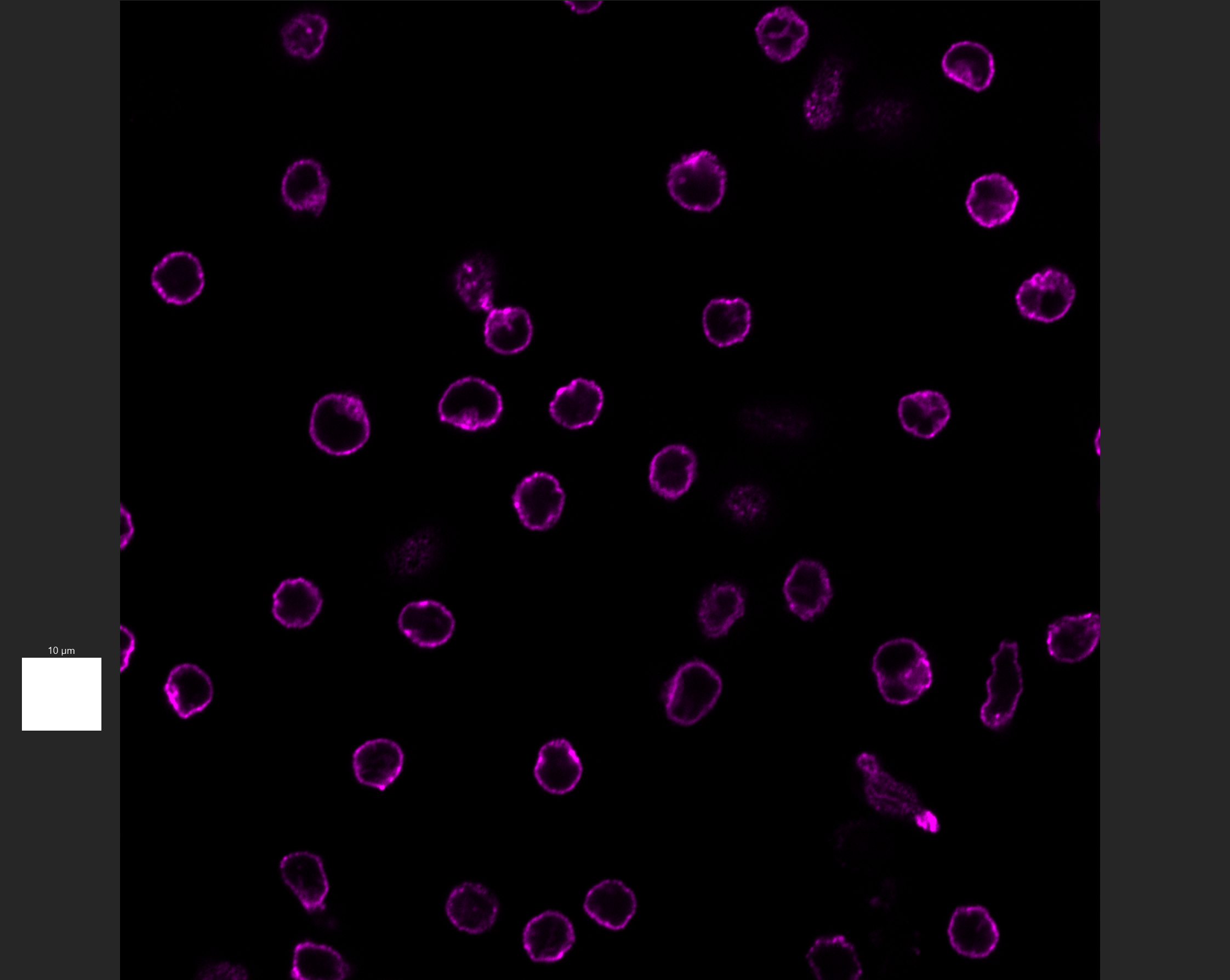

Supplement: Supplementary file 8 — Source data Fig. 7 [file 44319_2026_810_MOESM8_ESM.zip › Figure_7/7B/IF_TCR.tif]

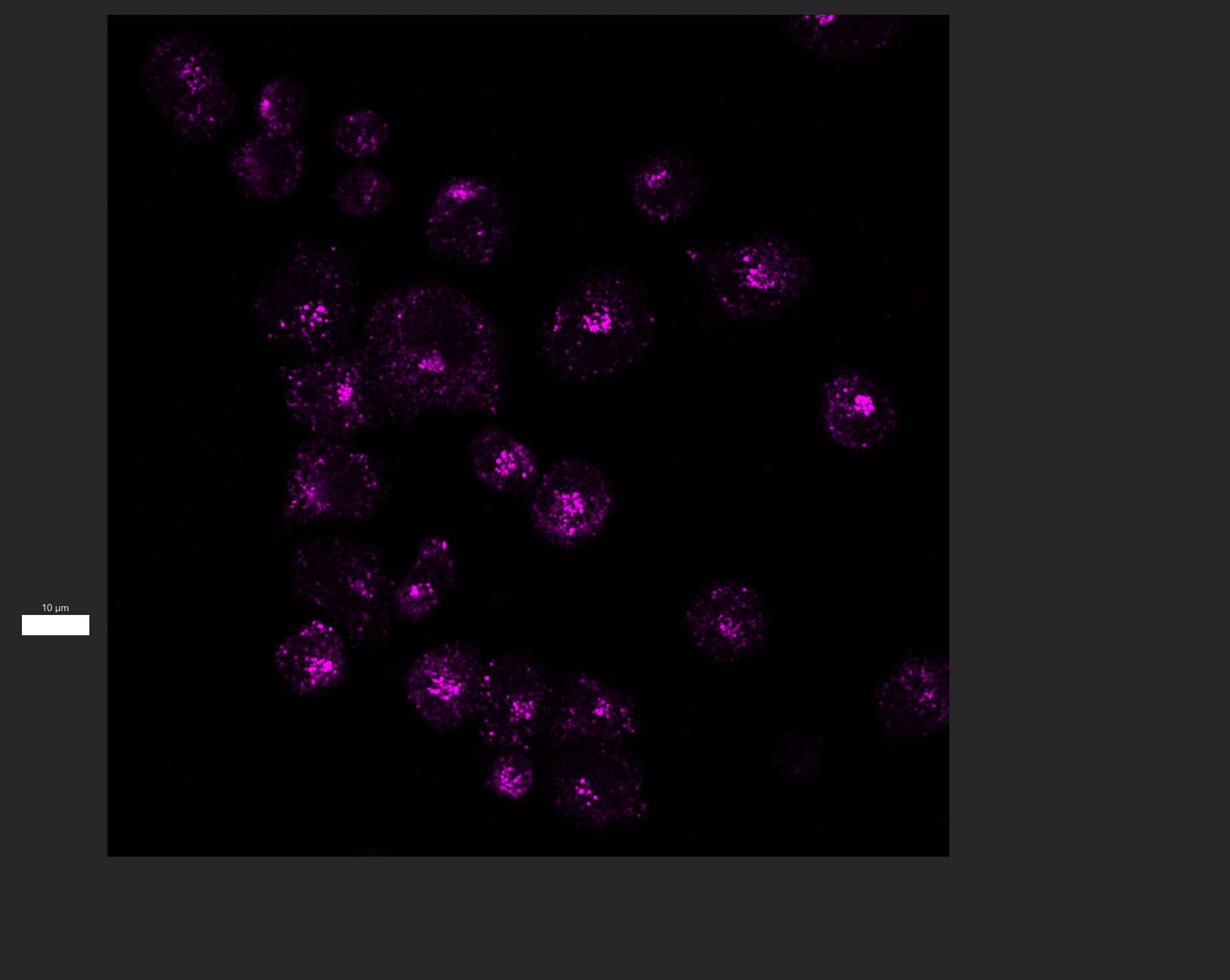

Supplement: Supplementary file 8 — Source data Fig. 7 [file 44319_2026_810_MOESM8_ESM.zip › Figure_7/7C/IF_AP1G1.tif]

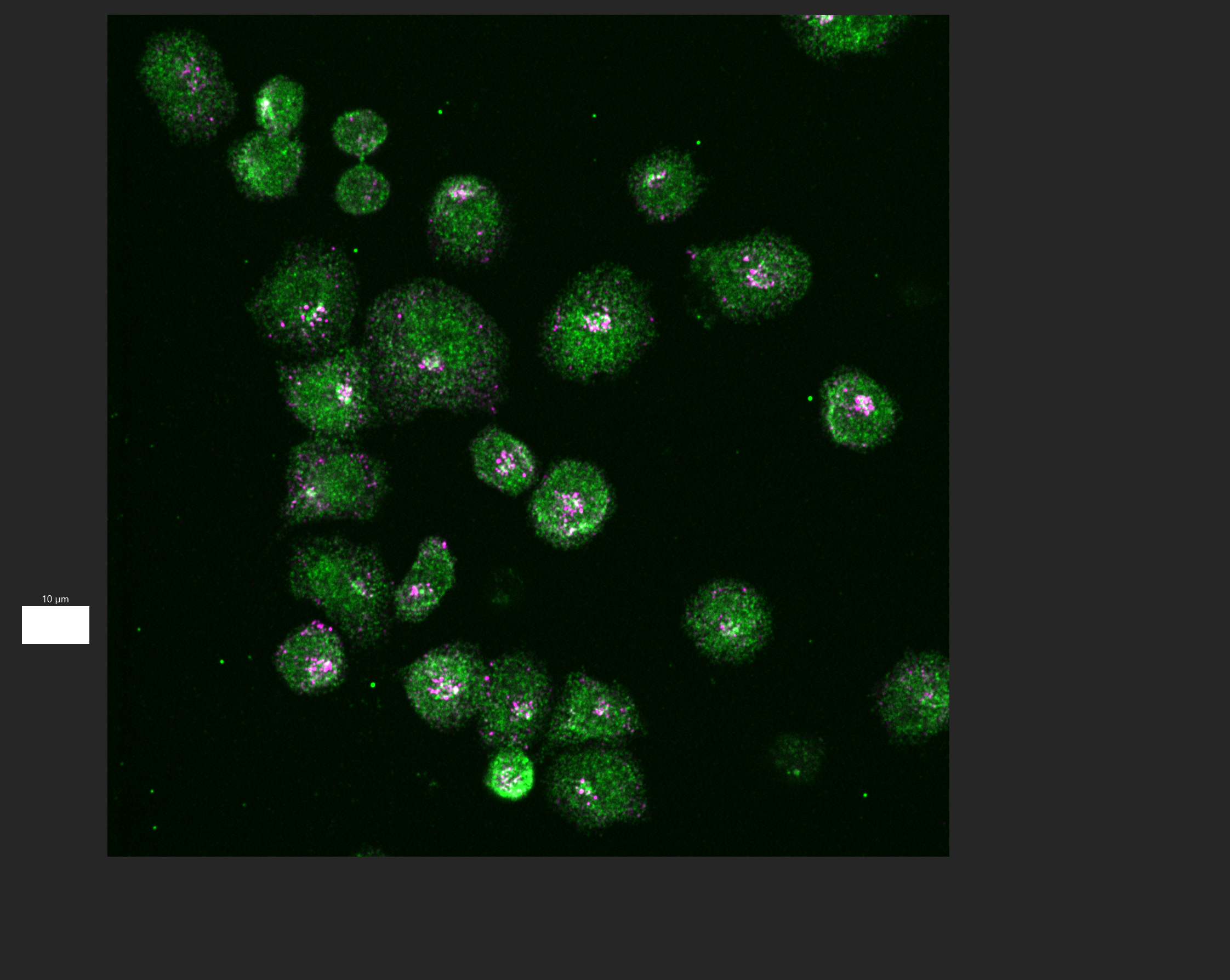

Supplement: Supplementary file 8 — Source data Fig. 7 [file 44319_2026_810_MOESM8_ESM.zip › Figure_7/7C/IF_Cav1_4+AP1G1.tif]

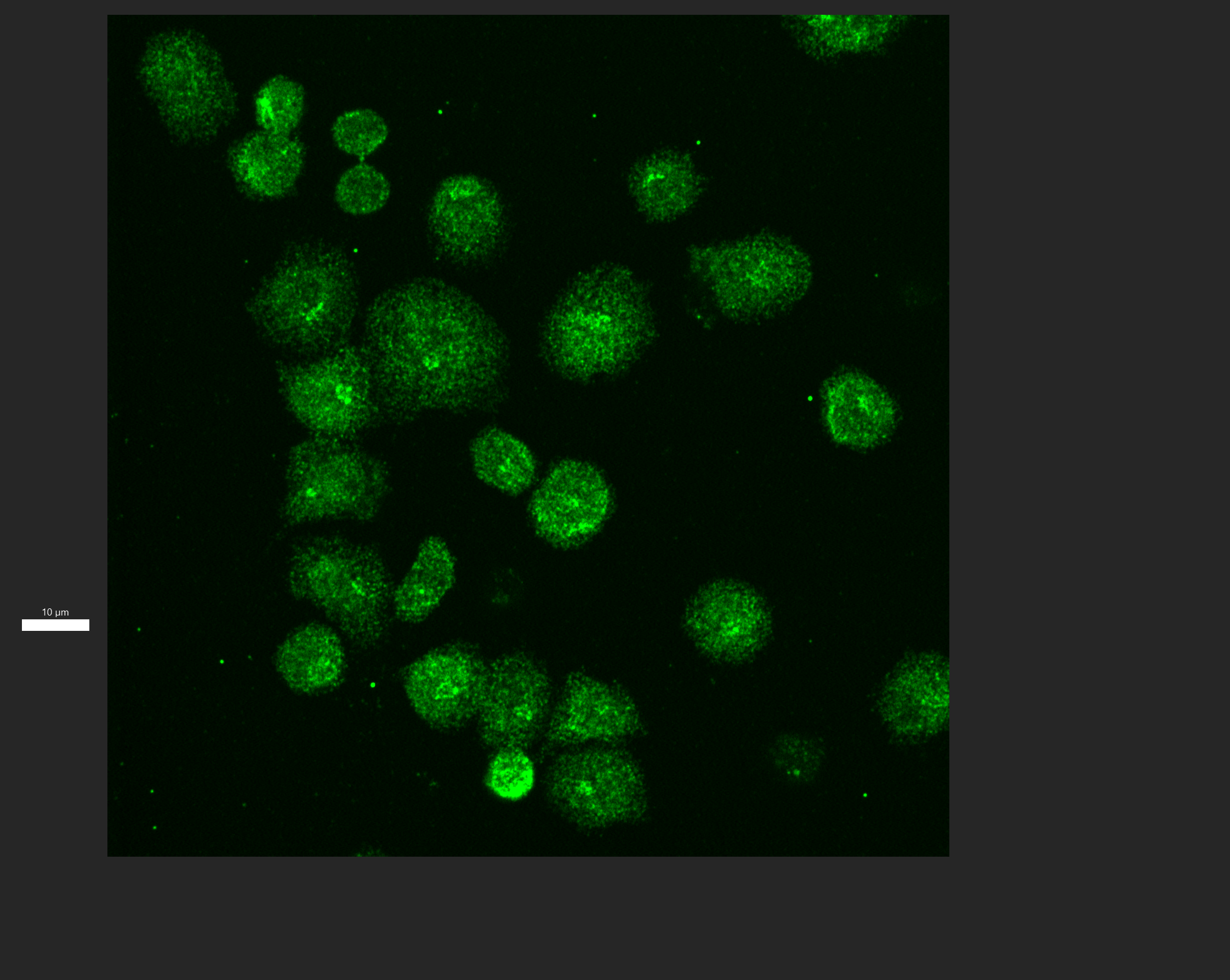

Supplement: Supplementary file 8 — Source data Fig. 7 [file 44319_2026_810_MOESM8_ESM.zip › Figure_7/7C/IF_Cav1_4.tif]

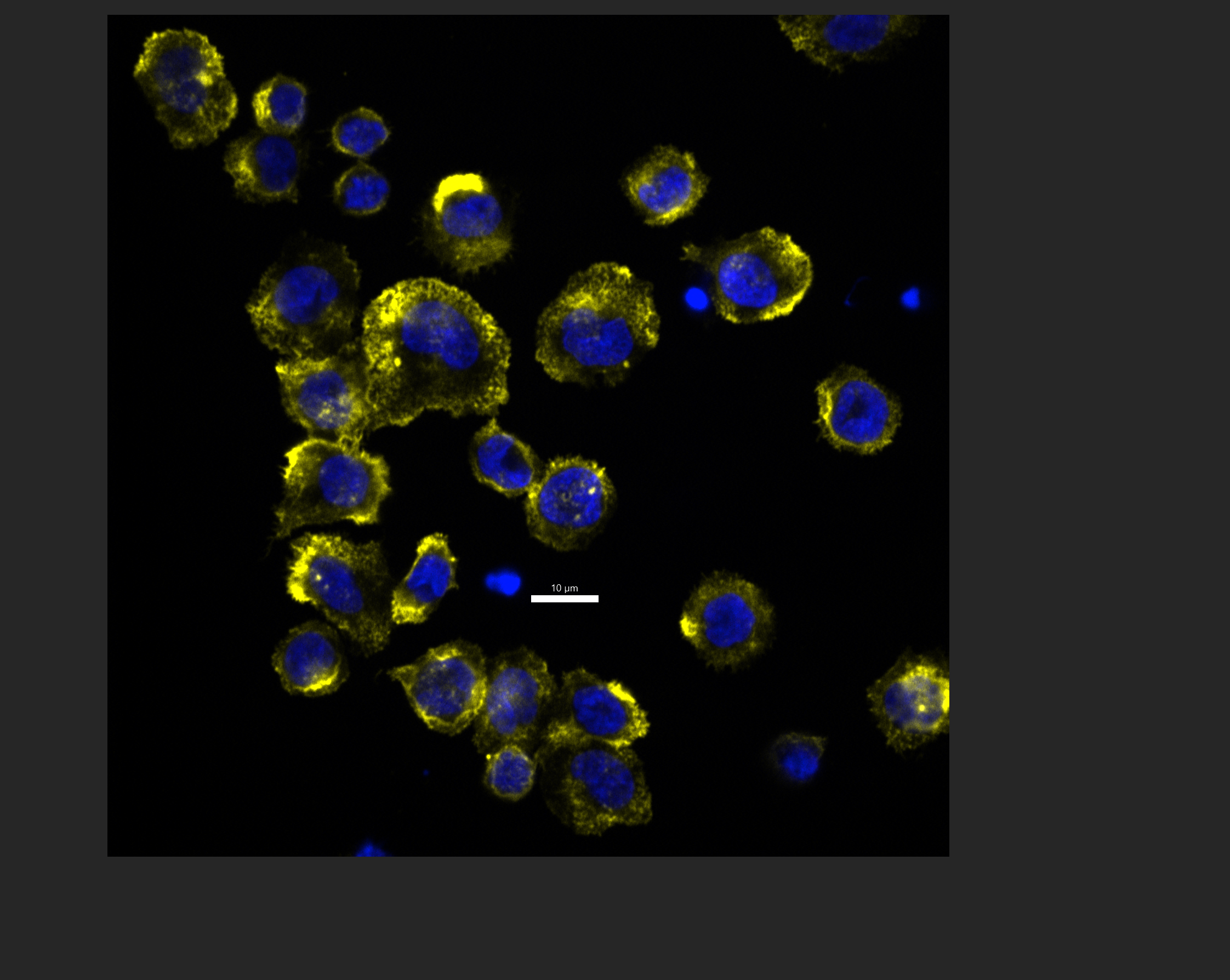

Supplement: Supplementary file 8 — Source data Fig. 7 [file 44319_2026_810_MOESM8_ESM.zip › Figure_7/7C/IF_phalloidin+DAPI.tif]

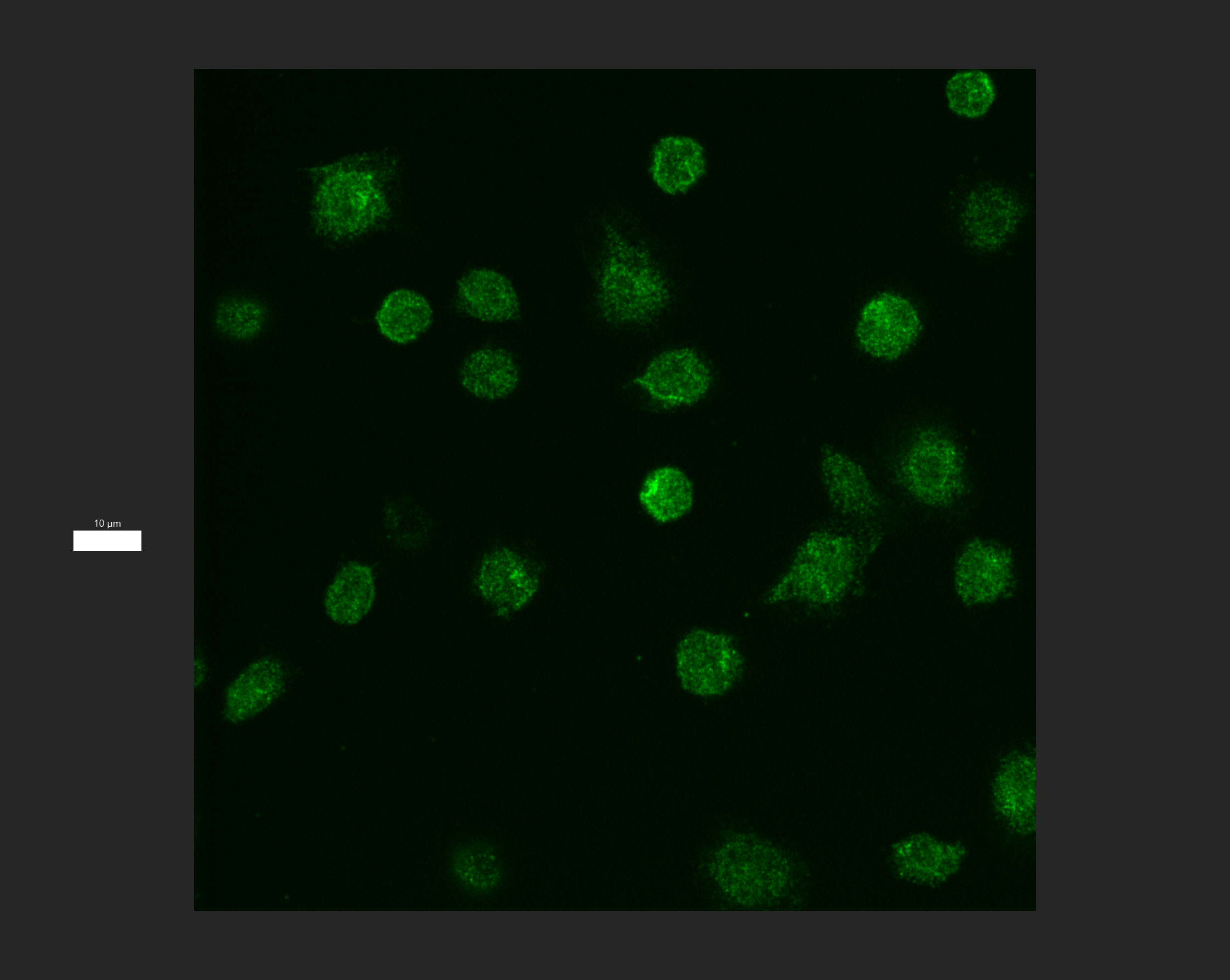

Supplement: Supplementary file 8 — Source data Fig. 7 [file 44319_2026_810_MOESM8_ESM.zip › Figure_7/7D/IF_Cav1_4.tif]

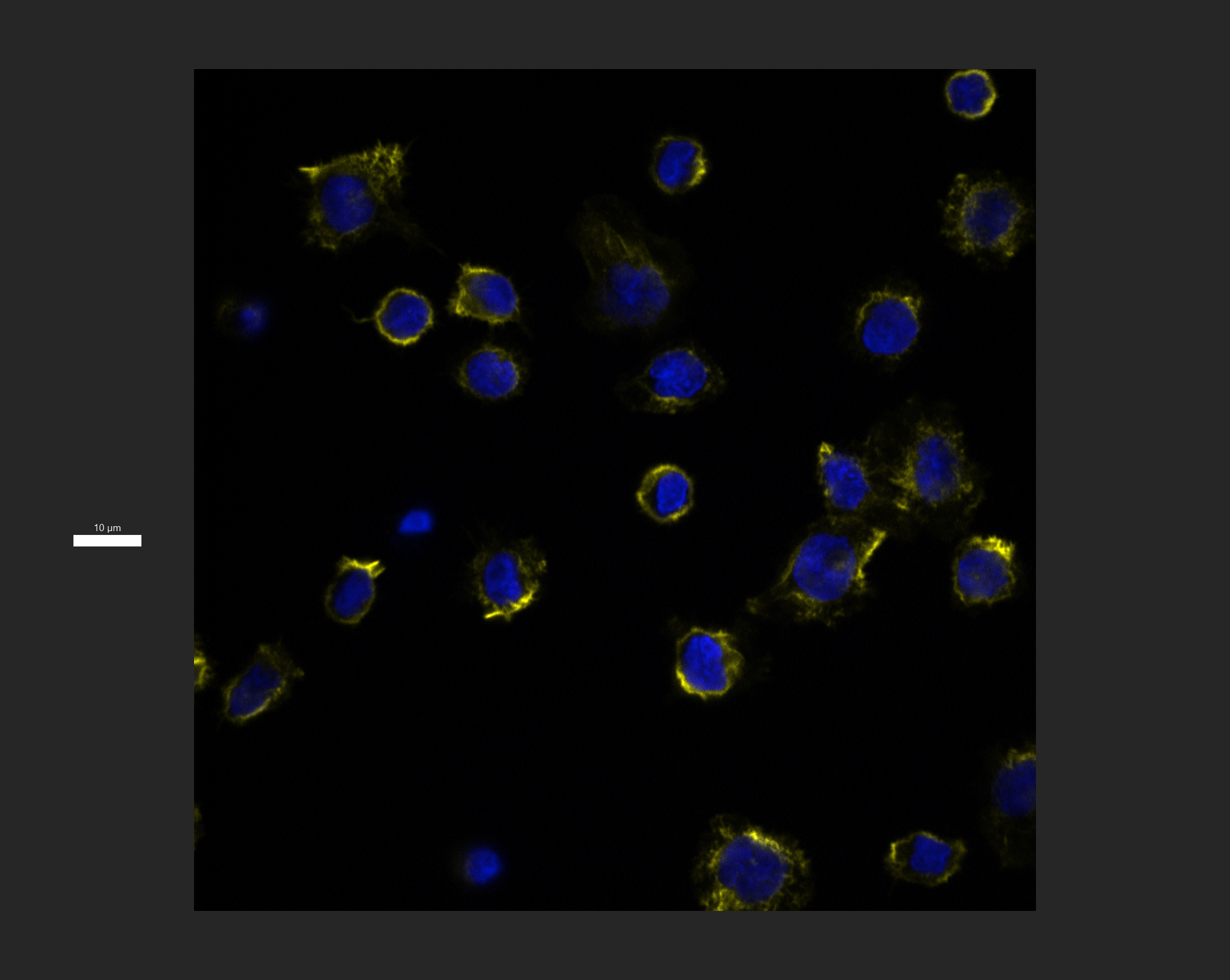

Supplement: Supplementary file 8 — Source data Fig. 7 [file 44319_2026_810_MOESM8_ESM.zip › Figure_7/7D/IF_phalloidin+DAPI.tif]

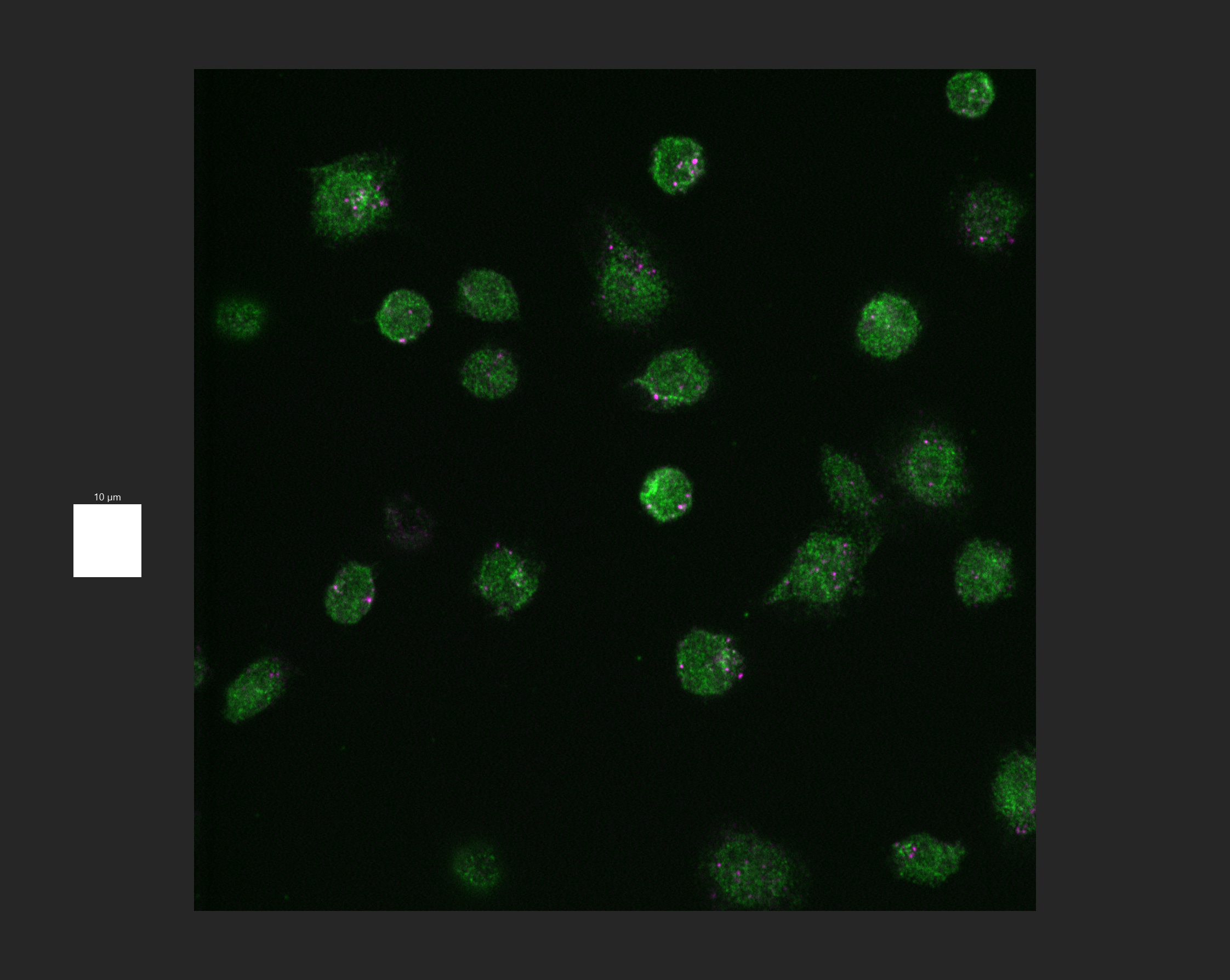

Supplement: Supplementary file 8 — Source data Fig. 7 [file 44319_2026_810_MOESM8_ESM.zip › Figure_7/7D/IF_VPS35+Cav1_4.tif]

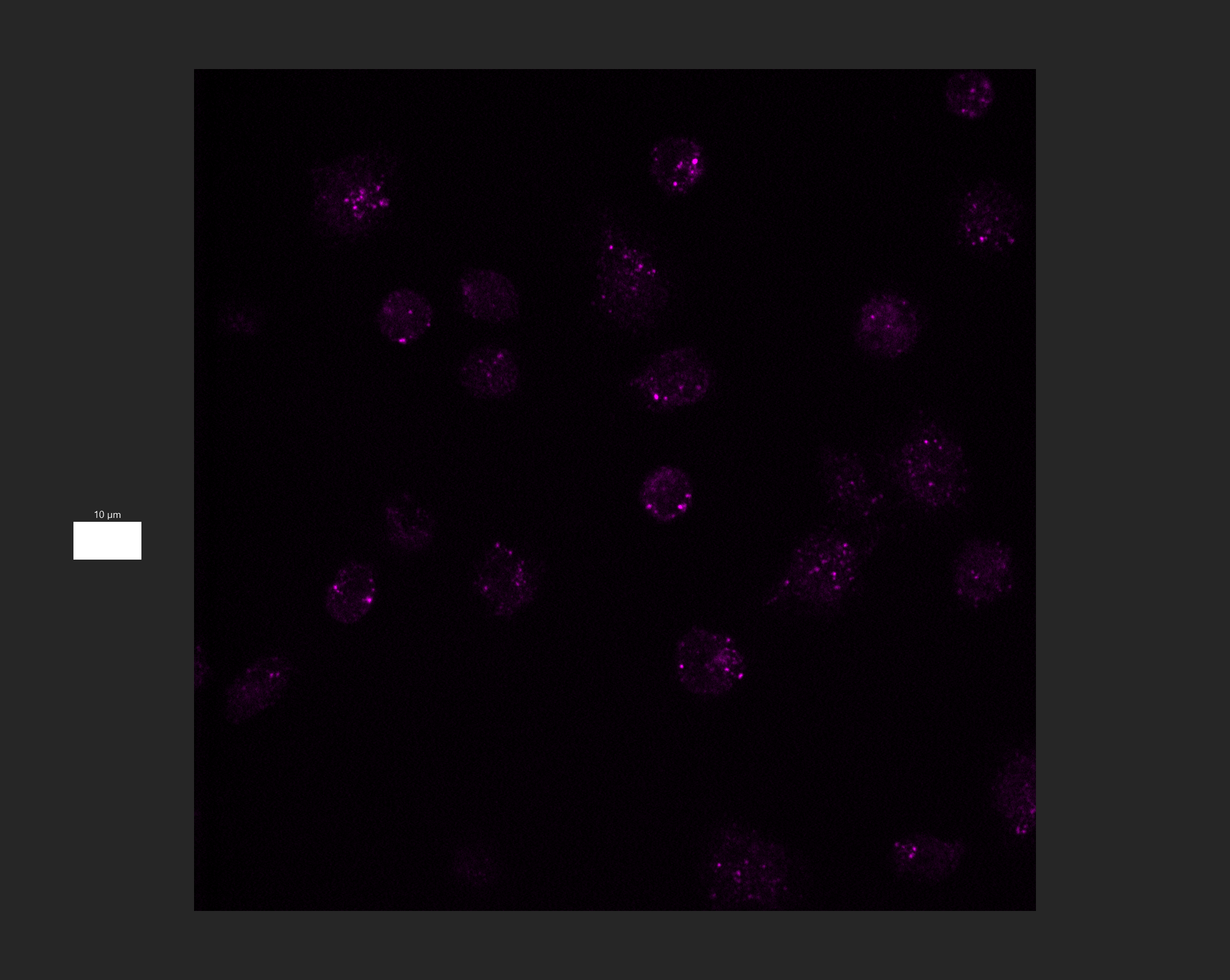

Supplement: Supplementary file 8 — Source data Fig. 7 [file 44319_2026_810_MOESM8_ESM.zip › Figure_7/7D/IF_VPS35.tif]

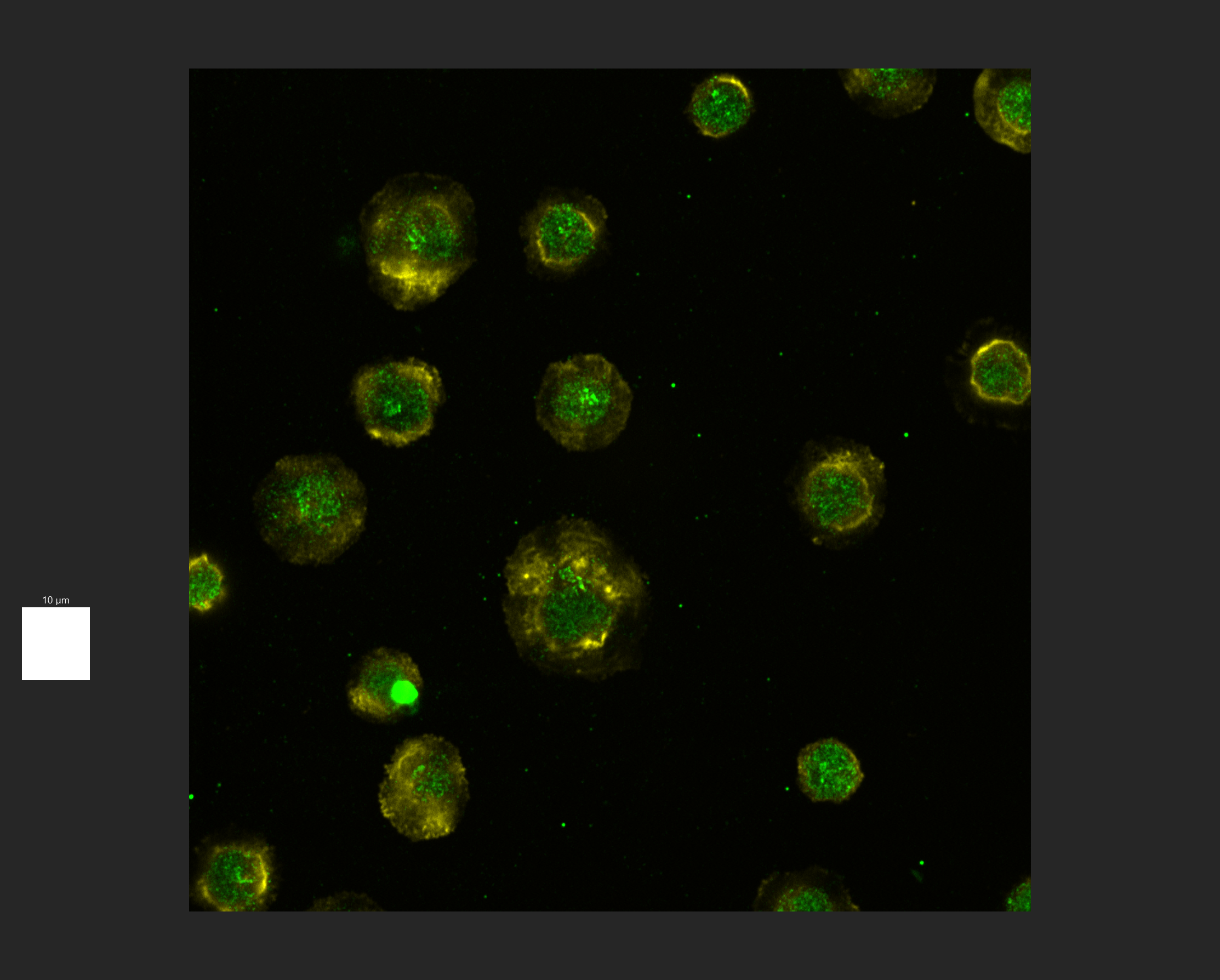

Supplement: Supplementary file 8 — Source data Fig. 7 [file 44319_2026_810_MOESM8_ESM.zip › Figure_7/7E/IF_Cav1_4+phalloidin.tif]

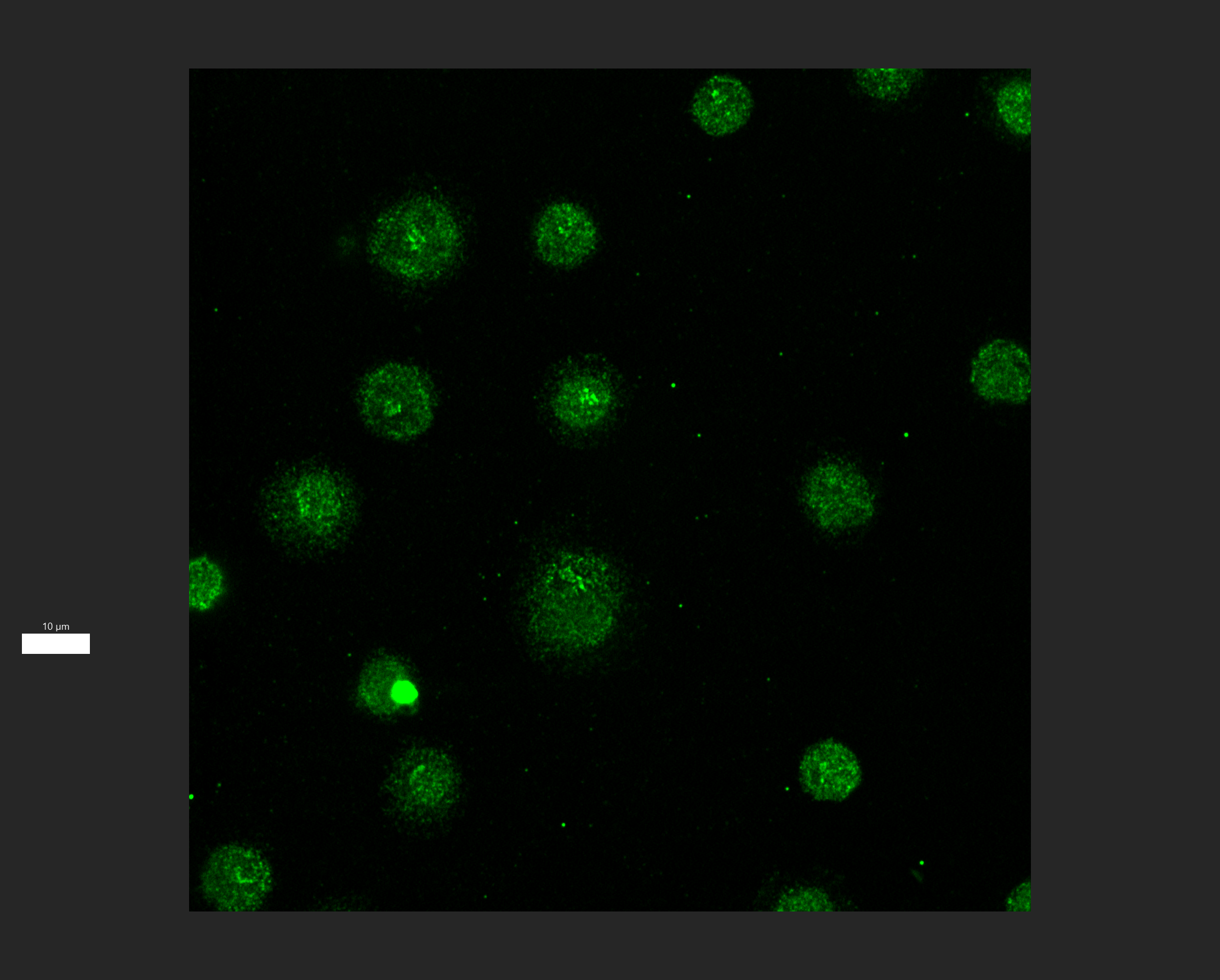

Supplement: Supplementary file 8 — Source data Fig. 7 [file 44319_2026_810_MOESM8_ESM.zip › Figure_7/7E/IF_Cav1_4.tif]

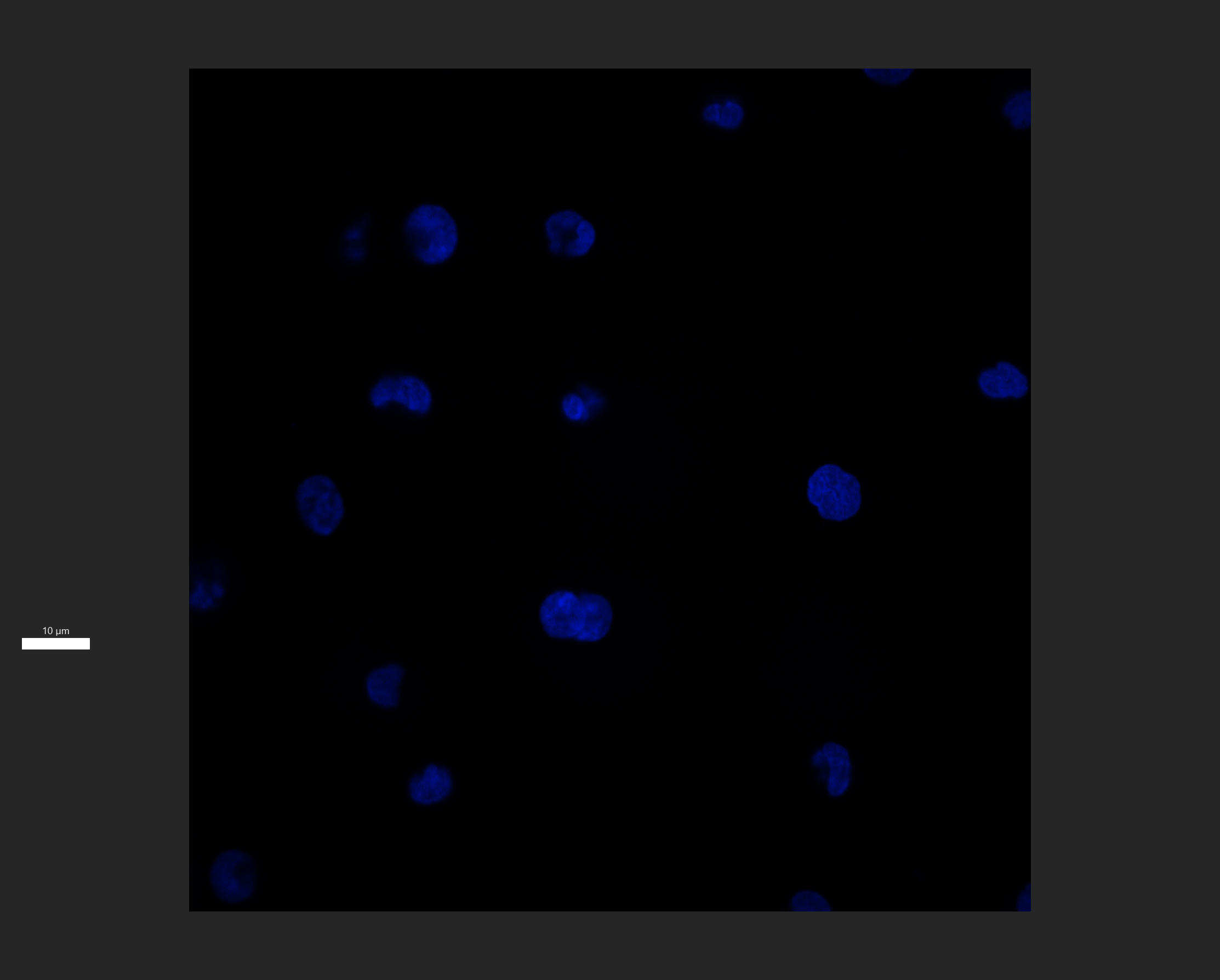

Supplement: Supplementary file 8 — Source data Fig. 7 [file 44319_2026_810_MOESM8_ESM.zip › Figure_7/7E/IF_DAPI.tif]

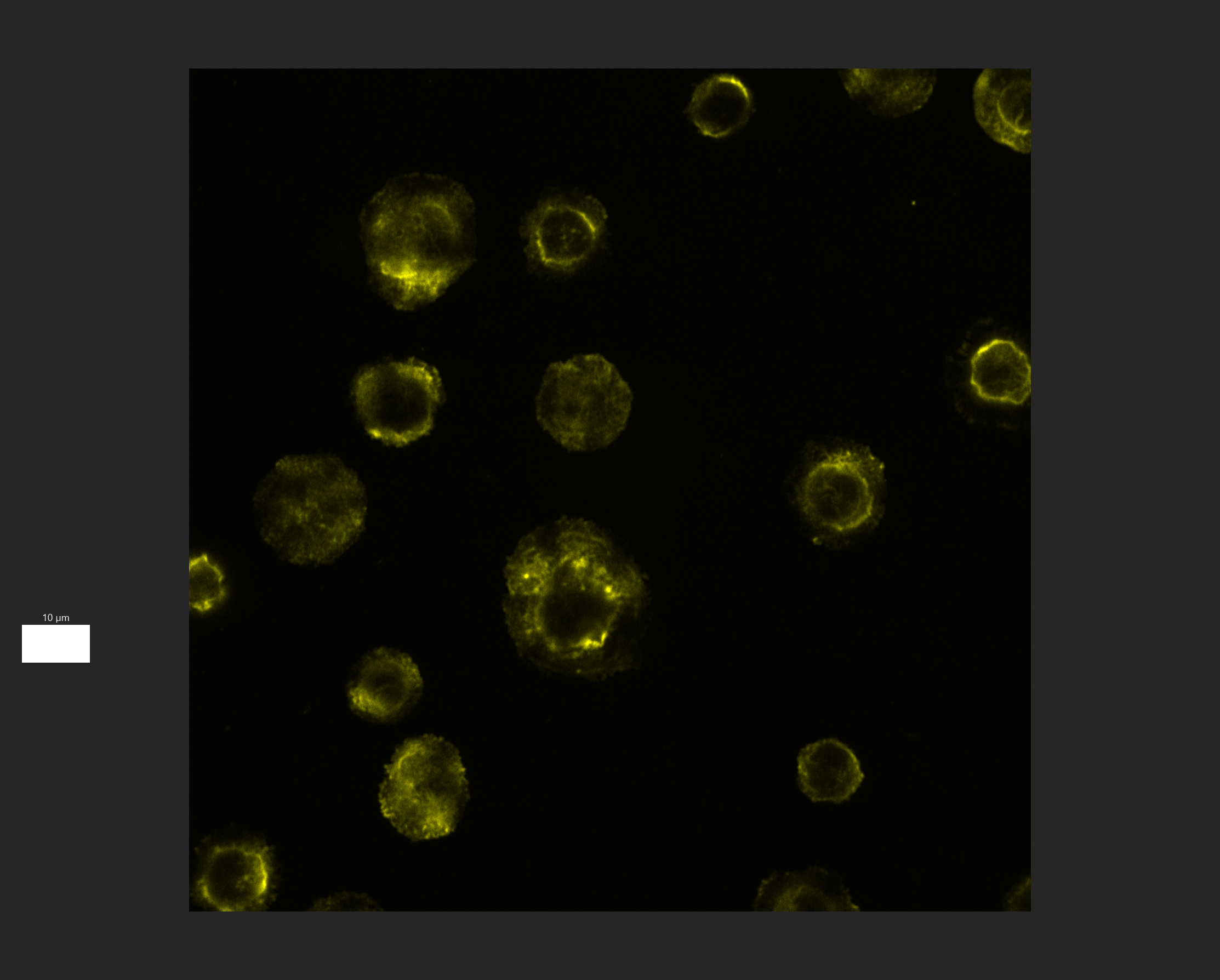

Supplement: Supplementary file 8 — Source data Fig. 7 [file 44319_2026_810_MOESM8_ESM.zip › Figure_7/7E/IF_phalloidin.tif]

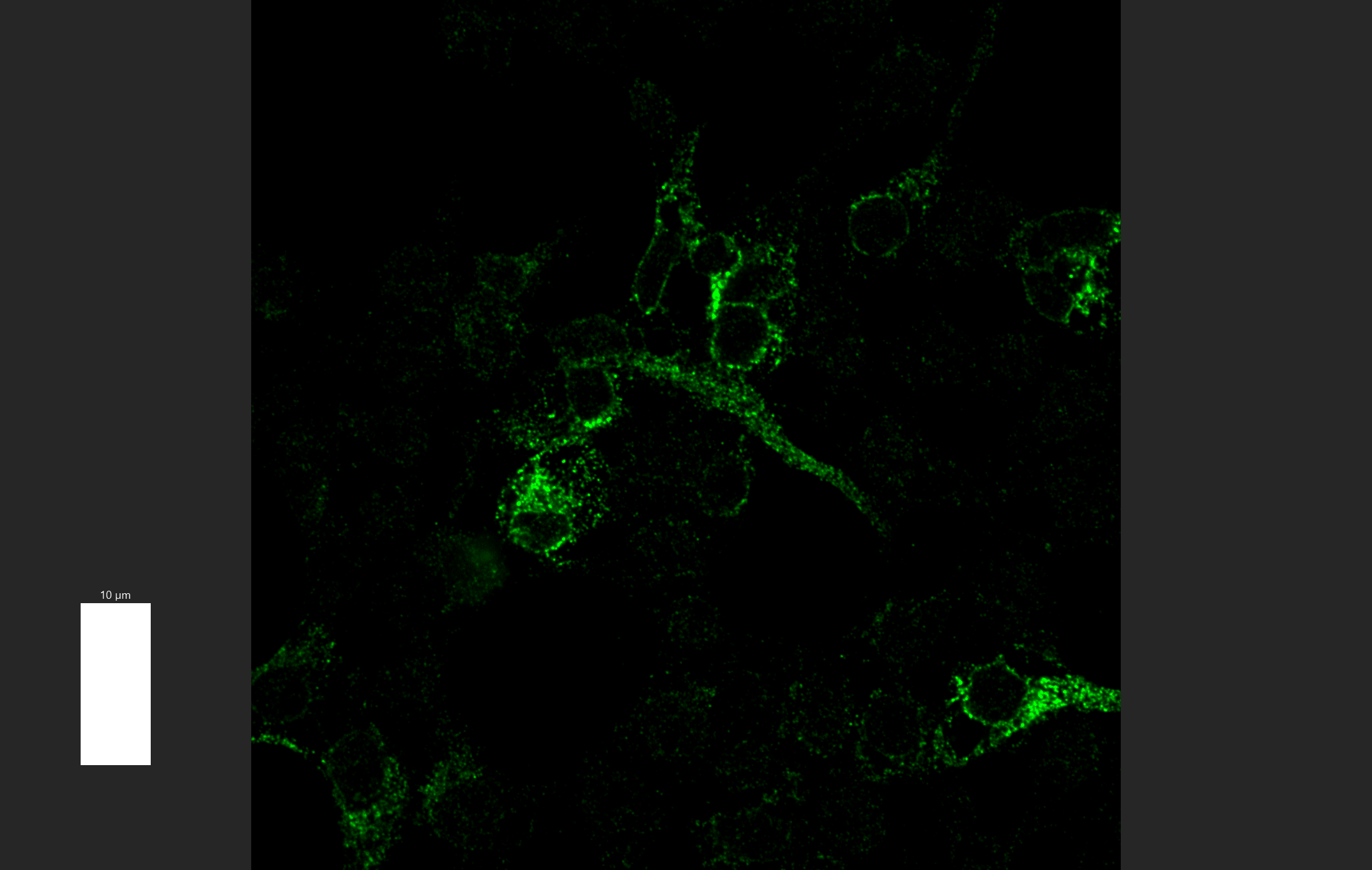

Supplement: Supplementary file 13 — Source data for Expanded View and Appendix [file 44319_2026_810_MOESM13_ESM.zip › Source Data for Expanded View and Appendix/Figure_EV3/EV3E/IF_Cav1_4.tif]

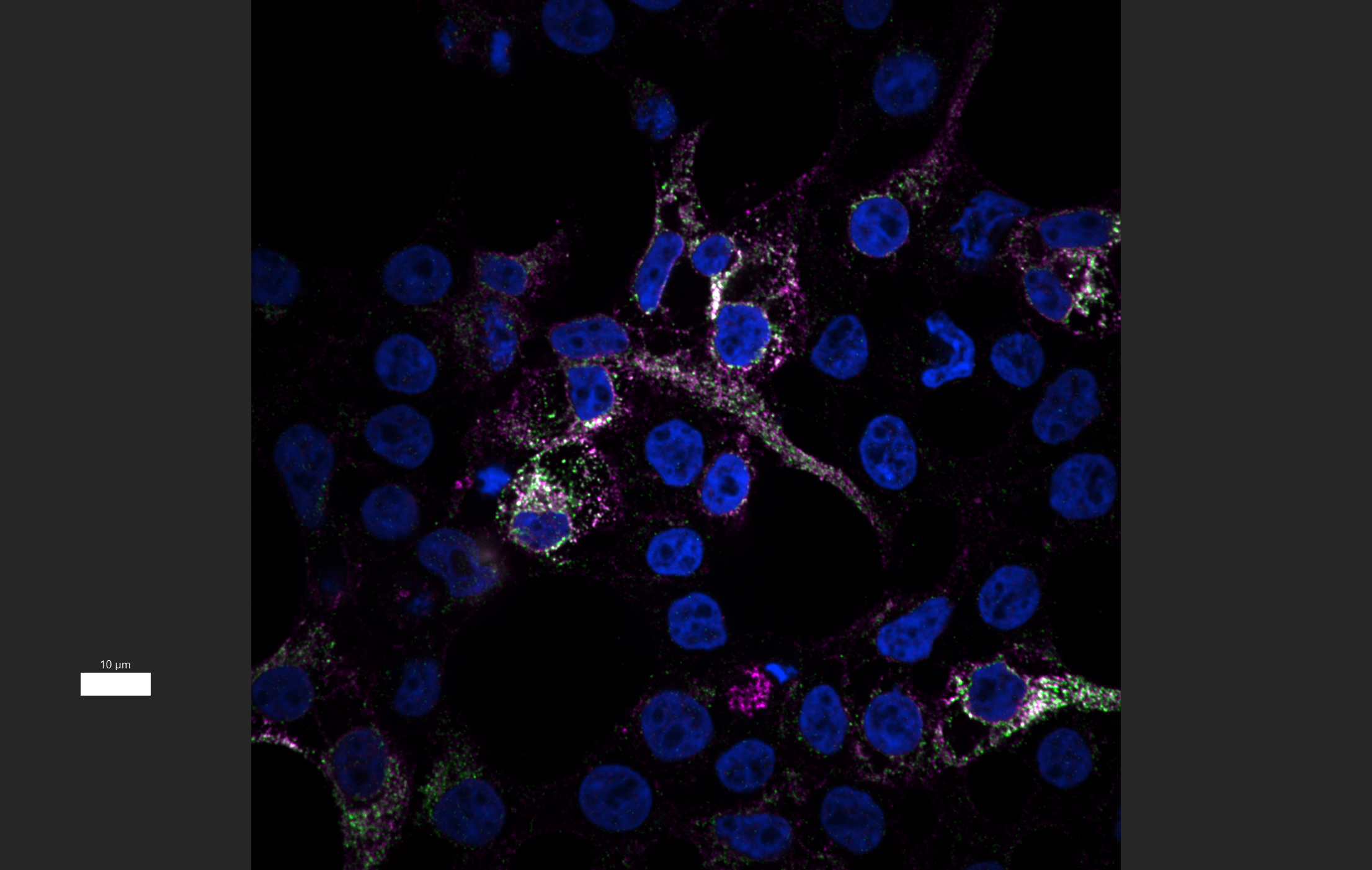

Supplement: Supplementary file 13 — Source data for Expanded View and Appendix [file 44319_2026_810_MOESM13_ESM.zip › Source Data for Expanded View and Appendix/Figure_EV3/EV3E/IF_DAPI+Cav1_4+HA.tif]

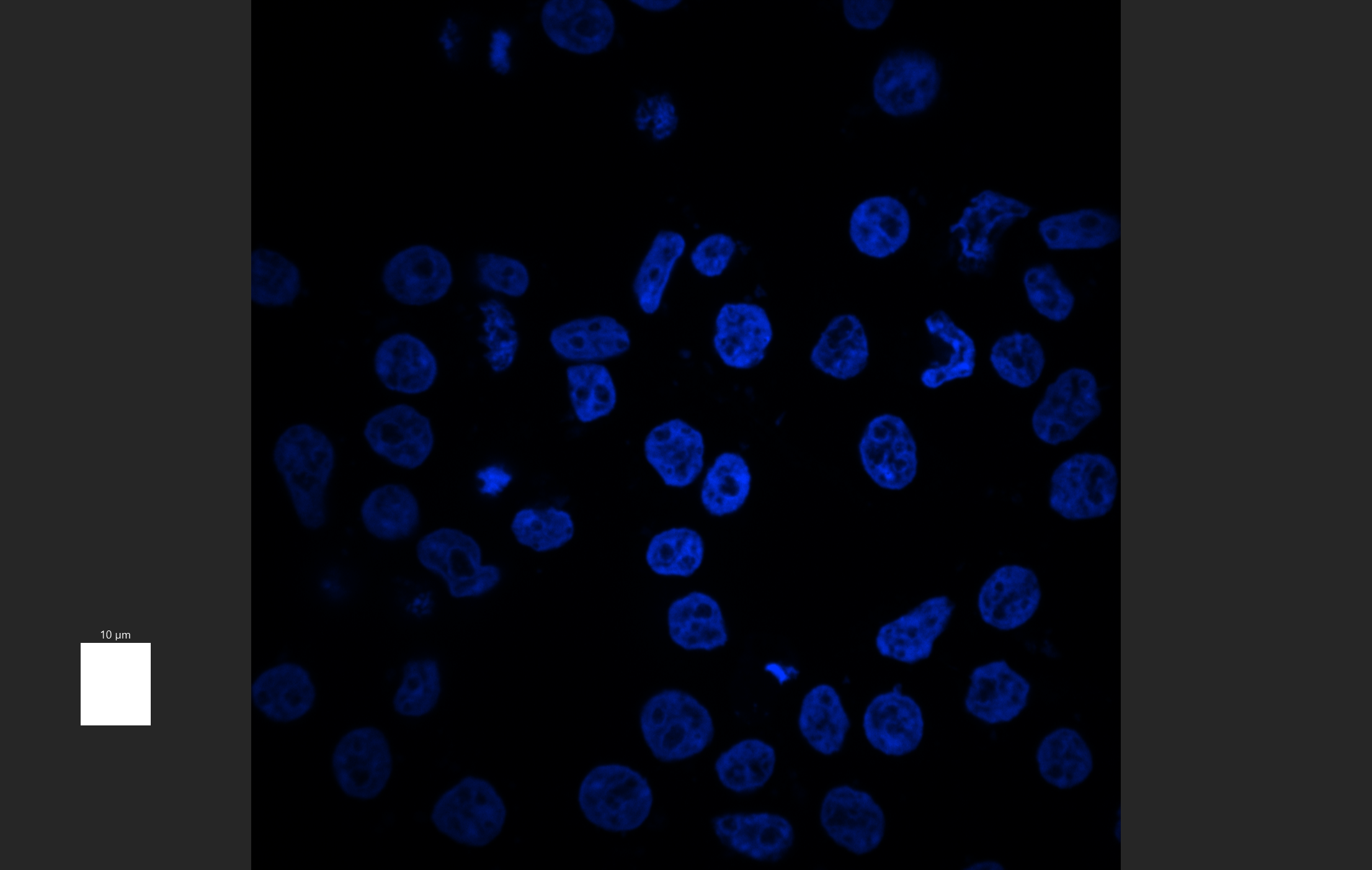

Supplement: Supplementary file 13 — Source data for Expanded View and Appendix [file 44319_2026_810_MOESM13_ESM.zip › Source Data for Expanded View and Appendix/Figure_EV3/EV3E/IF_DAPI.tif]

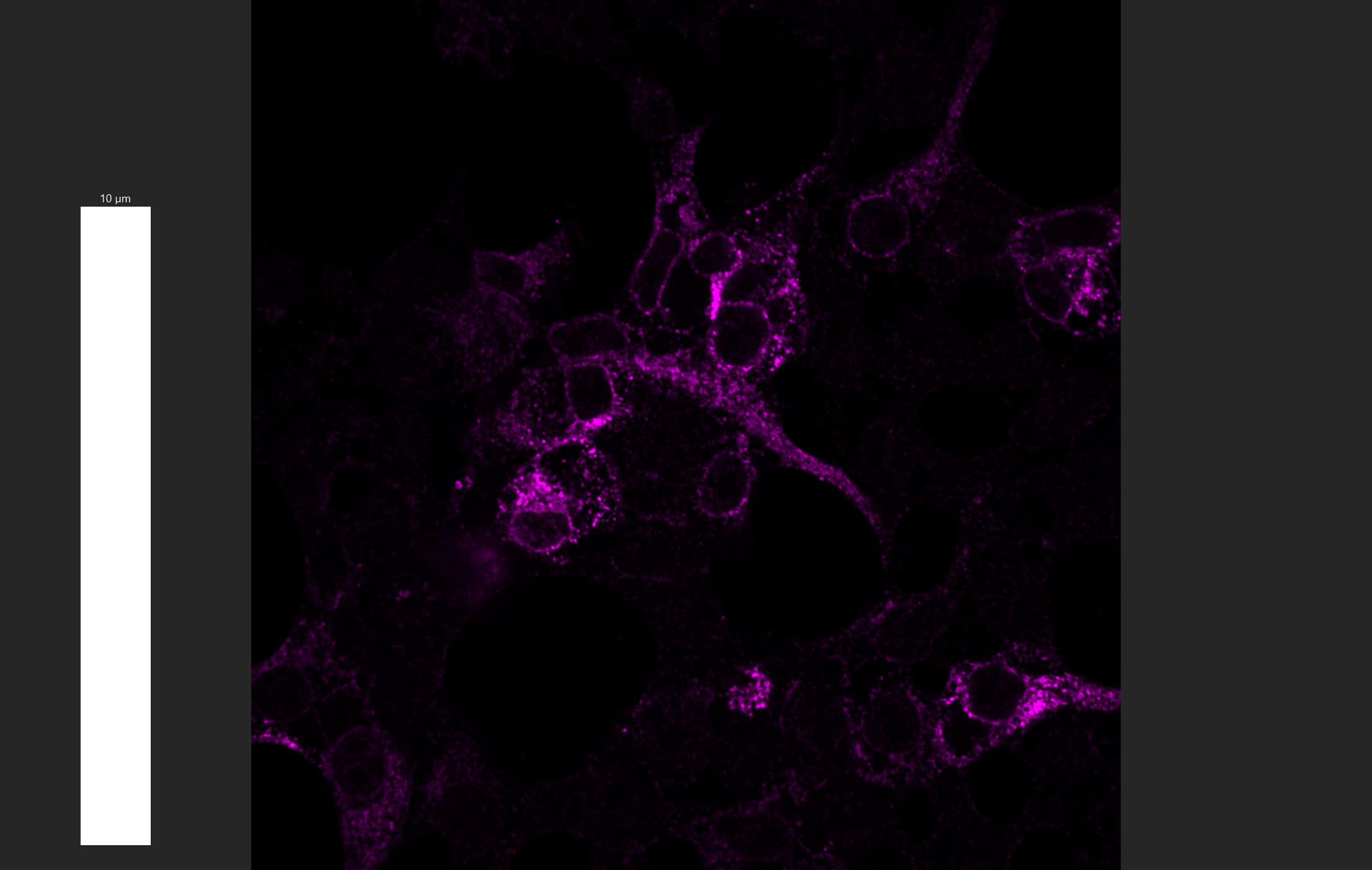

Supplement: Supplementary file 13 — Source data for Expanded View and Appendix [file 44319_2026_810_MOESM13_ESM.zip › Source Data for Expanded View and Appendix/Figure_EV3/EV3E/IF_HA.tif]

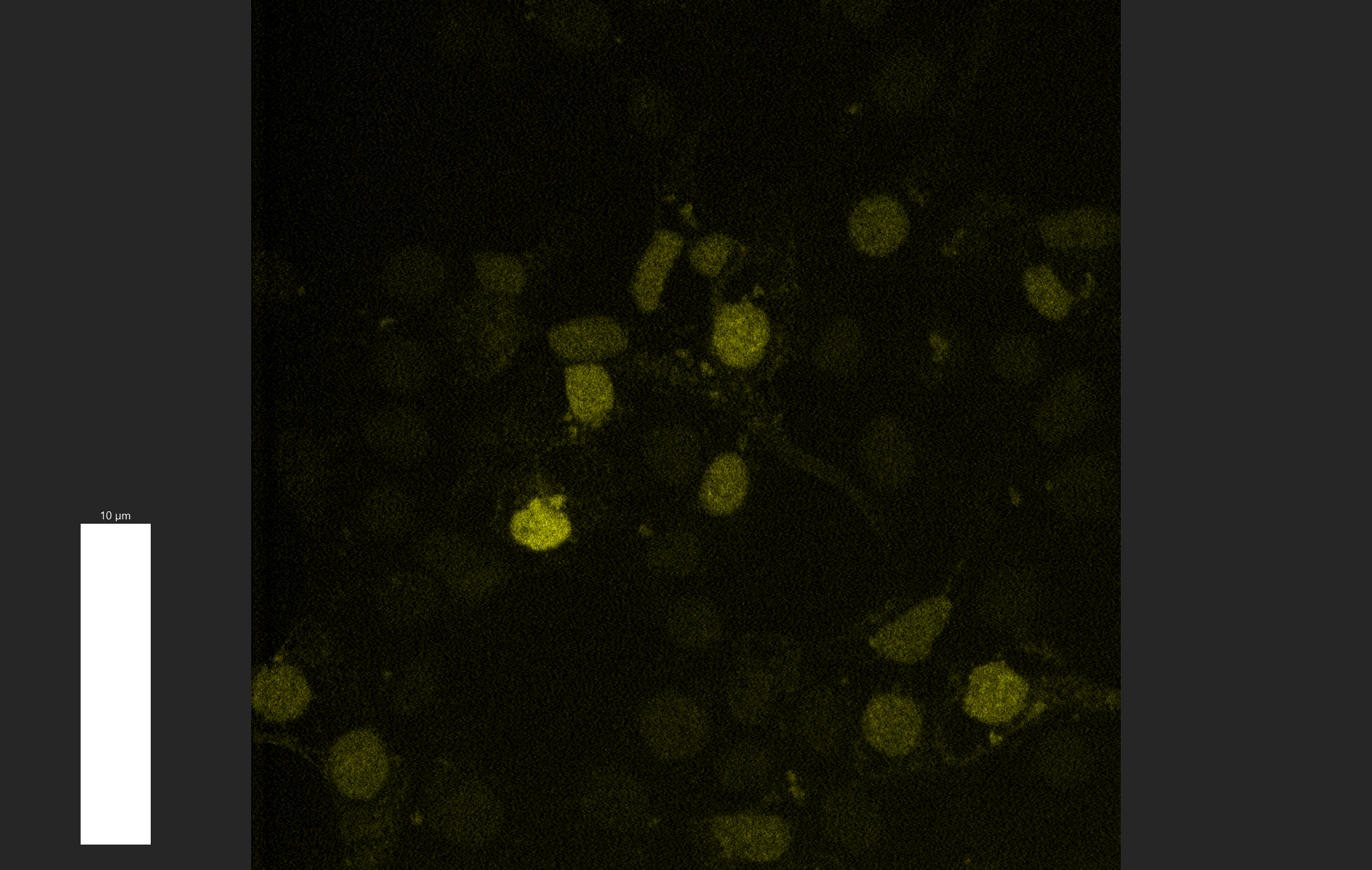

Supplement: Supplementary file 13 — Source data for Expanded View and Appendix [file 44319_2026_810_MOESM13_ESM.zip › Source Data for Expanded View and Appendix/Figure_EV3/EV3E/IF_mScarlet.tif]

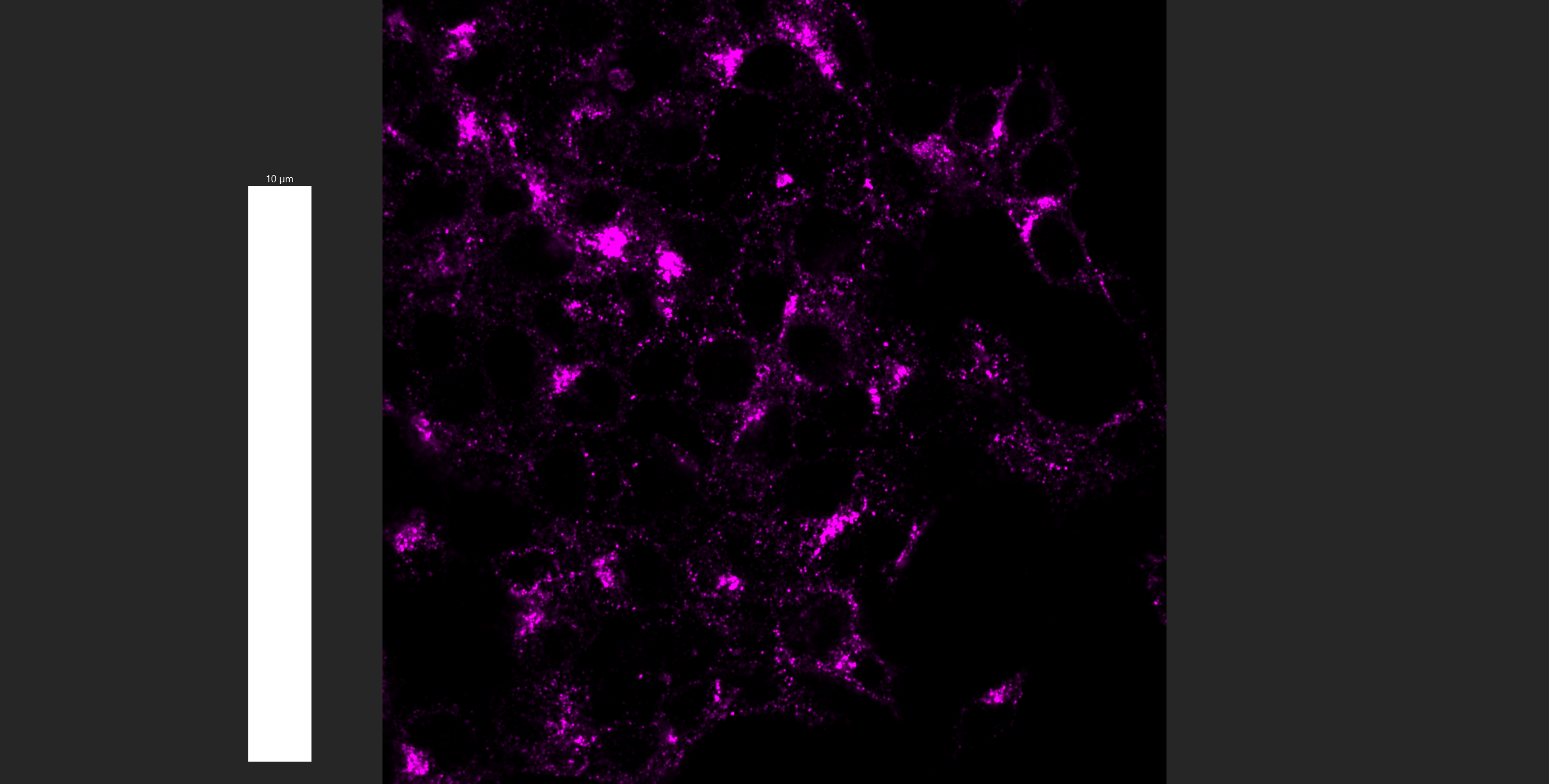

Supplement: Supplementary file 13 — Source data for Expanded View and Appendix [file 44319_2026_810_MOESM13_ESM.zip › Source Data for Expanded View and Appendix/Figure_EV3/EV3F/IF_AP1G1.tif]

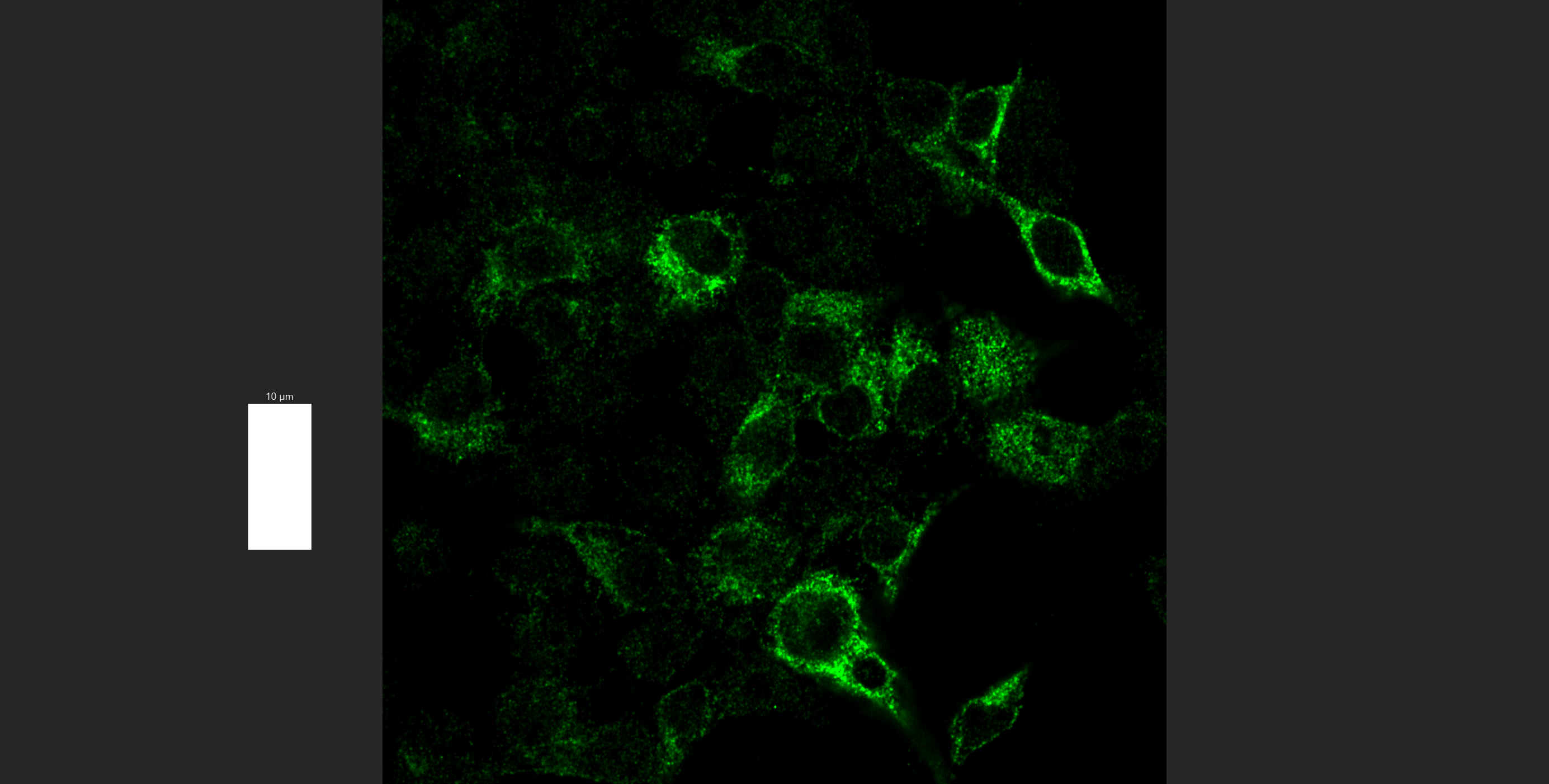

Supplement: Supplementary file 13 — Source data for Expanded View and Appendix [file 44319_2026_810_MOESM13_ESM.zip › Source Data for Expanded View and Appendix/Figure_EV3/EV3F/IF_Cav1_4.tif]

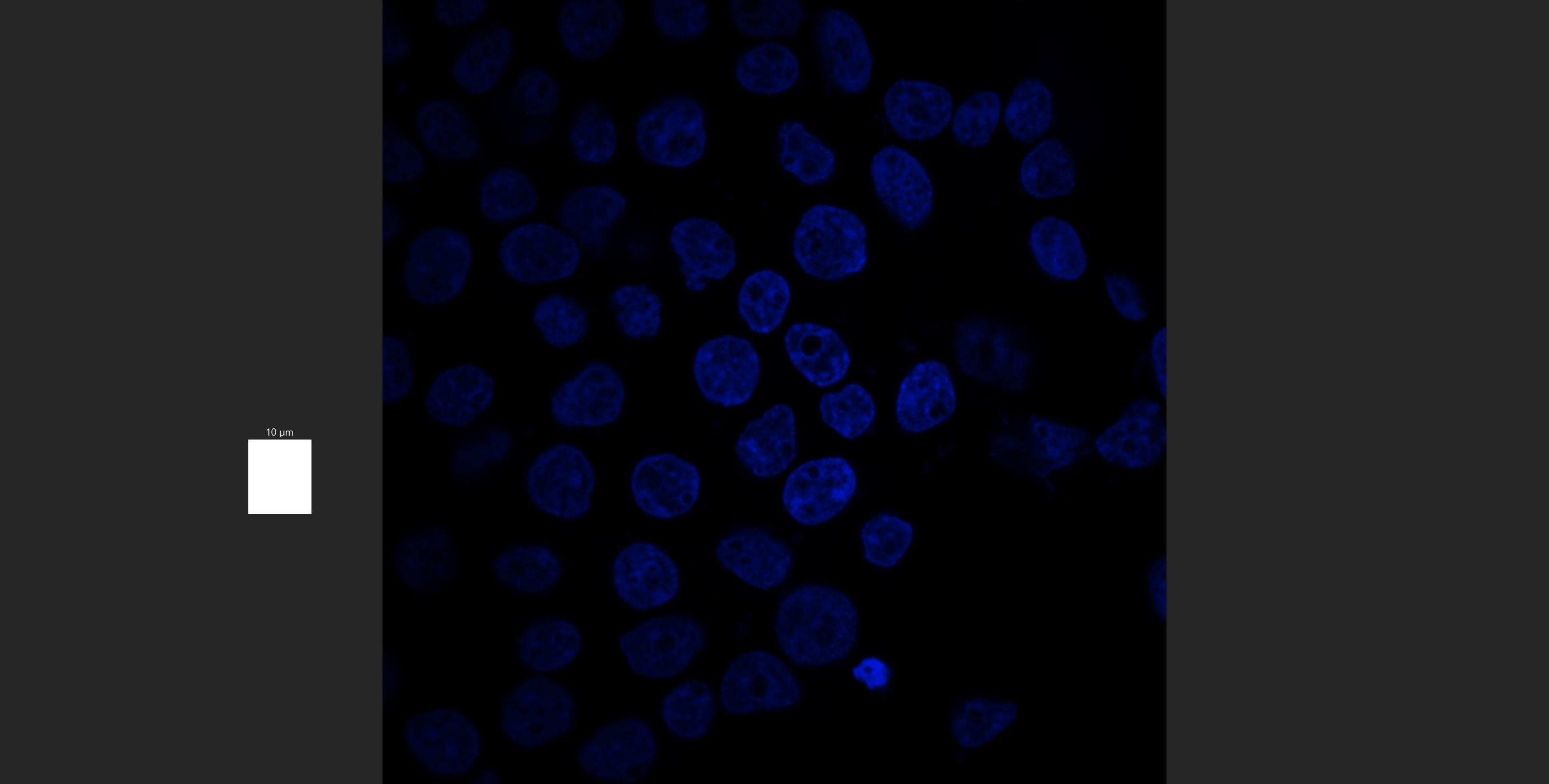

Supplement: Supplementary file 13 — Source data for Expanded View and Appendix [file 44319_2026_810_MOESM13_ESM.zip › Source Data for Expanded View and Appendix/Figure_EV3/EV3F/IF_DAPI.tif]

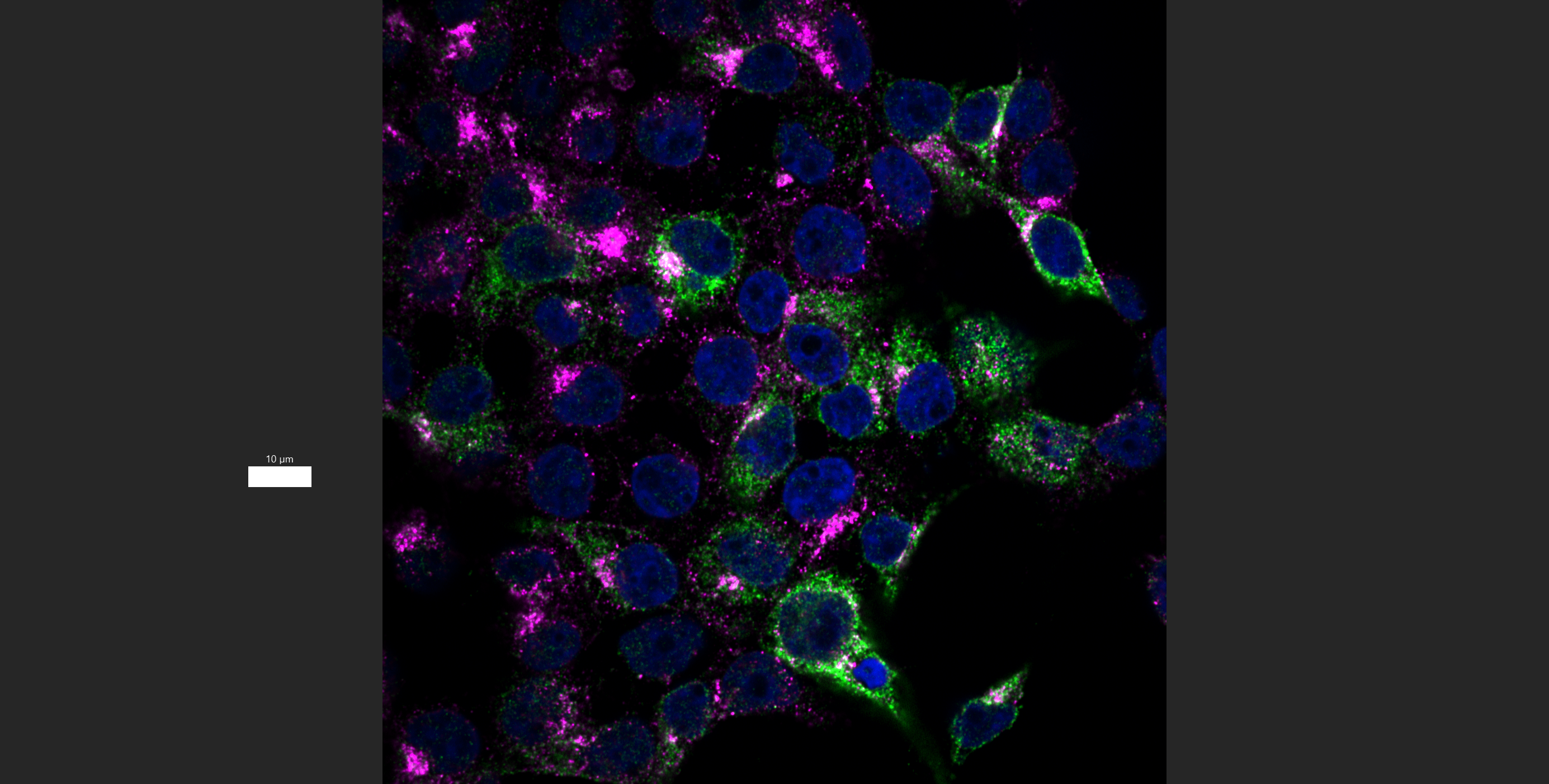

Supplement: Supplementary file 13 — Source data for Expanded View and Appendix [file 44319_2026_810_MOESM13_ESM.zip › Source Data for Expanded View and Appendix/Figure_EV3/EV3F/IF_DAPI_Cav1_4+AP1G1.tif]

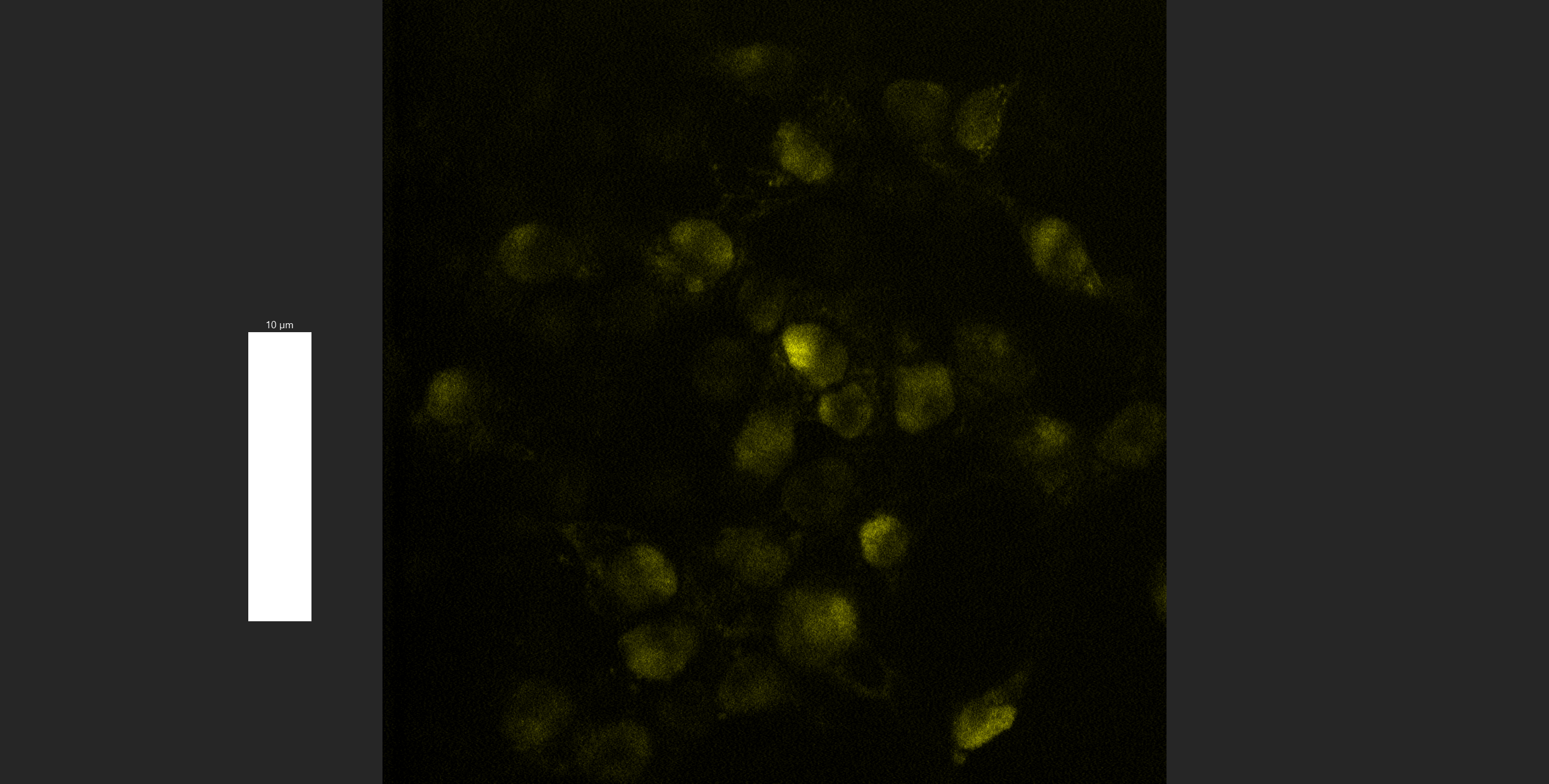

Supplement: Supplementary file 13 — Source data for Expanded View and Appendix [file 44319_2026_810_MOESM13_ESM.zip › Source Data for Expanded View and Appendix/Figure_EV3/EV3F/IF_mScarlet.tif]

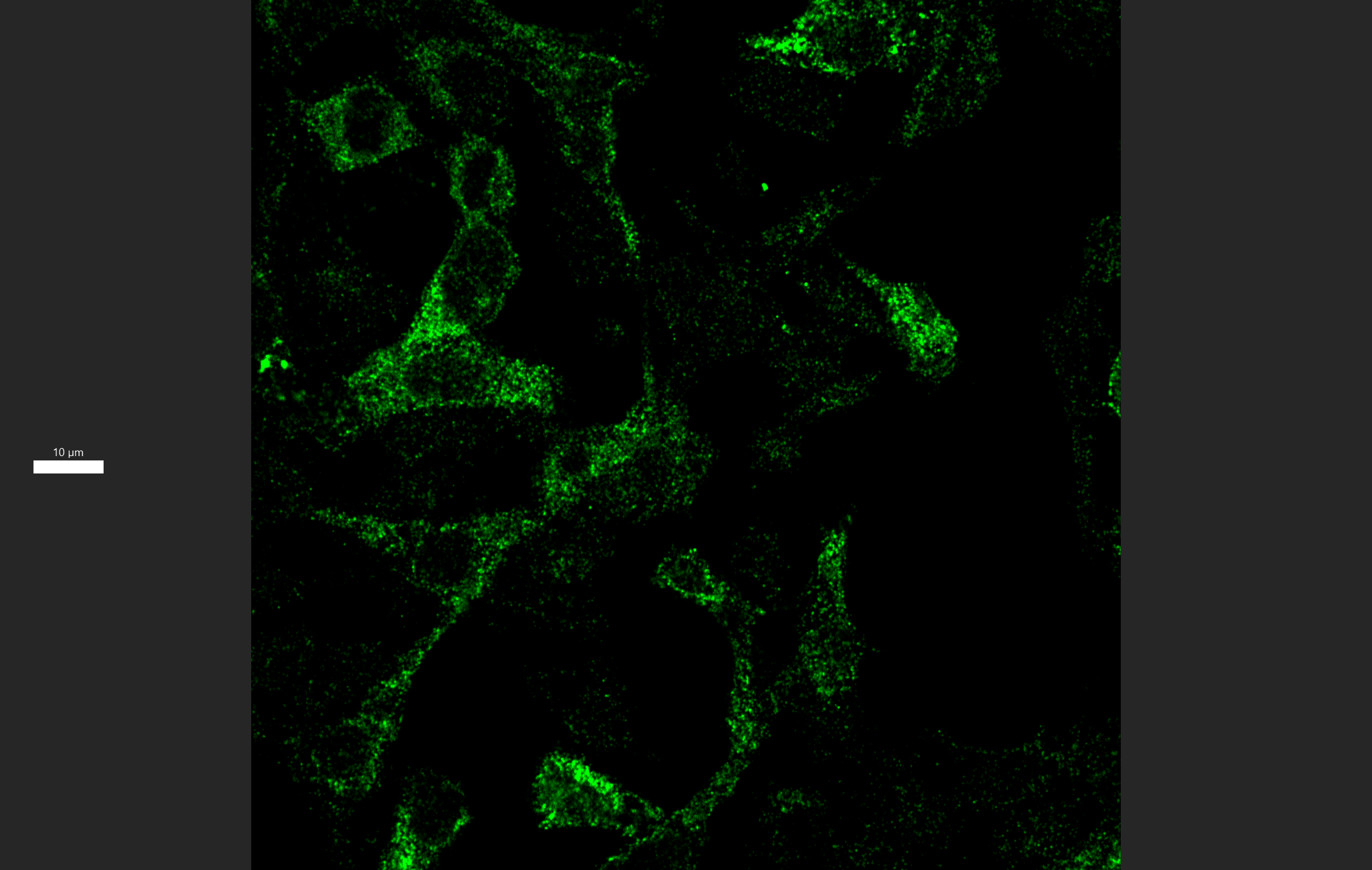

Supplement: Supplementary file 13 — Source data for Expanded View and Appendix [file 44319_2026_810_MOESM13_ESM.zip › Source Data for Expanded View and Appendix/Figure_EV3/EV3G/IF_Cav1_4.tif]

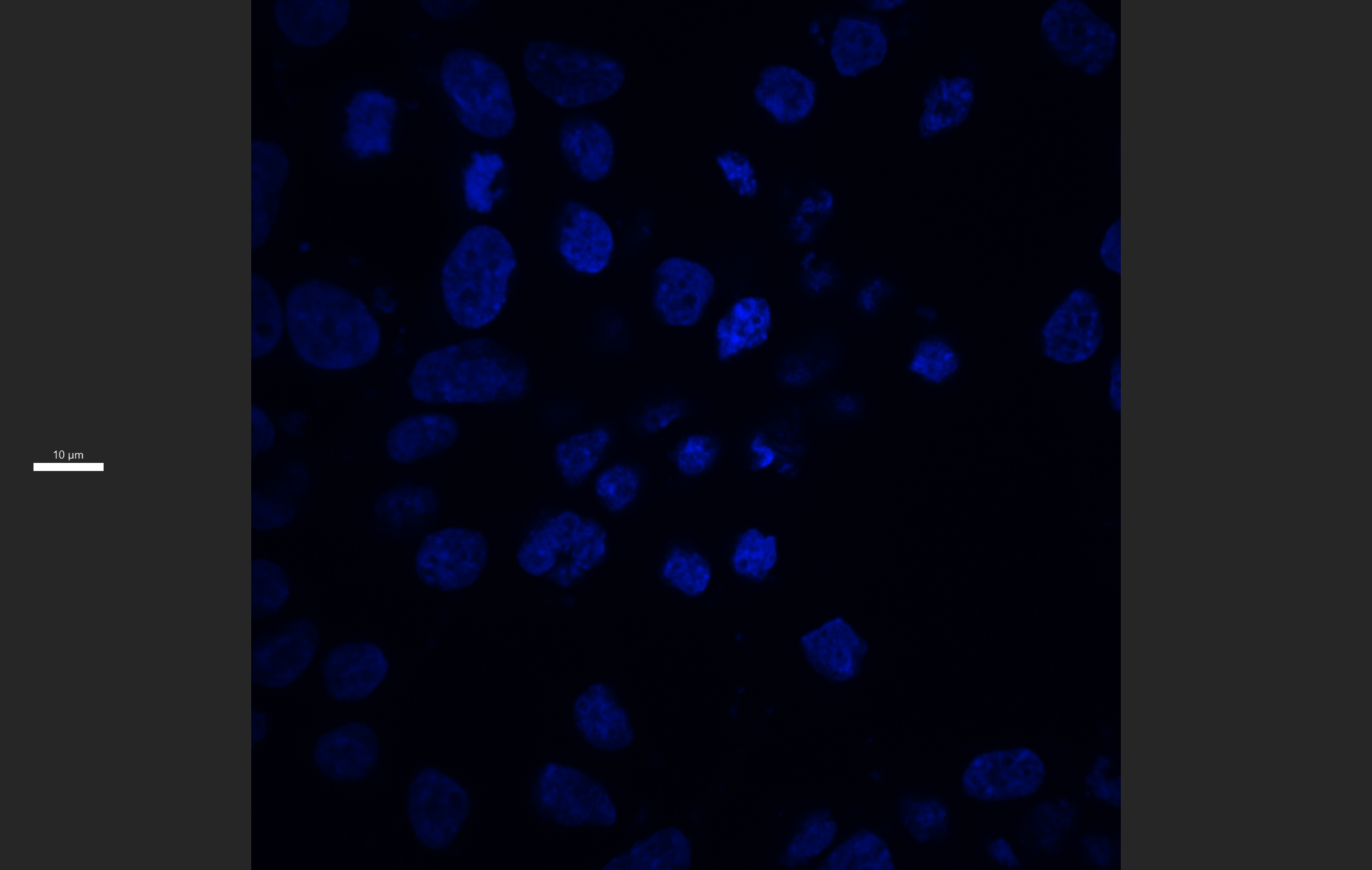

Supplement: Supplementary file 13 — Source data for Expanded View and Appendix [file 44319_2026_810_MOESM13_ESM.zip › Source Data for Expanded View and Appendix/Figure_EV3/EV3G/IF_DAPI.tif]

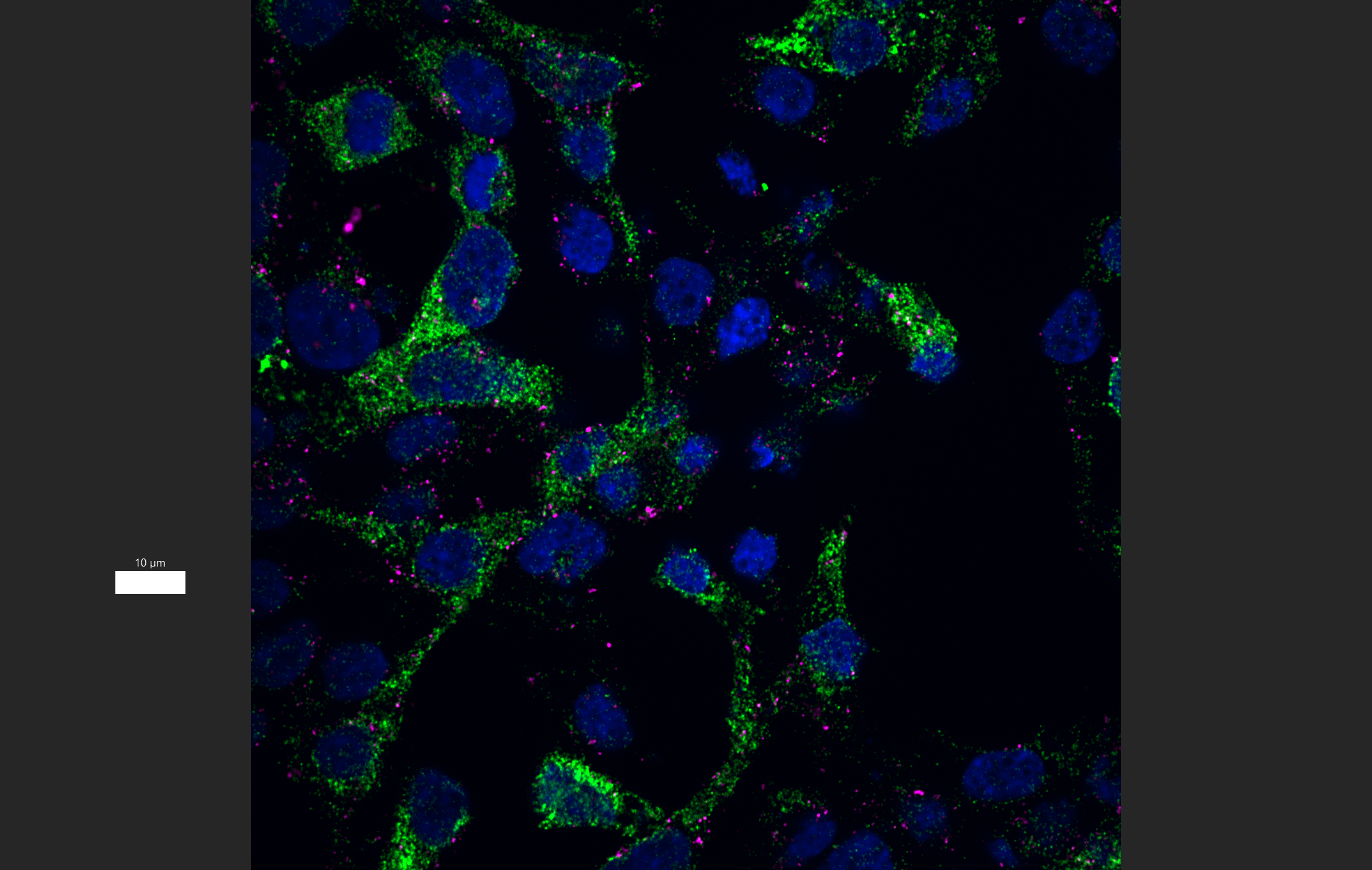

Supplement: Supplementary file 13 — Source data for Expanded View and Appendix [file 44319_2026_810_MOESM13_ESM.zip › Source Data for Expanded View and Appendix/Figure_EV3/EV3G/IF_DAPI_Cav1_4+VPS35.tif]

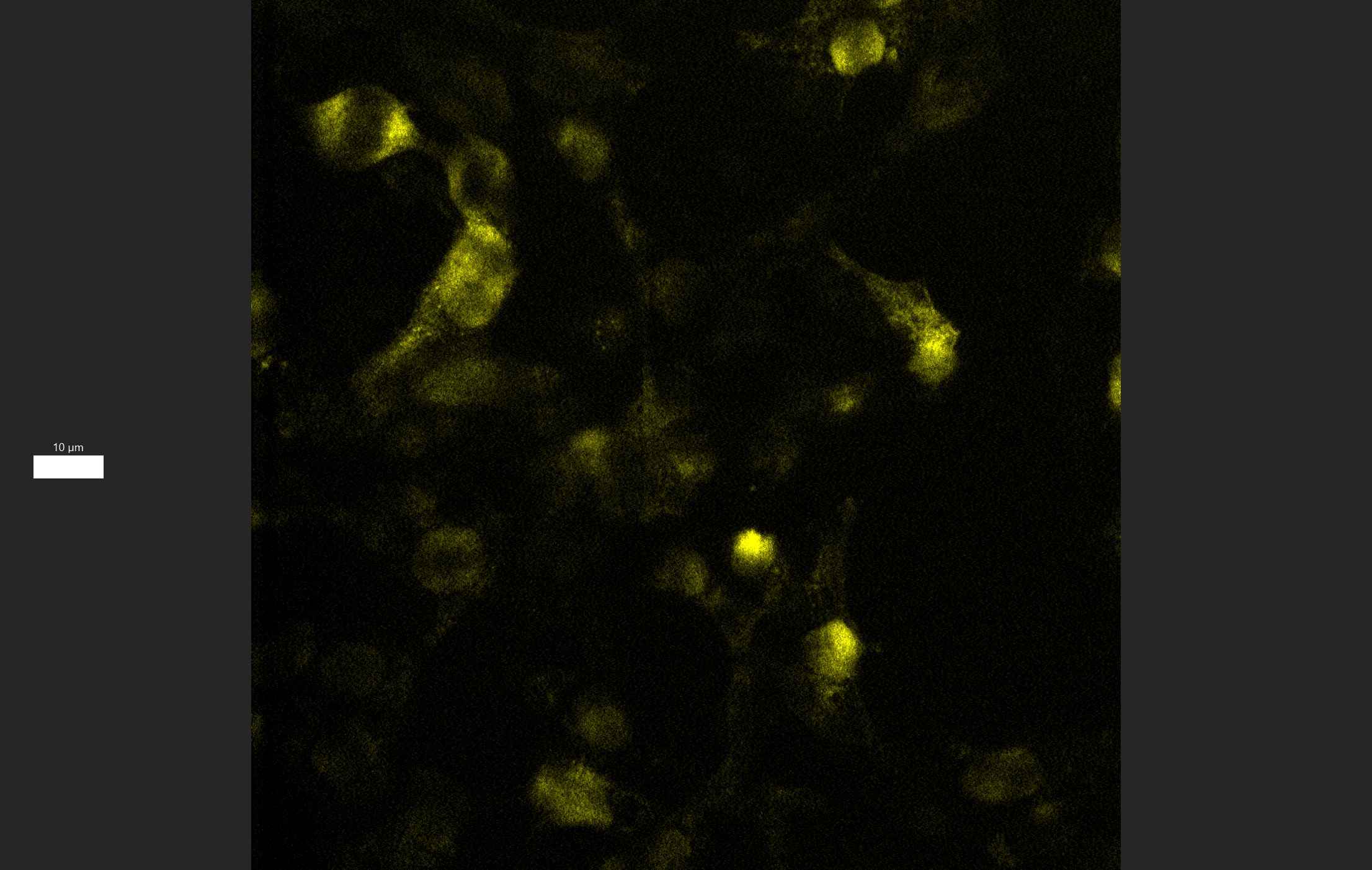

Supplement: Supplementary file 13 — Source data for Expanded View and Appendix [file 44319_2026_810_MOESM13_ESM.zip › Source Data for Expanded View and Appendix/Figure_EV3/EV3G/IF_mScarlet.tif]

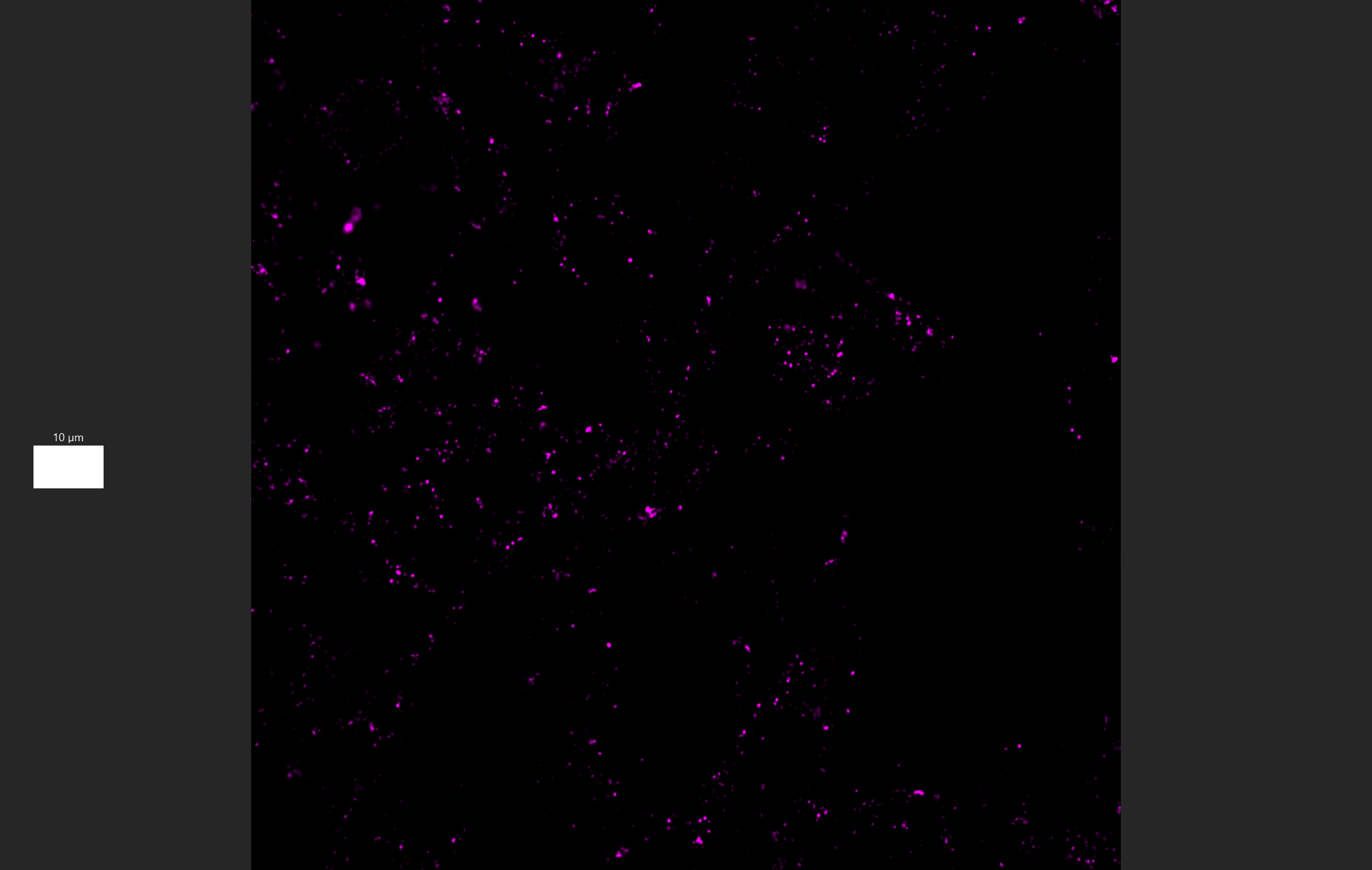

Supplement: Supplementary file 13 — Source data for Expanded View and Appendix [file 44319_2026_810_MOESM13_ESM.zip › Source Data for Expanded View and Appendix/Figure_EV3/EV3G/IF_VPS35.tif]
