# Supplementary material for: L-type voltage-gated Ca2+ channels control T cell killing via non-canonical Hedgehog signalling
Source: EMBO Rep. 2026 Jun 8;27(13):3689–730. doi: 10.1038/s44319-026-00810-8 (PMC13354577; doi:10.1038/s44319-026-00810-8)
Supplement: Supplementary file 14 — Expanded View Figures [file 44319_2026_810_MOESM14_ESM.pdf]

Expanded View Figures

Figure EV1. Phenotyping of *Gli1* KO mice, tumour-infiltrating *Gli1* KO T lymphocytes, and viability of GANT61-treated human CTLs.

(**A–C**) *Gli1* KO mice have phenotypically normal peripheral T cells. Splenocytes were isolated from *Gli1* WT, *Gli1* HET, and *Gli1* KO mice. (**A**) Splenocytes were stained for flow cytometry analysis of T cells ($CD3^{+}$, top panel), $CD4^{+}$ and $CD8^{+}$ T cells (middle panel), and naive ($CD62L^{+}$, $CD44^{-}$), central memory (CM, $CD62L^{+}$, $CD44^{+}$), and effector memory (EM, $CD62L^{-}$, $CD44^{-}$) subsets (bottom two panels). Representative flow cytometry plots for WT, HET, and KO mice are shown. (**B**, **C**) Quantitative analysis of percentages and cell numbers from stainings in (**A**). $n = 12$–19 biological replicates from 9 independent experiments. Every circle (WT), square (HET), and triangle (KO) represents a single mouse. Error bars indicate SD. Statistical significance was assessed using a one-way ANOVA with Dunnett's multiple comparison test. (**D–F**) Tumour-infiltrating *Gli1* WT and *Gli1* KO $CD8^{+}$ lymphocytes are phenotypically similar. Tumour-infiltrating $CD8^{+}$ lymphocytes ($CD8^{+}$ TILs) were isolated from tumours on day 27 (experimental setup shown in Fig. 1D). (**D**) Representative gating strategy. In brief, cells were gated on live, singlet $CD45^{+}$ $CD8^{+}$ cells for further analysis of CD44, CD62L, CD27, TIM3 and PD1 expression. Gating was performed based on FMO controls. (**E**) Absolute numbers of $CD45^{+}$ and $CD8^{+}$ cells within the tumour. (**F**) Mean fluorescence intensities (MFIs) of CD62L, CD44, TIM3, PD1 and CD27 on $CD8^{+}$ cells 27 (*Gli1* WT $n = 8$, *Gli1* KO $n = 6$ biological replicates pooled from 2 independent experiments). Bars represent the mean; error bars indicate SD. Statistical significance was assessed using an unpaired two-tailed Student's t test (**E**—left panel), a Mann-Whitney two-tailed test (**E**—right panel) or an unpaired two-tailed Student's t test (**F**). (**G**) Viability of human CTLs was determined by flow cytometry after treatment with 5 mM GANT61 for 8–18 h on day 12–14 post stimulation. $n = 6$ biological replicates from 2 independent experiments. Symbols indicate individual human donors. Bars represent the mean; error bars shown are SD. Statistical significance was assessed using an paired two-tailed Student's t test. ns = not significant.

 © The Author(s)

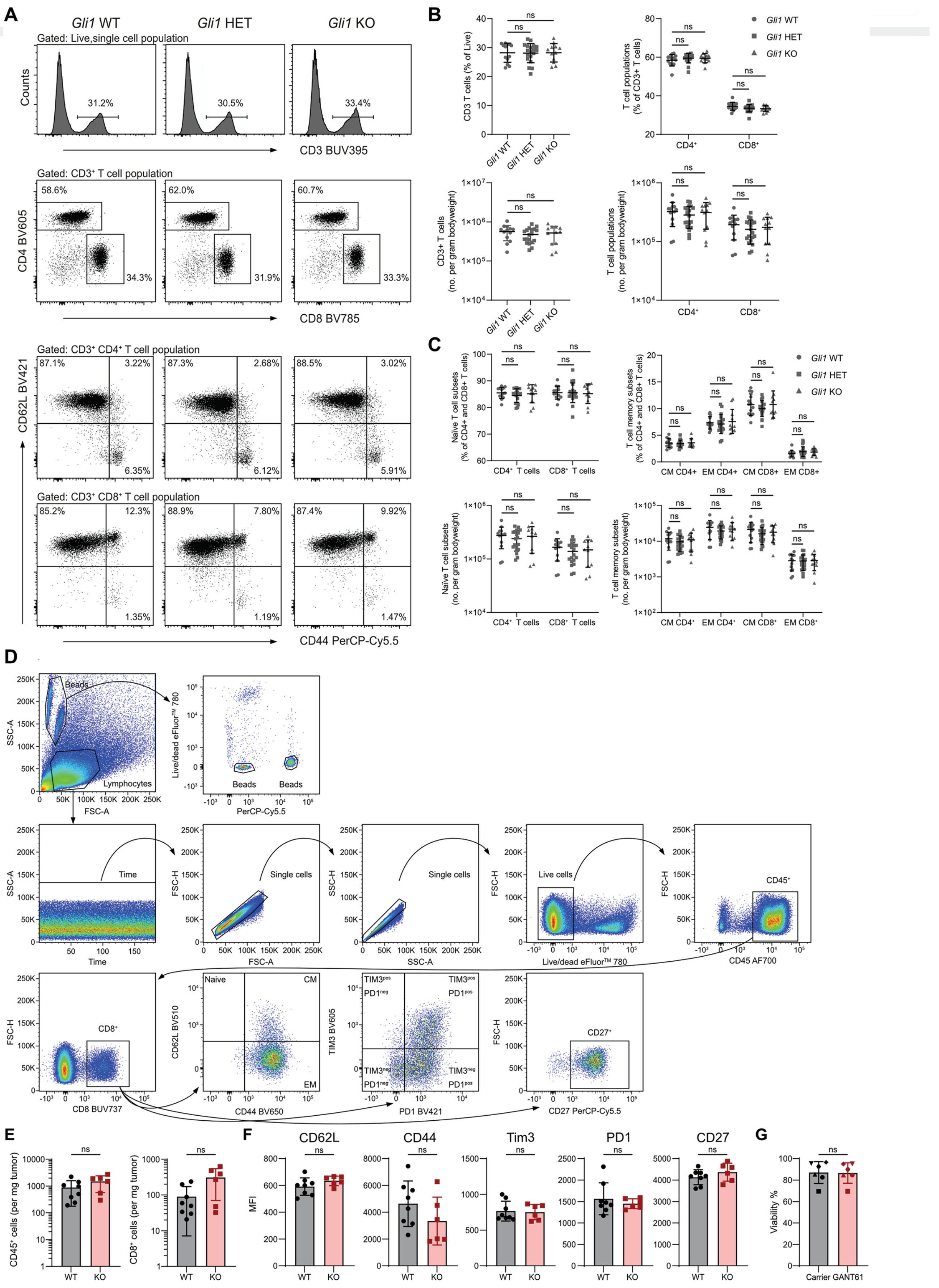
A
Gli1 WT
Gli1 HET
Gli1 KO
Gated: Live,single cell population
Counts
31.2%
30.5%
33.4%
CD3 BUV395
Gated: CD3+ T cell population
CD4 BV605
58.6%
34.3%
62.0%
31.9%
60.7%
33.3%
CD8 BV785
Gated: CD3+ CD4+ T cell population
CD62L BV421
87.1%
3.22%
6.35%
87.3%
2.68%
6.12%
88.5%
3.02%
5.91%
Gated: CD3+ CD8+ T cell population
85.2%
12.3%
1.35%
88.9%
7.80%
1.19%
87.4%
9.92%
1.47%
CD44 PerCP-Cy5.5
B
CD3 T cells (% of Live)
T cell populations (% of CD3+ T cells)
CD3+ T cells (no. per gram bodyweight)
T cell populations (no. per gram bodyweight)
CD4+
CD8+
ns
C
Naive T cell subsets (% of CD4+ and CD8+ T cells)
T cell memory subsets (% of CD4+ and CD8+ T cells)
Naive T cell subsets (no. per gram bodyweight)
T cell memory subsets (no. per gram bodyweight)
CD4+ T cells
CD8+ T cells
CM CD4+
EM CD4+
CM CD8+
EM CD8+
D
Beads
Lymphocytes
SSC-A
FSC-A
Live/dead eFluor™ 780
PerCP-Cy5.5
Time
Single cells
FSC-H
SSC-H
Live cells
CD45+
CD45 AF700
CD8+
CD8 BUV737
CD62L BV510
Naive
CM
EM
CD44 BV650
TIM3 BV605
TIM3pos PD1neg
TIM3pos PD1pos
TIM3neg PD1neg
TIM3neg PD1pos
PD1 BV421
CD27+
CD27 PerCP-Cy5.5
E
CD45+ cells (per mg tumor)
CD8+ cells (per mg tumor)
WT
KO
F
CD62L
CD44
Tim3
PD1
CD27
MFI
G
Viability %
Carrier
GANT61

Figure EV2. Validation of TCR signalling inhibitors, cell viability of mouse CD8 + T cells upon Ca^{2+} blockade and characterisation of *dLckCre*⁺ *Ihh* KO, *GzmBER*^{T2}*Cre*⁺ *Smo* KO mice and retroviral overexpression.

(**A–C**) Validation of TCR signalling inhibitors. (**A**) CTLs were nucleofected with GFP or GFP-VIVIT on day 6. Cells were restimulated with PMA/Ionomycin on day 7 prior to intracellular flow cytometric staining for IL-2 and IFN-γ. FACS plots are gated on GFP^+ cells. (**B**) CTLs were nucleofected with GFP or Dominant Negative IκBα (DN IκBα) on day 6. Cells were restimulated with anti-CD3 on day 7 for 1 h prior to ImageStream analysis. (**C**) Naive $CD8^+$ T cells were isolated from spleens and peripheral lymph nodes of *Rag2* KO *OT-I* mice and stimulated with cross-linked soluble anti-CD3/CD28 in the presence of 10 μM U0126 or carrier control prior to analysis by flow cytometry of phosphorylated Erk1/2 (pErk1/2). (**A–C**) One representative experiment of $n = 2$–3 biological replicates from 2–3 independent experiments is shown. (**D**, **E**) Cell viability of mouse $CD8^+$ T cells upon Ca^{2+} blockade. (**D**) Murine CTLs were restimulated with plate-bound anti-CD3ε antibodies for 3 h in the presence of indicated doses of the cell permeable Ca^{2+} chelator BAPTA-AM or carrier control before viability was assessed via the Beckman Coulter Vi-Cell XR. $n = 4$–5 biological replicates from 4–5 independent experiments. Symbols indicate biological replicates. Bars represent the mean; error bars shown are SD. Statistical significance was assessed using a one-way ANOVA with Dunnett's multiple comparison test comparing each condition to DMSO (0.025%). (**E**) Murine CTLs were restimulated with plate-bound anti-CD3e in the presence of the indicated Ca^{2+} channel inhibitors or carrier control for 15 h before flow cytometric analysis for cell viability. $n = 3$ biological replicates from 3 independent experiments. Symbols indicate biological replicates. Bars represent the mean; error bars indicate SD. Statistical significance was assessed using a one-way ANOVA with Dunnett's multiple comparison test comparing each condition to carrier. (**F–I**) Characterization of *dLckCre*⁺ *Ihh* KO, *GzmBER*^{T2}*Cre*⁺ *Smo* KO mice and retroviral transduction constructs. (**F**) Splenocytes were isolated from *dLckCre*⁺*Ihh*$^{+/fl}$ (*Ihh* HET) and *dLckCre*⁺*Ihh*$^{fl/fl}$ (*Ihh* KO) mice and subjected to flow cytometric phenotypic analysis. Representative FACS plots are shown (left 2 panels). Quantification of relative percentages, cell numbers and steady-state phenotype: naive ($CD62L^+$, $CD44^-$), central memory (CM, $CD62L^+$, $CD44^+$), and effector memory (EM, $CD62L^-$, $CD44^-$) shown on the right two panels. Every square represents one HET, and every triangle one individual KO mouse. $n = 5$–6 biological replicates from 5–6 independent experiments. Error bars indicate SD. Statistical significance was assessed using multiple unpaired Welch's tests with Holm-Sidak multiple comparisons test. (**G**) Splenocytes were isolated from *GzmBER*^{T2}*Cre*⁺*Smo*$^{+/+}$ (*Smo* WT) and *GzmBER*^{T2}*Cre*⁺ *Smo*$^{fl/fl}$ (*Smo* KO) mice and subjected to flow cytometric phenotypic analysis. Representative FACS plots are shown (left). Quantification of relative percentages and steady-state memory phenotype shown (middle). Every square represents one WT, and every triangle one individual KO mouse. CTLs were generated from these mice and restimulated at day 8/9 for qRT-PCR analysis of Smo (right). $n = 4$–7 biological replicates from 4 independent experiments. Error bars indicate SD. p values were calculated using multiple unpaired Welch's tests with Holm-Sidak multiple comparisons (0 h $p = 0.0296$, 3 h $p = 0.0222$). (H) $CD8^+$ T cells were retrovirally transduced with constructs encoding empty vector (EV), SmoM2 or Ihh, respectively. Representative flow cytometry plots of sorted, transduced ($Thy1.1^+$) cell populations shown (left panel). Sorted, transduced CTLs were restimulated at day 8/9 for qRT-PCR analysis of Smo (middle panel) or Ihh (right panel), respectively. $n = 3$ biological replicates from 3 independent experiments. p values were calculated using a two-way ANOVA with Sidak's multiple comparison test (Smo 0 h $p = 0.0073$, Smo 4 h $p < 0.0001$, Smo 15 h $p < 0.0001$, Ihh 0 h $p = 0.084$, Ihh 4 h $p = 0.0098$, Ihh 15 h $p = 0.0007$). (**I**) $CD8^+$ T cells were retrovirally transduced with pMig constructs encoding empty vector (EV), SmoM2 or Ihh, respectively. Sorted, transduced CTLs were restimulated on day 8/9 for qRT-PCR analysis. $n = 3$ biological replicates from 3 independent experiments. Statistical significance was assessed using a two-way ANOVA with Tukey's multiple comparison test. (**H**, **I**) Data is normalised to *CD3ε* as a reference gene. Similar results were obtained when *Tbp* was used as a reference gene. Symbols indicate biological replicates. Bars represent the mean; error bars indicate SD. *$p < 0.05$, **$p < 0.01$, ****$p < 0.0001$. ns = not significant.

 © The Author(s)

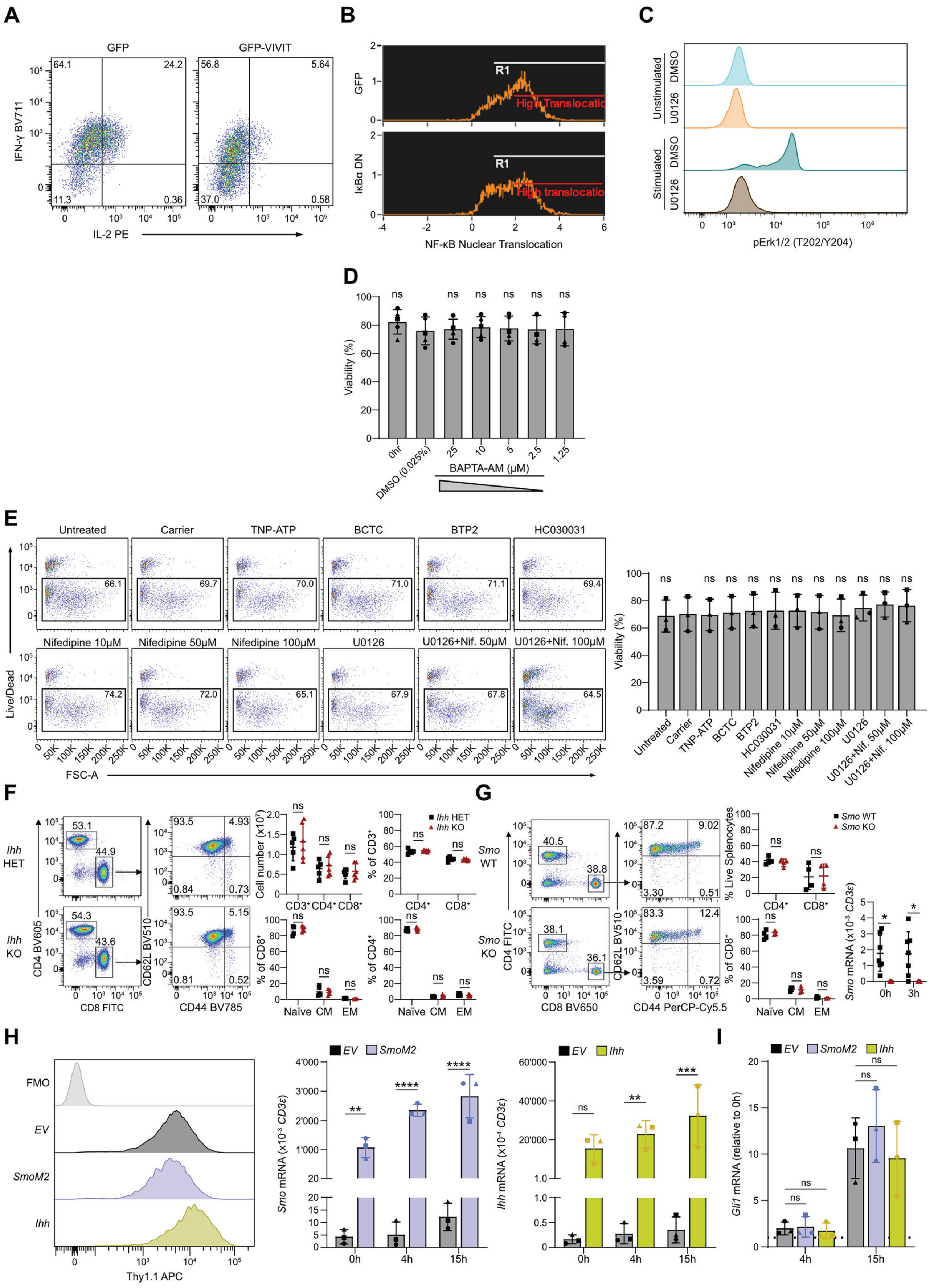

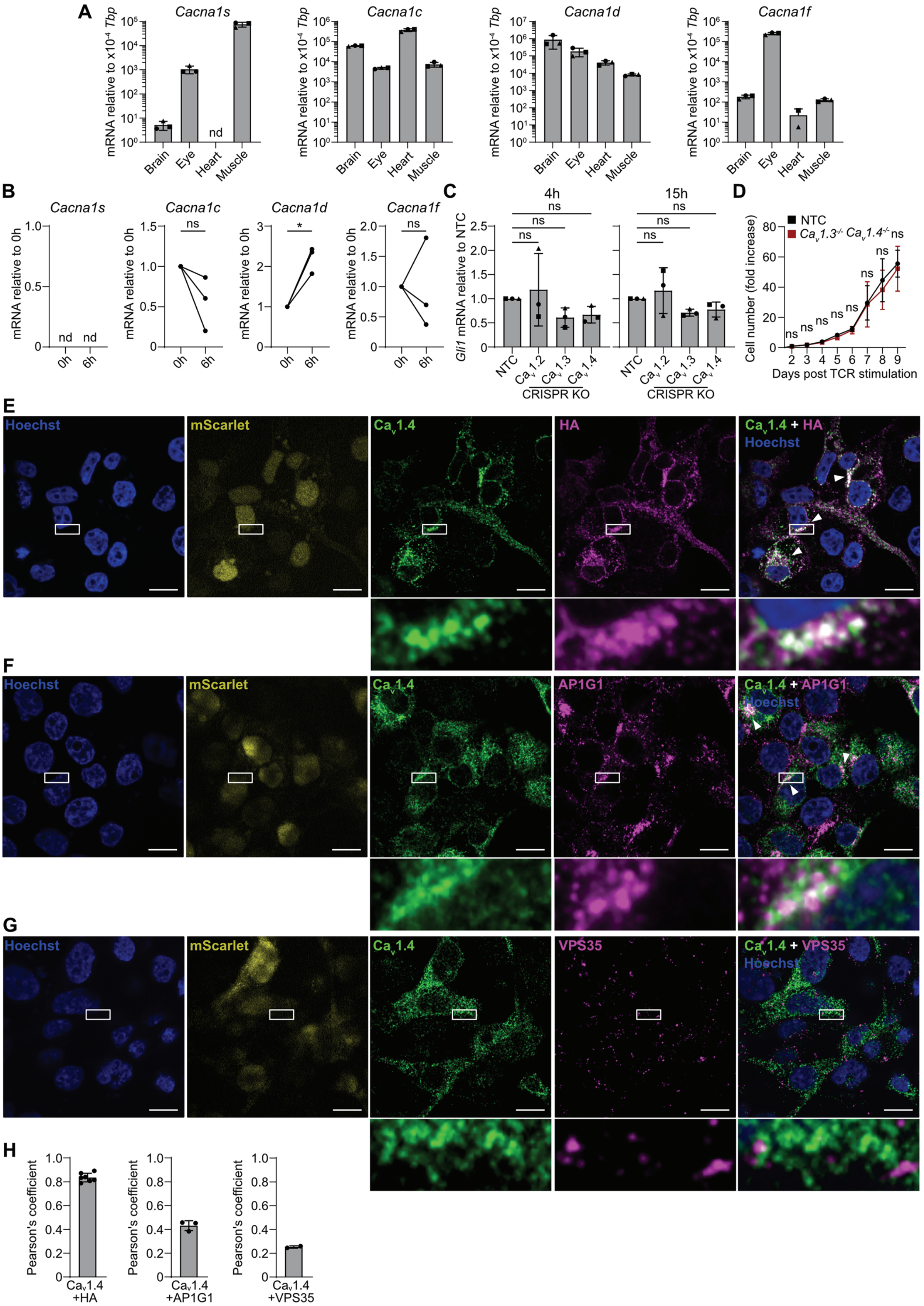
A
Cacna1s
Cacna1c
Cacna1d
Cacna1f
mRNA relative to x10⁻⁴ Tbp
Brain
Eye
Heart
Muscle
nd
B
Cacna1s
Cacna1c
Cacna1d
Cacna1f
mRNA relative to 0h
nd nd
ns
*
ns
0h
6h
C
4h
15h
ns
Gli1 mRNA relative to NTC
NTC
Cav1.2
Cav1.3
Cav1.4
CRISPR KO
D
NTC
Cav1.3-/- Cav1.4-/-
ns
Cell number (fold increase)
Days post TCR stimulation
E
Hoechst
mScarlet
Cav1.4
HA
Cav1.4 + HA Hoechst
F
AP1G1
Cav1.4 + AP1G1 Hoechst
G
VPS35
Cav1.4 + VPS35 Hoechst
H
Pearson's coefficient
Cav1.4 +HA
Cav1.4 +AP1G1
Cav1.4 +VPS35

 © The Author(s)

Figure EV3. Expression of Ca_v1 channels in murine CTLs, characterisation of CRISPR knockouts and validation of $Ca_v1.4$ antibody specificity and intracellular colocalisation in HEK293T cells.

(**A–D**) Expression of Ca_v1 channels and characterization of CRISPR knockouts. (**A**) Expression analysis of *Cacna1s,c,d,f* in murine tissues relative to *Tbp* as a housekeeping gene. $n = 3$ biological replicates. Similar results were obtained when *b2m* was used as a reference gene. Bars represent the mean; error bars indicate SD. (**B**) mRNA expression of *Cacna1s,c,d,f* in murine day 8 CTLs at steady state and after 6 h of TCR stimulation. Data is normalised to *Tbp* as a reference gene. Similar results were obtained when *b2m* was used as a reference gene. $n = 3$ biological replicates. *p* values were calculated using a two-tailed one sample t test ($p = 0.0245$). (**C**) CTLs were electroporated with RNP complexes at day 2 post stimulation to generate $Ca_v1.1^{-/-}$, $Ca_v1.2^{-/-}$, $Ca_v1.3^{-/-}$, $Ca_v1.4^{-/-}$ (KO) or non-targeting control (NTC) CTLs. CTLs were restimulated on day 8/9 with plate-bound anti-CD3ε for qRT-PCR analysis. $n = 3$ biological replicates from 3 independent experiments. Data is normalised to *CD3ε* as a reference gene. Similar results were obtained when *Tbp* was used as a reference gene. Bars represent the mean; error bars indicate SD. Statistical significance was assessed using a one-way ANOVA with Dunnett's multiple comparison test. (**D**) $Ca_v1.3^{-/-}$ and $Ca_v1.4^{-/-}$ CRISPR double KO CTLs and NTC CTLs were generated as described previously. Fold expansion in cell numbers after CRISPR performed at day 2 post-stimulation is shown. $n = 3$ biological replicates from 3 independent experiments. Symbols indicate biological replicates. Error bars indicate SD. Statistical significance was assessed using a two-way ANOVA with Sidak's multiple comparison test. (**E–H**) Validation of $Ca_v1.4$ antibody specificity and intracellular colocalisation with vesicular markers in HEK293T cells. HEK 293T cells were plated on coverslips prior to lipofection with a construct expressing an HA-tagged human $Ca_v1.4$ and mScarlet reporter. 18 h post lipofection, cells were fixed and stained with antibodies against (**E**) $Ca_v1.4$ and HA, (**F**) $Ca_v1.4$ and AP1G1, and (**G**) $Ca_v1.4$, VPS35. Nuclei were stained with Hoechst 33342. Representative images are shown. (**E**) $n = 3$ independent experiments. (**F**, **G**) $n = 1$ biological replicate. Single Z-stack is shown. Scale bar = 10 µm. White arrowheads indicate areas of colocalisation between $Ca_v1.4$ and HA (**E**) as well as $Ca_v1.4$ and AP1G1 (**F**). White box indicates the region shown at higher magnification in (**E–G**). Bar graphs in (**H**) show the Pearson's correlation coefficient between $Ca_v1.4$ and the indicated markers. Bars represent the mean; error bars indicate SD. ($Ca_v1.4$ + HA) $n = 198$ cells, from 7 images. ($Ca_v1.4$ + AP1G1) $n = 192$ cells, from 3 images. ($Ca_v1.4$ + VPS35) $n = 138$ cells, from 2 images. *$p < 0.05$. ns = not significant.

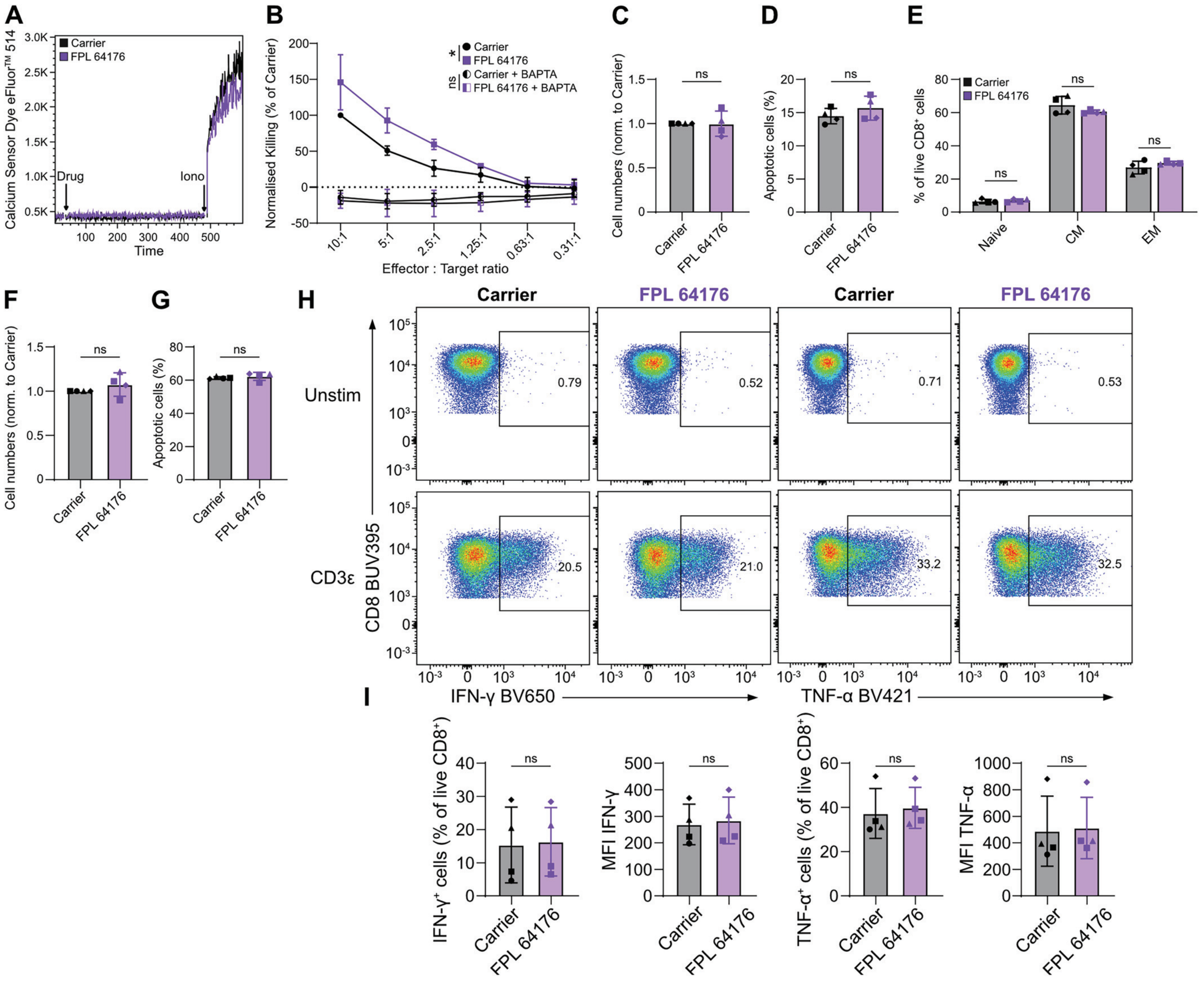

Figure EV4. Phenotypic characterisation of FPL 64176-treated murine and human CTLs.

(A–I) FPL 64176 does not lead to a Ca^{2+} response in the absence of TCR stimulation, enhances CTL killing via extracellular Ca^{2+} influx, and does not affect $CD8^+$ T cell viability, differentiation, proliferation or cytokine production. **(A)** Murine CTLs on day 6–8 post stimulation were loaded with Calcium Sensor Dye eFluor™ 514, stained with anti-CD8 and anti-CD3e and calcium (Ca^{2+}) flux was analysed by flow cytometry. After 30 s, FPL 64176 or carrier solution was added to a final concentration of 10 µM. After 8 min, ionomycin was added to measure maximal Ca^{2+} flux. Data shown is representative of $n = 4$ biological replicates from 4 independent experiments. **(B)** On day 7–8 post stimulation, murine CTLs were co-cultured with ovalbumin-pulsed EL-4 target cells at the indicated effector to target ratios in the presence of 10 µM FPL64176 and/or 1.25 mM BAPTA and their respective carrier controls and subjected to an LDH cytotoxicity assay for 3 h. $n = 4$ biological replicates from 4 independent experiments normalised to killing of carrier-treated cells at a 10:1 effector:target cell ratio. Error bars indicate SD. *p* values were calculated using a two-way ANOVA with E:T ratio and treatment as factors with Dunnett's multiple comparisons test comparing each treatment condition ($p = 0.0195$). **(C–E)** Naive $CD8^+$ T cells were stimulated with plate-bound anti-CD3/CD28 antibodies for 24 h in the presence of 10 µM FPL64176 or carrier control before flow cytometric analysis. Bars represent the mean; error bars indicate SD. **(C)** Quantitative analysis of live cell numbers normalised to carrier $n = 4$ biological replicates from 2 independent experiments. Statistical significance was assessed using a two-tailed one sample t test. **(D)** Percentage of apoptotic cells as detected by Apotracker™ Green staining $n = 4$ biological replicates from 2 independent experiments. Statistical significance was assessed using a two-tailed paired Student's t test. **(E)** Analysis of T cell subsets based on the expression of CD44 and CD62L: $CD44^-$ $CD62L^+$ (naive), $CD44^+$ $CD62L^+$ (CM = central memory), and $CD44^+$ $CD62L^-$ (EM = effector memory). $n = 4$ biological replicates from 2 independent experiments. Statistical significance was assessed using a two-way ANOVA with Sidak's multiple comparison test. **(F, G)** CTLs were restimulated on day 10 with plate-bound anti-CD3ε antibody for 24 h in the presence of 10 µM FPL64176 or carrier control before flow cytometric analysis. Quantitative analysis of live cell numbers normalised to carrier condition **(F)** and percentage of apoptotic cells as detected by Apotracker™ Green staining **(G)** are shown. $n = 4$ biological replicates from 2 independent experiments. Bars represent the mean; error bars indicate SD. Statistical significance was assessed using a two-tailed one sample t test **(F)** or two-tailed paired Student's t test **(G)**. **(H, I)** Between day 11 and 13 post stimulation, human CTLs were left unstimulated or were re-stimulated with 1 µg/mL plate-bound anti-CD3ε antibodies for 4.5 h in the presence of 10 µM FPL64176 or carrier control. IFN-γ and TNF-α production was assessed by intracellular cytokine staining. Representative flow cytometry plots (top) and quantification of percentages and median fluorescence intensity (MFI) (bottom) are shown. $n = 4$ individual healthy donors from 2 independent experiments. Symbols indicate individual healthy donors. Bars represent the mean and error bars indicate SD. Statistical significance was assessed using a two-tailed paired Student's t test. **p* < 0.05. ns = not significant.

 © The Author(s)

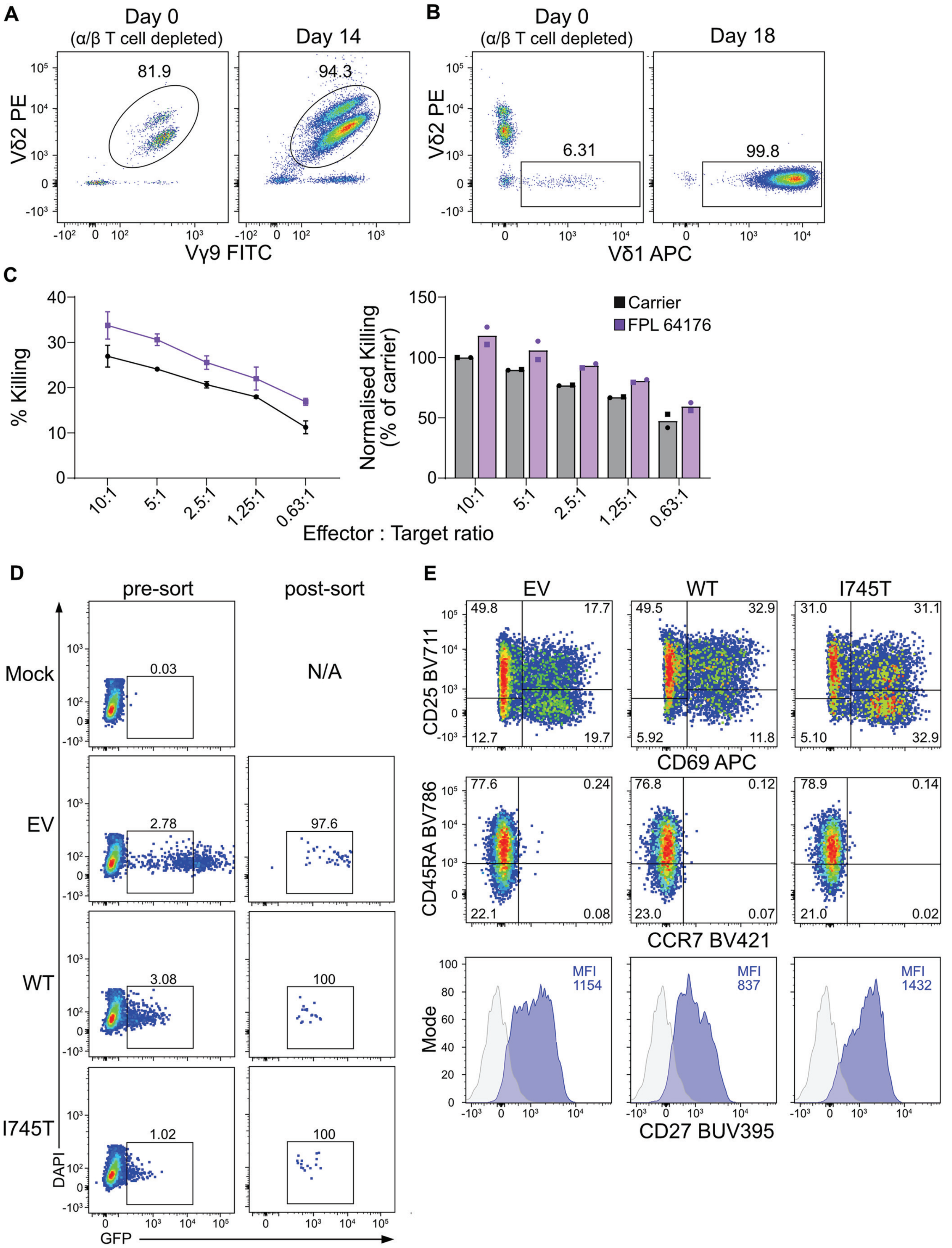
A
Day 0
(α/β T cell depleted)
Day 14
81.9
94.3
Vδ2 PE
Vγ9 FITC
B
Day 0
(α/β T cell depleted)
Day 18
6.31
99.8
Vδ2 PE
Vδ1 APC
C
% Killing
Normalised Killing
(% of carrier)
Carrier
FPL 64176
10:1
5:1
2.5:1
1.25:1
0.63:1
Effector : Target ratio
D
pre-sort
post-sort
Mock
0.03
N/A
EV
2.78
97.6
WT
3.08
100
I745T
1.02
100
DAPI
GFP
E
EV
WT
I745T
CD25 BV711
CD69 APC
49.8
17.7
12.7
19.7
49.5
32.9
5.92
11.8
31.0
31.1
5.10
32.9
CD45RA BV786
CCR7 BV421
77.6
0.24
22.1
0.08
76.8
0.12
23.0
0.07
78.9
0.14
21.0
0.02
Mode
CD27 BUV395
MFI
1154
MFI
837
MFI
1432

Figure EV5. Purity of expanded human Vγ9Vδ2^{+} and Vδ1^{+} T cells, effect of FPL 64176 on the killing capacity of Vδ1^{+} cells, purity and characterisation of human CD8^{+} T cells expressing $Ca_v1.4$ constructs.

(**A–C**) L-type voltage-gated Ca^{2+} channel agonist FPL 64176 enhances the killing capability of Vδ1^{+} T lymphocytes. (**A**) Human Vγ9Vδ2^{+} cells were expanded from αβ T cell-depleted PBMCs using 1 μM zoledronate before checking purity by flow cytometry on day 14. (**B**) Human Vδ1^{+} cells were expanded from αβ T cell-depleted PBMCs using anti-CD3ε antibodies and cytokines before checking purity by flow cytometry on day 18. (**C**) On day 22, human Vδ1^{+} T cells were co-cultured with P815 target cells at indicated effector to target ratios for 3 h and subjected to a flow cytometry-based cytotoxicity assay in the presence of 10 μM FPL 64176 or carrier control. Left panel shows a representative killing assay. Data points represent mean of two technical replicates; error bars indicate SD. Right panel shows quantification of $n = 2$ biological replicates from 1 independent experiment normalised to killing of carrier-treated cells at a 10:1 effector:target ratio. Bars represent the mean. (**D**, **E**) Sorting of human CD8^{+} T cells expressing $Ca_v1.4$ constructs and characterization of activation and differentiation markers. (**D**) Human CTLs were electroporated with PiggyBac vectors encoding GFP only (EV), human $Ca_v1.4$ (WT), or human $Ca_v1.4$ harbouring the gain-of-function mutation I745T (I745T) 48 h post stimulation and sorted between d17–28 of CTL culture based on GFP expression levels. Representative flow cytometry plots pre- and post-sorting are shown. $n = 7$ biological replicates from 5 independent experiments. (**E**) Sorted human CTLs expressing EV, WT, or I745T were restimulated for 72 h on day 19–20 of culture using plate-bound anti-CD3ε before being analysed by flow cytometry for activation and differentiation markers on day 28. Top Expression of CD25 and CD69 (top panel), CCR7 and CD45RA (middle panel) and CD27 (bottom panel) are shown. FMO control is shown in grey. Data shown is from $n = 2$ healthy donors.

 © The Author(s)
