# Supplementary material for: L-type voltage-gated Ca2+ channels control T cell killing via non-canonical Hedgehog signalling
Source: EMBO Rep. 2026 Jun 8;27(13):3689–730. doi: 10.1038/s44319-026-00810-8 (PMC13354577; doi:10.1038/s44319-026-00810-8)
Supplement: Supplementary file 2 — Peer Review File [file 44319_2026_810_MOESM2_ESM.pdf]

EMBO reports

L-type voltage-gated Ca2+ channels control T cell killing via non-canonical Hedgehog signalling

Flavio Beke, Joachim Hanna, Chrysa Kapeni, Louise M. O'Brien, Valentina Carbonaro, Nico Mueller, Sophie Trotter, Chandra Chilamakuri, and Maike de la Roche

Corresponding author(s): Maike de la Roche (maike.delaroche@cruk.cam.ac.uk)

Review Timeline:

Submission Date:	1st Feb 26
Editorial Decision:	13th Mar 26
Revision Received:	9th Apr 26
Editorial Decision:	28th Apr 26
Revision Received:	7th May 26
Accepted:	18th May 26

Editor: Achim Breiling

Transaction Report: This manuscript was submitted to EMBO Reports after peer review at another journal.

(Note: With the exception of the correction of typographical or spelling errors that could be a source of ambiguity, letters and reports are not edited. Depending on transfer agreements, referee reports obtained elsewhere may or may not be included in this compilation. Referee reports are anonymous unless the Referee chooses to sign their reports.)

Dear Dr. de la Roche,

Thank you for the submission of your manuscript to EMBO reports. As you know (I already forwarded the report), I have received input on your manuscript reviewed at a venue outside EMBO press from an arbitrator, which can also be found at the end of this email. The arbitrator indicates that most points raised by the referees have been well addressed during the revisions, and that the manuscript should be published in EMBO reports after further minor revisions. Going through your provisional point-by-point response (revision plan), I see that the remaining minor concerns of the arbitrator will be adequately addressed.

I thus invite you to revise your manuscript further as indicated in your revision plan. Revised manuscripts should be submitted within three months of a request for revision. Please contact me to discuss the revision (also by video chat) if you have questions or comments regarding the revision, or should you need additional time.

Moreover, the manuscript needs formatting to our journal style. Please carefully review the instructions that follow below.

PLEASE NOTE THAT upon resubmission revised manuscripts are subjected to an initial quality control prior to exposition to re-review. Upon failure in the initial quality control, the manuscripts are sent back to the authors, which may lead to delays. Frequent reasons for such a failure are the lack of the data availability section (please see below) and the presence of statistics based on n=2 (the authors are then asked to present scatter plots or provide more data points).

When submitting your revised manuscript, we will require:

1) a .docx formatted version of the final manuscript text (including legends for main figures, EV figures and tables), but without the figures included. Figure legends should be compiled at the end of the manuscript text.

2) individual production quality figure files as .eps, .tif, .jpg (one file per figure), of main figures and EV figures. Please upload these as separate, individual files upon re-submission.

The Expanded View format, which will be displayed in the main HTML of the paper in a collapsible format, has replaced the Supplementary information. You can submit up to 6 images as Expanded View. Please follow the nomenclature Figure EV1, Figure EV2 etc. The figure legend for these should be included in the main manuscript document file in a section called Expanded View Figure Legends after the main Figure Legends section. Additional Supplementary material should be supplied as a single pdf file labeled Appendix. The Appendix should have page numbers and needs to include a table of content on the first page (with page numbers) and legends for all content. Please follow the nomenclature Appendix Figure Sx, Appendix Table Sx etc. throughout the text, and also label the figures and tables according to this nomenclature.

For more details, please refer to our guide to authors:
https://link.springer.com/journal/44319/submission-guidelines#cms-Revised-submissions

Please consult our guide for figure preparation:
https://media.springernature.com/original/springer-cms/rest/v1/content/27825798/data/v1

3) a .docx formatted letter INCLUDING the report of the arbitrator and your detailed point-by-point response. As part of the EMBO Press transparent editorial process, the point-by-point response is part of the Review Process File (RPF), which will be published alongside your paper.

4) a complete author checklist, which you can download from our author guidelines (https://link.springer.com/journal/44319/submission-guidelines#cms-Revised-submissions). Please insert page numbers in the checklist to indicate where the requested information can be found in the manuscript. The completed author checklist will also be part of the RPF.

Please also follow our guidelines for the use of living organisms, and the respective reporting guidelines:
https://link.springer.com/partners/embo-press/editorial-policies#Animal%20and%20human%20studies

5) that primary datasets produced in this study (e.g. RNA-seq, ChIP-seq, structural and array data) are deposited in an appropriate public database. If no primary datasets have been deposited, please also state this in a dedicated section (e.g. 'No primary datasets have been generated and deposited'), see below.

See also: https://link.springer.com/partners/embo-press/editorial-policies#Data%20deposition

Please remember to provide a reviewer password if the datasets are not yet public.

The accession numbers and database should be listed in a formal "Data Availability" section that follows the model below. This

is now mandatory (like the COI statement). Please note that the Data Availability Section is restricted to new primary data that are part of this study. This section is mandatory. As indicated above, if no primary datasets have been deposited, please state this in this section

Data availability

The datasets produced in this study are available in the following databases:

- RNA-Seq data: Gene Expression Omnibus GSE46843 (https://www.ncbi.nlm.nih.gov/geo/query/acc.cgi?acc=GSE46843)
- [data type]: [name of the resource] [accession number/identifier/doi] ([URL or identifiers.org/DATABASE:ACCESSION])

*** Note - All links should resolve to a page where the data can be accessed. ***

See also: https://link.springer.com/partners/embo-press/editorial-policies#Data%20availability%20statement

Moreover, I have these editorial requests:

6) We now request the publication of original source data with the aim of making primary data more accessible and transparent to the reader. You will receive a separate email with instructions for providing source data with your revised manuscript, including information how to upload and organize the files.

7) Our journal encourages inclusion of *data citations in the reference list* to directly cite datasets that were re-used and obtained from public databases. Data citations in the article text are distinct from normal bibliographical citations and should directly link to the database records from which the data can be accessed. In the main text, data citations are formatted as follows: "Data ref: Smith et al, 2001" or "Data ref: NCBI Sequence Read Archive PRJNA342805, 2017". In the Reference list, data citations must be labeled with "[DATASET]". A data reference must provide the database name, accession number/identifiers and a resolvable link to the landing page from which the data can be accessed at the end of the reference. Further instructions are available at: https://link.springer.com/journal/44319/submission-guidelines#cms-Reference-guidelines

8) Regarding data quantification and statistics, please make sure that the number "n" for how many independent experiments were performed, their nature (biological versus technical replicates), the bars and error bars (e.g. SEM, SD) and the test used to calculate p-values is indicated in the respective figure legends (also for EV and Appendix figures). Please also check that all the p-values are explained in the legend, and that these fit to those shown in the figure. Please provide statistical testing where applicable. Please avoid the phrase 'independent experiment' but clearly state if these were biological or technical replicates. Please also indicate (e.g. with n.s.) if testing was performed, but the differences are not significant. In case n=2, please show the data as separate datapoints without error bars and statistics. See also:
https://link.springer.com/journal/44319/submission-guidelines#cms-Figure-and-data-presentation

If n<5, please show single datapoints for diagrams.

9) Please add scale bars of similar style and thickness to microscopic images, using clearly visible black or white bars (depending on the background). Please place these in the lower right corner of the images themselves. Please do not write on or near the bars in the image but define the size in the respective figure legend.

10) Please also note our reference format:
https://link.springer.com/journal/44319/submission-guidelines#cms-Reference-guidelines

11) We updated our journal's competing interests policy in January 2022 and request authors to consider both actual and perceived competing interests. Please name this section 'Disclosure and Competing Interests Statement' and put it after the Acknowledgements section.
See: https://link.springer.com/partners/embo-press/editorial-policies#Competing%20interest%20disclosures

12) We now use CRediT to specify the contributions of each author in the journal submission system. CRediT replaces the author contribution section. Please use the free text box to provide more detailed descriptions and do NOT provide your final manuscript text file with an author contributions section. See also our guide to authors (section 'Author contributions'):
https://link.springer.com/journal/44319/submission-guidelines#cms-Revised-submissions

13) All Materials and Methods need to be described in the main text using our 'Structured Methods' format, which is required for all research articles. According to this format, the Methods section should include a Reagents and Tools Table (listing key reagents, experimental models, software, and relevant equipment and including their sources and relevant identifiers), uploaded as separate file, and a Methods section in which we encourage the authors to describe their methods using a step-by-step protocol format with bullet points, to facilitate the adoption of the methodologies across labs. More information on how to adhere to this format as well as downloadable templates (.doc) for the Reagents and Tools Table can be found in our author guidelines (section 'Structured Methods'):

https://link.springer.com/journal/44319/submission-guidelines#cms-Manuscript-organisation-and-formatting

Please add all primer or antibody information directly to the Reagents and Tools table.

14) Please add up to five keywords and order the manuscript sections like this, using only these names:
Title page - Abstract (max. 175 words) - Keywords (max. 5) - Introduction - Results - Discussion - Methods - Data availability section - Acknowledgements (please include here all the funding information) - Disclosure and Competing Interests Statement - References - Figure legends - Expanded View Figure legends

15) Please make sure that all the funding information is also entered into the online submission system and that it is complete and similar to the one in the acknowledgement section of the manuscript text file.
In addition, I would need from you uploaded separately:
- a short, two-sentence summary of the manuscript (not more than 35 words).
- two to four short (!) bullet points highlighting the key findings of your study (two lines each).
- a schematic summary figure as separate file that provides a sketch of the major findings (not a data image) in jpeg or tiff format (with the exact width of 550 pixels and a height of not more than 400 pixels) that can be used as a visual synopsis on our website.

We would also welcome the submission of cover suggestions, or motifs to be used by our Graphics Illustrator in designing a cover.

As part of the EMBO publication's Transparent Editorial Process, EMBO reports publishes online a Review Process File (RPF) to accompany accepted manuscripts. This File will be published in conjunction with your paper and will include the referee reports, your point-by-point response and all pertinent correspondence relating to the manuscript.

You are able to opt out of this by letting the editorial office know (emboreports@embo.org). If you do opt out, the Review Process File link will point to the following statement: "No Review Process File is available with this article, as the authors have chosen not to make the review process public in this case."

I look forward to seeing a revised form of your manuscript when it is ready.

Best,

Achim Breiling
Senior Editor
EMBO Reports

Referee #1:

The manuscript "L-type voltage-gated Ca2+ channels control T cell killing via non-canonical Hedgehog signaling" examines the role of Cav1calcium channels on cytotoxic T lymphocyte (CTL) killing capacity via induction of Gli1.

The central claim of the manuscript is that it reveals a novel non-canonical Hedgehog pathway whereby extracellular Ca2+ influx through Cav1 channels induces the Hedgehog transcription factor Gli1 during TCR engagement which is 'essential' for subsequent CTL killing activity in vitro and in vivo. This is reported to be independent of previously reported canonical Hedgehog signalling (in CTL) and MAPK signalling (in other cell types) pathways impacts.

The key findings/observations required to support the central paper claim are that:

1) Gli1 regulates CTL killing activity
2) Extracellular calcium signalling is important for Gli1 mRNA induction during TCR engagement
3) Cav1 family calcium channels specifically are key contributors to Gli1 mRNA induction upon TCR engagement in naïve T cells and CTL
4) Cav1 inhibition reduces CTL killing activity. Cav1 agonist enhances CTL killing activity.
5) Cav1 effect on killing is mediated specifically via Gli1 induction during TCR engagement
6) TCR-mediated induction of Gli1 is not driven by canonical Hedgehog signalling
7) Cav1 induction of Gli1 during TCR engagement is independent of MAPK signalling

Supporting information that are less central to the proposed novel signalling mechanism are:
8) The localization of Cav1 in endosomes near the TCR
9) That the human genetic Cav1.4 variant impacts CTL killing activity

Here I assess whether reviewer concerns directly relevant to the central evidence / major paper conclusions are adequately addressed in the revised manuscript and author rebuttal/appeal, and whether the overall major findings/conclusions of the manuscript are technically sound.

For Round 1 Reviews:
Reviewer 1 comment/response beginning with "-Fig 2C-D, authors conclude that MAPK pathway...."

Although reviewer 1 did not bring this up themselves in their round 2 comments I thought this response to their original concern was inadequate.

If inhibitor effects are variable after 3 hrs in CTL (which in and of itself seems to be a result?) then why not look at the effects of blocking NFAT or NF-kB after 15 hrs to compare between all three at a timepoint where the MAPK inhibitor data is consistent? Many other CTL Gli1 mRNA assessments throughout the paper were only on longer timepoints so the ~15 hr post-TCR activation time point is pertinent.

To note, while not raised by the original reviewers, there also exists a similar discrepancy for the conclusion that there is a difference between the MAPK role in the naïve T cells vs CTL (i.e. that MAPK impacts Gli1 mRNA induction in CTL but not in naïve T cells). The naïve TCR engagement +/- MEKi was 6 hrs, for the CTL this was 15 hrs. The implication from the author response above is that MAPK doesn't actually have a consistent impact in CTL after only 3 hrs, not dissimilar to what is seen for the inhibitor in the naïve T cells after 6 hrs (Fig2D). This leaves open the question of whether MAPK signalling could play a role in Gli induction in both situations after a longer TCR engagement time.

To be clear either result would not negate an independent quantitative impact of Cav1 on Gli1 mRNA increase during TCR triggering, and as such are not required to support the novel mechanism proposed in the manuscript. However, in the current discussion the authors are highlighting this difference in MAPK dependence (paragraph around lines 815, 820) and discussing that typical CTL TCR engagement times are short so they need more than one mechanism to induce while naïve TCR engagement with APCs are longer so can just rely on Cav1, as a possible reason for the MAPK requirement differences - the aCD3 stimulation experimental system they've used here is almost the complete opposite of this situation and this particular conclusion is not well founded due to the stimulation time not being particularly comparable.

I believe all other round 1 review questions from reviewers 1, 2 and 3 were adequately addressed

For Round 2 of reviews:

1)
Reviewers 1&2 had a number of concerns about effect sizes in the KO and GOF mutant CTL - the Cav1.3/1,4 dKO in vivo killing assay were new to the revised text based on editor and reviewer requests. I think the authors have addressed these suitably in their appeal response. While the effects are modest they are measurable and statistically pass the significance threshold defining them as unlikely to be due to chance alone. I would suggest that the authors might want to more clearly highlight the caveat of potential redundancy in the Cav1 family in the CRISPR double KO model in their results/discussion. I, similar to the reviewers, was underwhelmed and less likely to rate the pathway as important due to smaller effect sizes in the in vivo dKO and the in vitro GOF experiments. The potential role for redundancy with Cav1.2 in the dKO system was easy to miss upon my manuscript readthrough as redundancy was only mentioned in the context of the single KOs having no effect. This smaller effect size in the dKO/GOF is not a problem for the validity of the mechanism proposed by the paper, but clearer acknowledgement and discussion of why the dKO versus inhibitor results differ could help readers better interpret the relative contribution of this pathway to overall T cell killing function.

On a related note to this, the authors state more than once throughout the text that this Cav1/Gli signalling mechanism is "essential" or "critical" or "required" etc. for CTL killing. All of their current evidence suggests that this mechanism quantitatively contributes to killing function, but that a substantial amount of killing activity remains when e.g. Gli1 is KOed, thus demonstrating that it is not "essential". The authors should consider toning down their language to more accurately reflect their findings.

2)
In relation to
Reviewer 1 comment/response beginning with "The reliance on pharmacological inhibitors..."
Reviewer 2 comment/response beginning with "Moreover, the authors do not show the effect of GANT61 on T cell viability"

The authors are correct that the inhibitor viability information for the CTL is in the manuscript and these were not raised during the initial round of reviews. However, these subsequent concerns from both reviewers on the inhibitors in the killing assay and on viability effects do highlight that this is important data for the conclusions made in the manuscript (even though they failed to raise these originally).

With regards the response here - as far as I can see there is not technically a viability measurement made for the targets cells only treated with the inhibitors (where inhibitor was present during killing assay) however, the conditions with the low number of

CTL relative to the targets largely suffice for this.

More importantly for the question of whether any of the multiple inhibitors used in the manuscript have cell viability effects there remains a substantial technical concern for experiments where viability assessment was performed by flow cytometry. In the supplementary graphs (S1G, S2E) authors show viability % in CTL cultures were somewhere in the ballpark of 97-98% in quite a few conditions - this extremely high % viability seems unrealistic, indeed using a different assessment method (Fig S2D) the authors are reporting 70-90% viability for the same types of CTL cultures.

A likely possibility for the flow cytometry data here is that there has been FSC/SSC pre-gating around a mostly viable cell population prior to when the viability dye+/- gates were set - this looks likely in Fig S2E where a sharp FSC lower limit is clearly visible indicating some degree of FSC thresholding has already taken place. All viability data assessed by flow cytometry in the manuscript should be checked and if required Fig S1G, S2E replaced with an updated analysis. I would also be keen to see bar charts showing biological replication of inhibitor effects for Fig S2E in addition to the exemplar dot plots.

This is hopefully a minor point that is easily fixed, however if one or more of the inhibitors are having a differential impact on CTL viability, depending on the inhibitor, the manuscript would need to be re-assessed to check if these differences impact strength of support for manuscript conclusions.

All other concerns from reviewers 1 and 2 from round 2 of review have been adequately addressed.

To note, I do not have any expertise in sub-cellular localization microscopy. By eye, I agree with reviewer 2 in the round 2 of comments that I'm not very sure from the T cell images about all the localization conclusions, but I also have no expertise in this so can't substantively comment on the question of endosome vs plasma membrane localization in the response/revision. For the major mechanism proposed in the manuscript this localization conclusion is not essential.

Additional thoughts on solidity of data/conclusions:
For almost all of the most important central findings for this signalling mechanism there is more than one line of evidence (e.g. KO and inhibitor) to support the conclusion, thus key findings that Cav1 is involved in Gli1 induction (independent of canonical Hedgehog and MAPK) and impacts CTL killing is solidly supported.

The only central point that does not have evidence by more than one method is the Gli1-dependence of Cav1 effect on killing, which is entirely dependent on Nifedipine (Nif) treatment of Gli1KO mouse CTL in a killing assay (Fig 4A) for functional testing. This individual experiment is still clear evidence in support of the mechanism proposed, and as such this conclusion is also supported.

Apart from the points discussed above I think the authors have suitably addressed the previous reviewer concerns and their proposed mechanism is solidly support by the manuscript data.

I have a few additional pieces of minor feedback:

1) Kotturi et al 2003, PMID 12954628 should be cited for the Nifedipine dose selection in the text (as should other relevant literature of calcium transport inhibitors at line 325). Nif is a pretty central tool used throughout the text and while the authors have shown the impact on Ca^{2+} flux at 100 µM, they haven't shown extensive validation data on dose chose. 50 and 100 µM instinctively 'felt' like high doses to me, but upon looking at Kotturi et al it became clearer why these doses were chosen and that they were suitable. This reference does open up the questions of whether the effects of Nif observed here are entirely based on extracellular calcium flux since in human T cells, Kotturi reported that Nif had a titratable impact on Ca^{2+} in the absence of extracellular calcium (see Kotturi et al 2003, PMID 12954628 Fig 3D). A bit of additional explanation/discussion/referencing of dose choices and whether Nif could be impacting intracellular calcium flux as well would be helpful.
2) Even though the Gli1KO human CTL didn't work (see review Fig 4) why not do a combination treatment with GANT61 and Nif to doubly support this point in human T cells instead? I would have thought that this experiment should show similar results to the mouse KO +/- Nif system?
3) Fig 6D doesn't have any statistics shown - is induction of Gli1 mRNA really reduced by Nif in human CD8 T cells (line ~493-4)? The data looks quite noisy.
4) I think Section 355 Fig reference to 3E should be 3G and S2F should be S2E?
5) I think Section 410 - Supp figure 2J-K should be 2H-I?

Manuscript number: EMBOR-2026-63766V1

Arbitrator:

The manuscript "L-type voltage-gated Ca2+ channels control T cell killing via non-canonical Hedgehog signaling" examines the role of Cav1calcium channels on cytotoxic T lymphocyte (CTL) killing capacity via induction of Gli1.

The central claim of the manuscript is that it reveals a novel non-canonical Hedgehog pathway whereby extracellular Ca2+ influx through Cav1 channels induces the Hedgehog transcription factor Gli1 during TCR engagement which is 'essential' for subsequent CTL killing activity in vitro and in vivo. This is reported to be independent of previously reported canonical Hedgehog signalling (in CTL) and MAPK signalling (in other cell types) pathways impacts.

The key findings/observations required to support the central paper claim are that:
1) Gli1 regulates CTL killing activity
2) Extracellular calcium signalling is important for Gli1 mRNA induction during TCR engagement
3) Cav1 family calcium channels specifically are key contributors to Gli1 mRNA induction upon TCR engagement in naïve T cells and CTL
4) Cav1 inhibition reduces CTL killing activity. Cav1 agonist enhances CTL killing activity.
5) Cav1 effect on killing is mediated specifically via Gli1 induction during TCR engagement
6) TCR-mediated induction of Gli1 is not driven by canonical Hedgehog signalling
7) Cav1 induction of Gli1 during TCR engagement is independent of MAPK signalling

Supporting information that are less central to the proposed novel signalling mechanism are:
8) The localization of Cav1 in endosomes near the TCR
9) That the human genetic Cav1.4 variant impacts CTL killing activity

Here I assess whether reviewer concerns directly relevant to the central evidence / major paper conclusions are adequately addressed in the revised manuscript and author rebuttal/appeal, and whether the overall major findings/conclusions of the manuscript are technically sound.

For Round 1 Reviews:
Reviewer 1 comment/response beginning with "-Fig 2C-D, authors conclude that MAPK pathway...."

Although reviewer 1 did not bring this up themselves in their round 2 comments I thought this response to their original concern was inadequate.

If inhibitor effects are variable after 3 hrs in CTL (which in and of itself seems to be a result?) then why not look at the effects of blocking NFAT or NF-kB after 15 hrs to compare between all three at a timepoint where the MAPK inhibitor data is consistent? Many other CTL Gli1 mRNA assessments throughout the paper were only on longer timepoints so the ~15 hr post-TCR activation time point is pertinent.

To note, while not raised by the original reviewers, there also exists a similar discrepancy for the conclusion that there is a difference between the MAPK role in the naïve T cells vs CTL (i.e. that MAPK impacts Gli1 mRNA induction in CTL but not in naïve T cells). The naïve TCR engagement +/- MEKi was 6 hrs, for the CTL this was 15 hrs. The implication from the author response above is that MAPK doesn't actually have a consistent impact in CTL after only 3 hrs, not dissimilar to what is seen for the inhibitor in the naïve T cells after 6 hrs (Fig2D). This leaves open the question of whether MAPK signalling could play a role in Gli induction in both situations after a longer TCR engagement

time.
To be clear either result would not negate an independent quantitative impact of Cav1 on Gli1 mRNA increase during TCR triggering, and as such are not required to support the novel mechanism proposed in the manuscript. However, in the current discussion the authors are highlighting this difference in MAPK dependence (paragraph around lines 815, 820) and discussing that typical CTL TCR engagement times are short so they need more than one mechanism to induce while naïve TCR engagement with APCs are longer so can just rely on Cav1, as a possible reason for the MAPK requirement differences - the aCD3 stimulation experimental system they've used here is almost the complete opposite of this situation and this particular conclusion is not well founded due to the stimulation time not being particularly comparable.
We agree with the arbitrator. We have repeated the experiment in naïve CD8⁺ T cells using the MAPK inhibitor and stimulated the cells for 15h. The results are very similar to the 6h timepoint We have included the new data in the manuscript for consistency. Please see below.

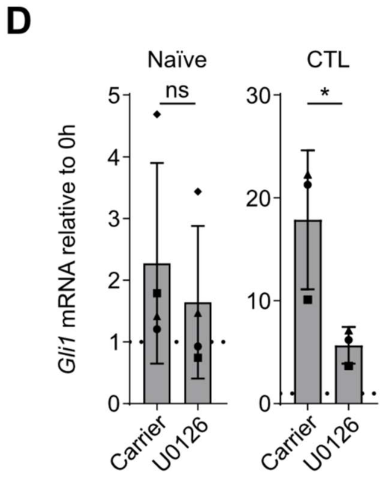

Figure 2D: MAPK signalling regulates *Gli1* induction in CTLs but not in naïve T cells. Naïve CD8⁺ T cells were stimulated for 15h with plate-bound anti-CD3/CD28 antibodies in the presence of 10µM U0126 or carrier control (left). CTLs were restimulated for 15h with plate-bound anti-CD3ε in the presence of 10µM U0126 or carrier control (right) before RNA was extracted for qRT-PCR analysis. n=4 (left panel) or n=3 (right panel) biological replicates from 2 and 3 independent experiments, respectively. Data is normalized to *Tbp* as a reference gene. Similar results were obtained when *CD3ε* was used as a reference gene. Error bars indicate SD. p values were calculated using an unpaired two-tailed Student's t test.

I believe all other round 1 review questions from reviewers 1, 2 and 3 were adequately addressed

For Round 2 of reviews:
1)Reviewers 1&2 had a number of concerns about effect sizes in the KO and GOF mutant CTL - the Cav1.3/1,4 dKO in vivo killing assay were new to the revised text based on editor and reviewer requests. I think the authors have addressed these suitably in their appeal response. While the effects are modest they are measurable and statistically pass the significance threshold defining them as unlikely to be due to chance alone. I would suggest that the authors might want to more clearly highlight the caveat of potential redundancy in the Cav1 family in the CRISPR double KO model in their results/discussion.
I, similar to the reviewers, was underwhelmed and less likely to rate the pathway as important due to smaller effect sizes in the in vivo dKO and the in vitro GOF experiments. The potential role for redundancy with Cav1.2 in the dKO system was easy to miss upon my manuscript readthrough as redundancy was only mentioned in the context of the single KOs having no effect. This smaller effect size in the dKO/GOF is not a problem for the validity of the mechanism proposed by the paper, but

clearer acknowledgement and discussion of why the dKO versus inhibitor results differ could help readers better interpret the relative contribution of this pathway to overall T cell killing function.
We agree with the arbitrator and have highlighted the redundancy of Cav1 channels in the results and discussion sections (line 336-338 and 548-552).

On a related note to this, the authors state more than once throughout the text that this Cav1/Gli signalling mechanism is "essential" or "critical" or "required" etc. for CTL killing. All of their current evidence suggests that this mechanism quantitatively contributes to killing function, but that a substantial amount of killing activity remains when e.g. Gli1 is KOed, thus demonstrating that it is not "essential". The authors should consider toning down their language to more accurately reflect their findings.
We agree with the arbitrator and have now toned down the language throughout the manuscript.

2)In relation to
Reviewer 1 comment/response beginning with "The reliance on pharmacological inhibitors..."
Reviewer 2 comment/response beginning with "Moreover, the authors do not show the effect of GANT61 on T cell viability"

The authors are correct that the inhibitor viability information for the CTL is in the manuscript and these were not raised during the initial round of reviews. However, these subsequent concerns from both reviewers on the inhibitors in the killing assay and on viability effects do highlight that this is important data for the conclusions made in the manuscript (even though they failed to raise these originally).

With regards the response here - as far as I can see there is not technically a viability measurement made for the targets cells only treated with the inhibitors (where inhibitor was present during killing assay) however, the conditions with the low number of CTL relative to the targets largely suffice for this.
We apologise for not making this point clear. All our killing assays (LDH-based and flowcytometry-based) contain target cells +/- inhibitor controls to specifically monitor effects of the inhibitors on the target cells. We do not see an effect of the inhibitors on the target cells within the timeframe of the assay. We have now included this information in the materials and methods.
Furthermore, we have previously done in depth analysis of the effects of the inhibitors and Ca_v1 channel agonist on our target cell lines used in the study (EL-4 and P815) with regards to viability, MHCI and CD32 expression. We included these data below:

(Note that even though we show GANT61 effects on target cells here, GANT61 is not present in our killing assays but only the CTLs are pre-incubated with it.)

Figure for referee with unpublished data and its description has been removed upon request by the authors.

More importantly for the question of whether any of the multiple inhibitors used in the manuscript have cell viability effects there remains a substantial technical concern for experiments where viability assessment was performed by flow cytometry. In the supplementary graphs (S1G, S2E) authors show viability % in CTL cultures were somewhere in the ballpark of 97-98% in quite a few conditions - this

extremely high % viability seems unrealistic, indeed using a different assessment method (Fig S2D) the authors are reporting 70-90% viability for the same types of CTL cultures.
We thank the arbitrator for picking up on this. In fact, the graphs in Suppl. Fig 1G,2E are gated on the lymphocyte gate in the FSC/SCC plot. We apologise for this and now show viability of the ungated populations. Please see below.

G

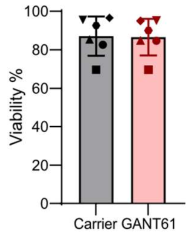

NEW FigEV1G: GANT61 pre-incubation does not affect human CTL viability.
Viability of human CTLs was determined by flow cytometry after treatment with 5mM GANT61 for 8-18 hours on day 12-14 post stimulation. n=6 biological replicates from 2 independent experiments. Symbols indicate individual human donors. Error bars indicate SD.

E

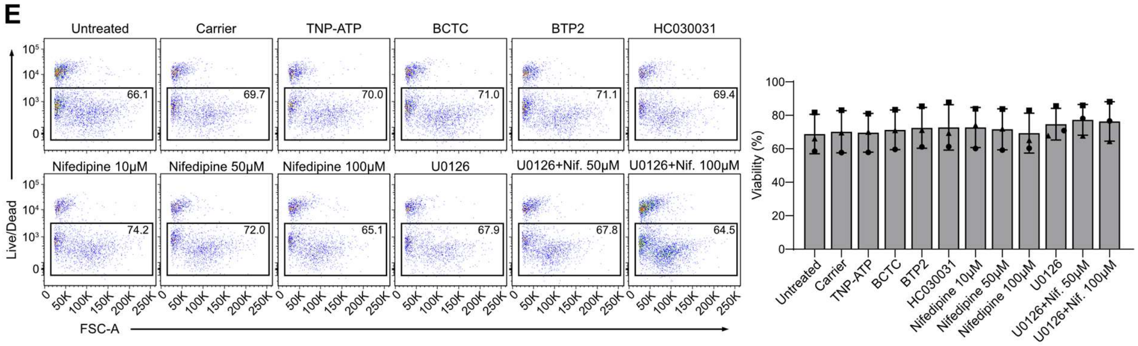

NEW FigEV2E: Ca^{2+} channel inhibitors do not affect murine CTL viability upon restimulation.
Murine CTLs were restimulated with plate-bound anti-CD3e in the presence of the indicated Ca2+ channel inhibitors or carrier control for 15h before flowcytometric analysis for cell viability. n=3 biological replicates from 3 independent experiments. Symbols indicate biological replicates. Error bars indicate SD.

A likely possibility for the flow cytometry data here is that there has been FSC/SSC pre-gating around a mostly viable cell population prior to when the viability dye+/- gates were set - this looks likely in Fig S2E where a sharp FSC lower limit is clearly visible indicating some degree of FSC thresholding has already taken place. All viability data assessed by flow cytometry in the manuscript should be checked and if required Fig S1G, S2E replaced with an updated analysis. I would also be keen to see bar charts showing biological replication of inhibitor effects for Fig S2E in addition to the exemplar dot plots.
We now show biological repeats for Fig S2E and updated the figure legend accordingly (please see above).

This is hopefully a minor point that is easily fixed, however if one or more of the inhibitors are having a differential impact on CTL viability, depending on the inhibitor, the manuscript would need to be re-assessed to check if these differences impact strength of support for manuscript conclusions.

All other concerns from reviewers 1 and 2 from round 2 of review have been adequately addressed.

To note, I do not have any expertise in sub-cellular localization microscopy. By eye, I agree with reviewer 2 in the round 2 of comments that I'm not very sure from the T cell images about all the

localization conclusions, but I also have no expertise in this so can't substantively comment on the question of endosome vs plasma membrane localization in the response/revision. For the major mechanism proposed in the manuscript this localization conclusion is not essential.

Additional thoughts on solidity of data/conclusions:
For almost all of the most important central findings for this signalling mechanism there is more than one line of evidence (e.g. KO and inhibitor) to support the conclusion, thus key findings that Cav1 is involved in Gli1 induction (independent of canonical Hedgehog and MAPK) and impacts CTL killing is solidly supported.

The only central point that does not have evidence by more than one method is the Gli1-dependence of Cav1 effect on killing, which is entirely dependent on Nifedipine (Nif) treatment of Gli1KO mouse CTL in a killing assay (Fig 4A) for functional testing. This individual experiment is still clear evidence in support of the mechanism proposed, and as such this conclusion is also supported.

Apart from the points discussed above I think the authors have suitably addressed the previous reviewer concerns and their proposed mechanism is solidly support by the manuscript data.

I have a few additional pieces of minor feedback:

1) Kotturi et al 2003, PMID 12954628 should be cited for the Nifedipine dose selection in the text (as should other relevant literature of calcium transport inhibitors at line 325). Nif is a pretty central tool used throughout the text and while the authors have shown the impact on Ca2+ flux at 100 µM, they haven't shown extensive validation data on dose chose. 50 and 100 µM instinctively 'felt' like high doses to me, but upon looking at Kotturi et al it became clearer why these doses were chosen and that they were suitable. This reference does open up the questions of whether the effects of Nif observed here are entirely based on extracellular calcium flux since in human T cells, Kotturi reported that Nif had a titratable impact on Ca2+ in the absence of extracellular calcium (see Kotturi et al 2003, PMID 12954628 Fig 3D). A bit of additional explanation/discussion/referencing of dose choices and whether Nif could be impacting intracellular calcium flux as well would be helpful.
We have included these insightful suggestions in the manuscript text (lines 248-249 and 552-555).
2) Even though the Gli1KO human CTL didn't work (see review Fig 4) why not do a combination treatment with GANT61 and Nif to doubly support this point in human T cells instead? I would have thought that this experiment should show similar results to the mouse KO +/- Nif system?
We think that we have shown this more elegantly in our murine genetic knockout model (*Gli1* KO) + nifedipine (see Fig. 4A).

3) Fig 6D doesn't have any statistics shown - is induction of Gli1 mRNA really reduced by Nif in human CD8 T cells (line ~493-4)? The data looks quite noisy.
We thank the arbitrator for picking up on this. We now show the statistics.

4) I think Section 355 Fig reference to 3E should be 3G and S2F should be S2E?
We thank the arbitrator for picking this up. We have now corrected this.

5) I think Section 410 - Supp figure 2J-K should be 2H-I?
We thank the arbitrator for picking this up. We have now corrected this.

Dear Dr. de la Roche,

Thank you for the submission of your revised manuscript to our editorial offices. I have now received the report from the arbitrator that was asked to re-evaluate the study, you will find below. As you will see, the arbitrator supports the publication of the study in EMBO reports. However, the arbitrator has a final point I ask you to address in a final revised manuscript.

Moreover, I have the editorial requests below I also ask you to address. Please also provide a final p-b-p-response regarding the remaining point of the arbitrator and the editorial requests.

Editorial requests:

- Please make sure that the number "n" for how many independent experiments were performed, their nature (biological versus technical replicates), the bars and error bars (e.g. SEM, SD) and the test used to calculate p-values is indicated in the respective figure legends (main and EV figures). Please also check that all the p-values are explained in the legend, and that these fit to those shown in the figure. Please provide statistical testing where applicable. Please avoid the phrase 'independent experiment' but clearly state if these were biological or technical replicates. Please also indicate (e.g. with n.s.) if testing was performed, but the differences are not significant. In case n=2, please show the data as separate datapoints without error bars and statistics. See also:

https://link.springer.com/journal/44319/submission-guidelines#cms-Figure-and-data-presentation

If n<5, please show single datapoints for diagrams. Presently, some diagrams lack statistics or the 'n.s.'. Please provide proper and complete statistical testing for all diagrams shown (except for those with n=2). Moreover:
- Please note that the error bars are not defined in the legends of figures 1G, 3A, 7A-D; 8A, EV3 H
- Please note that the measure of center for the error bars needs to be defined in the legends of figures 1B, F, I, J; 2B-D; 3B, D, G, I; 4A, B, C, D, E; 5A, B, H; 6A, B, C, D, G; 9C-E; 10B, C; EV1 B, C, E-G; EV2 D, E, F, G, H, I; EV3 A-D; EV4 B, C, D, E, F, G, I; EV5 C, D.
- Please note that the exact p values are not provided in the legends of figures 1C, E, F, J; 2D, 3A, B, D, F, H, I; 4E, 5B, D, F, G, H; 6F, G; 8A-D, F; 9A, C, E; 10B, EV2 G, H, I; EV3 B
- Please note that the white arrow heads 7A-C, EV2 E, F are not defined in the legend of figure. This needs to be rectified.

- Please make sure that all the funding information is also entered into the online submission system and that it is complete and similar to the one in the acknowledgement section of the manuscript text file. Grants from the Cambridge School of Clinical Medicine and Frank Edward Elmore Fund are missing in the submission system (unless these go under 1954837 grant number and funder). Please check.

- There is a callout to "Suppl. Table 1" in the legend of Fig. 8. Does this refer to one of the Appendix figures (then please put the correct callout), or to a table in Winklmayr et al, 2010?

- Please remove the instructions text form the Reagents & Tools table.

In addition, I would need from you uploaded separately:
- a short, two-sentence summary of the manuscript (not more than 35 words).
- two to four short (!) bullet points highlighting the key findings of your study (two lines each).
- a schematic summary figure as separate file that provides a sketch of the major findings (not a data image) in jpeg or tiff format (with the exact width of 550 pixels and the exact height of 300 pixels) that can be used as a visual synopsis on our website.

I look forward to seeing the further revised version of your manuscript when it is ready. Please let me know if you have questions regarding the revision.

I look forward to seeing a new revised version of your manuscript as soon as possible.

Best,

Achim

Achim Breiling
Senior Editor
EMBO Reports

Arbitrator:

The authors have addressed my most major concern satisfactorily with the update to Fig EV1G and EV2E.

1) There is still an issue that requires revision in Fig 6D / line 384. The statistics now included in 6D don't show a statistically significant impact of Nif on Gli1 expression in human T cells but the text says "but GLI1 induction was moderately reduced in nifedipine-treated human CD8+ T cells (Fig. 6D)." This is not accurate as to what the data is showing - two strong outliers are making the average bar charts look like there might be a decrease, but actually the data is pretty noisy and doesn't seem to show a clear consistent pattern, with a number of replicates showing Nif reduces Gli1, a number of replicates shown Nif increase Gli1 and a number of replicates showing not much difference between Nif/carrier treatment.

The time point used here is actually very different to the ones being used to look at Gli1 mRNA in mouse cells and to assess Nif impact on human CTL calcium flux and killing in the subsequent Fig 6E-G. The manuscript either needs to be modified to accurately state what this data shows (that at 2 days post-activation there is no impact of Nif on Gli1 mRNA) or additional data is required assessing a more suitable condition/timepoint i.e look at Gli1 mRNA in TCR stimulated human CTL where a difference in Gli1 mRNA would be expected to be measurable.

2) There was also one non-essential comment that was left unaddressed.

Reviewer #1 comment beginning with "-Fig 2C-D, authors conclude that MAPK pathway...." and my comment "Although reviewer 1 did not bring this up themselves in their round 2 comments I thought this response to their original concern was inadequate. If inhibitor effects are variable after 3 hrs in CTL (which in and of itself seems to be a result?) then why not look at the effects of blocking NFAT or NF-kB after 15 hrs to compare between all three at a timepoint where the MAPK inhibitor data is consistent? Many other CTL Gli1 mRNA assessments throughout the paper were only on longer timepoints so the ~15 hr post-TCR activation time point is pertinent...."

This assessment at a later time point for nucleofected CTL to examine NFAT or NFkB signalling has not been performed (though the 15 hr time point for MAPKi on naïve cells, which was also part of my feedback on this point has been in revised Fig 2D). These specific experiments are not essential to the novel mechanism proposed in the manuscript and the data from the experiments performed is suitably discussed in the text so I have no issue with these not being part of the revised text.

Beke & Hanna et al. – point-by-point response

Manuscript number: EMBOR-2026-63766V1

Dear Dr. Breiling,

We thank the arbitrator and the editorial team for the insightful review of our manuscript. Please find below our final *point-by-point* response to the remaining point raised by the arbitrator and the editorial requests.

Editorial requests:

- Please make sure that the number "n" for how many independent experiments were performed, their nature (biological versus technical replicates), the bars and error bars (e.g. SEM, SD) and the test used to calculate p-values is indicated in the respective figure legends (main and EV figures). Please also check that all the p-values are explained in the legend, and that these fit to those shown in the figure. Please provide statistical testing where applicable. Please avoid the phrase 'independent experiment' but clearly state if these were biological or technical replicates. Please also indicate (e.g. with n.s.) if testing was performed, but the differences are not significant. In case n=2, please show the data as separate datapoints without error bars and statistics.

If n<5, please show single datapoints for diagrams. Presently, some diagrams lack statistics or the 'n.s.'. Please provide proper and complete statistical testing for all diagrams shown (except for those with n=2).
We have now addressed these points.

Moreover:
- Please note that the error bars are not defined in the legends of figures 1G, 3A, 7A-D; 8A, EV3 H
- Please note that the measure of center for the error bars needs to be defined in the legends of figures 1B, F, I, J; 2B-D; 3B, D, G, I; 4A, B, C, D, E; 5A, B, H; 6A, B, C, D, G; 9C-E; 10B, C; EV1 B, C, E-G; EV2 D, E, F, G, H, I; EV3 A-D; EV4 B, C, D, E, F, G, I; EV5 C, D.
- Please note that the exact p values are not provided in the legends of figures 1C, E, F, J; 2D, 3A, B, D, F, H, I; 4E, 5B, D, F, G, H; 6F, G; 8A-D, F; 9A, C, E; 10B, EV2 G, H, I; EV3 B
- Please note that the white arrow heads 7A-C, EV2 E, F are not defined in the legend of figure. This needs to be rectified.
We have now rectified this.

- Please make sure that all the funding information is also entered into the online submission system and that it is complete and similar to the one in the acknowledgement section of the manuscript text file. Grants from the Cambridge School of Clinical Medicine and Frank Edward Elmore Fund are missing in the submission system (unless these go under 1954837 grant number and funder). Please check. The grant goes under grant number 1954837 and the respective funder in the submission sytem.

- There is a callout to "Suppl. Table 1" in the legend of Fig. 8. Does this refer to one of the Appendix figures (then please put the correct callout), or to a table in Winklmayr et al, 2010? We have rectified this in the legend of Figure 8 (callout is for Appendix Table S8).

- Please remove the instructions text form the Reagents & Tools table. Done.

In addition, I would need from you uploaded separately:
- a short, two-sentence summary of the manuscript (not more than 35 words). Done.
- two to four short (!) bullet points highlighting the key findings of your study (two lines each). Done.
- a schematic summary figure as separate file that provides a sketch of the major findings (not a data image) in jpeg or tiff format (with the exact width of 550 pixels and the exact height of 300 pixels) that can be used as a visual synopsis on our website. Done.

Arbitrator:

The authors have addressed my most major concern satisfactorily with the update to Fig EV1G and EV2E.

1) There is still an issue that requires revision in Fig 6D / line 384. The statistics now included in 6D don't show a statistically significant impact of Nif on Gli1 expression in human T cells but the text says "but GLI1 induction was moderately reduced in nifedipine-treated human CD8+ T cells (Fig. 6D)." This is not accurate as to what the data is showing - two strong outliers are making the average bar charts look like there might be a decrease, but actually the data is pretty noisy and doesn't seem to show a clear consistent pattern, with a number of replicates showing Nif reduces Gli1, a number of replicates shown Nif increase Gli1 and a number of replicates showing not much difference between Nif/carrier treatment.
The time point used here is actually very different to the ones being used to look at Gli1 mRNA in mouse cells and to assess Nif impact on human CTL calcium flux and killing in the subsequent Fig 6E-G. The manuscript either needs to be modified to accurately state what this data shows (that at 2 days post-activation there is no impact of Nif on Gli1 mRNA) or additional data is required assessing a more suitable condition/timepoint i.e look at Gli1 mRNA in TCR stimulated human CTL where a difference in Gli1 mRNA would be expected to be measurable.
We thank the arbitrator for picking this up and agree with the overall assessment. We identified 3 problems with this set of experiments: (1) 3 days after stimulation is not the right timepoint to assess peak GLI1 mRNA expression in $CD8^+$ T cells, (2) Nifedipine half-life in tissue culture was not taken into account, (3) Variability in naïve $CD8^+$ T cell quality after isolation (mainly due to age and storage of buffy coat which is out of our control) and consequent variability of stimulation efficiency and kinetics.
We have thus taken out Figure 6D from the manuscript.

2) There was also one non-essential comment that was left unaddressed.

Reviewer #1 comment beginning with "-Fig 2C-D, authors conclude that MAPK pathway...." and my comment "Although reviewer 1 did not bring this up themselves in their round 2 comments I thought this response to their original concern was inadequate. If inhibitor effects are variable after 3 hrs in CTL (which in and of itself seems to be a result?) then why not look at the effects of blocking NFAT or NF-kB after 15 hrs to compare between all three

at a timepoint where the MAPK inhibitor data is consistent? Many other CTL Gli1 mRNA assessments throughout the paper were only on longer timepoints so the ~15 hr post-TCR activation time point is pertinent...."

This assessment at a later time point for nucleofected CTL to examine NFAT or NFkB signalling has not been performed (though the 15 hr time point for MAPKi on naïve cells, which was also part of my feedback on this point has been in revised Fig 2D).

Gli1 mRNA in CTLs becomes upregulated already at 3h post re-stimulation and *Gli1* upregulation further increases over the course of the next 20hours. Thus, effects can be observed over a period of time. Cell numbers can be an issue and restrict the amount of timepoints that can be assessed.

These specific experiments are not essential to the novel mechanism proposed in the manuscript and the data from the experiments performed is suitably discussed in the text so I have no issue with these not being part of the revised text.

We agree with the overall assessment by the arbitrator.

Dr. Maike de la Roche
University of Cambridge
CRUK Cambridge Institute
Li Ka Shing Centre
Robinson Way
Cambridge, Cambridgeshire CB2 0RE
United Kingdom

Dear Dr. de la Roche,

Thank you for the submission of your final revised manuscript to our editorial offices. I now went through this and your final p-b-p-response and consider the remaining points of the arbitrator and the editorial requests as adequately addressed.

I am thus very pleased to accept your manuscript for publication in the next available issue of EMBO reports. Thank you for your contribution to our journal.

Your manuscript will be processed for publication by EMBO Press. It will be copy edited and you will receive page proofs prior to publication. Please note that you will be contacted by Springer Nature Author Services to complete licensing and payment information.

You may qualify for financial assistance for your publication charges - either via a Springer Nature fully open access agreement or an EMBO initiative. Check your eligibility: https://link.springer.com/journal/44319/how-to-publish-with-us

Should you be planning a Press Release on your article, please get in contact with embo_production@springernature.com as early as possible in order to coordinate publication and release dates.

If you have any questions, please do not hesitate to contact the Editorial Office. Thank you for your contribution to EMBO Reports.

Yours sincerely,

Achim Breiling
Senior Editor
EMBO Reports

>>> Please note that it is EMBO Reports policy for the transcript of the editorial process (containing referee reports and your response letter) to be published as an online supplement to each paper. If you do NOT want this, you will need to inform the Editorial Office via email immediately. More information is available here: https://link.springer.com/partners/embo-press/editorial-policies#Peer%20review

EMBO Press Author Checklist

Corresponding Author Name: Dr Maike de la Roche	
Journal Submitted to: EMBO Reports	
Manuscript Number: EMBOR-2026-63766	

USEFUL LINKS FOR COMPLETING THIS FORM
[The EMBO Journal - Author Guidelines]
[EMBO Reports - Author Guidelines]
[Molecular Systems Biology - Author Guidelines]
[EMBO Molecular Medicine - Author Guidelines]

Reporting Checklist for Life Science Articles (updated January
This checklist is adapted from Materials Design Analysis Reporting (MDAR) Checklist for Authors. MDAR establishes a minimum set of requirements in transparent reporting in the life sciences (see Statement of Task: 10.31222/osf.io/9sm4x). Please follow the journal's guidelines in preparing your
Please note that a copy of this checklist will be published alongside your article.

Abridged guidelines for figures
1. Data
The data shown in figures should satisfy the following conditions:
- ➞ the data were obtained and processed according to the field's best practice and are presented to reflect the results of the experiments in an accurate and unbiased manner.
- ➞ ideally, figure panels should include only measurements that are directly comparable to each other and obtained with the same assay.
- ➞ plots include clearly labeled error bars for independent experiments and sample sizes. Unless justified, error bars should not be shown for technical
- ➞ if n<5, the individual data points from each experiment should be plotted. Any statistical test employed should be justified.
- ➞ Source Data should be included to report the data underlying figures according to the guidelines set out in the authorship guidelines on Data

2. Captions
Each figure caption should contain the following information, for each panel where they are relevant:
- ➞ a specification of the experimental system investigated (eg cell line, species name).
- ➞ the assay(s) and method(s) used to carry out the reported observations and measurements.
- ➞ an explicit mention of the biological and chemical entity(ies) that are being measured.
- ➞ an explicit mention of the biological and chemical entity(ies) that are altered/varied/perturbed in a controlled manner.
- ➞ the exact sample size (n) for each experimental group/condition, given as a number, not a range;
- ➞ a description of the sample collection allowing the reader to understand whether the samples represent technical or biological replicates (including how many animals, litters, cultures, etc.).
- ➞ a statement of how many times the experiment shown was independently replicated in the laboratory.
- ➞ definitions of statistical methods and measures:
  - common tests, such as t-test (please specify whether paired vs. unpaired), simple $\chi 2$ tests, Wilcoxon and Mann-Whitney tests, can be unambiguously identified by name only, but more complex techniques should be described in the methods section;
  - are tests one-sided or two-sided?
  - are there adjustments for multiple comparisons?
  - exact statistical test results, e.g., P values = x but not P values < x;
  - definition of 'center values' as median or average;
  - definition of error bars as s.d. or s.e.m.

Please complete ALL of the questions below.
Select "Not Applicable" only when the requested information is not relevant for your study.

Materials

Newly Created Materials	Information included in the manuscript?	In which section is the information available? (Reagents and Tools Table, Materials and Methods, Figures, Data Availability Section)
New materials and reagents need to be available; do any restrictions apply?	Yes	Reagents and Tools Table, Matherials and Methods

Antibodies	Information included in the manuscript?	In which section is the information available? (Reagents and Tools Table, Materials and Methods, Figures, Data Availability Section)
For **antibodies** provide the following information:<br>- Commercial antibodies: RRID (if possible) or supplier name, catalogue number and or/clone number<br>- Non-commercial: RRID or citation	Yes	Matherials and Methods & Appendix

DNA and RNA sequences	Information included in the manuscript?	In which section is the information available? (Reagents and Tools Table, Materials and Methods, Figures, Data Availability Section)
**Short novel DNA or RNA including primers, probes**: provide the sequences.	Yes	Reagents and Tools Table, Materials and Methods

Cell materials	Information included in the manuscript?	In which section is the information available? (Reagents and Tools Table, Materials and Methods, Figures, Data Availability Section)
**Cell lines:** Provide species information, strain. Provide accession number in repository **OR** supplier name, catalog number, clone number, and/**OR** RRID.	Yes	Reagents and Tools Table, Materials and Methods
**Primary cultures:** Provide species, strain, sex of origin, genetic modification status.	Yes	Materials and Methods
Report if the cell lines were recently **authenticated** (e.g., by STR profiling) and tested for mycoplasma contamination.	Yes	Materials and Methods

Experimental animals	Information included in the manuscript?	In which section is the information available? (Reagents and Tools Table, Materials and Methods, Figures, Data Availability Section)
**Laboratory animals or Model organisms:** Provide species, strain, sex, age, genetic modification status. Provide accession number in repository **OR** supplier name, catalog number, clone number, **OR** RRID.	Yes	Reagents and Tools Table, Materials and Methods
**Animal observed in or captured from the field:** Provide species, sex, and age where possible.	Not Applicable	
Please detail **housing and husbandry conditions**.	Yes	Materials and Methods

Plants and microbes	Information included in the manuscript?	In which section is the information available? (Reagents and Tools Table, Materials and Methods, Figures, Data Availability Section)
**Plants:** provide species and strain, ecotype and cultivar where relevant, unique accession number if available, and source (including location for collected wild specimens).	Not Applicable	
**Microbes:** provide species and strain, unique accession number if available, and source.	Yes	Reagents and Tools Table, Materials and Methods

Human research participants	Information included in the manuscript?	In which section is the information available? (Reagents and Tools Table, Materials and Methods, Figures, Data Availability Section)
If collected and within the bounds of privacy constraints report on age, sex and gender or ethnicity for all study participants.	Not Applicable	

Core facilities	Information included in the manuscript?	In which section is the information available? (Reagents and Tools Table, Materials and Methods, Figures, Data Availability Section)
If your work benefited from core facilities, was their service mentioned in the acknowledgments section?	Yes	Acknowledgements

Design

Study protocol	Information included in the manuscript?	In which section is the information available? (Reagents and Tools Table, Materials and Methods, Figures, Data Availability Section)
If study protocol has been **pre-registered, provide DOI in the manuscript**. For clinical trials, provide the trial registration number **OR** cite DOI.	Not Applicable	
Report the **clinical trial registration number** (at ClinicalTrials.gov or equivalent), where applicable.	Not Applicable	

Laboratory protocol	Information included in the manuscript?	In which section is the information available? (Reagents and Tools Table, Materials and Methods, Figures, Data Availability Section)
Provide DOI OR other citation details if **external detailed step-by-step protocols** are available.	Not Applicable	

Experimental study design and statistics	Information included in the manuscript?	In which section is the information available? (Reagents and Tools Table, Materials and Methods, Figures, Data Availability Section)
Include a statement about **sample size** estimate even if no statistical methods were used.	Yes	Materials and Methods
Were any steps taken to minimize the effects of subjective bias when allocating animals/samples to treatment (e.g. **randomization procedure**)? If yes, have they been described?	Yes	Materials and Methods
Include a statement about **blinding** even if no blinding was done.	Yes	Materials and Methods
Describe **inclusion/exclusion criteria** if samples or animals were excluded from the analysis. Were the criteria pre-established?<br><br>If sample or data points were omitted from analysis, report if this was due to attrition or intentional exclusion and provide justification.	Not Applicable	
For every figure, are **statistical tests** justified as appropriate? Do the data meet the assumptions of the tests (e.g., normal distribution)? Describe any methods used to assess it. Is there an estimate of variation within each group of data? Is the variance similar between the groups that are being statistically compared?	Yes	Figure legends, Materials and Methods

Sample definition and in-laboratory replication	Information included in the manuscript?	In which section is the information available? (Reagents and Tools Table, Materials and Methods, Figures, Data Availability Section)
In the figure legends: state number of times the experiment was **replicated** in laboratory.	Yes	Figure legends
In the figure legends: define whether data describe **technical or biological replicates**.	Yes	Figure legends

Ethics

Ethics	Information included in the manuscript?	In which section is the information available? (Reagents and Tools Table, Materials and Methods, Figures, Data Availability Section)
Studies involving **human participants**: State details of **authority granting ethics approval** (IRB or equivalent committee(s), provide reference number for approval.	Yes	Materials and Methods
Studies involving **human participants**: Include a statement confirming that **informed consent** was obtained from all subjects and that the experiments conformed to the principles set out in the WMA Declaration of Helsinki and the Department of Health and Human Services Belmont Report.	Yes	Materials and Methods
Studies involving **human participants:** For publication of **patient photos**, include a statement confirming that consent to publish was obtained.	Not Applicable	
Studies involving experimental **animals**: State details of **authority granting ethics approval** (IRB or equivalent committee(s), provide reference number for approval. Include a statement of compliance with ethical regulations.	Yes	Materials and Methods
Studies involving **specimen and field samples:** State if relevant **permits** obtained, provide details of authority approving study; if none were required, explain why.	Not Applicable	

Dual Use Research of Concern (DURC)	Information included in the manuscript?	In which section is the information available? (Reagents and Tools Table, Materials and Methods, Figures, Data Availability Section)
Could your study fall under dual use research restrictions? Please check biosecurity documents and list of **select agents and toxins** (CDC): https://www.selectagents.gov/sat/list.htm	Not Applicable	
If you used a select agent, is the security level of the lab appropriate and reported in the manuscript?	Not Applicable	
If a study is subject to dual use research of concern regulations, is the name of the **authority granting approval and reference number** for the regulatory approval provided in the manuscript?	Not Applicable	

Reporting

The MDAR framework recommends adoption of discipline-specific guidelines, established and endorsed through community initiatives. Journals have their own policy about requiring specific guidelines and recommendations to complement MDAR.

Adherence to community standards	Information included in the manuscript?	In which section is the information available? (Reagents and Tools Table, Materials and Methods, Figures, Data Availability Section)
State if relevant guidelines or checklists (e.g., **ICMJE, MIBBI, ARRIVE, PRISMA**) have been followed or provided.	Not Applicable	
For **tumor marker prognostic studies,** we recommend that you follow the **REMARK** reporting guidelines (see link list at top right). See author guidelines, under 'Reporting Guidelines'. Please confirm you have followed these guidelines.	Not Applicable	
For **phase II and III randomized controlled trials**, please refer to the **CONSORT** flow diagram (see link list at top right) and submit the CONSORT checklist (see link list at top right) with your submission. See author guidelines, under 'Reporting Guidelines'. Please confirm you have submitted this list.	Not Applicable	

Data Availability

Data availability	Information included in the manuscript?	In which section is the information available? (Reagents and Tools Table, Materials and Methods, Figures, Data Availability Section)
Have **primary datasets** been deposited according to the journal's guidelines (see 'Data Deposition' section) and the respective accession numbers provided in the Data Availability Section?	Yes	Data Availability Section
Were **human clinical and genomic datasets** deposited in a public access-controlled repository in accordance to ethical obligations to the patients and to the applicable consent agreement?	Not Applicable	
Are **computational models** that are central and integral to a study available without restrictions in a machine-readable form? Were the relevant accession numbers or links provided?	Not Applicable	
If publicly available data were reused, provide the respective **data citations in the reference list**.	Yes	References
